# Functional Switch and Ethyl Group Formation in the Bacterial Polytrichastrene Synthase from *Chryseobacterium polytrichastri*

**DOI:** 10.1002/anie.202109465

**Published:** 2021-08-13

**Authors:** Anwei Hou, Bernd Goldfuss, Jeroen S. Dickschat

**Affiliations:** ^1^ Kekulé-Institute for Organic Chemistry and Biochemistry University of Bonn Gerhard-Domagk-Straße 1 53121 Bonn Germany; ^2^ Department of Chemistry University of Cologne Greinstraße 4 50939 Cologne Germany

**Keywords:** biosynthesis, enzyme mechanisms, isotopes, mutagenesis, terpenes

## Abstract

A reinvestigation of the linalool synthase from *Chryseobacterium polytrichastri* uncovered its diterpene synthase activity, yielding polytrichastrene A and polytrichastrol A with new skeletons, besides known wanju‐2,5‐diene and thunbergol. The enzyme mechanism was investigated by isotopic labeling experiments and DFT calculations to explain an unusual ethyl group formation. Rationally designed exchanges of active site residues showed major functional switches, resulting for I66F in the production of five more new compounds, including polytrichastrene B and polytrichastrol B, while A87T, A192V and the double exchange A87T, A192V gave a product shift towards wanju‐2,5‐diene.

## Graphical abstract

Linalool synthase from *Chryseobacterium polytrichastri* was found to have diterpene synthase activity and produced diterpenes with new skeletons containing a rare ethyl group. Its formation was mechanistically studied through isotopic labeling and DFT calculations. Exchange of active site residues resulted in a functional switch leading to additional novel diterpenes. This work lays the ground for future rational engineering of terpene synthases.

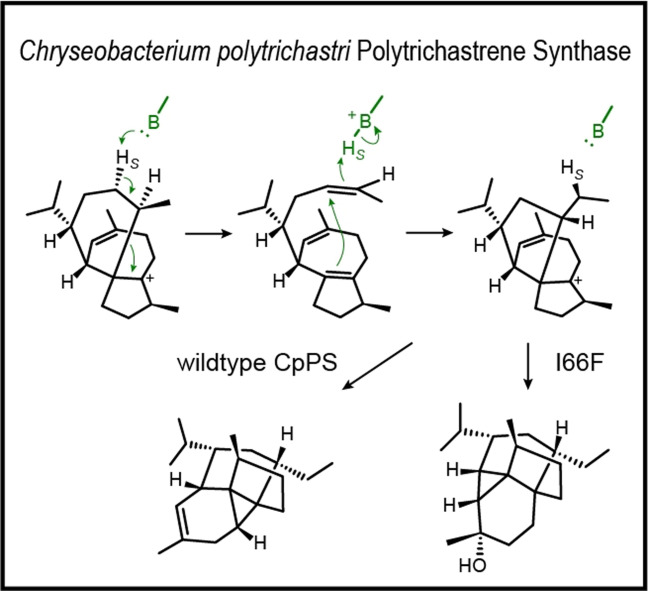

Terpenoid biosynthesis starts with the cyclization of an acyclic oligoprenyl diphosphate into a structurally complex terpene hydrocarbon, which can be followed by several functionalizations to introduce bioactivity. The cyclization step is catalyzed by a single enzyme, a terpene synthase (TPS), and proceeds by substrate ionization through abstraction of diphosphate (type I TPSs) or protonation (type II) to initiate a cationic cascade reaction.^[1]^ Among bacterial type I TPSs, several sesquiterpene synthases (STPSs)[^2^, ^3^, ^4^, ^5^] and diterpene synthases (DTPSs)[^6^, ^7^, ^8^, ^9^] have been reported, while monoterpene synthases (MTPSs) such as 1,8‐cineol synthase from *S. clavuligerus* are rare.^[10]^ The sestermobaraene synthase recently discovered from *S. mobaraensis* is the only known bacterial type I sesterterpene synthase (StTPS).^[11]^

While the overall structures of type I TPSs are highly conserved and exhibit a similar α‐helical fold as avian farnesyl diphosphate synthase,^[12]^ their amino acid sequences can be very different which challenges a sequence‐based function prediction. It has been suggested that initial cyclization modes can be predicted from phylogenetic analyses,[^13^, ^14^] and an algorithm for sequence based function predictions has been implemented into antiSMASH.^[15]^ Computational approaches to predict the product from an enzyme homology model by fitting cationic intermediates into the active site,^[16]^ or to distinguish between the functions of enzymes as MTPS, STPS or DTPS^[17]^ have been developed. A phylogenetic tree constructed from the amino acid sequences of 3278 bacterial TPS homologs reveals a scattered distribution of MTPSs, STPSs, DTPSs and StTPSs and of cyclization modes, demonstrating that a function prediction from amino acid sequences of bacterial TPSs is difficult (Figure S1). Therefore, we turned our attention to a rational approach based on the crystal structure of selina‐4(15),7(11)‐diene synthase from *S. pristinaespiralis* (SdS) in complex with the substrate surrogate 2,3‐dihydrofarnesyl diphosphate (DHFPP).^[18]^ For TPSs using large substrates (geranylfarnesyl diphosphate, GFPP for StTPSs or geranylgeranyl diphosphate, GGPP for DTPSs) the active site cavity should offer a larger space than for TPSs using small substrates (farnesyl diphosphate, FPP for STPSs or geranyl diphosphate, GPP for MTPSs).

Several non‐polar active site residues enclose DHFPP in the SdS structure that define the substrate conformation and the available space within the active site (Figure 1). Their van der Waals volumes (*V*_vdW_ in Å^3^) can be calculated according to Abraham and co‐workers (Table S1).^[19]^ A summary of the 13 residues located in the analogous positions of other characterized TPSs, grouped according to their functions from MTPSs to StTPSs, along with their added *V*_vdW_ (Σ*V*_vdw_) is given in Table S2. The spanned regions and mean ± standard deviation for each group of TPSs are summarized in Figure 2. While the bands in this analysis overlap, there is a clear trend for the size of the active site residues, as manifested by the average Σ*V*_vdw_ ranging from 907 Å^3^ for MTPSs to 733 Å^3^ for StTPSs, with an average decrease of 58 Å^3^ between two consecutive TPS groups. This value is similar to the increase of the substrate size of 84 Å^3^ for each isoprene unit (Table S1), suggesting that the active site space becomes larger according to the requirements of the substrate. We have recently described a TPS from *Chryseobacterium polytrichastri* as linalool synthase (CpLS), based on the observed activities towards different substrates in a mixed Tris/phosphate buffer.^[20]^ Analysis of its active site residues gave a Σ*V*_vdw_=550 Å^3^, which is the smallest value among all TPSs (red entry in Table S2) and did not seem to fit for a MTPS, but would rather suggest a function as DTPS or StTPS.

**Figure 1:**
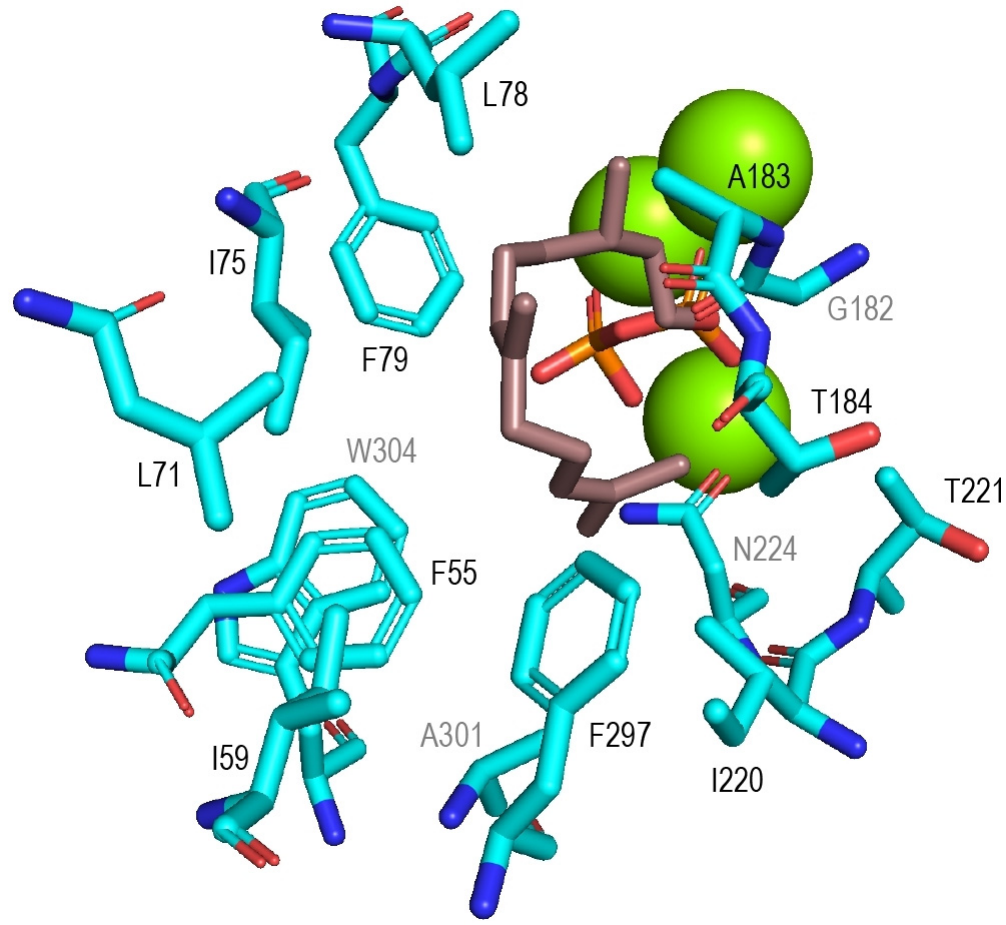
Active site of SdS showing several non‐polar residues enclosing the substrate surrogate DHFPP (brown). Black labels are for foreground residues, grey labels for background residues. Green spheres represent Mg^2+^ cations.

**Figure 2:**
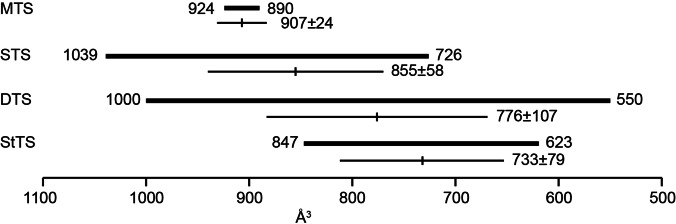
Added *V*_vdw_ (Σ*V*_vdw_) of TPS active site residues. Bold bars indicate spanned regions, thin bars show the mean ± SD for each group of TPSs.

A reinvestigation of this enzyme indeed resulted under optimized conditions with a solely Tris‐based incubation buffer in the efficient conversion of GGPP into several unknown diterpenes, while GFPP did not yield any products and GPP and FPP gave only acyclic terpenes (Figure S2). The main hydrocarbon and a minor alcohol were isolated and their structures elucidated by NMR spectroscopy (Figures S3–S18, Tables S3 and S4), resulting in polytrichastrene A (**1**) and polytrichastrol A (**2**), both featuring novel skeletons with an ethyl group (Scheme 1), which is an unusual structural element in terpenes that occurs only in a few examples as in the cleistanthane diterpenes veadeirol and veadeiroic acid.^[21]^ Furthermore, wanju‐2,5‐diene (**3**), known from the *C. wanjuense* wanju‐2,5‐diene synthase (CwWS),^[22]^ and thunbergol (**4**)^[23]^ were isolated (Figures S19–S26, Table S5). Thus, the enzyme originally described as CpLS was reassigned as *Chryseobacterium polytrichastri* Polytrichastrene Synthase (CpPS). The absolute configurations of **1** and **2** were determined by stereoselective deuteration using dimethylallyl diphosphate (DMAPP) and (*R*)‐ or (*S*)‐(1‐^13^C,1‐^2^H)isopentenyl diphosphate (IPP)^[24]^ that were converted with GGPP synthase (GGPPS) from *Streptomyces cyaneofuscatus* ^[8]^ and CpPS (Table S6, Figures S27 and S28). The relative orientations of the natural stereogenic centers to the introduced stereogenic anchors at the deuterated carbons allow to assign the absolute configurations of the terpenes. Additional experiments with DMAPP, (*E*)‐ and (*Z*)‐(4‐^13^C,4‐^2^H)IPP,^[25]^ GGPPS and CpPS (Figures S29 and S30) confirmed the absolute configurations of **1** and **2**.

**Scheme 1:**
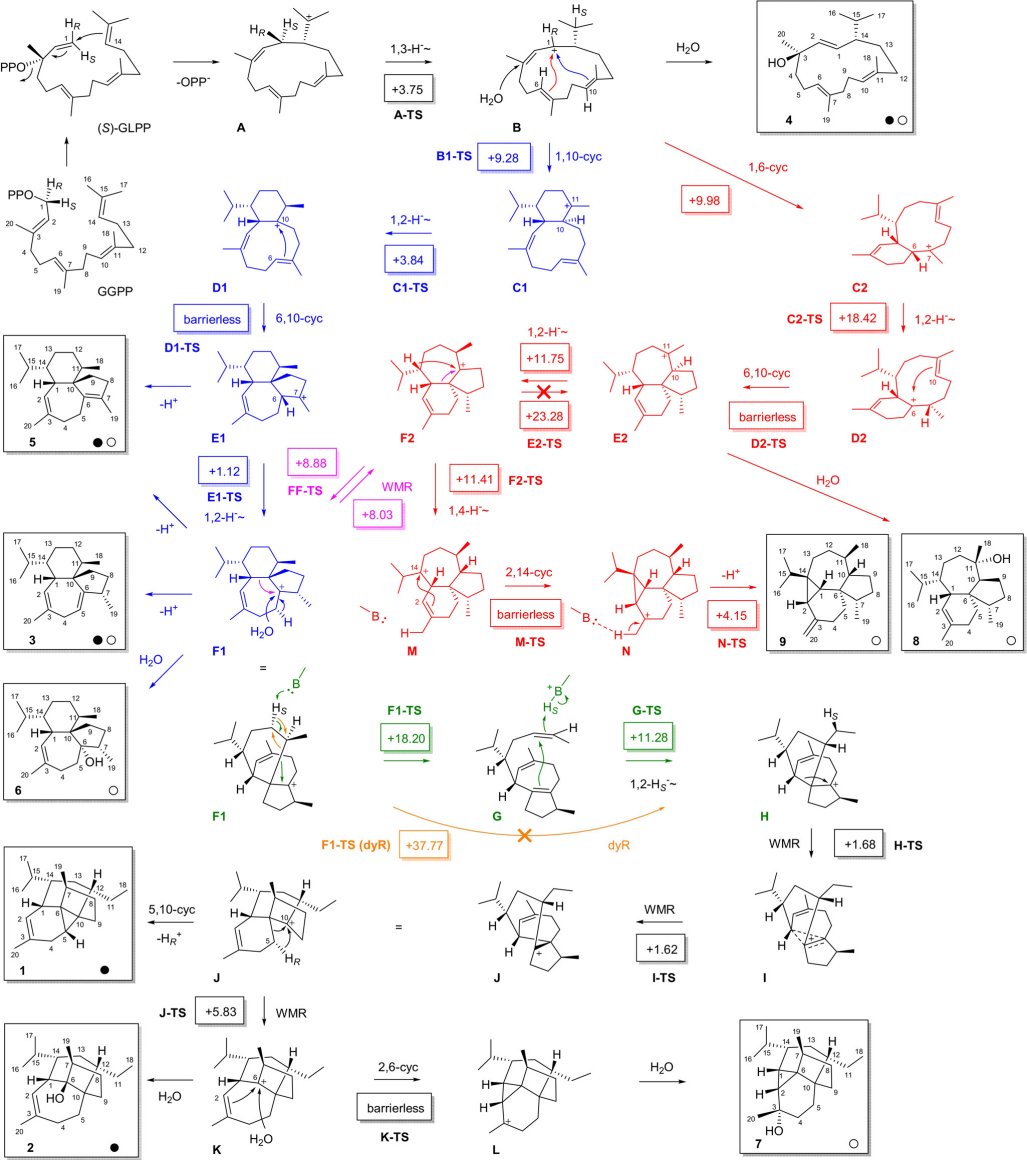
Cyclization of GGPP to **1**–**9** (dyR=dyotropic rearrangement, WMR=Wagner–Meerwein rearrangement, black dots=from CpPS, white dots=from CpPS I66F). Carbon numbering follows GGPP numbering to indicate the origin of each carbon. Numbers in boxes are transition state barriers in kcal mol^−1^ (mPW1PW91/6–311+G(d,p)//B97D3/6‐31G(d,p), with base (‐B:) in **M** and **F1**=MeNH_2_). Barriers >20 kcal mol^−1^ are considered too high (crossed arrows).

The cyclization mechanism by CpPS requires GGPP isomerization to GLPP to explain the 2*Z* double bond in **1**–**3**. Following Arigoni's mechanistic model for cadalanes,^[26]^ this isomerization proceeds through (*S*)‐GLPP by *syn*‐allylic transposition of diphosphate with a specific stereochemical fate for the 1‐*pro*‐*R* and the 1‐*pro*‐*S* hydrogen of GGPP, ending up in the 1*E* and 1*Z* positions of GLPP, respectively. After vinyl group rotation around C2‐C3 the 1,14‐cyclization continues with attack of C14 at C1 from the *Si* face (top, *anti*‐S_N_2′ reaction) to install the correct configuration at C14 in **A**, which also turns H1_*S*_ to the bottom. This hydrogen is now closer to the cation at C15 than H1_*R*_ and shifts into the *i*Pr group in **B**, as confirmed experimentally with (*R*)‐ and (*S*)‐(1‐^2^H)GGPP (Figure S31). For the enantiomers of **1**–**4** the migration of H1_*R*_ into the *i*Pr group would be expected, and thus the migration of H1_*S*_ versus H1_*R*_ can give evidence for the absolute configurations of terpenes, as demonstrated previously for other sesqui‐ and diterpenes.[^22^, ^25^, ^26^, ^27^] The optical rotation of **4** from CpPS ([*α*]_D_^25^=+35.0, *c*=0.1, CHCl_3_) discloses that this is the same enantiomer as in *Pseudotsuga menziesii* ([*α*]_D_=+74.4, *c*=6.3, CHCl_3_).^[23a]^

Cation **B** is a first branching point and can be attacked by water to yield **4**, while the steps from **B** to **F1** towards **3** (shown in blue) were supported by previous labeling experiments.^[22]^ Along these steps two 1,2‐hydride migrations from **C1** to **D1** and from **E1** to **F1** occur that were both supported for **1** with (11‐^13^C,10‐^2^H)GGPP and (7‐^13^C,6‐^2^H)GGPP, enzymatically prepared from (3‐^13^C,2‐^2^H)GPP^[28]^ and (3‐^13^C,2‐^2^H)FPP^[29]^ with IPP and GGPPS. In these experiments a deuterium atom migrates to the labeled carbon, resulting in a slightly upfield shifted triplet in the ^13^C‐NMR spectrum (Figures S32 and S33). The steps from **F1** towards **1** may include a dyotropic rearrangement^[30]^ to **H** (orange arrow) to explain the Et group formation. Stereoselective deuterations introduced at C12 using DMAPP plus (*E*)‐ or (*Z*)‐(4‐^13^C,4‐^2^H)IPP and GGPPS revealed the specific shift of the 12‐*pro*‐*S* hydrogen, while the usage of (3‐^13^C,4,4‐^2^H_2_)IPP^[11]^ demonstrated its migration to C11 (Figure S34). A subsequent Wagner–Meerwein rearrangement (WMR) leads to **J**. Deprotonation to **1** with cyclopropanation proceeds with loss of the 5‐*pro*‐*R* hydrogen, as demonstrated by stereoselective deuterium labeling introduced with (*R*)‐ and (*S*)‐(1‐^13^C,1‐^2^H)IPP (Figure S35). Another WMR from **J** results in **K**, the precursor of **2** that is formed by quenching with water. The overall mechanism towards **1**–**4** is also in line with ^13^C‐labeling experiments, in which each of the twenty carbons of GGPP were labeled individually (Figures S36–S55).

For a deeper understanding of the role of active site residues in TPS catalysis (Figure S56), several CpPS variants targeting positions homologous to SdS active site residues were constructed (Tables S7 and S8). The purified enzyme variants were adjusted to same concentration (Figure S57), followed by conversion of GGPP and GC/MS analysis of the products (Figures S58 and S59, Table S9). The position equivalent to I66 in CpPS (=F55 in SdS, Figure 1) is in many TPSs and especially DTPSs occupied by an aromatic residue (Table S2). Indeed, the I66F variant gave an enhanced diterpene production (195±43 % of wildtype level, Figure 3) and a strongly changed product profile, with only low amounts of **1**, but no **2** and slightly increased **3** and **4**. In addition, several new compounds were obtained. A preparative scale incubation allowed the isolation and structure elucidation by NMR spectroscopy (Figures S61–S100, Tables S10–S14) of wanju‐2,6‐diene (**5**), wanju‐2‐en‐6α‐ol (**6**), polytrichastrol B (**7**), bonn‐2‐en‐11α‐ol (**8**) and polytrichastrene B (**9**). Their absolute configurations were determined by analogous stereoselective deuterium labeling experiments as described above for **1**–**4**, giving uniform results for all nine compounds (Figures S101–S107).

**Figure 3:**
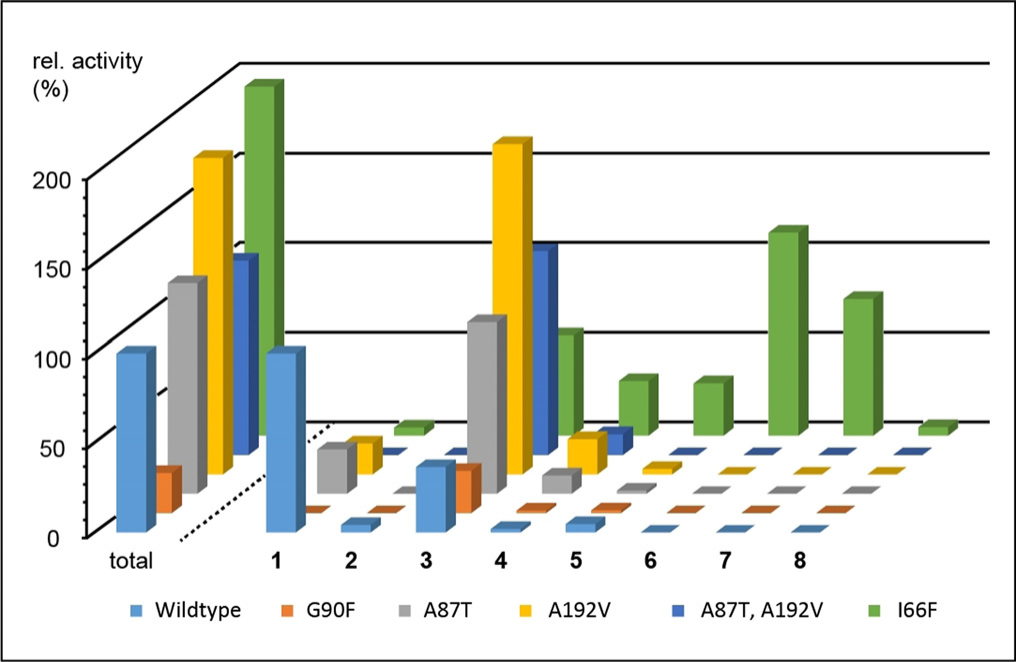
Products and relative activities of CpPS and its variants. Bars left of dashed line show relative total diterpene production (wildtype=100 %). Bars right of dashed line show relative production of **1**–**8** (**1** by wildtype=100 %). Means from triplicates, for standard deviations cf. Table S9 and Figure S60.

Alcohol **8** exhibits the skeleton of bonnadiene that is a side product of CwWS.^[22]^ The enantiomer is known from bonnadiene synthase (BdS) from *Allokutzneria albata*.^[25]^ All compounds fit well into the biosynthesis mechanism (Scheme 1), as **5** can be explained by deprotonation of **E1**, **6** can arise by water addition to **F1**, and **7** can be formed from **K** by another cyclization to **L** and capture with water. Only compounds **8** and **9** seem to follow a pathway that branches out early at **B** by 1,6‐cyclization to **C2**, 1,2‐hydride shift to **D2** and 6,10‐cyclization to **E2**, the precursor to **8** by water quenching (red pathway). A 1,4‐hydride shift from **F2** to **M**, 2,14‐cyclization and deprotonation can lead to **9**. Notably, **F2** can also be linked to **F1** through WMR (purple), so that the cyclization cascade along **B**, the red branch to **F2**, the purple link to **F1** and the downstream reactions to **L** contain all intermediates in one direct line to explain all products by simple deprotonation or attack of water (only **9** requires a short side branch via **M** and **N**). The labeling experiments performed here cannot distinguish between the blue and the red pathway, because both pathways contain analogous elementary steps, so that all substrate atoms end up in the same positions.

To distinguish between these pathways, DFT calculations were performed (Table S15, Figure S108). The 1,3‐hydride shift from **A** to **B** proceeds smoothly, ending in a **B** conformer that allows both cyclization reactions to **C1** and **C2** through similar transition state (TS) barriers (Scheme 1). The steps from **C1** to **F1** (blue) are all associated with low TS barriers or even barrierless, while the step from **C2** to **D2** has a fairly high barrier (+18.42 kcal mol^−1^). The conversions from **D2** to **F2** are more feasible, and also the purple link between **F1** and **F2** can be passed in both directions. This analysis suggests that the blue pathway may be preferred for the formation of **1**–**7**, and also **9** can be reached via this pathway and the purple link to **F2**, as the 1,4‐hydride transfer to **M** can be realized computationally. Ring closure of **M** to **N** was only possible with assistance of a base (MeNH_2_) in contact with Me20 to suppress spontaneous ring opening of **N** to **M**, ultimately leading to **9**. An alternative two‐step process with 1,3‐ and 1,2‐hydride transfer is geometrically not possible, similar to 1,3‐hydride transfers in guaiane biosynthesis that are rare and only allowed for the few stereoisomers with suitable geometry.^[31]^ Compound **8** may be the only case for which the red path is entered from **B**, because the step from **F2** to **E2** has an even higher TS than the **C2** to **D2** conversion. The high TS barriers to reach **E2** from any side may explain the observation of **8** only as a minor compound in the I66F variant.

Based on the calculations the concerted **F1** to **H** dyotropic rearrangement discussed above seems not possible (+37.77 kcal mol^−1^), but assistance by a catalytic base, for which MeNH_2_ was selected as a model (initial attempts with water or NH_3_ failed), allows a stepwise process with low barriers through deprotonation of H12_*S*_ and fragmentation to **G** followed by back transfer of the same proton to C11 and ring closure to **H**.

The SdS positions I75, L78, L79 and F297 are also often occupied by aromatic residues in DTPSs. In the corresponding positions of CpPS small (Gly, Ala) or non‐polar (Met) residues are found that were exchanged to obtain the A87F, G90F, A91F, and M308W variants. G90F showed a decreased activity (23±2 %) with main product **3**, while A87F and A91F were inactive, presumably because of steric interference with GGPP. The M308W exchange did not yield a soluble protein, suggesting a major impact on the enzyme structure.

Side product **3** of CpPS is the main product of CwWS, and these enzymes differ in their active site residues in only two positions: A87 and A192 in CpPS are taken by T86 and V191 in CwWS (Table S2). The importance of active site residues to control the product profile of a TPS is impressively demonstrated by the A87T, A192V and—with two simultaneous exchanges—the A87T,A192V enzyme variants, all of which gave **3** as main product. The A192V variant showed an increased activity (176±8 %), but also the single A87T and the double A87T,A192V exchange did not disturb substrate acceptance (117±6 % and 108±13 % activity, respectively).

In summary, we have characterized a bacterial DTPS that converts GGPP into diterpenes with novel skeletons. An unusual ethyl group formation was studied in detail by isotopic labeling experiments and DFT calculations, revealing a two‐step rather than a concerted dyotropic rearrangement likely assisted by an active site base that needs to be identified by future structural work ideally in conjunction with QM/MM simulations. We have also identified 13 active site residues that are promising candidates for functional switches within TPSs, which allows for a rational design of engineered biocatalysts in future work.

## Conflict of interest

The authors declare no conflict of interest.

## Supporting information

As a service to our authors and readers, this journal provides supporting information supplied by the authors. Such materials are peer reviewed and may be re‐organized for online delivery, but are not copy‐edited or typeset. Technical support issues arising from supporting information (other than missing files) should be addressed to the authors.

Supporting Information
