# Supplementary material for: Functional Switch and Ethyl Group Formation in the Bacterial Polytrichastrene Synthase from Chryseobacterium polytrichastri
Source: Angew Chem Int Ed Engl. 2021 Aug 13;60(38):20781–5. doi: 10.1002/anie.202109465 (PMC8518897; doi:10.1002/anie.202109465)
Supplement: Supplementary file 1 — Supporting Information [file ANIE-60-20781-s001.pdf]

Angewandte Chemie
Eine Zeitschrift der Gesellschaft Deutscher Chemiker

Supporting Information

Functional Switch and Ethyl Group Formation in the Bacterial Polytrichastrene Synthase from *Chryseobacterium polytrichastri*

*Anwei Hou, Bernd Goldfuss, and Jeroen S. Dickschat**

anie_202109465_sm_miscellaneous_information.pdf

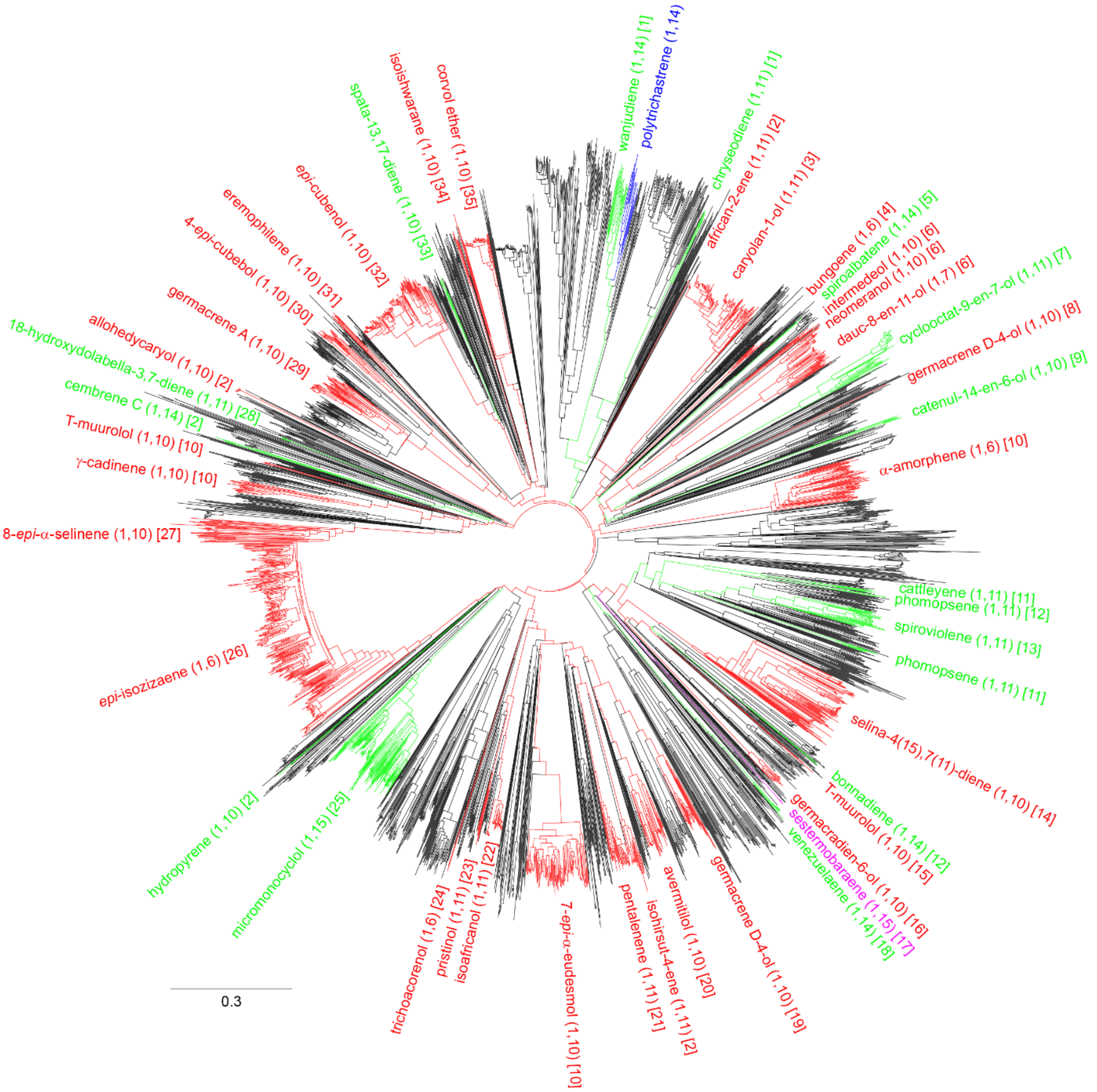

Figure S1. Phylogenetic tree constructed from 3278 amino acid sequences of bacterial terpene synthase homologs. The tree was built using the tree builder function of Geneious (alignment type: global alignment with free end gaps, cost matrix: Blosum45, genetic distance model: Jukes-Cantor, tree build method: neighbor-joining, gap open panelty: 8, gap extension penalty: 2; the bar indicates substitutions per site). Functionally characterized enzymes[1-35] (references are also indicated in the tree) and their closest relatives with likely the same function are shown in red (sesquiterpene synthases), green (diterpene synthases) and purple (sesterterpene synthases). The branch for polytrichastrene synthase characterized in this work is shown in blue. Numbers in round brackets at each enzyme indicate initial cyclization mode (n,m-cyclization).

Table S1. Calculated van der Waals volumes (Å^3) for amino acid side chains and oligoprenyl diphosphates.[36]

Group	C[a]	H	N	O	S	P	R_A	R_{NA}	N_B	V_{vdW}
Gly[b]		1							0	7.24
Ala	1	3							3	24.54
Ser	1	3		1					4	33.33
Cys[c]	1	3			1				4	43.05
Pro	3	6						1	9	48.10
Thr	2	5		1					7	50.63
Asp	2	2		2					5	55.46
Asn	2	4	1	1					7	58.99
Val	3	7							9	59.14
His[d]	4	5	2				1		11	69.90
Glu	3	4		2					8	72.76
Gln	3	6	1	1					10	76.29
Leu	4	9							12	76.44
Ile	4	9							12	76.44
Met	3	7			1				10	77.65
Lys[e]	4	11	1						15	88.76
Phe	7	7					1		14	97.16
Tyr	7	7		1			1		15	105.95
Arg	4	10	3						16	106.80
Trp	9	8	1				2		19	116.86
GPP	10	17		7		2			35	273.51
FPP	15	25		7		2			48	357.37
GGPP	20	33		7		2			61	441.23
GFPP	25	41		7		2			74	525.09

[a] Calculations according to equation (4) in ref. [36]. Herein, the numbers of C, H, N, O, S and P, of aromatic (R_A) and non-aromatic rings (R_{NA}), and of bonds (N_B) are relevant. [b] Small residues (< 40 Å^3) and substrate GPP (highlighted in green), [c] medium residues (40 – 60 Å^3) and substrate FPP (blue), [d] large residues (60 – 80 Å^3) and substrate GGPP (yellow), [e] very large residues (> 80 Å^3) and substrate GFPP (red).

Table S2. Sum of calculated van der Waals volumes (Å^3) of active site residues in characterised bacterial, fungal and protist terpene synthases.

**Enzyme**[a]	**SdS position**													**ΣV_{vdW}**
	**F55**	**I59**	**L71**	**I75**	**L78**	**F79**	**R178**	**G182**	**A183**	**T184**	**I220**	**T221**	**F297**	**(Å^3)**
**MTS**														
1,8-cineol (I) (*Streptomyces clavuligerus*)[37]	V	W	G	F	F	F	R	G	F	M	I	N	A	924
1,8-cineol (II) (*Hypoxylon* sp.)[38]	A	M	M	N	I	F	R	F	A	V	F	S	V	890
mean ± SD														907±24
**STS**														
(*Z*)-γ-bisabolene (*Cryptosporangium arvum*)[39]	L	L	L	V	M	F	R	F	A	W	M	I	L	1039
β-himachalene (*Cryptosporangium arvum*)[40]	W	I	V	T	G	F	R	M	R	L	V	T	L	961
epi-isozizaene (*Streptomyces coelicolor*)[26]	L	Y	I	S	F	F	R	F	A	H	A	A	W	951
bungoene (*Streptomyces bungoensis*)[4]	L	L	I	N	L	F	R	F	A	F	N	A	L	948
trichobrasilenol (*Trichoderma reesei*)[41]	L	Y	L	L	I	F	R	V	G	V	T	I	L	945
1-*epi*-cubenol (*Streptomyces griseus*)[32]	F	V	V	F	L	F	R	G	A	I	V	C	W	920
T-muurolol (I) (*Streptomyces clavuligerus*)[15]	L	I	F	H	L	F	R	G	A	M	V	S	W	919
10-*epi*-cubebol (*Sorangium cellulosum*)[42]	L	C	I	Y	L	F	R	S	T	M	A	S	W	919
selina-4(15),7(11)-diene (*S. pristinaespiralis*)[14]	F	I	L	I	L	F	R	G	A	T	I	T	F	914
isohirsut-4-ene (*Streptomyces lactacystinaeus*)[2]	I	F	G	Q	Y	F	R	I	G	V	I	I	T	913
presilphiperfolan-8β-ol (*Botrytis cinerea*)[43]	L	W	M	N	V	F	R	I	G	V	V	T	V	905
pentalenene (*Streptomyces exfoliatus*)[21]	L	F	G	M	F	F	R	I	G	V	N	L	V	897
pristinol (*Streptomyces pristinaespiralis*)[23]	L	F	A	N	F	F	R	I	G	V	V	I	V	896
α-acorenol (*Fusarium fujikuroi*)[44]	C	L	L	L	L	F	R	I	A	K	S	I	C	895
aristolochene (*Aspergillus terreus*)[45]	V	Y	A	L	L	F	R	V	G	K	L	S	Q	888
allohedycaryol (*Mycobacterium marinum*)[2]	L	T	M	I	L	F	R	S	A	V	V	S	W	888
neomeranol B (*Streptomyces scabiei*)[6]	F	T	L	H	I	W	R	G	A	A	V	D	W	882
δ-cadinene (*Streptomyces clavuligerus*)[15]	L	C	I	M	L	F	R	G	A	I	V	C	W	881
caryolan-1-ol (*Streptomyces griseus*)[3]	W	I	F	L	A	F	R	V	A	M	S	G	L	874
germacrene D-4-ol (I) (*Streptomyces citricolor*)[19]	L	V	A	M	Y	F	R	A	A	M	P	S	W	873
(*E*)-β-caryophyllene (*Saccharothrix espanaensis*)[30]	L	F	G	M	F	F	R	I	G	T	V	V	V	871
isoafricanol (*Streptomyces violaceusniger*)[22]	L	F	G	V	Y	F	R	I	G	V	V	V	V	870

eremophilene (I) (*Fusarium fujikuroi*)[46]	C	M	M	L	I	F	R	V	G	G	A	W	F	867
isohirsut-1-ene (*Streptomyces clavuligerus*)[2]	L	F	A	M	Y	F	R	T	S	S	I	G	L	863
isoishwarane (*Streptomyces lincolnensis*)[34]	F	T	C	L	T	F	R	V	G	M	V	A	W	866
4-*epi*-cubebol (*Streptosporangium roseum*)[30]	L	T	V	C	L	F	R	G	A	M	V	V	W	854
african-2-ene (*Streptomyces clavuligerus*)[2]	I	Y	L	M	A	W	R	S	G	E	M	G	H	853
selina-3,7(11)-diene (*Streptomyces* sp.)[2]	F	I	L	V	L	F	R	G	A	A	I	S	F	853
nerolidol (*Streptomyces clavuligerus*)[47]	I	G	C	L	T	F	R	I	C	C	V	I	F	853
trichoacorenol (*Amycolatopsis benzoatilytica*)[24]	F	N	I	V	W	S	R	G	A	V	I	N	L	852
trichodiene (*Fusarium sporotrichioides*)[48]	M	S	L	Y	T	L	R	G	L	G	M	V	F	852
asterisca-2(9),6-diene (*Dictyostelium discoideum*)[49]	L	F	G	M	F	F	R	I	G	A	I	A	M	846
protoillud-7-ene (*Dictyostelium discoideum*)[49]	L	F	G	M	F	F	R	I	G	A	T	T	L	846
guaia-6,10(14)-diene (*Fusarium fujikuroi*)[46]	L	W	S	N	A	F	R	L	G	A	I	L	H	845
(1(10)*E*,5*E*)-germacradiene-11-ol (*D. purpureum*)[50]	C	F	I	M	T	F	R	V	G	V	V	S	L	843
α-selinene (*Herpetosiphon aurantiacus*)[2]	L	C	I	N	L	F	R	S	A	V	L	S	L	839
germacrene D-4-ol (II) (*Termitomyces* sp.)[51]	L	T	C	L	L	F	R	S	G	C	V	T	W	839
8-*epi*-α-selinene (*Nostoc punctiforme*)[27]	L	A	T	L	V	F	R	V	G	V	I	A	W	835
γ-cadinene (I) (*Termitomyces* sp.)[51]	F	S	C	F	A	F	R	I	G	A	M	T	F	833
corvol ether (*Kitasatospora setae*)[35]	L	C	C	F	F	F	R	S	G	T	V	C	M	832
germacrene A (I) (*Micromonospora marina*)[29]	L	A	L	I	L	F	R	G	G	V	V	C	W	827
avermitilol (*Streptomyces avermitilis*)[20]	L	G	C	M	F	F	R	A	G	T	T	L	W	827
fusagramineol (*Fusarium graminearum*)[52]	C	L	L	V	L	F	R	V	G	K	S	I	A	825
intermedeol (I) (*Streptomyces clavuligerus*)[6]	W	T	V	H	L	W	R	G	G	L	I	S	A	822
7-*epi*-α-eudesmol (*S. viridochromogenes*)[10]	A	T	L	F	A	F	R	V	G	I	I	G	W	821
epi-α-bisabolol (*Streptomyces citricolor*)[19]	L	A	L	L	F	T	R	S	C	L	A	T	M	814
T-muurolol (II) (*Roseiflexus castenholzii*)[10]	L	A	A	I	L	F	R	G	A	V	I	C	W	810
α-amorphene (*Streptomyces viridochromogenes*)[10]	L	N	A	I	Q	F	R	A	G	V	A	V	W	808
γ-cadinene (II) (*Chitinophaga pinensis*)[10]	L	A	I	N	L	F	R	G	A	L	V	C	I	804
β-barbatene (*Dictyostelium discoideum*)[53]	S	L	A	L	C	F	R	G	T	Y	M	A	I	800
koraiol (*Fusarium fujikuroi*)[44]	M	C	M	N	V	F	R	V	G	A	V	T	I	798
eremophilene (II) (*Sorangium cellulosum*)[31]	L	L	A	T	L	F	R	G	A	V	L	C	M	797
(1(10)*E*,4*E*,6*S*,7*R*)-germacradien-6-ol (*S. pratensis*)[16]	I	G	T	L	L	F	R	S	G	V	I	G	W	791
dauc-8-en-11-ol (*Streptomyces venezuelae*)[6]	L	T	N	N	A	L	R	F	A	A	M	G	F	781

germacrene A (II) (*Nostoc* sp.)[27]	L	A	A	I	L	F	R	A	A	A	L	A	W	774
germacrene D (*Fusarium fujikuroi*)[54]	F	A	M	G	V	F	R	S	G	V	V	L	V	764
germacrene D-4-ol (III) (*Collimonas pratensis*)[8]	L	M	A	L	Y	F	R	G	G	M	A	C	A	749
hedycaryol (*Kitasatospora setae*)[55]	S	V	G	I	T	F	R	V	G	M	I	A	N	735
intermedeol (II) (*Termitomyces* sp.)[51]	L	A	C	L	A	F	R	I	G	A	I	S	V	726
mean ± SD														855±58
**DTC**														
chryseodiene (*Chryseobacterium polytrichastri*)[1]	M	W	L	M	A	M	R	L	M	V	I	I	I	1000
(*S*)-nephthenol (*Dictyostelium purpureum*)[53]	F	Y	I	V	F	S	R	F	G	F	C	V	V	939
venezuelaene (*Streptomyces venezuelae*)[18]	Y	T	I	Y	V	T	R	F	G	C	L	I	L	932
spata-13,17-diene (*Streptomyces xinghaiensis*)[33]	F	T	V	F	L	F	R	G	A	I	V	V	W	928
odyverdiene (*Streptomyces* sp.)[2]	L	A	S	M	M	F	R	S	G	M	I	I	W	882
catenul-14-en-6-ol (*Catenulispora acidiphila*)[9]	M	V	Y	M	F	F	R	T	A	A	I	A	V	881
cembrene C (*Rubrobacter xylanophilus*)[2]	L	F	I	C	M	F	R	G	G	M	V	C	R	876
micromonocyclol (*Micromonospora marina*)[25]	Y	L	L	F	F	F	R	S	A	A	L	S	A	874
cembrene A (I) (*Allokutzneria albata*)[5]	L	E	V	Y	M	F	R	S	A	T	N	S	M	874
obscuronatin (*Herpetosiphon aurantiacus*)[2]	L	A	I	S	L	F	R	S	G	T	V	F	W	856
spinodiene (*Saccharopolyspora spinosa*)[56]	F	V	V	V	A	W	R	S	G	P	I	S	W	838
dolastadiene (*C. gloeosporioides*)*[57]	V	W	I	S	L	F	R	A	G	A	M	I	C	820
(*R*)-nephthenol (*Streptomyces* sp.)[58]	A	P	V	L	Y	F	R	V	A	S	C	L	V	814
β-pinacene (*Dictyostelium discoideum*)[59]	I	F	G	L	C	V	R	I	G	I	I	S	L	813
β-araneosene (*Dictyostelium purpureum*)[53]	S	M	T	I	S	F	R	V	G	I	I	S	I	804
variediene (*Aspergillus brasiliensis*)*[60]	F	V	I	F	A	F	R	T	G	A	L	G	Q	801
bonnadiene (*Allokutzneria albata*)[12]	L	A	V	V	W	T	R	G	G	V	I	A	W	785
hydropyrene (*Streptomyces clavuligerus*)[2]	A	V	M	M	Y	A	R	G	H	V	T	S	M	774
cembrene A (II) (*Streptomyces mobaraensis*)[17]	V	I	A	L	H	T	I	A	I	G	F	V	T	749
tsukubadiene (*Streptomyces tsukubaensis*)[2]	L	W	V	N	A	F	R	A	A	A	A	T	V	748
spiroalbatene (*Allokutzneria albata*)[5]	Y	A	G	F	G	S	R	I	A	M	T	A	Y	742
phomopsene (I) (*Phomopsis amygdali*)*[61]	F	T	V	M	A	F	R	C	A	S	A	A	M	741
myrothec-15(17)-en-7-ol (*M. graminearum*)[62]	F	T	I	N	A	F	R	A	G	E	L	G	S	733
fusicoccadiene (*Phomopsis amygdali*)*[63]	F	A	I	N	A	F	R	V	G	E	V	G	S	724

cycloaraneosene (*Sordaria araneosa*)[64]	L	A	G	C	A	F	R	I	G	A	T	W	V	715
cyclopiane-type diterpene (*P. chrysogenum*)*[65]	F	C	V	V	A	F	R	A	A	A	L	C	S	713
18-hydroxydolabella-3,7-diene (*C. pinensis*)[28]	M	F	A	L	L	F	R	G	G	A	G	C	V	705
cattleyene (*Streptomyces cattleya*)[11]	F	I	A	C	M	F	R	A	A	G	A	A	L	705
cyclooctat-7(8),10(14)-diene (*S. lactacystinaeus*)[2]	Y	C	I	M	G	G	R	V	A	G	V	G	W	699
clavulatriene (*Streptomyces clavuligerus*)[2]	W	C	L	H	A	F	R	A	G	G	G	V	V	699
phomopsene (II) (*Allokutzneria albata*)[12]	F	L	A	G	M	F	R	A	G	A	A	A	A	617
spiroviolene (*Streptomyces violens*)[13]	F	F	T	V	G	F	R	A	G	G	A	A	G	611
wanjudiene (*Chryseobacterium wanjuense*)[1]	I	G	L	T	G	A	R	S	G	V	I	G	M	610
phomopsene (III) (*Nocardia testacea*)[11]	F	M	A	A	G	F	R	A	A	G	A	A	V	600
polytrichastrene (*Chryseobacterium polytrichastri*)[66]	I	G	L	A	G	A	R	S	G	A	I	G	M	550
mean ± SD														776±107
**StTS**														
stellatatriene (*Aspergillus stellatus*)*[67]	L	V	V	N	A	F	R	I	G	L	I	M	T	847
asperterpenol A (*Aspergillus calidoustus*)*[68]	L	G	F	T	Y	F	R	G	D	S	T	L	V	824
Bm1 (*Bipolaris maydis*)*[69]	W	V	L	T	G	L	R	G	G	M	L	V	I	798
sesterfisherol (*Aspergillus fischeri*)*[70]	L	A	V	T	G	I	R	F	G	I	I	I	T	786
mangicdiene (*Fusarium graminearum*)*[71]	F	T	V	N	A	F	R	V	G	Y	V	T	G	784
Bm3 (*Bipolaris maydis*)*[69]	W	I	L	S	G	L	R	G	G	M	L	L	A	763
quiannulatene (*Aspergillus stellatus*)*[72]	V	T	M	T	T	F	R	S	G	I	T	T	T	762
ophiobolin F (*Aspergillus clavatus*)*[73]	F	A	V	L	G	F	R	V	G	Y	A	S	T	749
preasperterpenoid A (*Talaromyces verruculosus*)*[74]	G	A	V	N	A	F	R	L	G	Q	L	A	S	673
aspergildiene (*Aspergillus ustus*)*[75]	Y	A	V	C	G	F	R	G	G	P	G	V	L	649
sesterbrasiliatriene (*Penicillium brasilianum*)*[74]	F	A	C	C	G	F	R	V	G	A	A	A	M	637
astellifadiene (*Aspergillus stellatus*)*[76]	G	A	V	S	A	F	R	V	G	M	M	A	S	632
sestermobaraene (*Streptomyces mobaraensis*)[17]	L	L	L	N	A	F	G	G	G	P	A	V	V	623
mean ± SD														733±79

[a] Asterisks indicate bifunctional enzymes with two domains (terpene synthase + oligoprenyl diphosphate synthase domain). The enzyme marked in red (CpPS) was previously reported as linalool synthase.[66] [b] Active site residues for SdS[14] as shown in Figure 1 of main text. For all other enzymes the residues in the corresponding positions as listed here were identified by amino acid sequence alignment with SdS.

Enzyme incubation of GGPP with wildtype CpPS and compound isolation

Geranylgeraniol was isolated from *Bixa orellana* and converted chemically into GGPP through known procedures.[77,78] Wildtype CpPS was expressed and purified as reported previously (from 4 L of culture volume, note that CpLS is identical to CpPS)[66] to yield 50 mL of a protein solution in elution buffer (50 mM Tris, 300 mM NaCl, 300 mM imidazole, 5% glycerol, pH 7.6) with a protein concentration of ca. 7 mg/mL.

GGPP trisammonium salt (50 mg, 0.13 mmol) was dissolved in NH_4HCO_3 solution (50 mL; 25 mM in H_2O). Tris buffer (150 mL; 50 mM Tris, 1 mM $MgCl_2$, pH 7.6), incubation buffer (250 mL; 50 mM Tris, 5 mM $MgCl_2$, 5% glycerol, pH 7.6) and purified protein solution (50 mL) were added. The mixture was incubated at 28 °C overnight, and then extracted with hexane (2 x 300 mL). This enzymatic reaction was performed four times (total amount of converted GGPP trisammonium salt: 200 mg, 398.8 μmol), and all extracts were combined and purified via silica gel chromatography. The first fractions with elution of pentane afforded impure **1**, chromatographed again on silica gel with pentane to obtain pure **1** (1.2 mg, 4.4 μmol, 1.1%), and pure **3** (2.1 mg, 7.7 μmol, 1.9%), the next fraction through elution with pentane/Et_2O (5/1) afforded **2** (0.3 mg, 1.0 μmol, 0.3%), and the last fraction obtained with pentane/Et_2O (1/1) yielded **4** (1.0 mg, 3.4 μmol, 0.9%).

GC/MS

GC/MS analyses were performed on a 7890B/5977A series gas chromatography/mass selective detector (Agilent, Santa Clara, CA, USA). The GC was equipped with an HP5-MS fused silica capillary column (30 m, 0.25 mm i. d., 0.50 μm film; Agilent). GC settings were 1) inlet pressure: 77.1 kPa, He at 23.3 mL min^{-1}, 2) injection volume: 1 – 2 μL, 3) temperature program: 5 min at 50 °C then increasing 5 °C min^{-1} to 320 °C, 4) 60 s valve time, and 5) carrier gas: He at 1.2 mL min^{-1}. MS settings were 1) source: 230 °C, 2) transfer line: 250 °C, 3) quadrupole: 150 °C and 4) electron energy: 70 eV. Retention indices (*I*) were calculated from retention times in comparison to retention times of *n*-alkanes (C_7-C_{40}).

HRMS

High resolution mass spectroscopy analyses were conducted on a 7890B/7200 series gas chromatography/accurate mass Q-ToF detector system (Agilent). The GC was equipped with a HP5-MS fused silica capillary column (30 m, 0.25 mm i. d., 0.50 mm film). GC settings were 1) injection volume: 1 μL, 2) temperature program: 5 min at 50 °C, increasing 10 °C min^{-1} to 320 °C, 3) split ratio: 5:1, 60 s valve time and 4) carrier gas flow: He at 1 ml min^{-1}. MS settings were 1) inlet pressure: 83.2 kPa, He flow at 24.6 ml min^{-1}, 2) transfer line temperature: 250 °C, 3) ionization energy: 70 eV.

NMR spectroscopy

NMR spectra were recorded at 298 K on a Bruker (Billerica, MA, USA) Avance III HD Cryo (700 MHz) NMR spectrometer. Spectra were measured in C_6D_6 and referenced against solvent signals (^{1}H-NMR, residual proton signal: δ = 7.16 ppm; ^{13}C-NMR: δ = 128.06 ppm).[79]

IR spectroscopy

IR spectra were recorded on a Bruker α infrared spectrometer with a diamond ATR probehead. Peak intensities are given as s (strong), m (medium), w (weak) and br (broad).

Optical rotations

Optical rotations were recorded on a Modular Compact Polarimeter MCP 100 (Anton Paar, Graz, Austria). The temperature setting was 25 °C; the wavelength of the light used was 589 nanometers (the sodium D line); the path-length was 10 cm, the compound concentrations *c* are given in g 100 mL^{-1}.

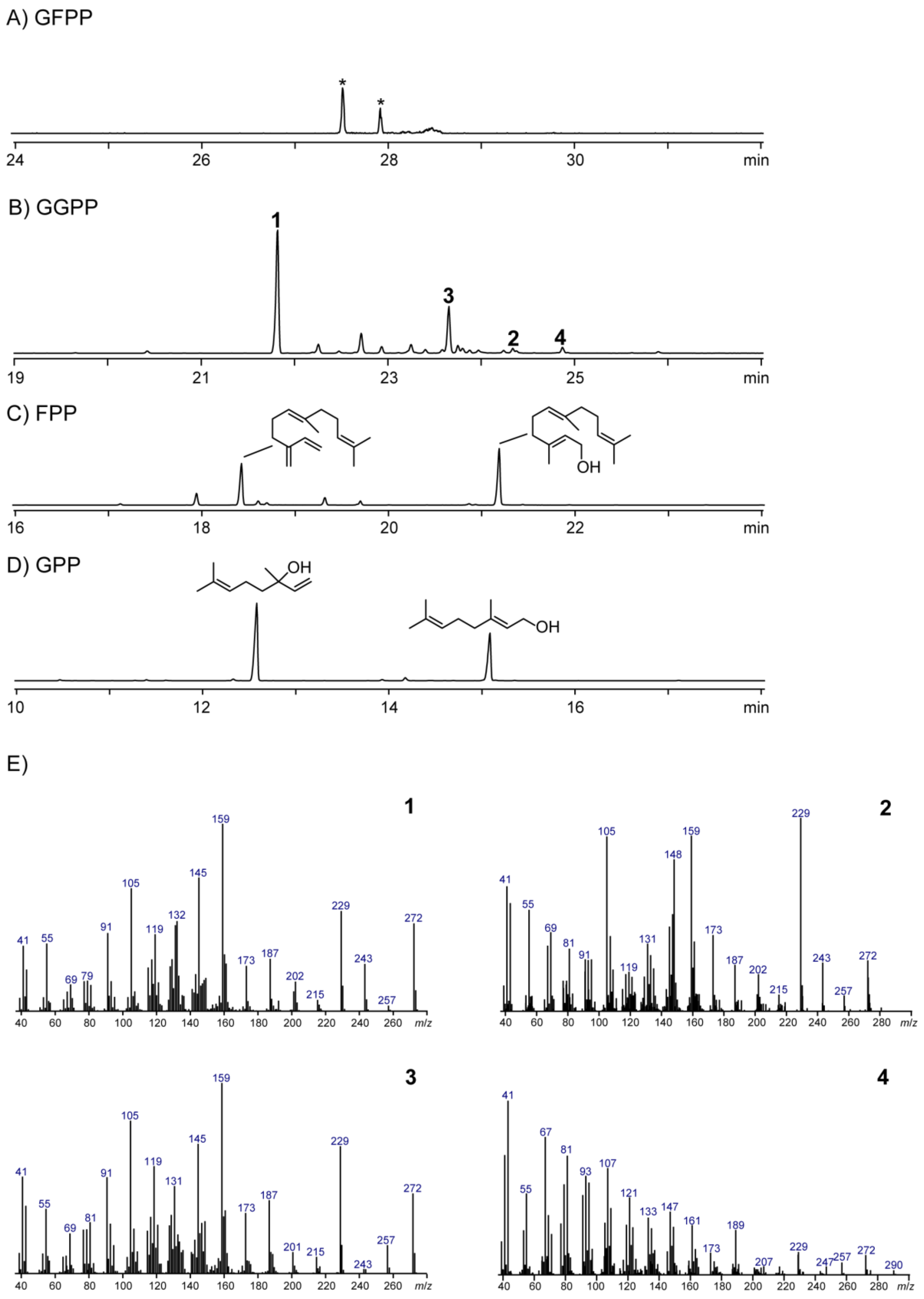

Figure S2. Conversion of GFPP, GGPP, FPP and GPP by CpPS. Partial total ion chromatogram showing the extracts from the incubation of A) GFPP, B) GGPP, C) FPP and D) GPP with CpPS. Asterisks indicate degradation products from GFPP also observed without enzyme. Labels at peaks refer to compound numbers as in Scheme 1 of main text. E) EI mass spectra of compounds **1** – **4**.

Polytrichastrene A (1). Yield: 1.2 mg (4.4 μmol, 1.1%), from 200 mg (398.8 μmol) GGPP trisammonium salt. TLC (pentane): R_f = 0.86. GC (HP5-MS): *I* = 1790. IR (diamond ATR): ṽ = 2952 (m), 2925 (s), 2870 (m), 2853 (m), 2278 (w), 1676 (w), 1566 (w), 1462 (m), 1452 (m), 1373 (w), 1345 (w), 1325 (w), 1316 (w), 1260 (w), 1159 (w), 1094 (w), 1016 (w), 995 (w), 955 (w), 875 (w), 842 (w), 815 (m), 802 (m), 745 (w), 587 (w), 543 (s), 466 (w), 439 (w), 419 (w) cm^{-1}. HR-MS (APCI): calc. for $[C_{20}H_{32}]^+$ m/z = 272.2499; found: m/z = 272.2495. Optical rotary power: $[\alpha]_D^{25}$ = +15.83 (*c* 0.12, CH_2Cl_2).

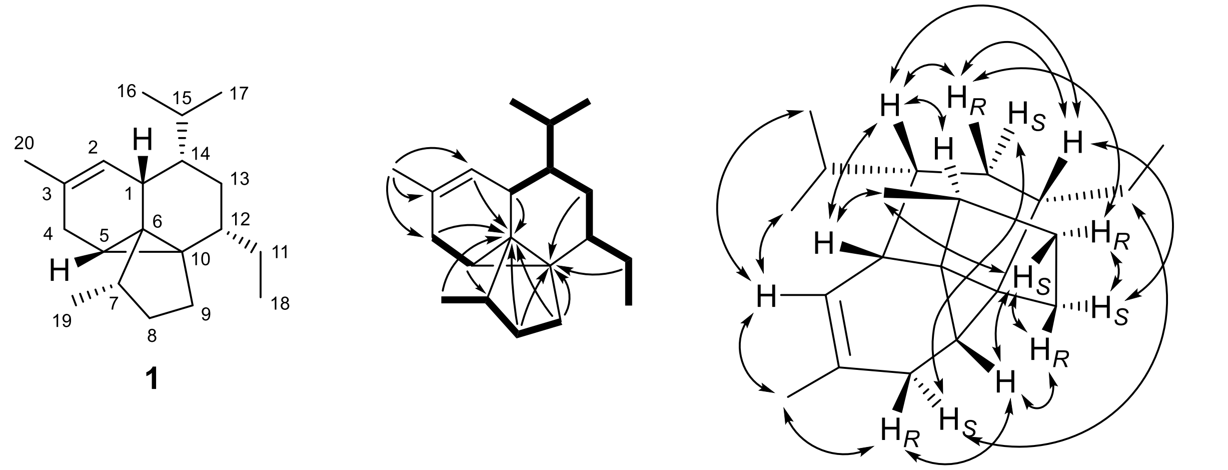

Figure S3. Structure elucidation of **1**. Bold: $^1H,^1H$-COSY, single headed arrows: key HMBC, and double headed arrows: NOESY correlations. Carbon numbering follows GGPP numbering to indicate the origin of each carbon. Diastereotopic hydrogens are labelled H_R (*pro-R*) and H_S (*pro-S*).

Table S3. NMR data of polytrichastrene A (**1**) in C_6D_6 recorded at 298 K.

C[a]	type	^{1}H[b]	^{13}C[b]
1	CH	2.74 (dd, *J* = 5.0, 5.0)	33.89
2	CH	5.50 (m)	121.54
3	C_q	–	135.09
4	CH_2	2.14 (m, 2H)	26.63
5	CH	0.82 (dd, *J* = 8.6, 3.7)	16.33
6	C_q	–	35.81
7	CH	2.09 (m)	40.61
8	CH_2	1.61 (m, H_R)	31.26
		0.75 (dddd, *J* = 13.3, 11.2 10.4, 8.4, H_S)	
9	CH_2	2.01 (dd, *J* = 12.4, 8.4, H_R)	38.76
		1.42 (m, H_S)	
10	C_q	–	31.53
11	CH_2	1.71 (ddq, *J* = 13.1, 6.5, 7.6)	27.89
		1.39 (m)	
12	CH	1.63 (m)	45.25
13	CH_2	1.48 (dd, *J* = 13.3, 6.5, H_R)	28.14
		0.47 (ddd, *J* = 13.1, 12.2, 12.2, H_S)	
14	CH	1.09 (dddd, *J* = 11.8, 9.8, 4.3, 2.0)	48.74
15	CH	1.35 (m)	29.59
16	CH_3	1.06 (d, *J* = 6.8)	21.54
17	CH_3	0.94 (d, *J* = 6.7)	21.91
18	CH_3	1.04 (t, *J* = 7.4)	13.30
19	CH_3	0.97 (d, *J* = 6.5)	16.45
20	CH_3	1.66 (br s)	23.70

[a] Carbon numbering as shown in main text. [b] Chemical shifts δ in ppm, multiplicity: s = singlet, d = doublet, t = triplet, q = quartet, m = multiplet, br = broad, coupling constants *J* are given in Hertz.

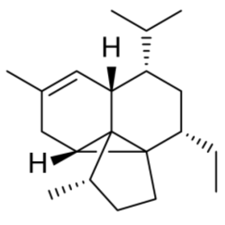

1

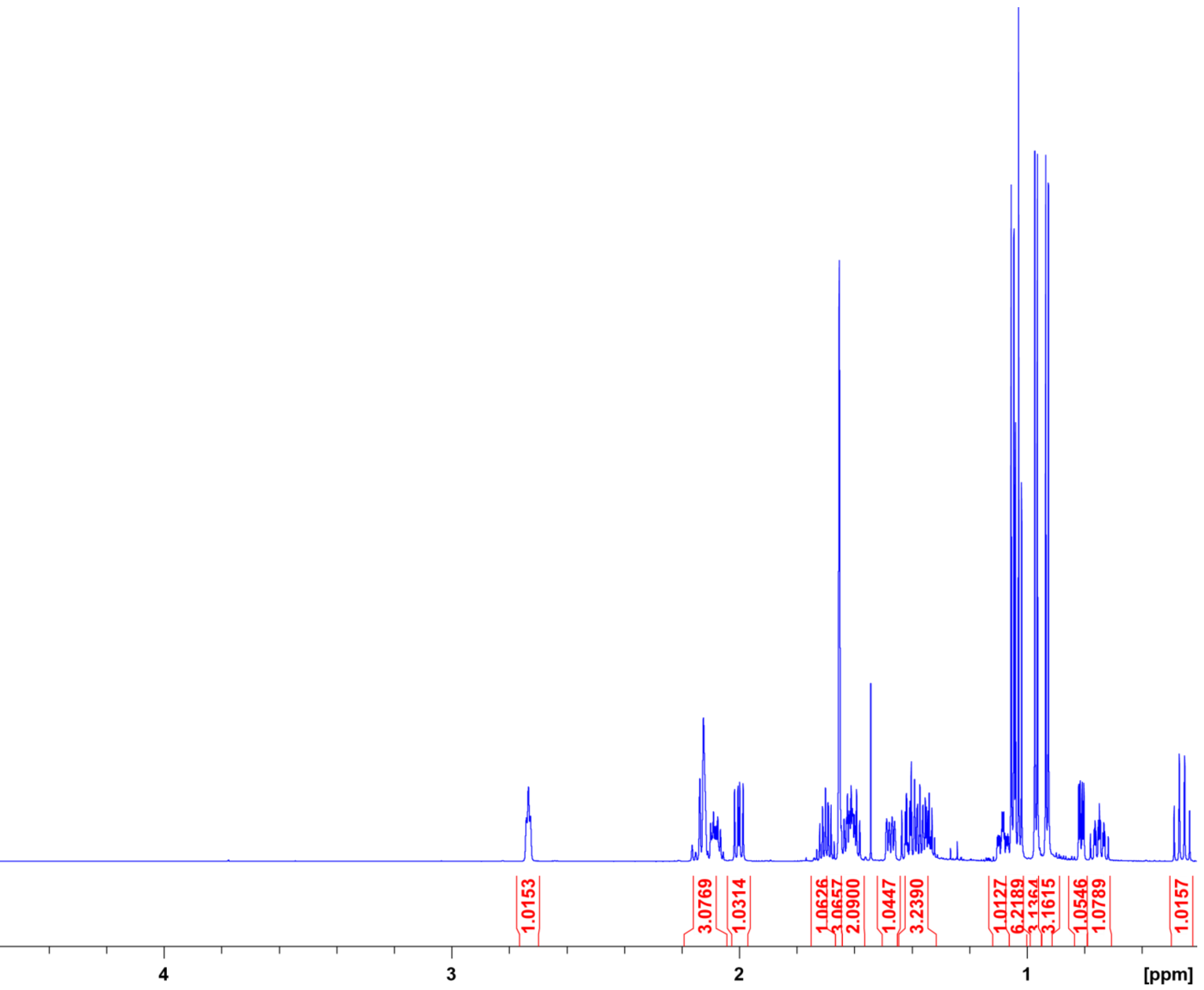

Figure S4. ^{1}H-NMR spectrum of **1** (700 MHz, C_6D_6).

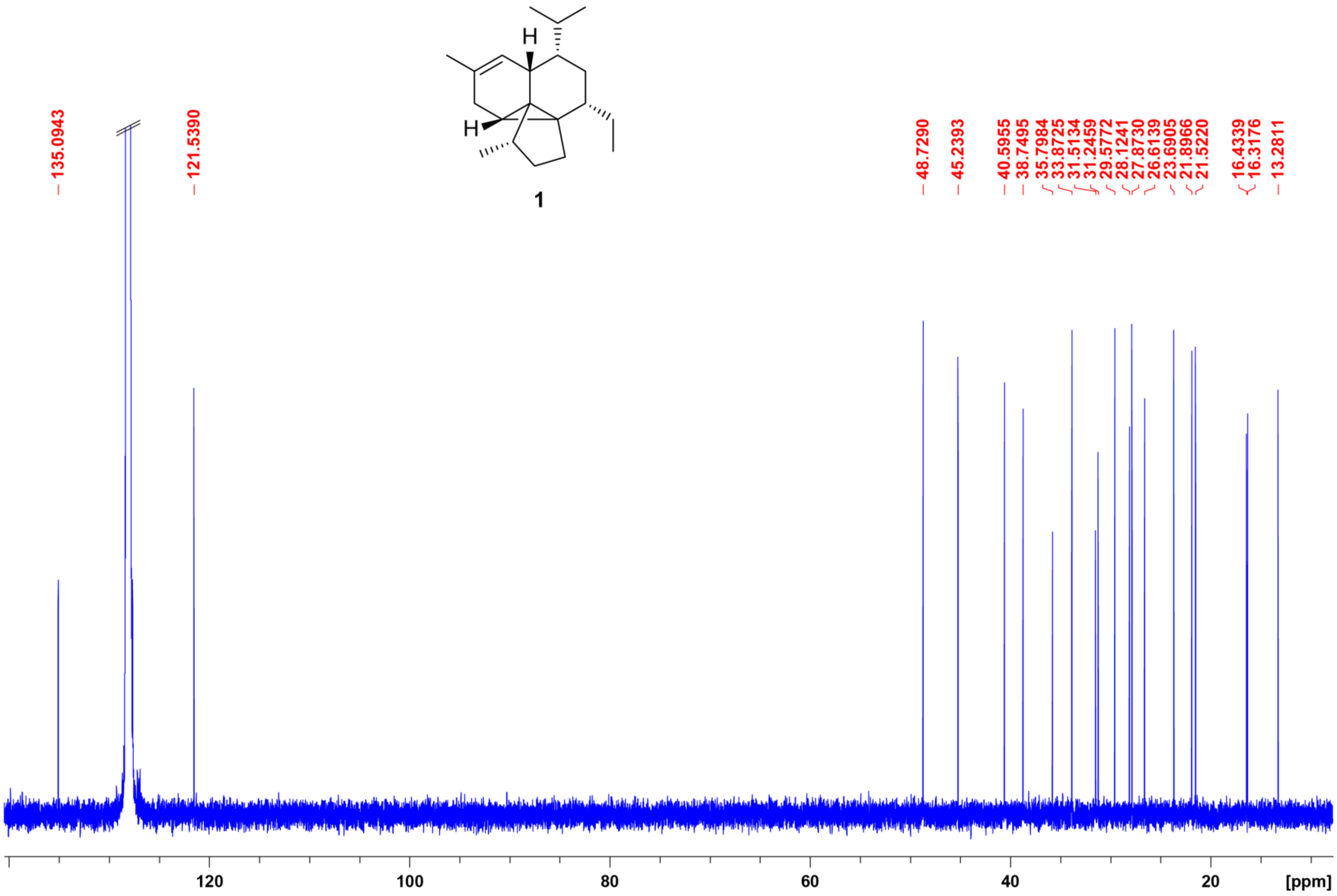

Figure S5. ^{13}C-NMR spectrum of **1** (176 MHz, C_6D_6).

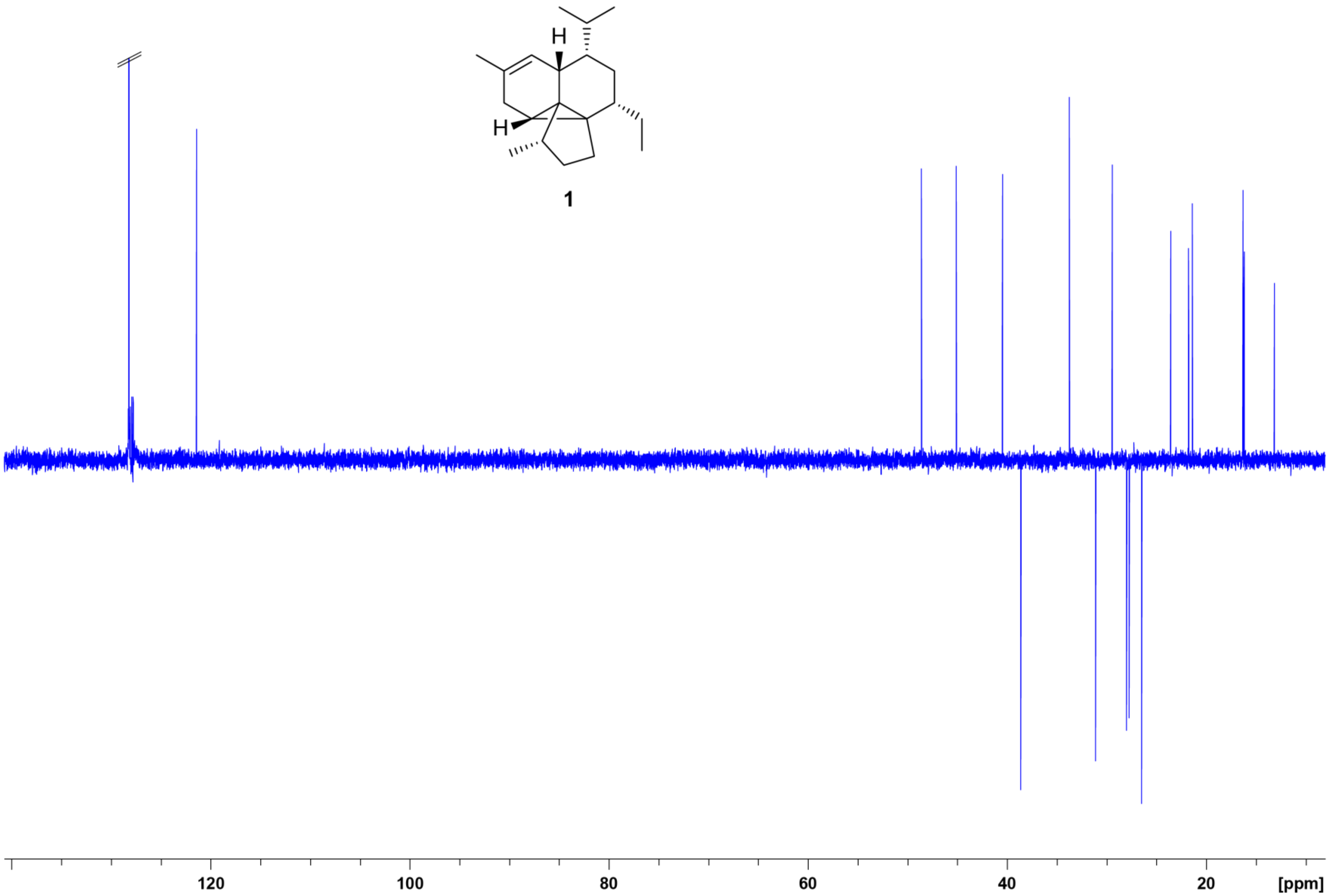

Figure S6. ^{13}C-DEPT spectrum of **1** (176 MHz, C_6D_6).

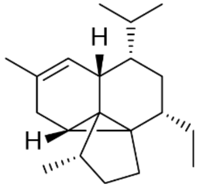

1

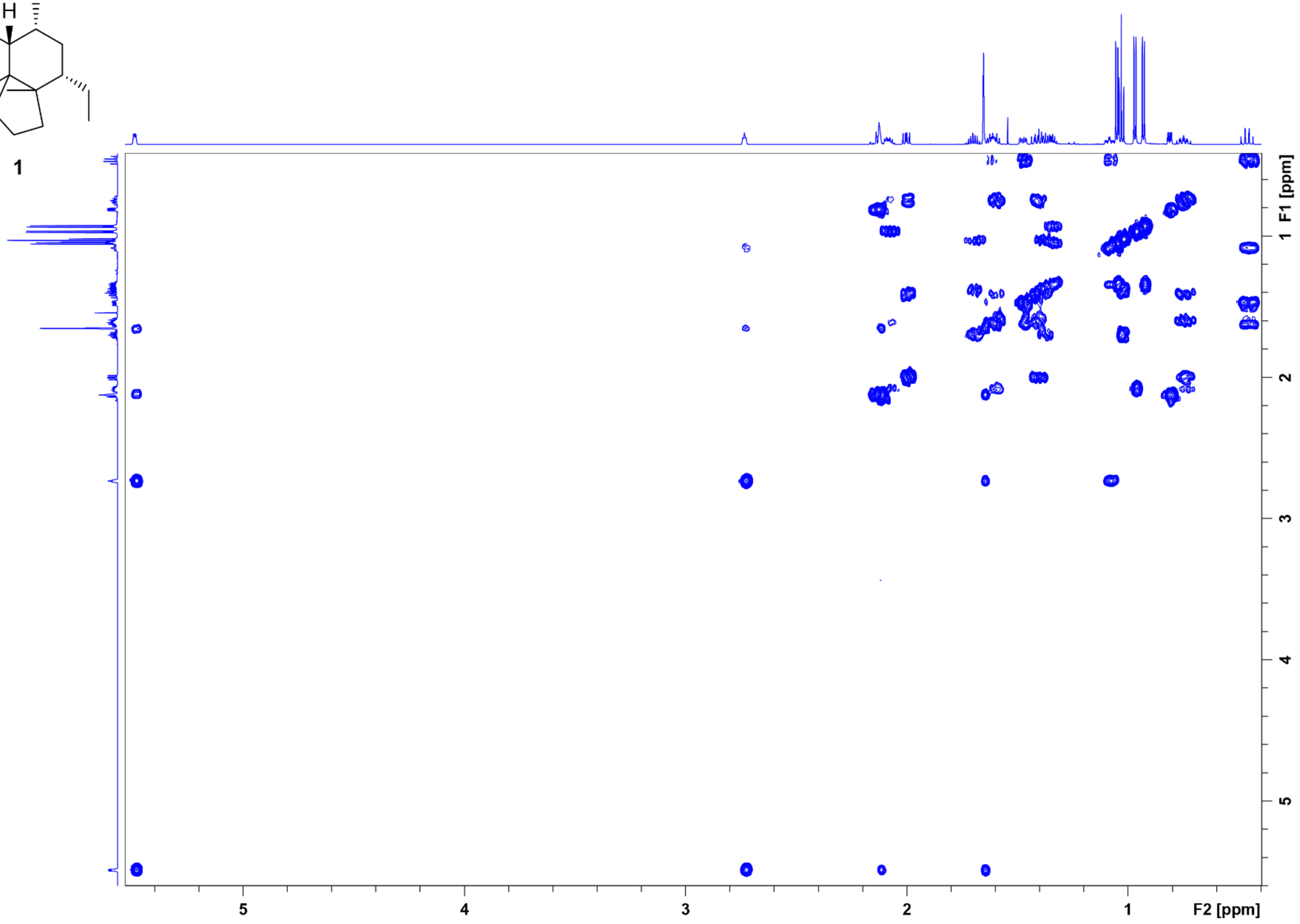

Figure S7. ^{1}H-^{1}H-COSY spectrum of **1** (700 MHz, C_6D_6).

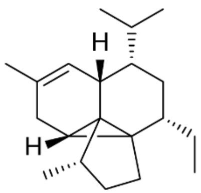

1

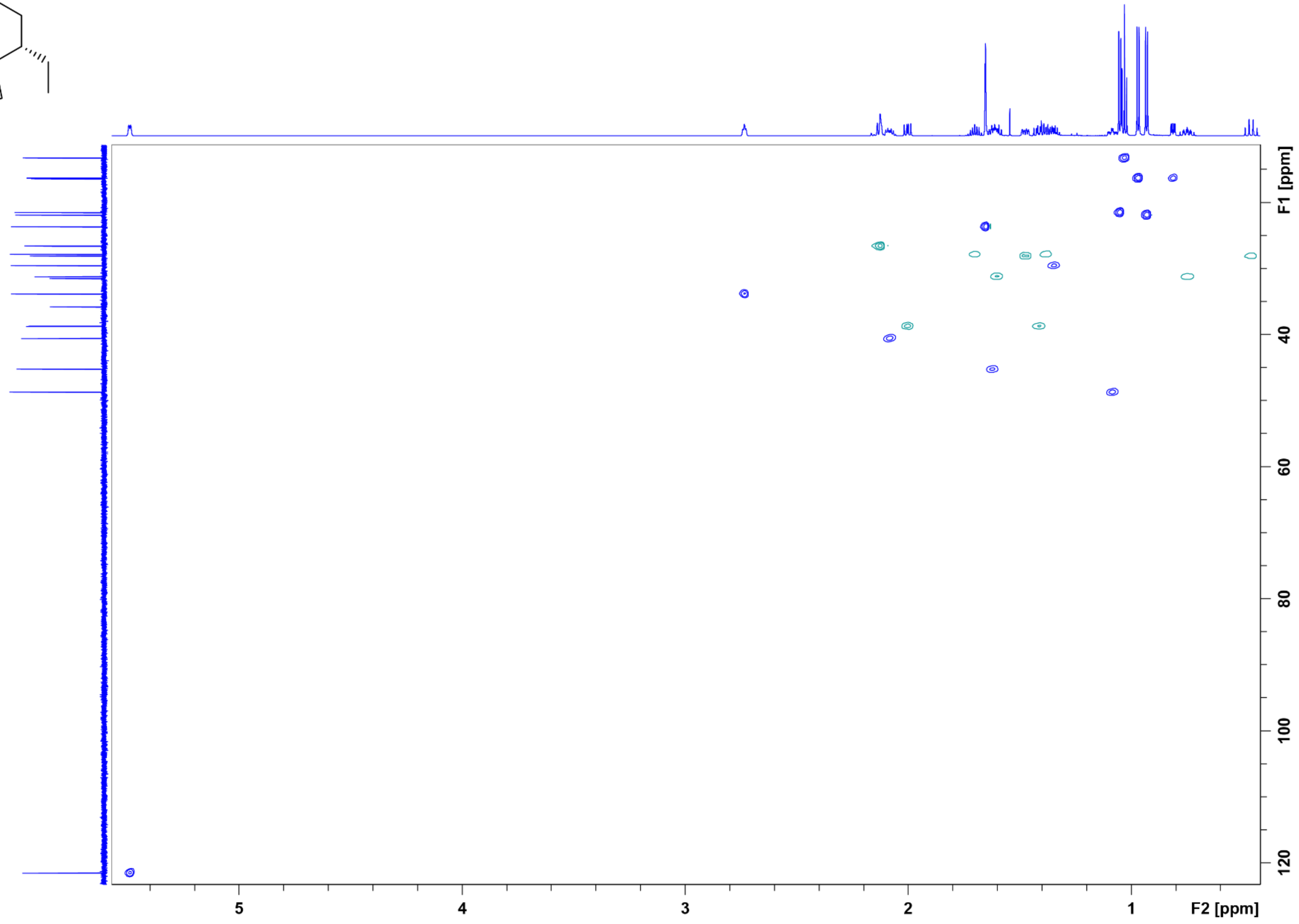

Figure S8. HSQC spectrum of **1** (C_6D_6).

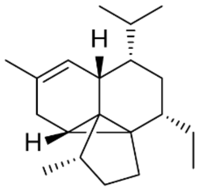

1

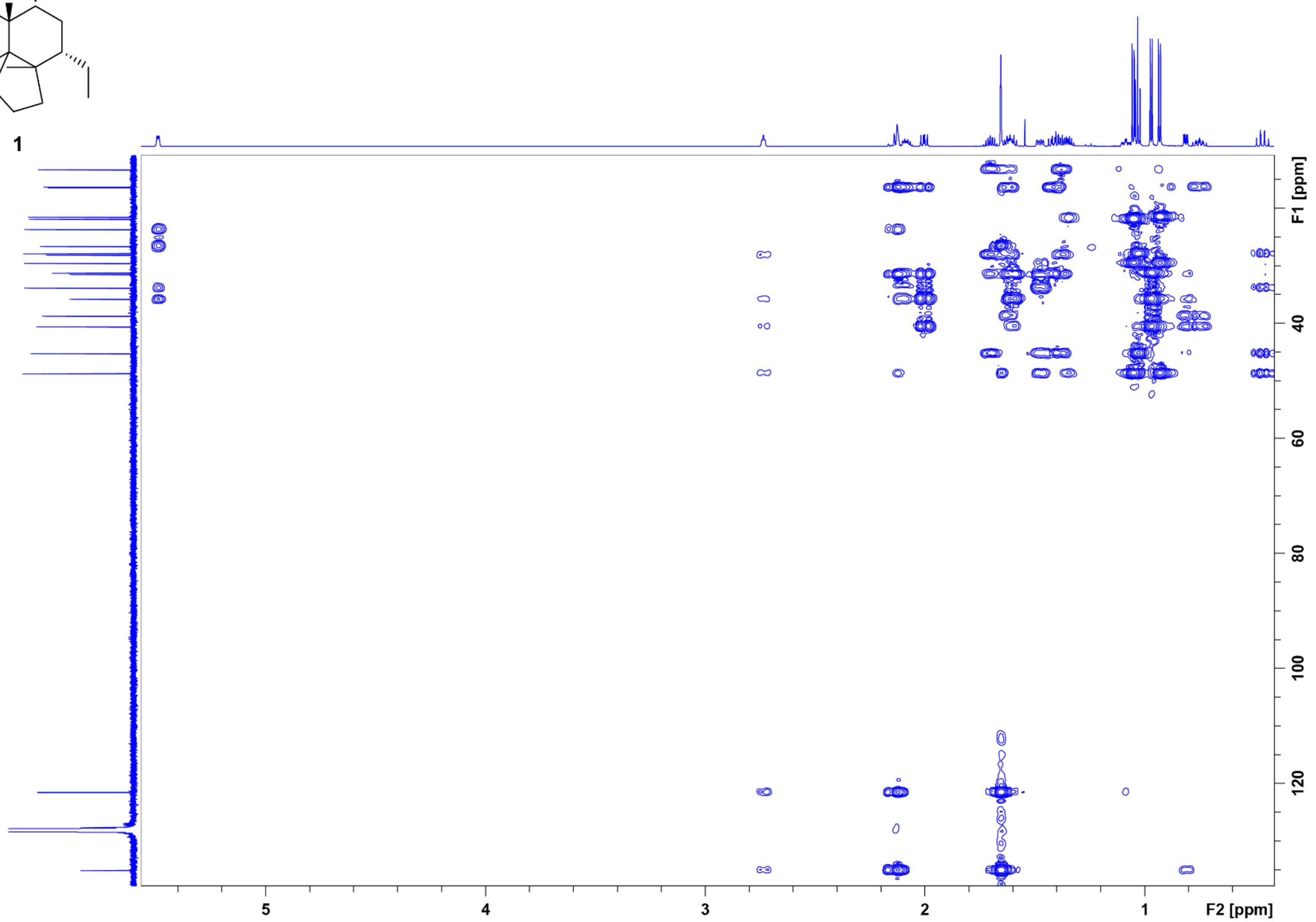

Figure S9. HMBC spectrum of **1** (C_6D_6).

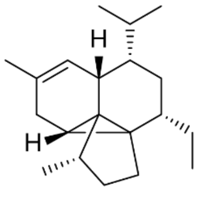

1

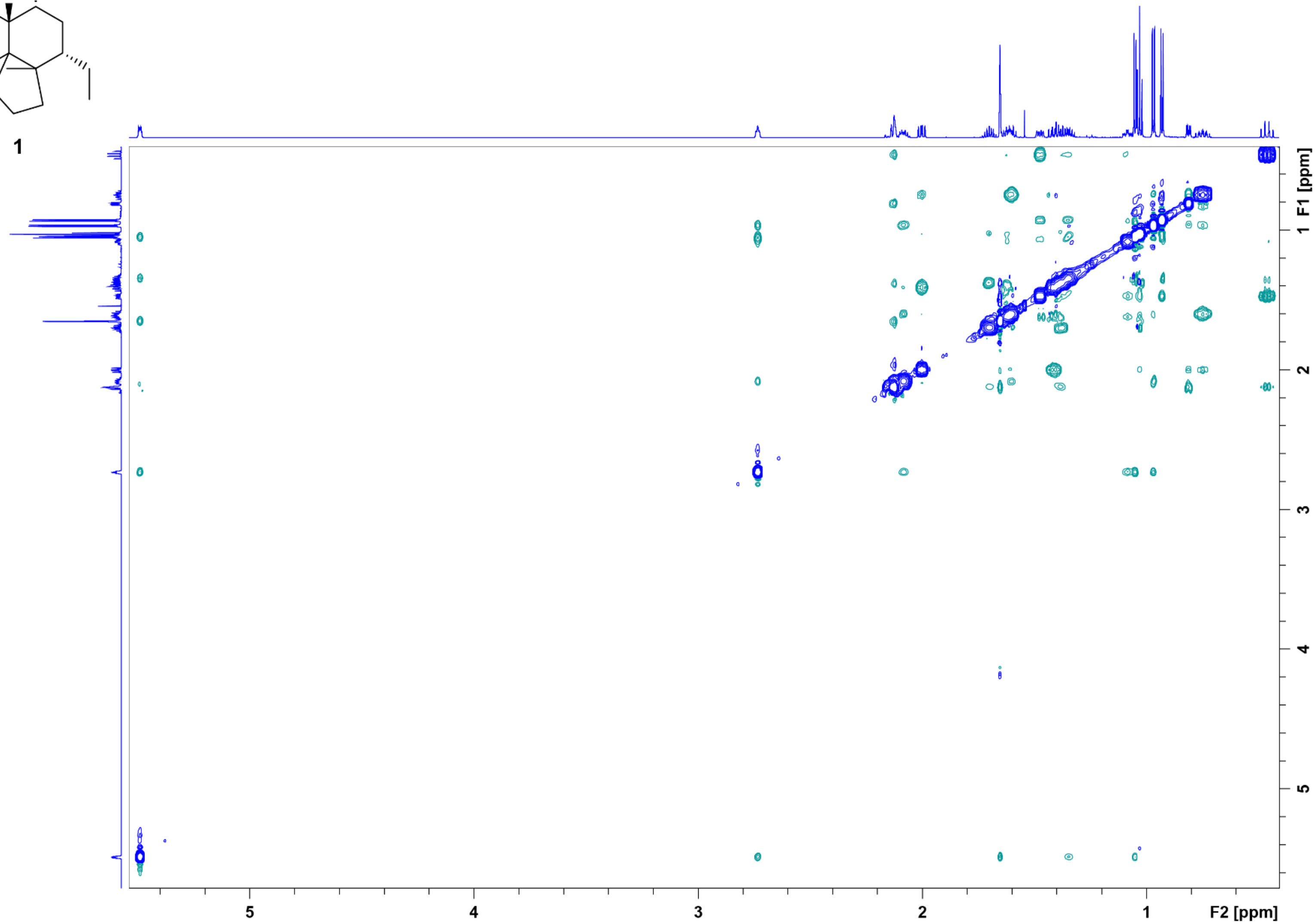

Figure S10. NOESY spectrum of **1** (C_6D_6).

Polytrichastrol A (2). Yield: 0.3 mg (1.0 μmol, 0.3%), from 200 mg (398.8 μmol) GGPP trisammonium salt. TLC (pentane/Et_2O = 5/1): R_f = 0.7. GC (HP5-MS): I = 2038. IR (diamond ATR): ṽ = 3568 (w), 3337 (w), 2953 (m), 2925 (s), 2868 (m), 2855 (m), 2277 (w), 1728 (w), 1673 (w), 1459 (w), 1376 (w), 1368 (w), 1345 (w), 1285 (w), 1261 (w), 1172 (w), 1125 (w), 1090 (w), 975 (w), 958 (w), 925 (w), 878 (w), 816 (w), 803 (w), 722 (w), 587 (w), 543 (s), 467 (w), 438 (w) cm^{-1}. HR-MS (Q-TOF, 70 eV): calc. for $[C_{20}H_{34}O + H]^+$ m/z = 291.2682; found: m/z = 291.2680. Optical rotary power: $[\alpha]_D^{25}$ = +26.67 (c 0.03, CH_2Cl_2).

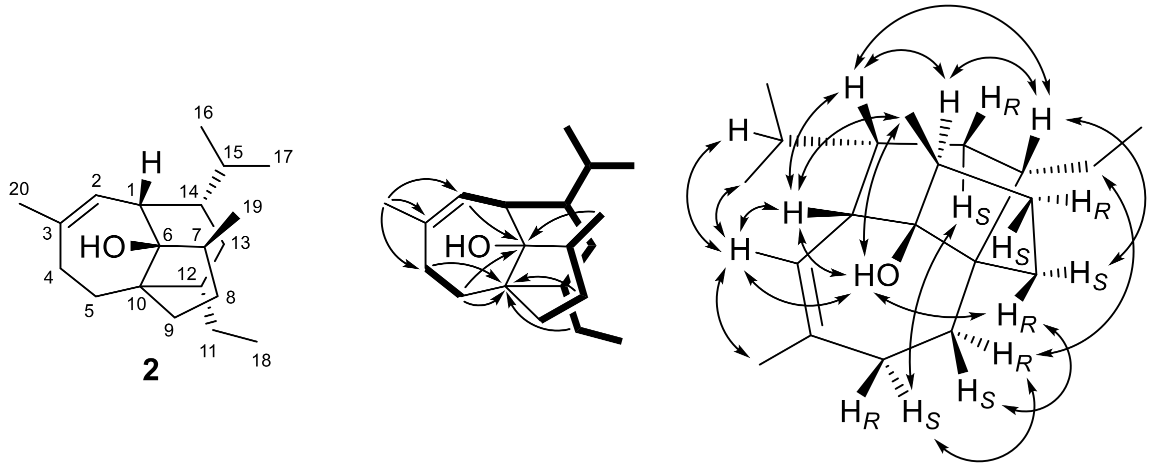

Figure S11. Structure elucidation of **2**. Bold: $^1H,^1H$-COSY, single headed arrows: key HMBC, and double headed arrows: NOESY correlations. Carbon numbering follows GGPP numbering to indicate the origin of each carbon. Diastereotopic hydrogens are labelled H_R (*pro-R*) and H_S (*pro-S*).

Table S4. NMR data of polytrichastrol A (**2**) in C_6D_6 recorded at 298 K.

C[a]	type	^{1}H[b]	^{13}C[b]
1	CH	2.65 (dd, *J* = 8.3, 4.1)	43.12
2	CH	5.24 (dsext, *J* = 8.3, 1.5)	122.41
3	C_q	–	141.21
4	CH_2	2.14 (tm, *J* = 15.7, H_S)	33.57
		1.72 (m, H_R)	
5	CH_2	1.82 (dddd, *J* = 14.4, 14.4, 5.2, 1.3, H_S)	28.83
		1.48 (ddd, *J* = 14.8, 5.4, 2.8, H_R)	
6	C_q	–	81.80
7	CH	2.35 (m)	38.18
8	CH_2	1.74 (m, 2H)	29.20
9	CH_2	1.59 (m, H_S)	36.50
		1.55 (m, H_R)	
10	C_q	–	51.85
11	CH_2	1.60 (m)	24.48
		1.02 (m)	
12	CH	1.16 (m)	44.81
13	CH_2	1.73 (m, H_R)	30.21
		0.72 (m, H_S)	
14	CH	1.27 (m)	47.39
15	CH	1.37 (m)	30.86
16	CH_3	0.92 (d, *J* = 6.6)	21.33
17	CH_3	0.87 (d, *J* = 6.6)	21.37
18	CH_3	0.94 (t, *J* = 7.3)	13.77
19	CH_3	1.12 (d, *J* = 6.7)	12.92
20	CH_3	1.58 (br s)	29.07
	OH	1.78 (d, ^{4}J = 1.9)	–

[a] Carbon numbering as shown in main text. [b] Chemical shifts δ in ppm, multiplicity: s = singlet, d = doublet, t = triplet, sext = sextet, m = multiplet, br = broad, coupling constants *J* are given in Hertz.

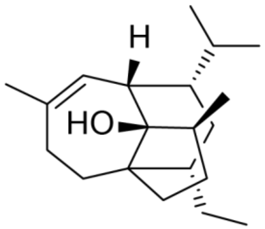

2

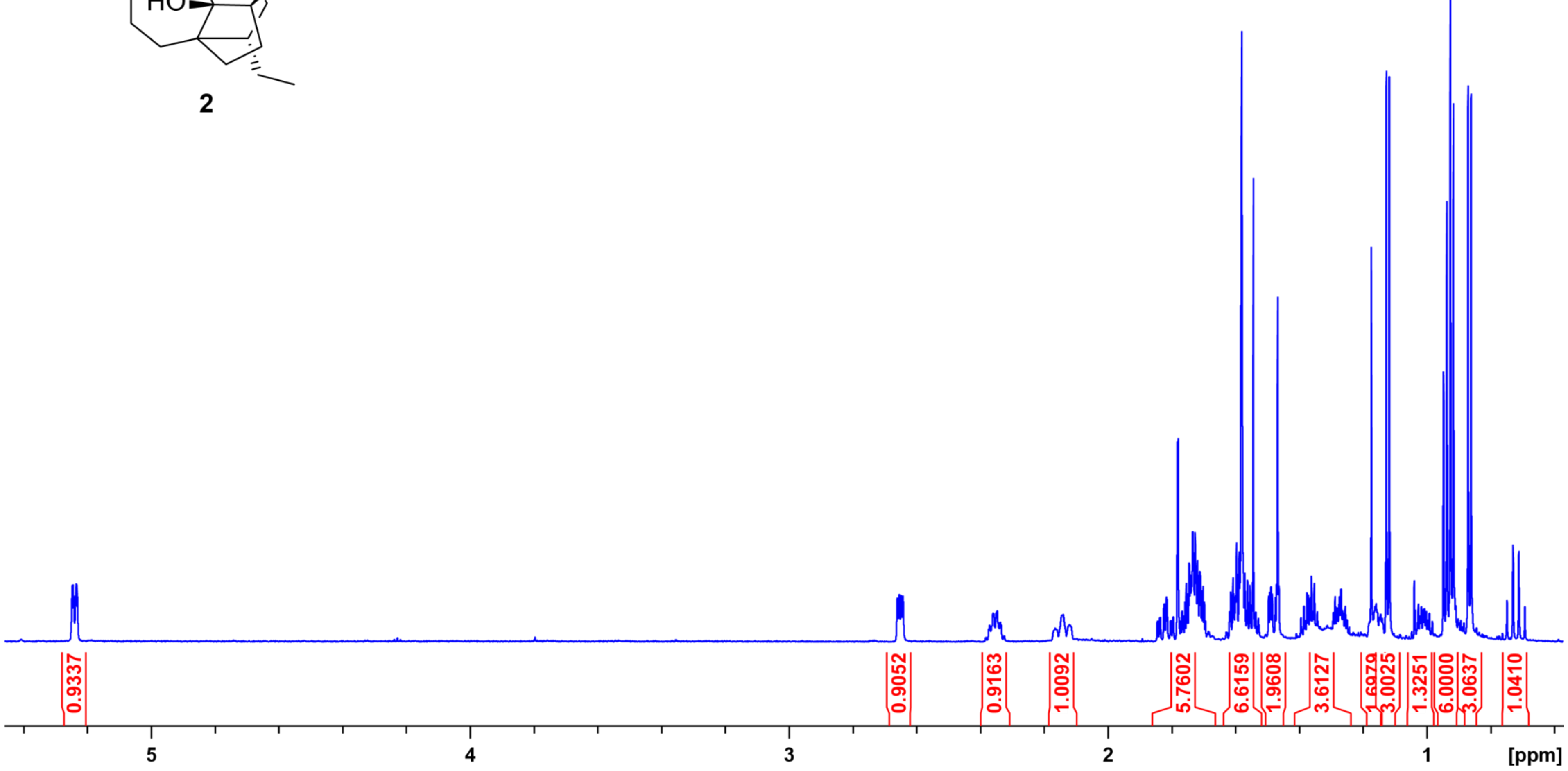

Figure S12. ^{1}H-NMR spectrum of **2** (700 MHz, C_6D_6).

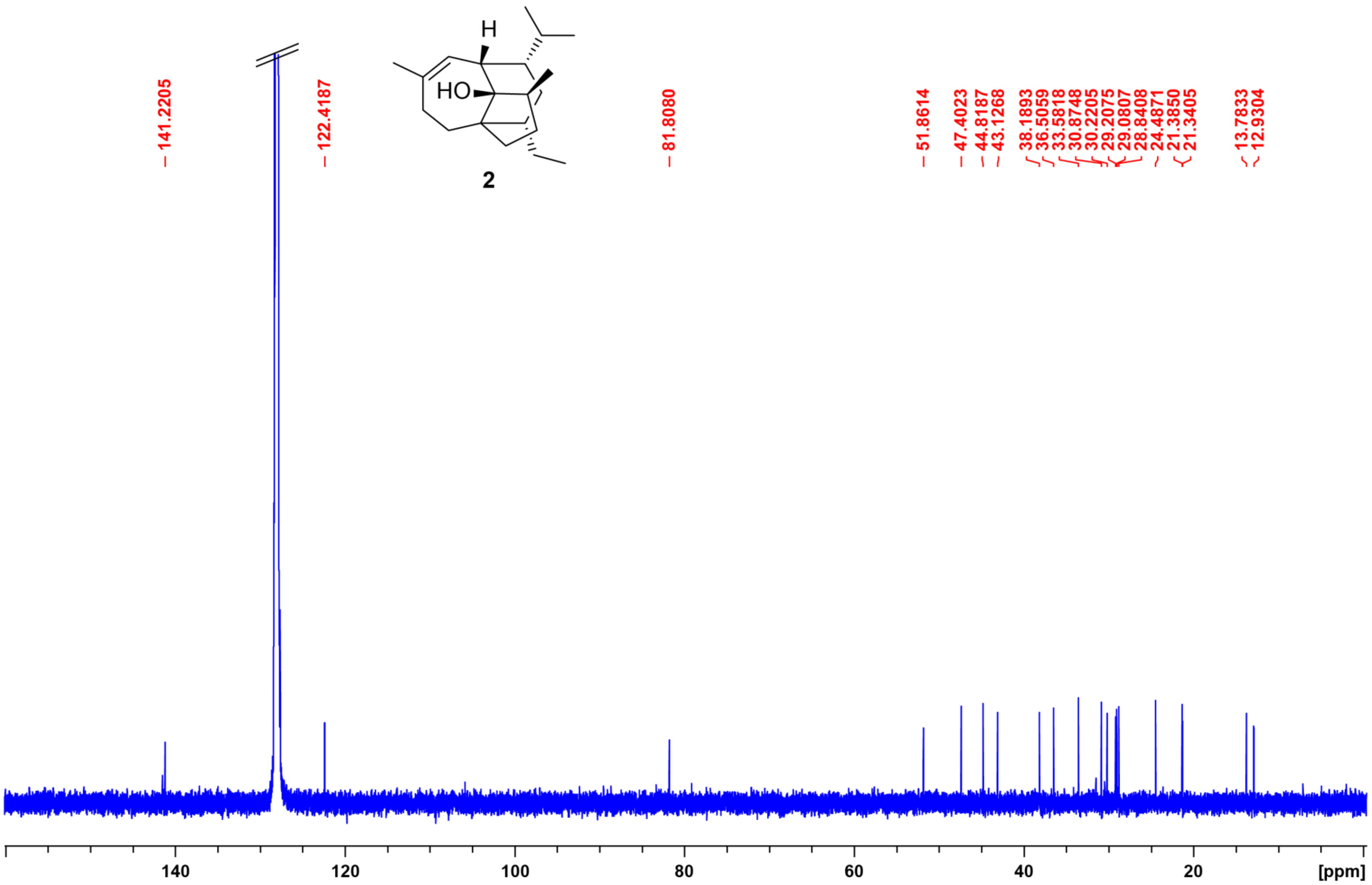

Figure S13. ^{13}C-NMR spectrum of **2** (176 MHz, C_6D_6).

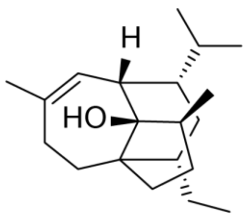

2

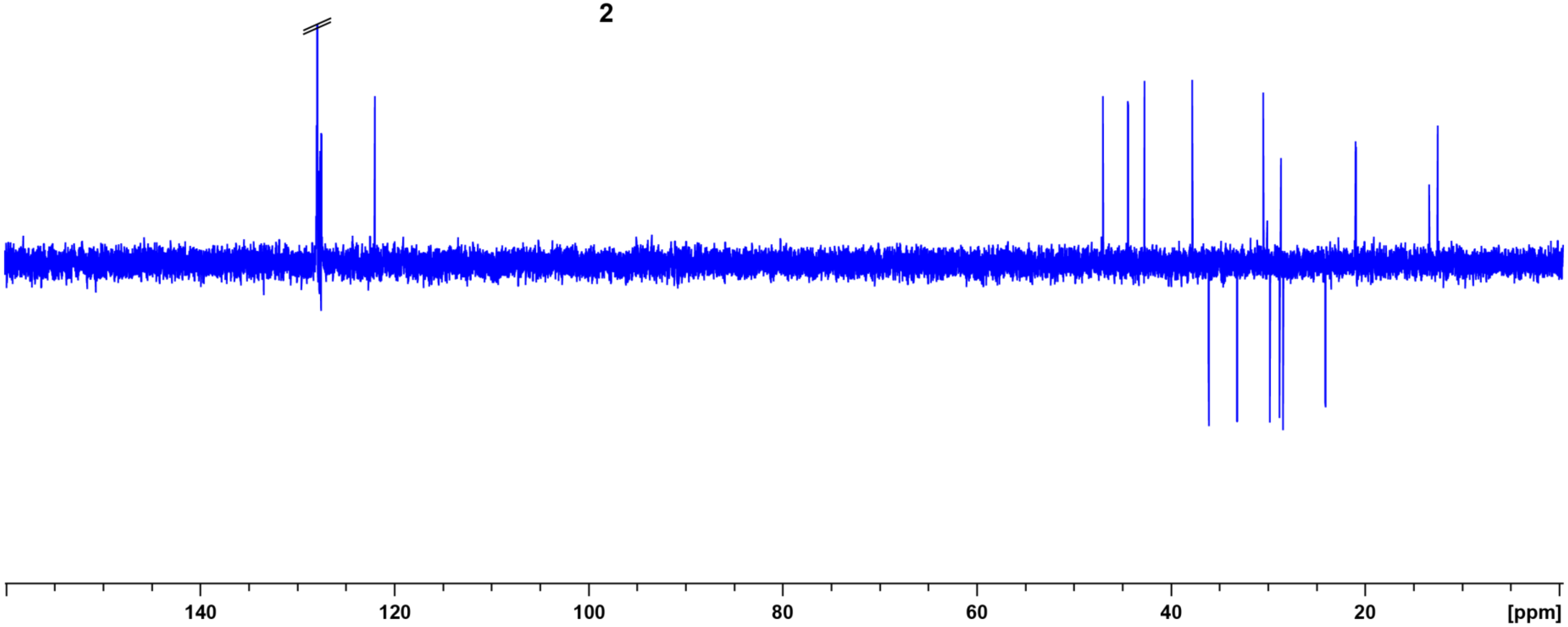

Figure S14. ^{13}C-DEPT spectrum of **2** (176 MHz, C_6D_6).

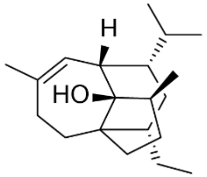

2

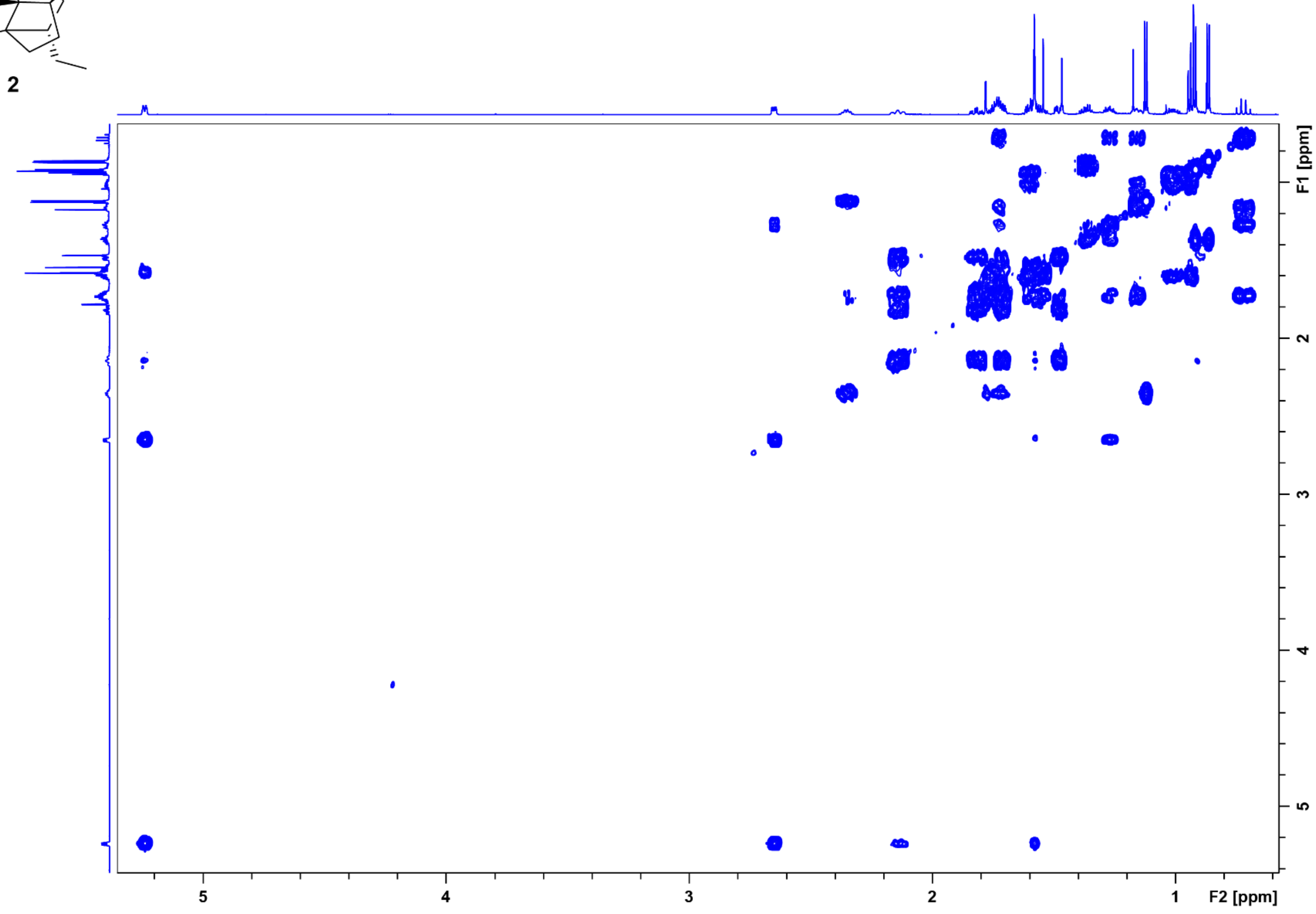

Figure S15. ^{1}H-^{1}H-COSY spectrum of **2** (700 MHz, C_6D_6).

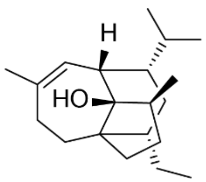

2

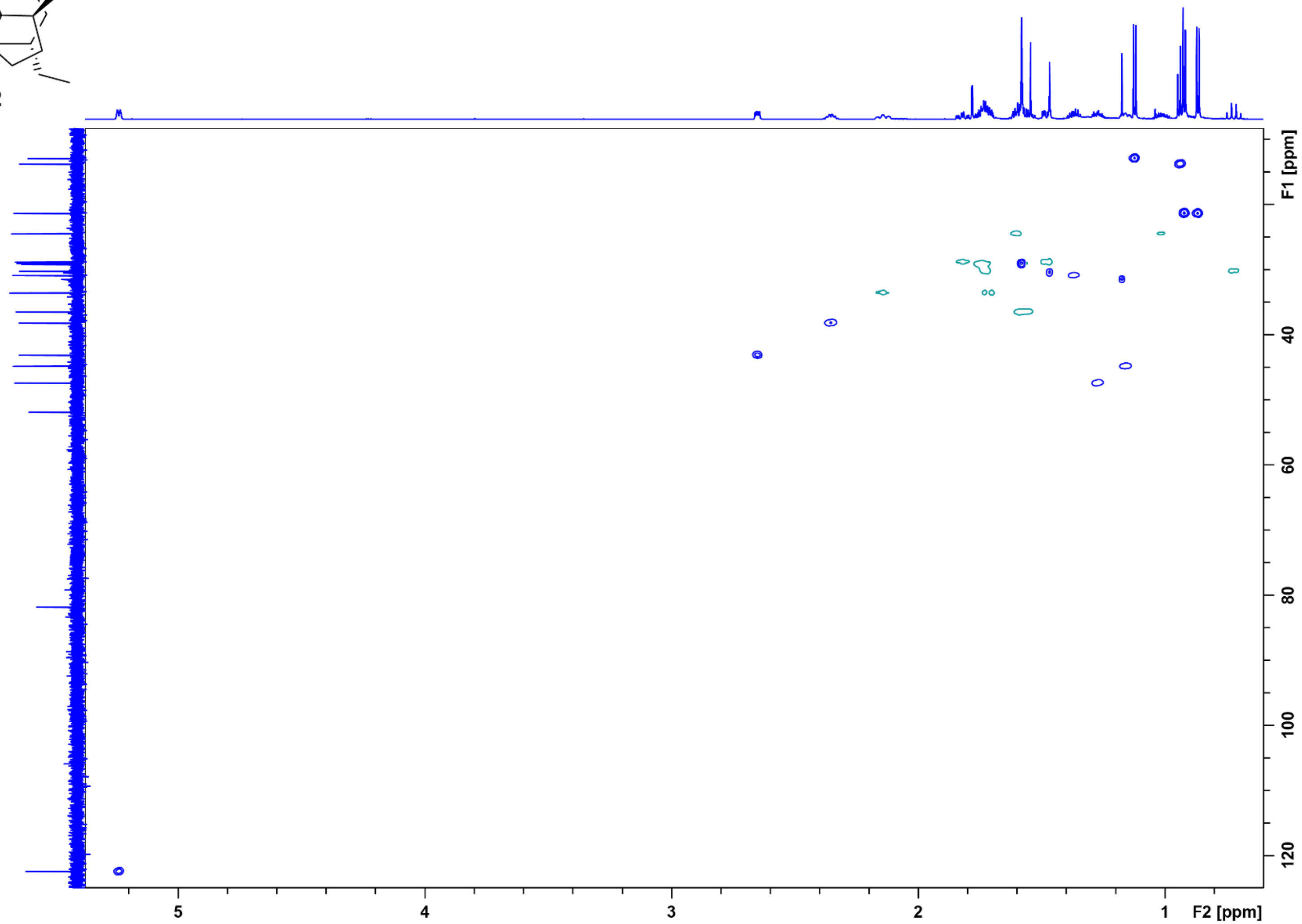

Figure S16. HSQC spectrum of **2** (C_6D_6).

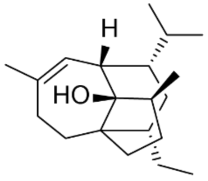

2

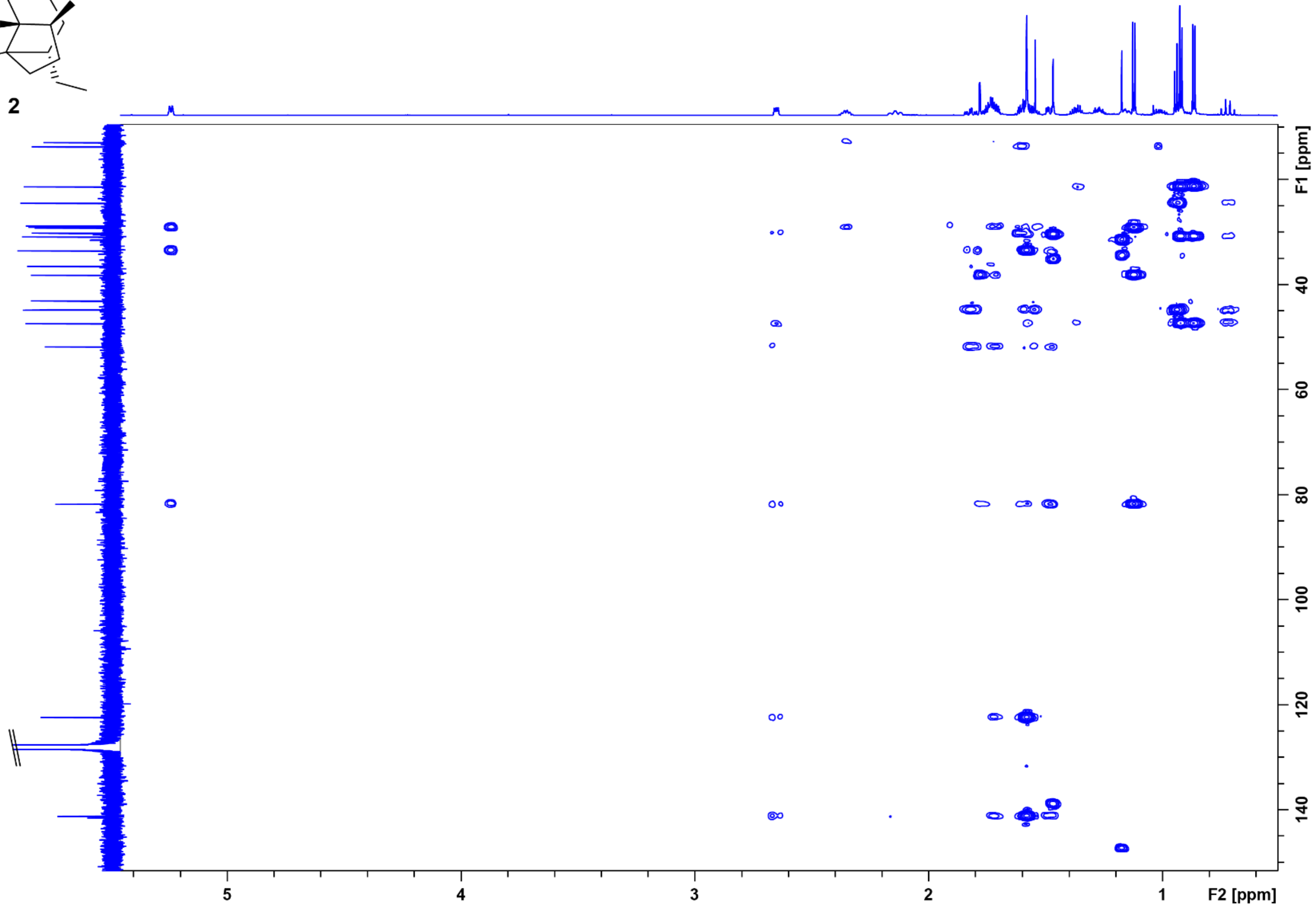

Figure S17. HMBC spectrum of **2** (C_6D_6).

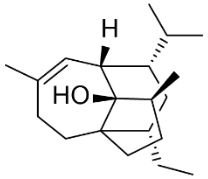

2

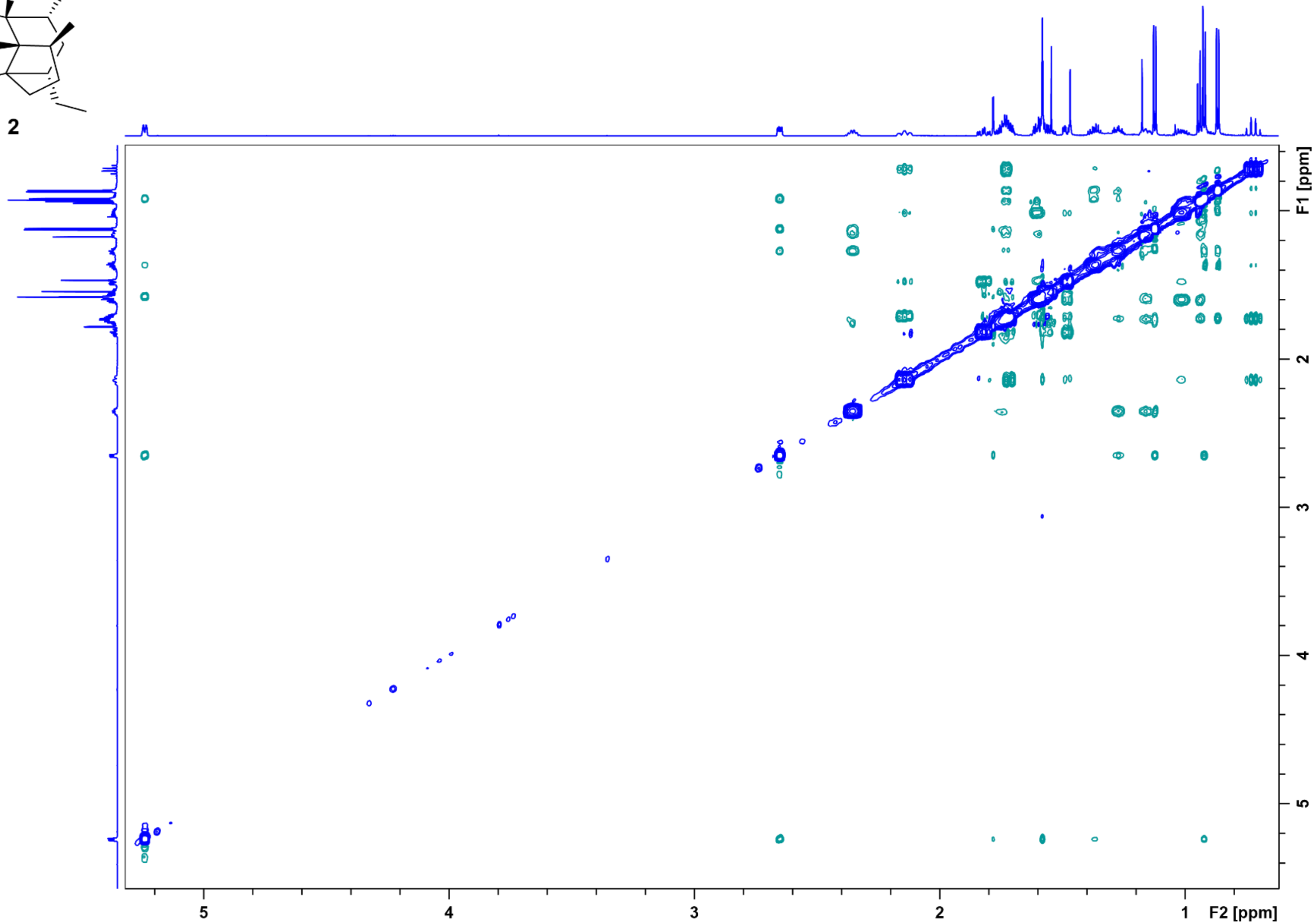

Figure S18. NOESY spectrum of **2** (C_6D_6).

Wanju-2,5-diene (3). Yield: 2.1 mg (7.7 μmol, 1.9%), from 200 mg (398.8 μmol) GGPP trisammonium salt. Spectroscopic data were identical to previously published data.[1] Optical rotary power: $[\alpha]_D^{25} = -12.4$ (*c* 0.21, CH_2Cl_2), lit. $[\alpha]_D^{25} = -14.3$ (*c* 1.91, C_6D_6).[1]

Thunbergol (4). Yield: 1.0 mg (3.4 μmol, 0.9%), from 200 mg (398.8 μmol) GGPP trisammonium salt. TLC (pentane/Et_2O = 1/1): $R_f = 0.71$. GC (HP5-MS): *I* = 2091. Optical rotary power: $[\alpha]_D^{25} = +35.0$ (*c* 0.1, $CHCl_3$).

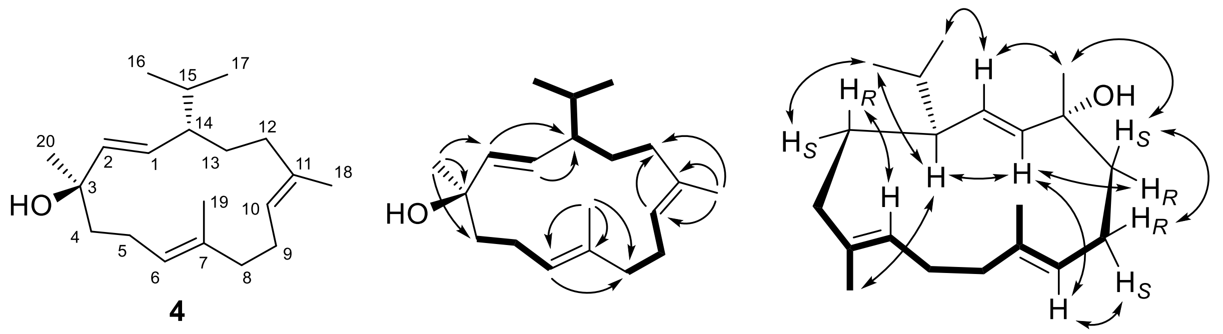

4

Figure S19. Structure elucidation of **4**. Bold: $^1H,^1H$-COSY, single headed arrows: key HMBC, and double headed arrows: NOESY correlations. Carbon numbering follows GGPP numbering to indicate the origin of each carbon. Diastereotopic hydrogens are labelled H_R (*pro-R*) and H_S (*pro-S*).

Table S5. NMR data of thunbergol (**4**) in C_6D_6 recorded at 298 K.

C[a]	type	^1H[b]	^{13}C[b,c]
1	CH	5.19 (dd, *J* = 15.6, 9.2)	128.47
2	CH	5.82 (d, *J* = 15.6)	139.39
3	C_q	–	72.16
4	CH_2	1.85 (ddd, *J* = 13.9, 9.4, 2.0, H_S)	43.58
		1.46 (ddd, *J* = 13.9, 9.3, 2.0, H_R)	
5	CH_2	2.53 (m, H_S)	23.00
		2.10 (m, H_R)	
6	CH	5.24 (br t, *J* = 7.6)	129.28
7	C_q	–	132.14
8	CH_2	2.08 (m)	39.56
		2.03 (m)	
9	CH_2	2.20 (m)	24.14
		2.07 (m)	
10	CH	5.16 (br m)	125.76
11	C_q	–	132.31
12	CH_2	2.05 (m, 2H)	37.25
13	CH_2	1.55 (m, H_S)	28.06
		1.21 (m, H_R)	
14	CH	1.73 (m)	46.41
15	CH	1.48 (m)	33.38
16	CH_3	0.84 (d, *J* = 6.8)	19.65
17	CH_3	0.88 (d, *J* = 6.7)	20.73
18	CH_3	1.51 (br s)	14.91
19	CH_3	1.54 (br s)	15.14
20	CH_3	1.26 (s)	28.45

[a] Carbon numbering as shown in main text. [b] Chemical shifts δ in ppm, multiplicity: s = singlet, d = doublet, t = triplet, sext = sextet, m = multiplet, br = broad, coupling constants *J* are given in Hertz. [c] ^{13}C-NMR data in $CDCl_3$ were reported previously.[80]

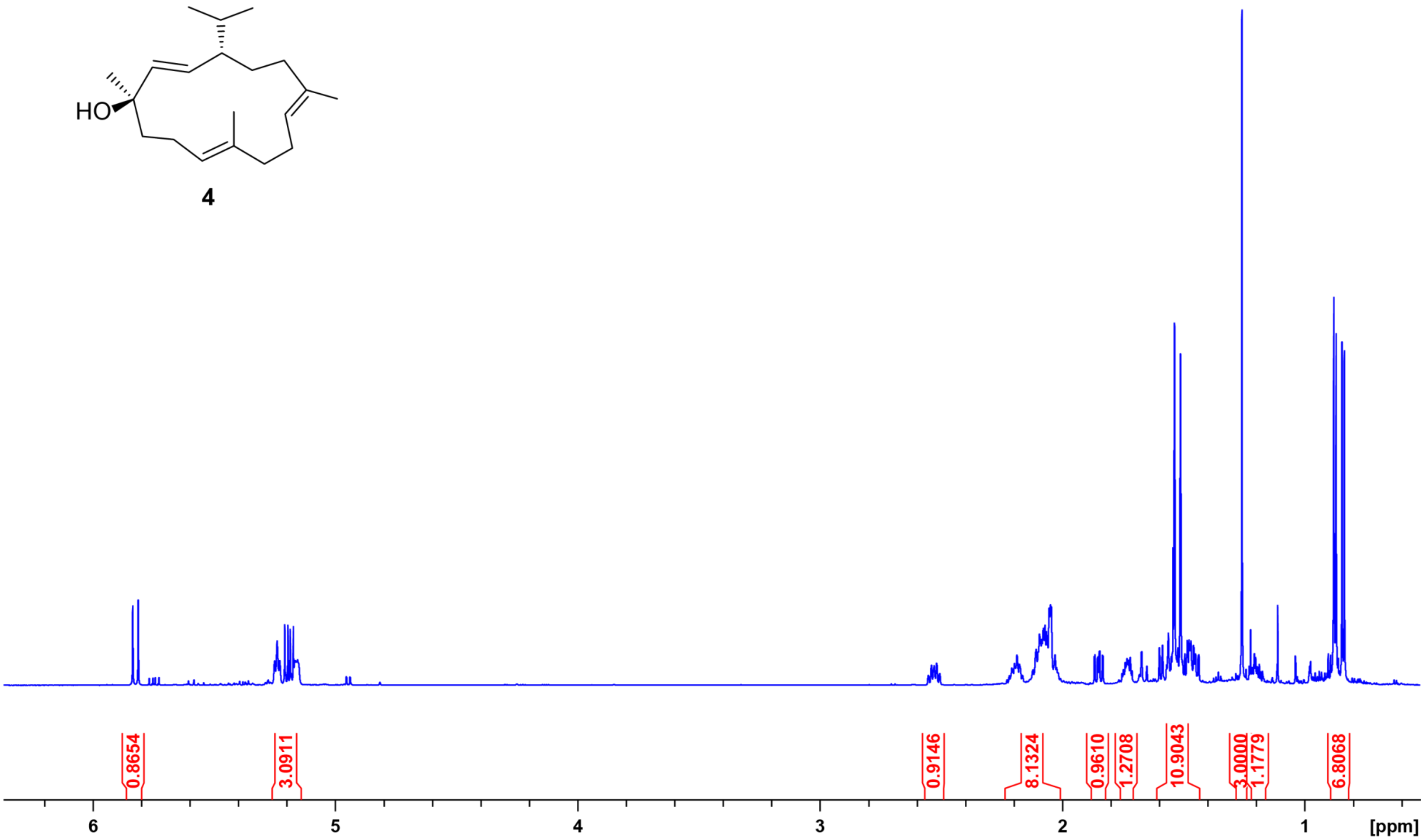

Figure S20. ^{1}H-NMR spectrum of **4** (700 MHz, C_6D_6).

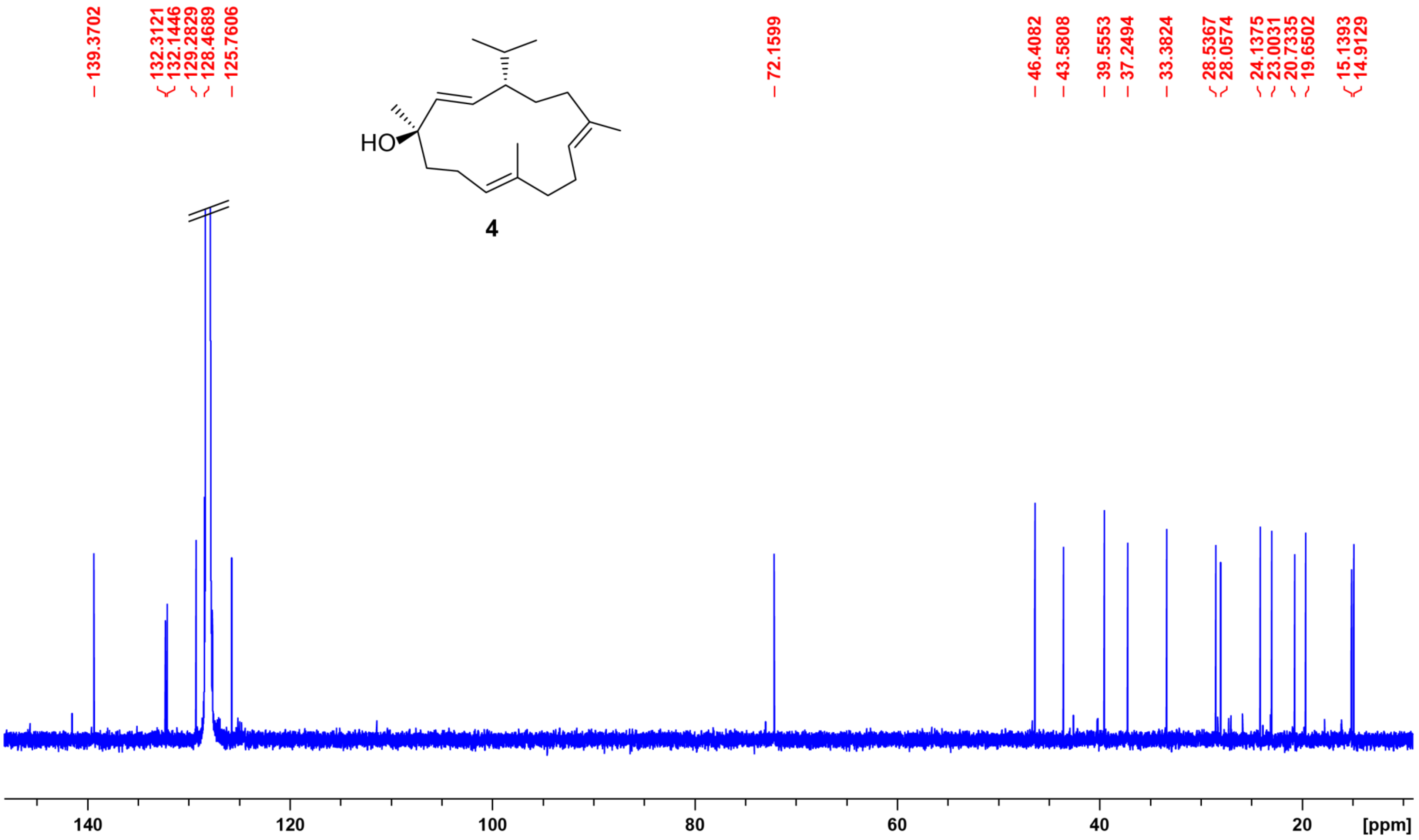

Figure S21. ^{13}C-NMR spectrum of **4** (176 MHz, C_6D_6).

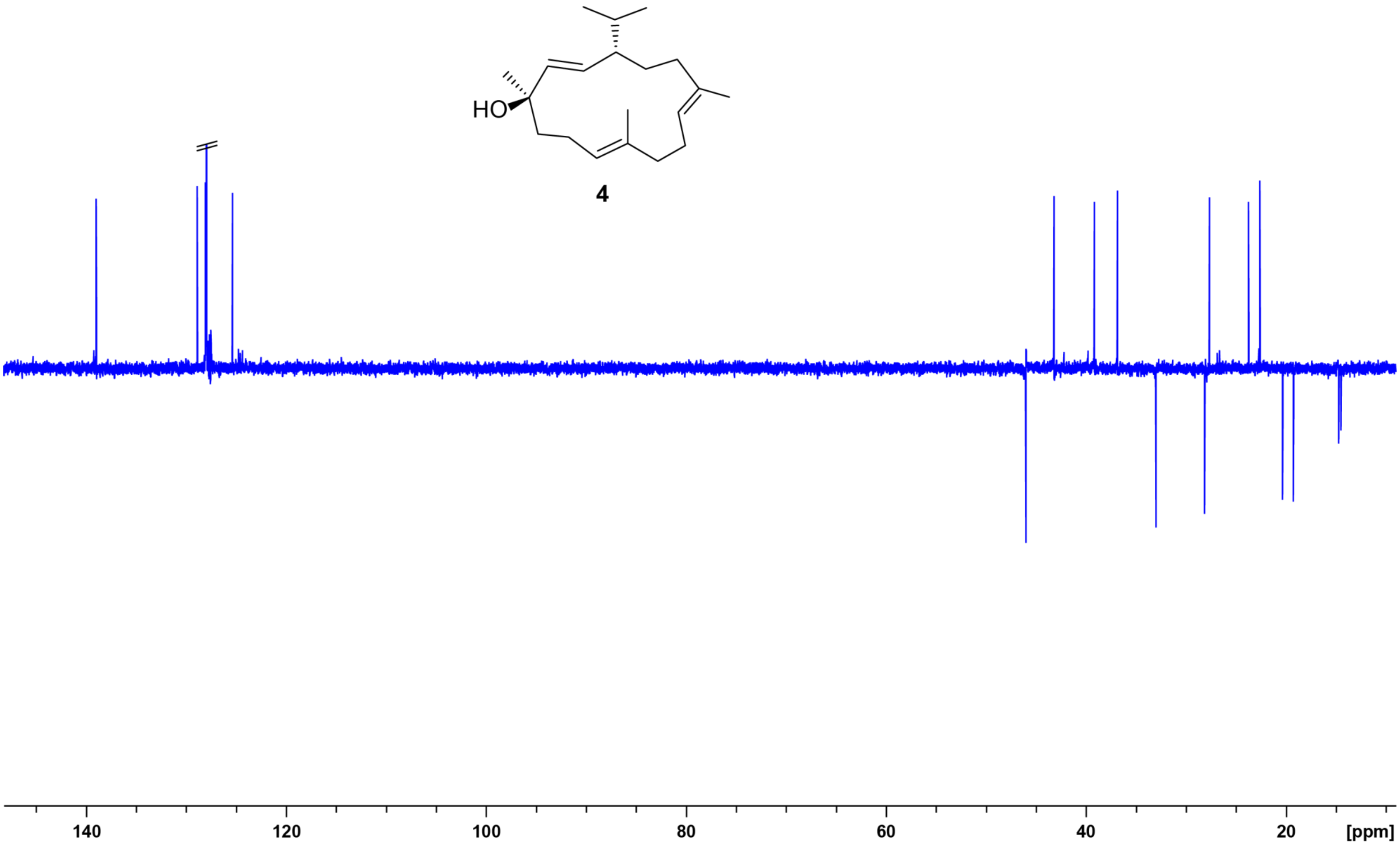

Figure S22. ^{13}C-DEPT spectrum of **4** (176 MHz, C_6D_6).

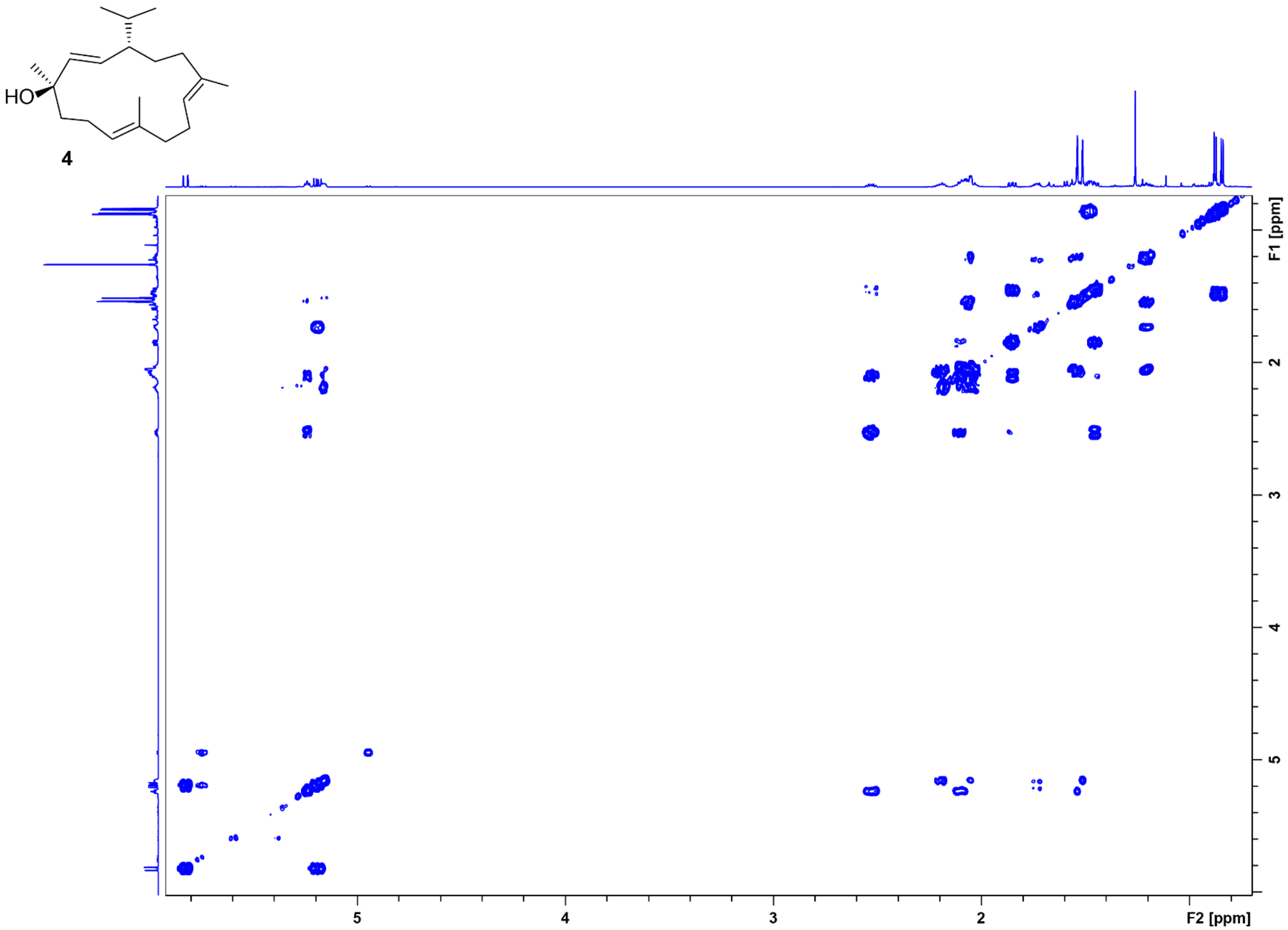

Figure 23. ^{1}H-^{1}H-COSY spectrum of **4** (700 MHz, C_6D_6).

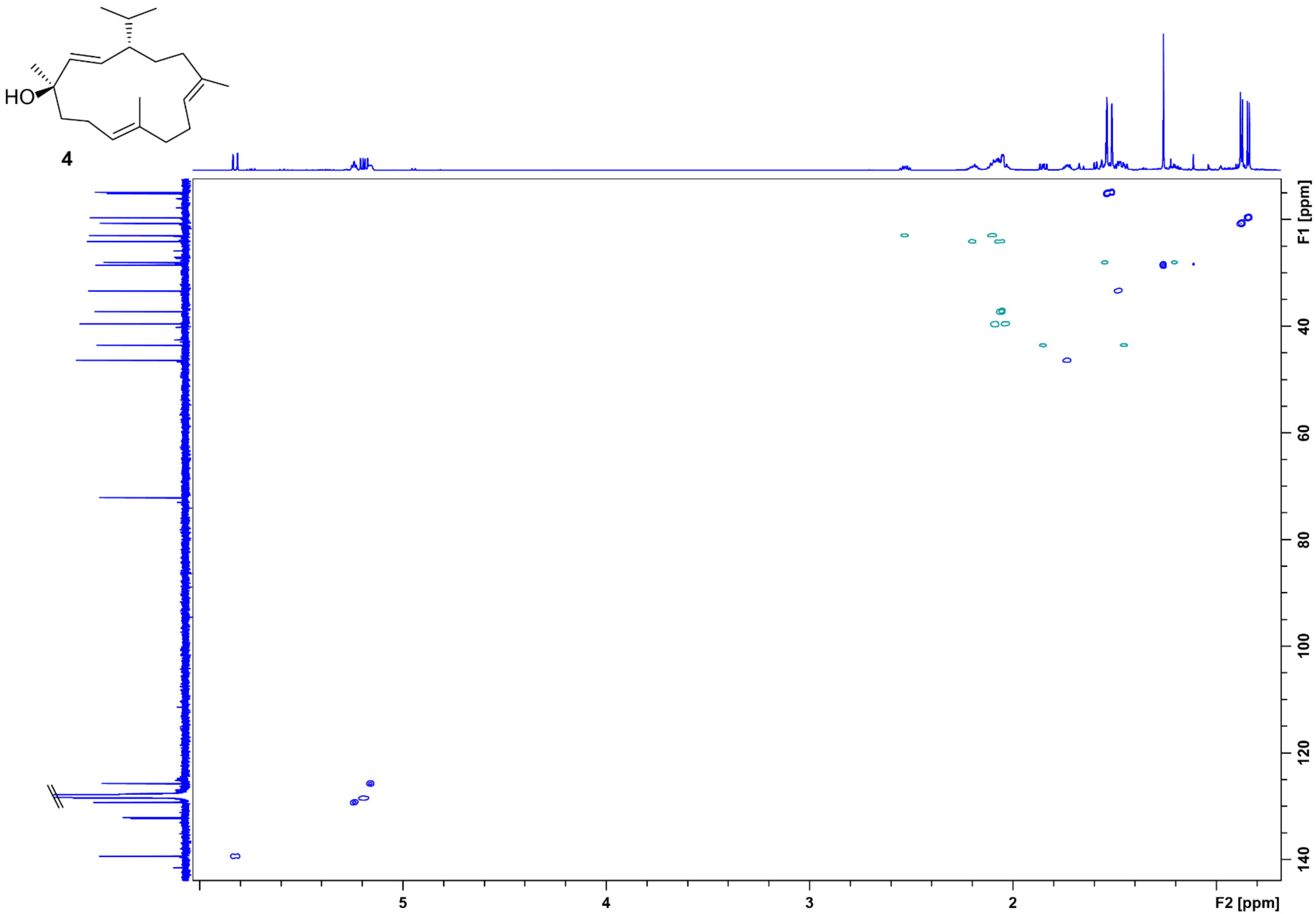

4

Figure S24. HSQC spectrum of **4** (C_6D_6).

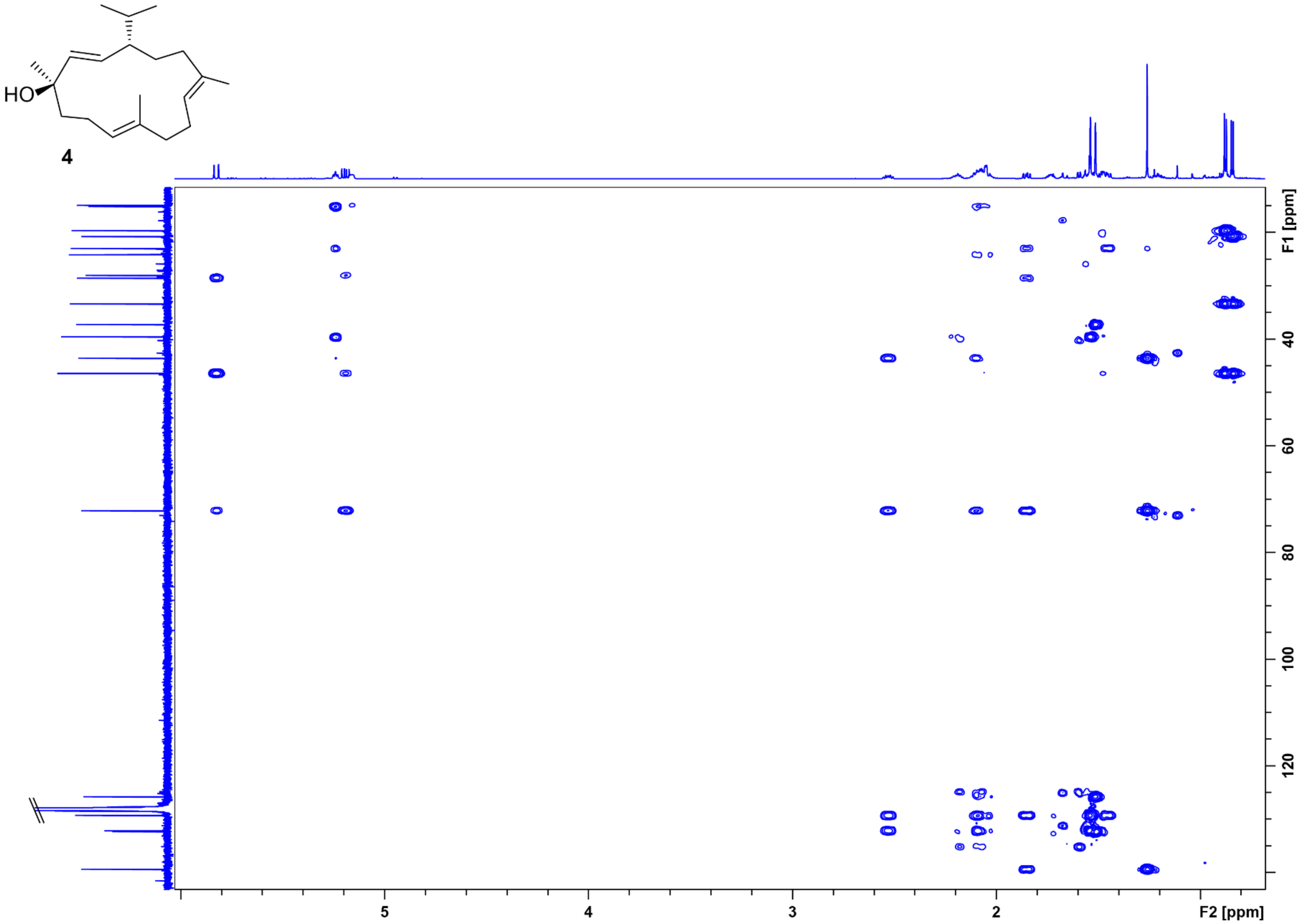

Figure S25. HMBC spectrum of **4** (C_6D_6).

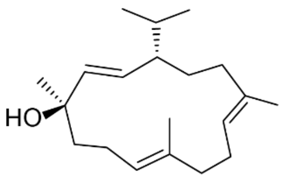

4

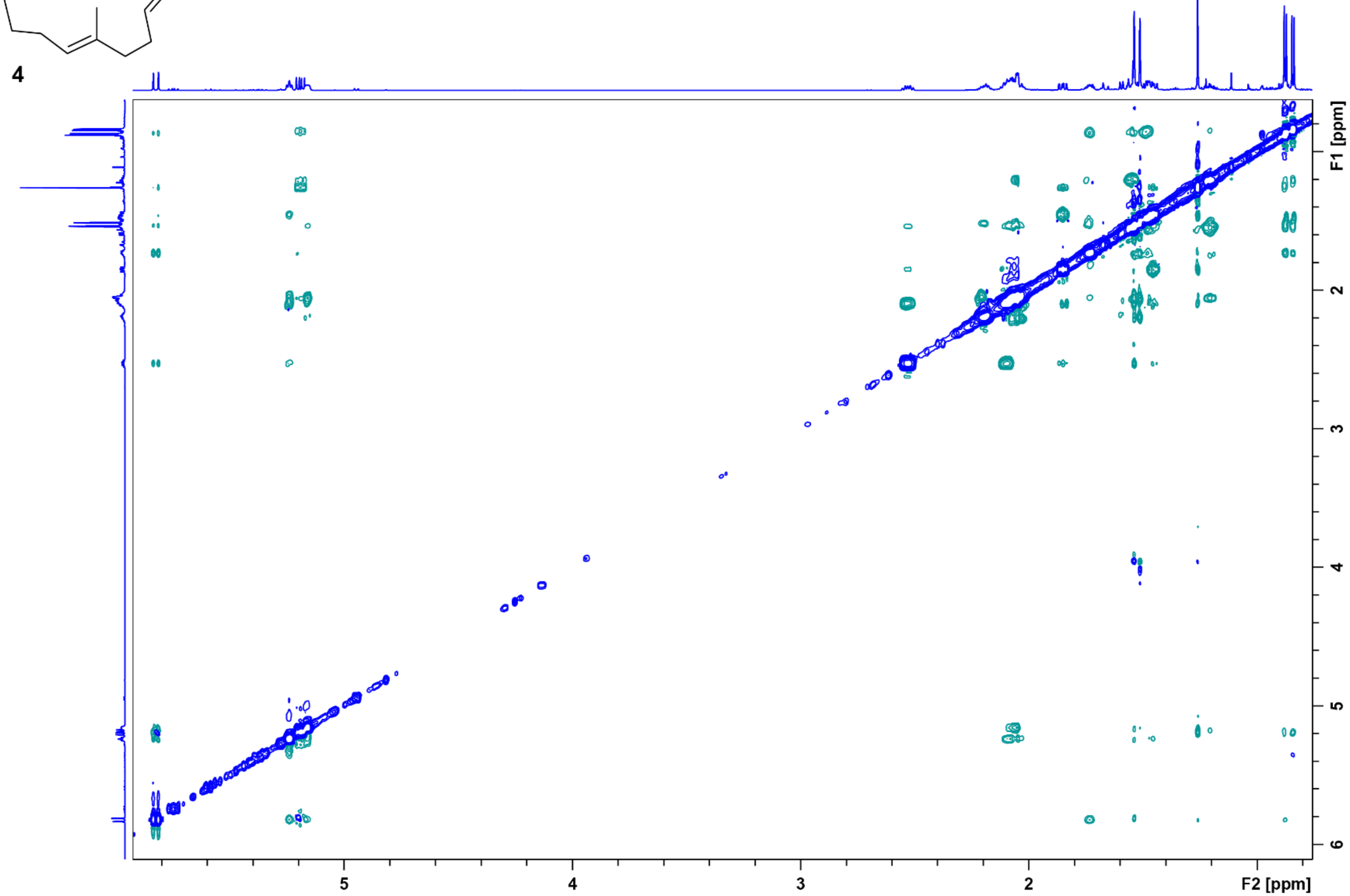

Figure S26. NOESY spectrum of **4** (C_6D_6).

Incubation experiments with labeled substrates

Isotopic labeling experiments were performed with ca. 1 mg labeled GGPP or its precursors in aqueous NH_4HCO_3 (1 mL; 25 mM in H_2O), Tris buffer (3 mL; 50 mM Tris, 1 mM $MgCl_2$, pH 7.6), incubation buffer (5 mL; 50 mM Tris, 8 mM $MgCl_2$, 8% glycerol, pH 7.6) and purified enzymes (each 1 mL) as listed in Table S6. The reaction mixtures were incubated at 28 °C overnight, and the products were extracted with C_6D_6 (600 µL + 300 µL) or hexane (300 µL), and then analyzed by NMR and/or GC/MS.

Table S6. Labeling experiments with CpPS.

entry	substrates	enzymes	results shown in
1	DMAPP + (*R*)-(1-^{13}C,1-^{2}H)IPP[29]	GGPPS, CpPS	Figures S27, S28 and S35
2	DMAPP + (*S*)-(1-^{13}C,1-^{2}H)IPP[29]	GGPPS, CpPS	Figures S27, S28 and S35
3	DMAPP + (*E*)-(4-^{13}C,4-^{2}H)IPP[12]	GGPPS, CpPS	Figures S29, S30 and S34
4	DMAPP + (*Z*)-(4-^{13}C,4-^{2}H)IPP[12]	GGPPS, CpPS	Figures S29, S30 and S34
5	(*R*)-(1-^{2}H)GGPP[13]	CpPS	Figure S31
6	(*S*)-(1-^{2}H)GGPP[13]	CpPS	Figure S31
7	(3-^{13}C,2-^{2}H)GPP + IPP[5]	GGPPS, CpPS	Figure S32
8	(3-^{13}C,2-^{2}H)FPP + IPP[23]	GGPPS, CpPS	Figure S33
9	DMAPP + (3-^{13}C,4-$^{2}H_2$)IPP[17]	GGPPS, CpPS	Figure S34
10	(1-^{13}C)GGPP[81]	CpPS	Figure S36
11	FPP + (2-^{13}C)IPP[56]	GGPPS, CpPS	Figure S37
12	FPP + (3-^{13}C)IPP[13]	GGPPS, CpPS	Figure S38
13	FPP + (4-^{13}C)IPP[13]	GGPPS, CpPS	Figure S39
14	(1-^{13}C)FPP + IPP[16]	GGPPS, CpPS	Figure S40
15	(2-^{13}C)FPP + IPP[16]	GGPPS, CpPS	Figure S41
16	(3-^{13}C)FPP + IPP[16]	GGPPS, CpPS	Figure S42
17	(4-^{13}C)FPP + IPP[16]	GGPPS, CpPS	Figure S43
18	(5-^{13}C)FPP + IPP[16]	GGPPS, CpPS	Figure S44
19	(6-^{13}C)FPP + IPP[16]	GGPPS, CpPS	Figure S45
20	(7-^{13}C)FPP + IPP[16]	GGPPS, CpPS	Figure S46
21	(8-^{13}C)FPP + IPP[16]	GGPPS, CpPS	Figure S47
22	(9-^{13}C)FPP + IPP[16]	GGPPS, CpPS	Figure S48
23	(10-^{13}C)FPP + IPP[16]	GGPPS, CpPS	Figure S49
24	(11-^{13}C)FPP + IPP[16]	GGPPS, CpPS	Figure S50
25	(12-^{13}C)FPP + IPP[16]	GGPPS, CpPS	Figure S51
26	(9-^{13}C)GPP + IPP[57]	GGPPS, CpPS	Figure S52
27	(14-^{13}C)FPP + IPP[16]	GGPPS, CpPS	Figure S53
28	(15-^{13}C)FPP + IPP[16]	GGPPS, CpPS	Figure S54
29	(20-^{13}C)GGPP[13]	GGPPS, CpPS	Figure S55
30	DMAPP + (*R*)-(1-^{13}C,1-^{2}H)IPP[29]	GGPPS, CpPS(I66F)	Figures S101, S102 and S103
31	DMAPP + (*S*)-(1-^{13}C,1-^{2}H)IPP[29]	GGPPS, CpPS(I66F)	Figures S101, S102 and S103
32	DMAPP + (*E*)-(4-^{13}C,4-^{2}H)IPP[12]	GGPPS, CpPS(I66F)	Figures S104, S105 and S106
33	DMAPP + (*Z*)-(4-^{13}C,4-^{2}H)IPP[12]	GGPPS, CpPS(I66F)	Figures S104, S105 and S106
34	(*R*)-(1-^{2}H)GGPP[13]	CpPS(I66F)	Figure S107
35	(*S*)-(1-^{2}H)GGPP[13]	CpPS(I66F)	Figure S107

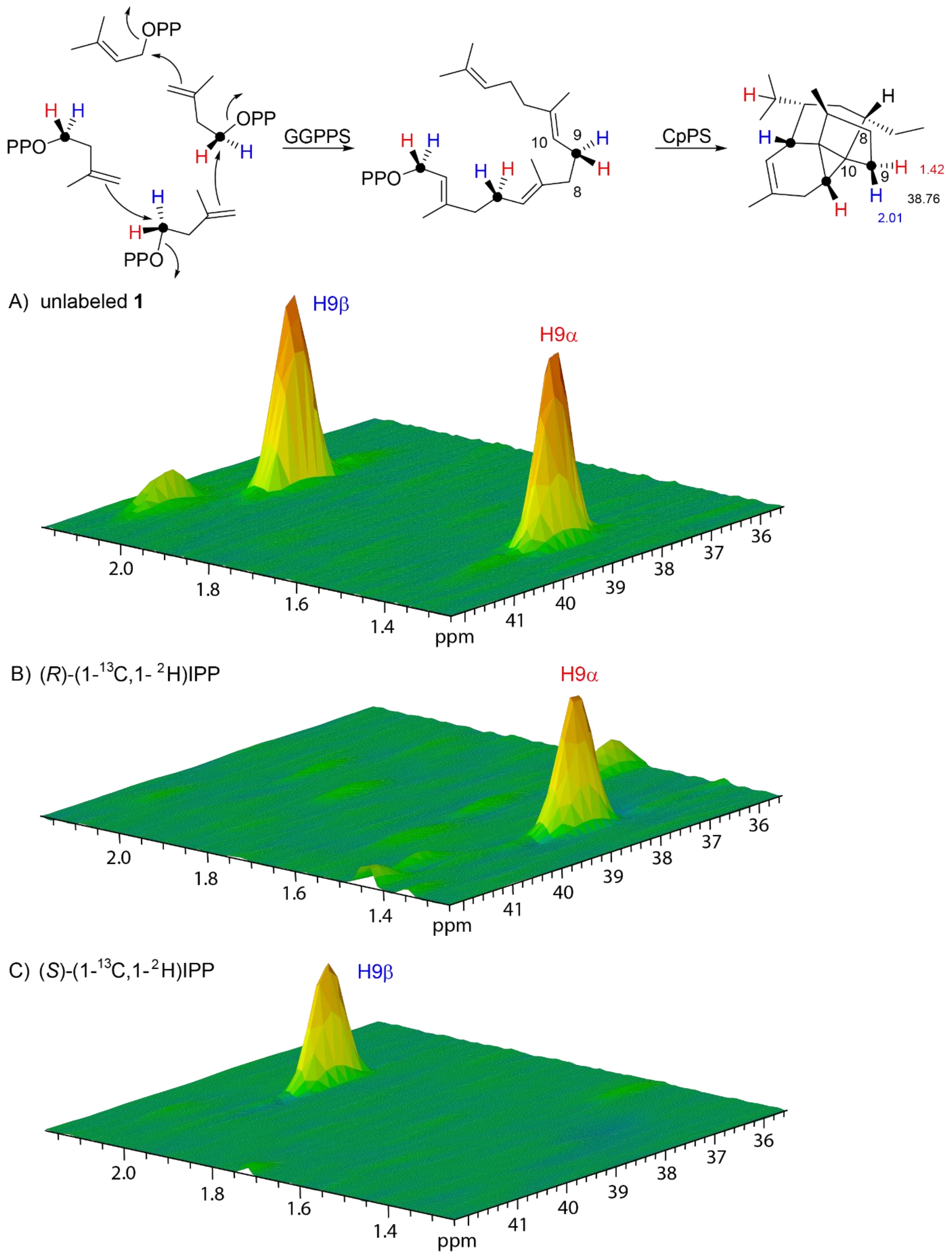

Figure S27. The absolute configuration of **1**. Partial HSQC spectra of A) unlabeled **1**, B) labeled **1** obtained from DMAPP and (*R*)-(1-^{13}C,1-^{2}H)IPP (blue H = ^{2}H), and C) labeled **1** from DMAPP and (*S*)-(1-^{13}C,1-^{2}H)IPP (red H = ^{2}H). The specific incorporation at C9 with known configuration at this carbon in experiments B) and C) together with the NOESY based assignments of relative orientations of H9α and H9β (Figure S3) with respect to the naturally present stereogenic centers in **1** allows to assign the shown absolute configuration for **1**. Black dots represent ^{13}C-labeled carbons.

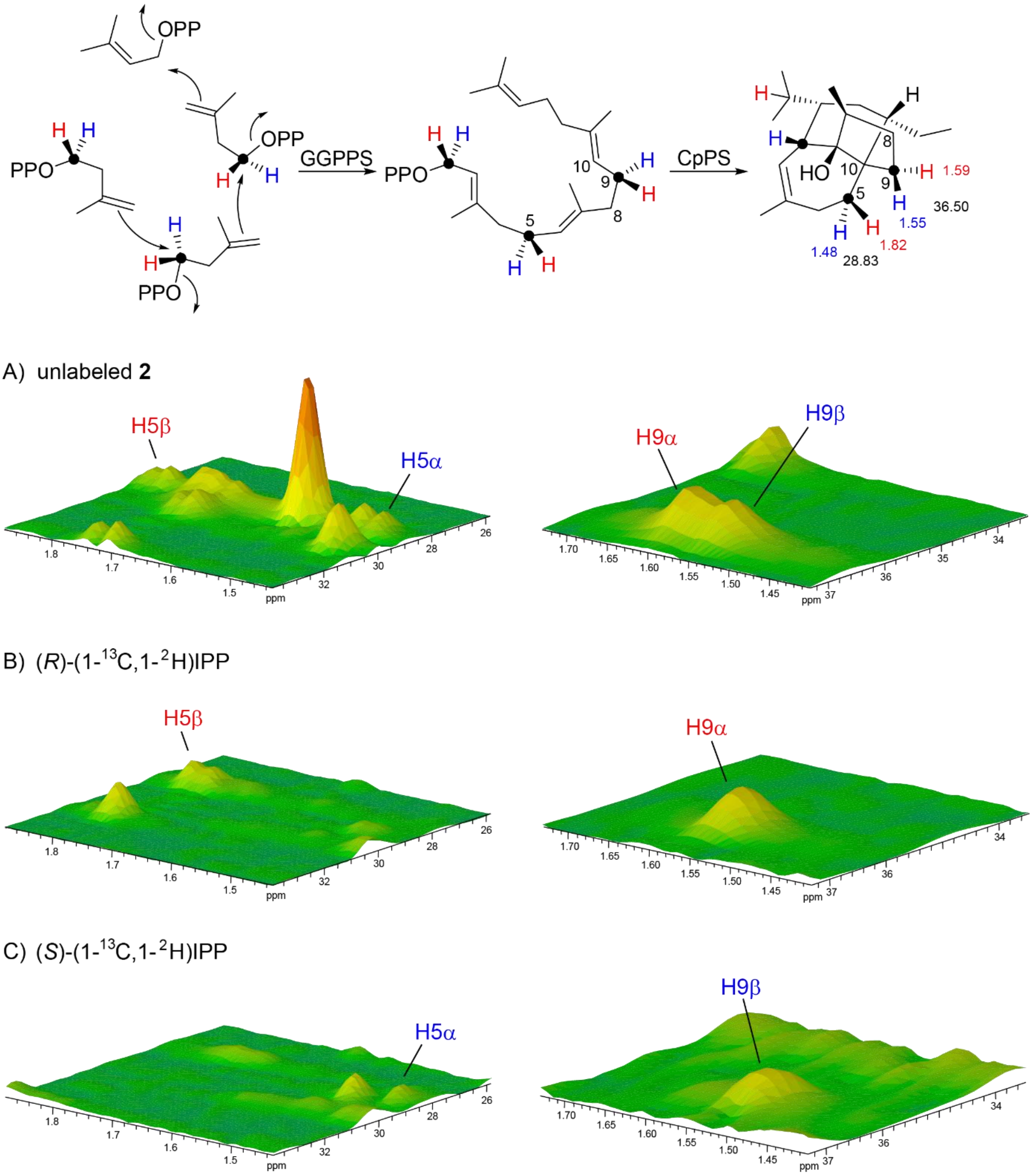

Figure S28. The absolute configuration of **2**. Partial HSQC spectra of A) unlabeled **2**, B) labeled **2** obtained from DMAPP and (*R*)-(1-^{13}C,1-^{2}H)IPP (blue H = ^{2}H), and C) labeled **2** from DMAPP and (*S*)-(1-^{13}C,1-^{2}H)IPP (red H = ^{2}H). The specific incorporation at C5 and C9 with known configuration at these carbons in experiments B) and C) together with the NOESY based assignments of relative orientations of H5α, H5β, H9α and H9β (Figure S11) with respect to the naturally present stereogenic centers in **2** allows to assign the shown absolute configuration for **2**. Black dots represent ^{13}C-labeled carbons.

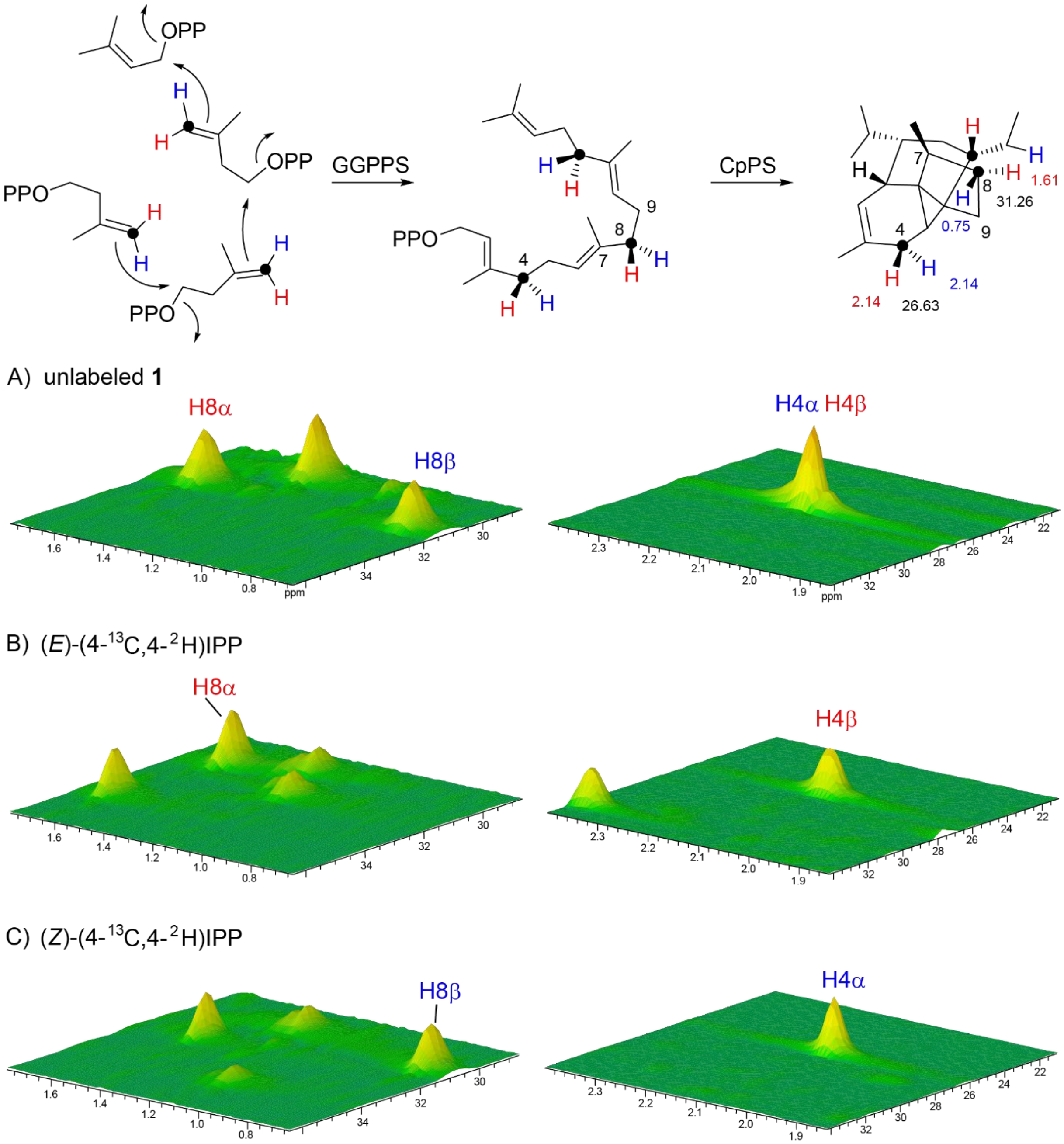

Figure S29. The absolute configuration of **1**. Partial HSQC spectra of A) unlabeled **1**, B) labeled **1** obtained from DMAPP and (*E*)-(4-^{13}C,4-^{2}H)IPP (blue H = ^{2}H), and C) labeled **1** from DMAPP and (*Z*)-(4-^{13}C,4-^{2}H)IPP (red H = ^{2}H). The specific incorporation at C8 with known configuration at this carbons in experiments B) and C) together with the NOESY based assignments of relative orientations of H8α and H8β (Figure S3) with respect to the naturally present stereogenic centers in **2** allows to assign the shown absolute configuration for **2**. Signals for H4α and H4β cannot be used for this assignment, because they show the same chemical shift. Black dots represent ^{13}C-labeled carbons.

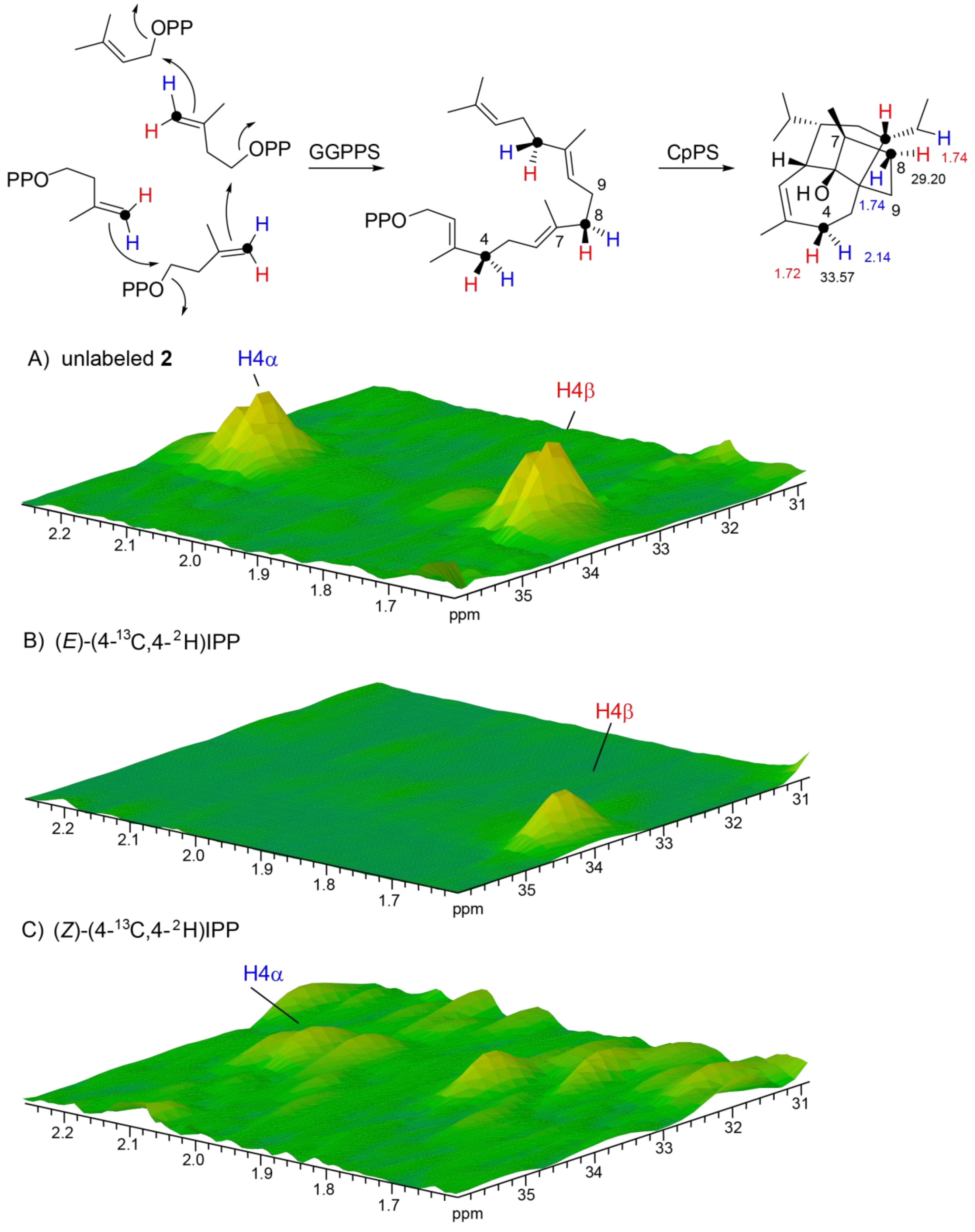

Figure S30. The absolute configuration of **2**. Partial HSQC spectra of A) unlabeled **2**, B) labeled **2** obtained from DMAPP and (*E*)-(4-^{13}C,4-^{2}H)IPP (blue H = ^{2}H), and C) labeled **2** from DMAPP and (*Z*)-(4-^{13}C,4-^{2}H)IPP (red H = ^{2}H). The specific incorporation at C4 with known configuration at this carbon in experiments B) and C) together with the NOESY based assignments of relative orientations of H4α and H4β (Figure S11) with respect to the naturally present stereogenic centers in **2** allows to assign the shown absolute configuration for **2**. Black dots represent ^{13}C-labeled carbons.

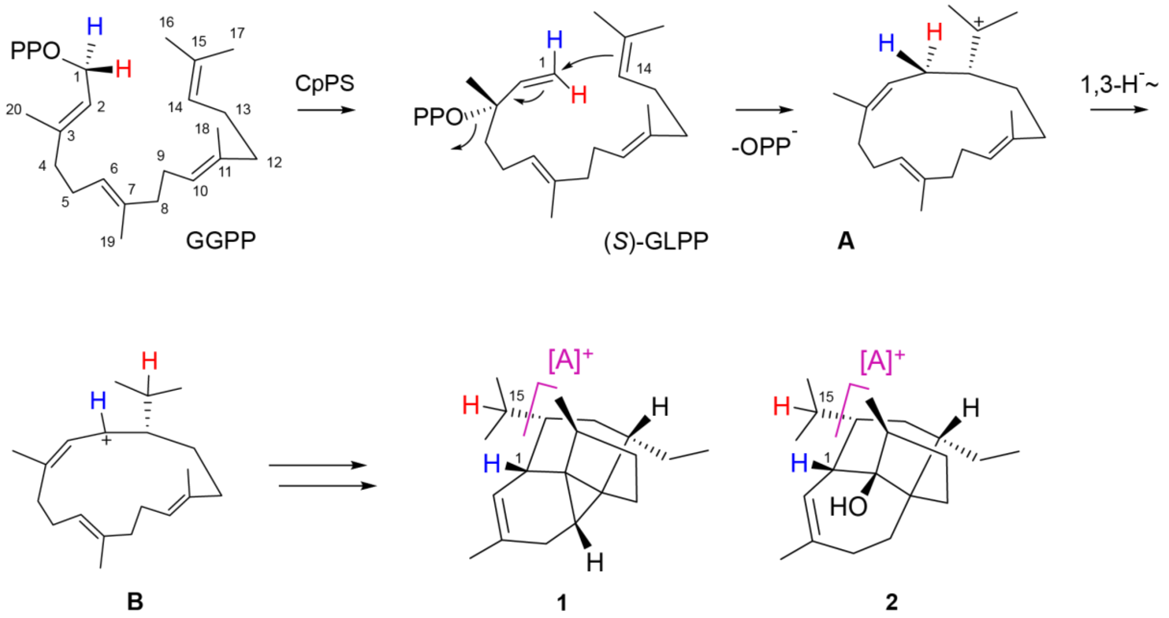

A) (*R*)-(1-^{2}H)GGPP

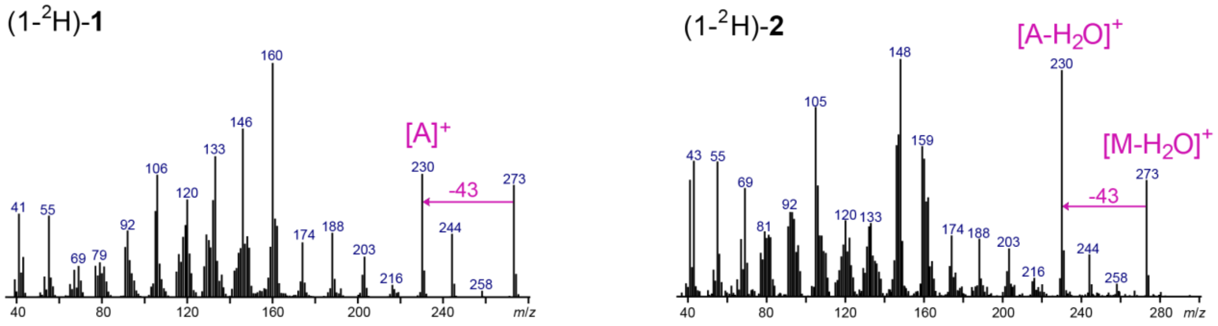

B) (*S*)-(1-^{2}H)GGPP

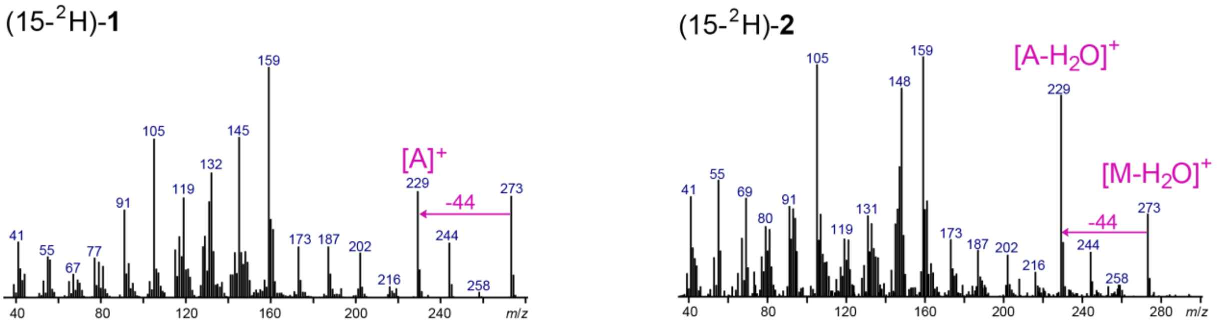

Figure S31. The absolute configurations of **1** and **2**. A) Mass spectra of labeled **1** and **2** obtained from (*R*)-(1-^{2}H)GGPP (blue H = ^{2}H). B) Mass spectra of labeled **1** and **2** obtained from (*S*)-(1-^{2}H)GGPP (red H = ^{2}H). The fragment ion $[A]^+$ arises by cleavage of an *i*Pr group, showing retainment of deuterium from (*R*)-(1-^{2}H)GGPP and loss of deuterium from (*S*)-(1-^{2}H)GGPP. These findings are explainable through the shown stereochemical course for the steps from GGPP to **B** and thus point to the shown absolute configurations of **1** and **2**.

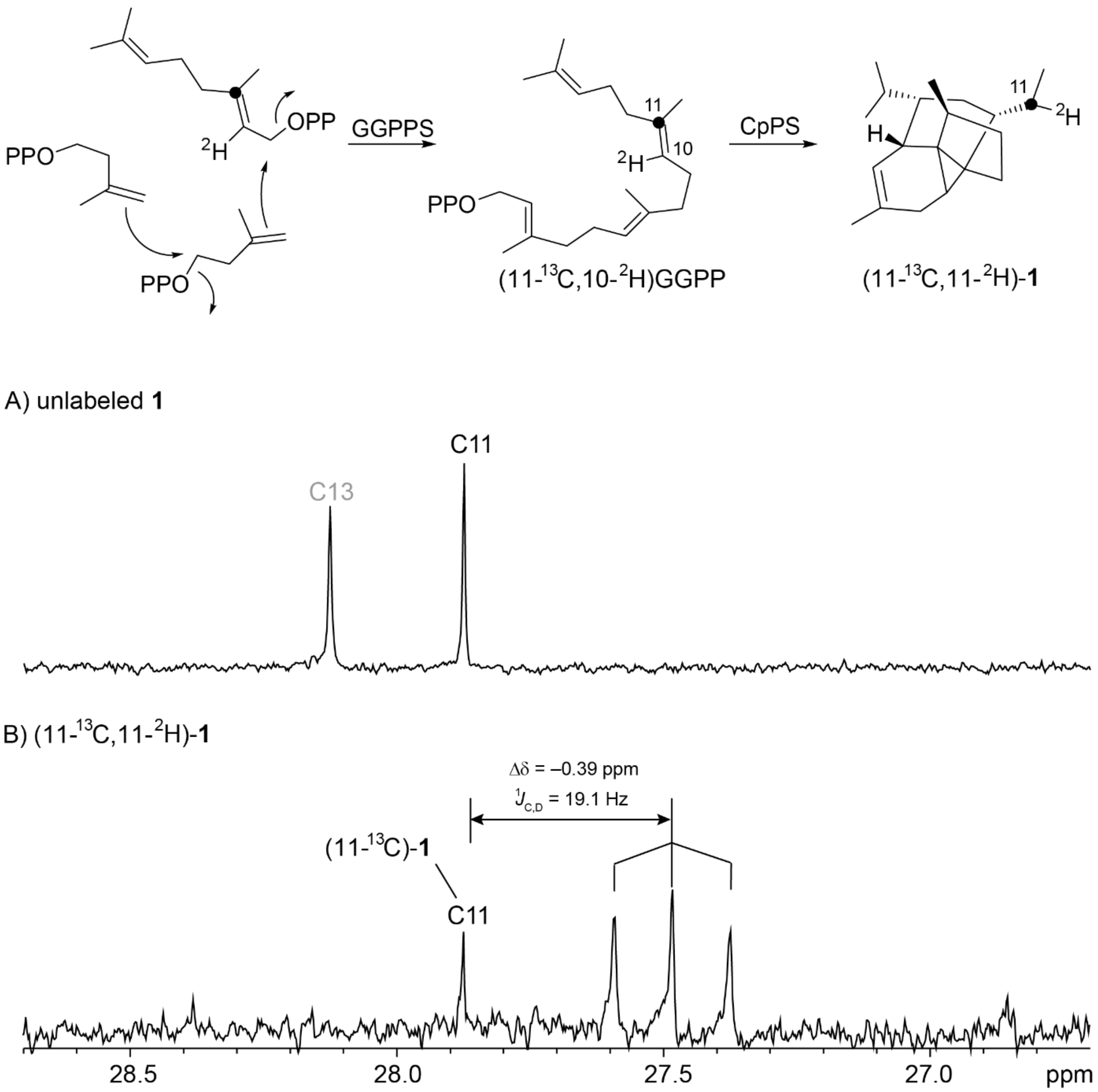

Figure S32. The 1,2-hydride shift from **C1** to **D1** (blue path in Scheme 1 of main text) or **E2** to **F2** (red path). ^{13}C NMR spectra show A) the signal for C11 of unlabeled **1**, B) the signal for the deuterated C11 of labeled **1** obtained from (3-^{13}C,2-^{2}H)GPP and IPP. The coupling constant of the triplet and the slight upfield chemical shift value indicate a direct ^{2}H-^{13}C bond and support the 1,2-hydride migration. The residual singlet signal for C11 in B) is a result of incomplete deuteration at C2 of the labeled substrate (3-^{13}C,2-^{2}H)GPP. Black dots indicate ^{13}C-labeled carbons.

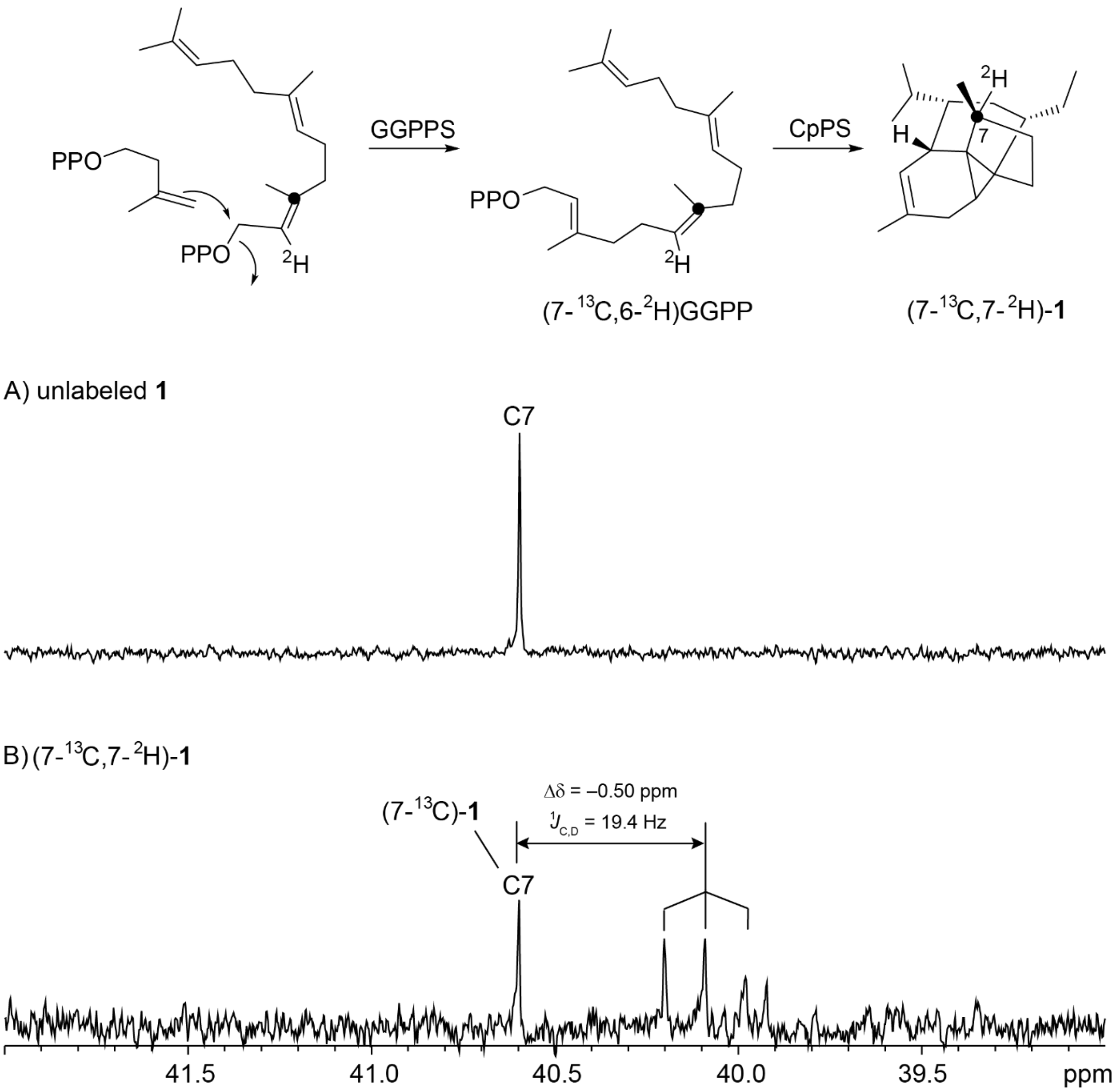

Figure S33. The 1,2-hydride shift from **E1** to **F1** (blue path in Scheme 1 of main text) or **C2** to **D2** (red path). ^{13}C NMR spectra show A) the signal for C7 of unlabeled **1**, B) the signal for the deuterated C7 of labeled **1** obtained from (3-^{13}C,2-^{2}H)FPP and IPP. The coupling constant of the triplet and the slight upfield chemical shift value indicate a direct ^{2}H-^{13}C bond and support the 1,2-hydride migration. The residual singlet signal for C7 in B) is a result of incomplete deuteration at C2 of the labeled substrate (3-^{13}C,2-^{2}H)FPP. Black dots indicate ^{13}C-labeled carbons.

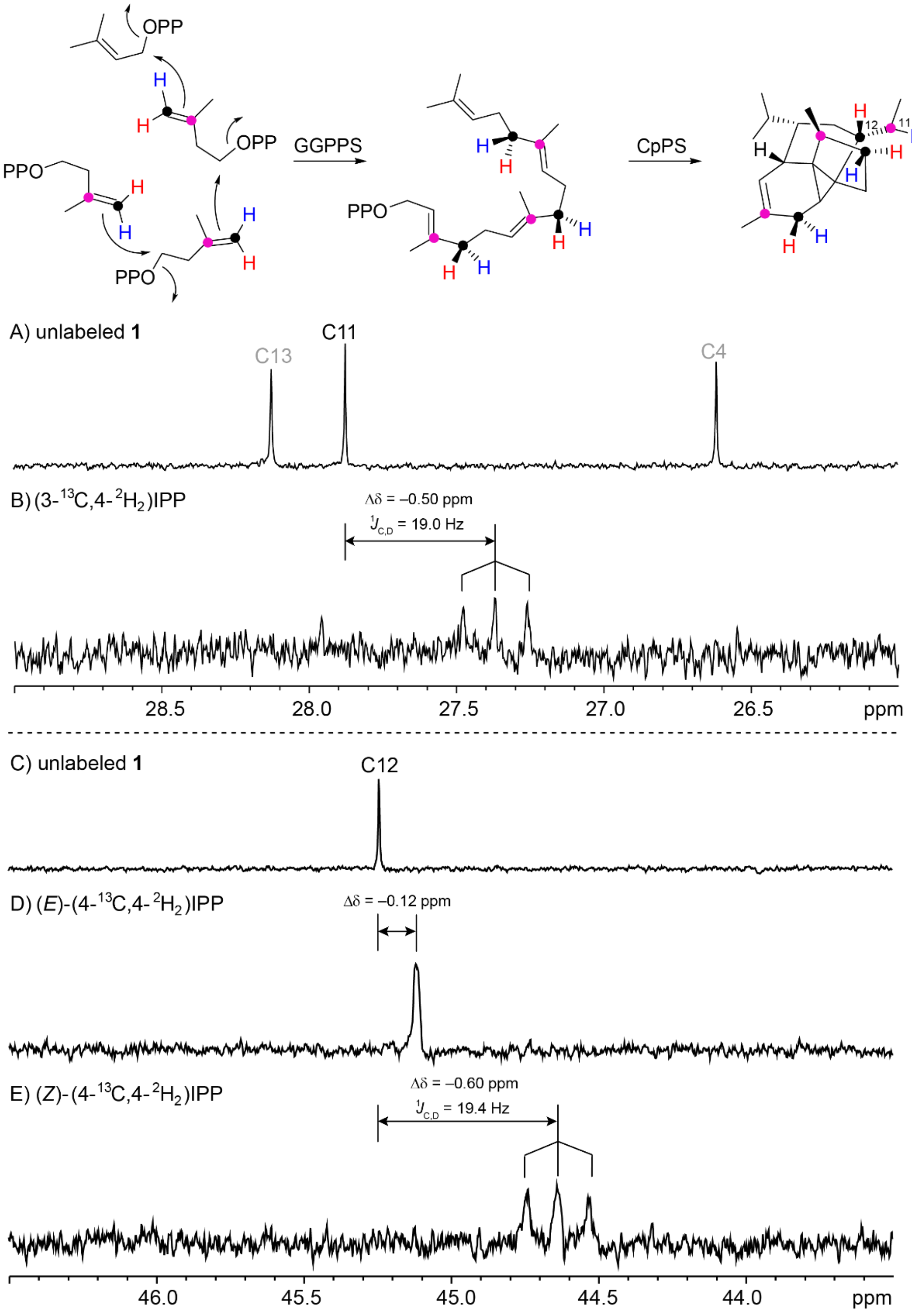

Figure S34. The stereochemical course of the rearrangement from **F1** to **H**. ^{13}C NMR spectra show A) the signal for C11 of unlabeled **1**, B) the signal for the deuterated C11 of labeled **1** obtained from DMAPP and (3-^{13}C,4-^{2}H$_2$)IPP (blue and red H = ^{2}H, purple dot = ^{13}C). The upfield shifted triplet for C11 indicates that one of the two hydrogens migrates from C12 to C11. C) The signal for C12 of unlabeled **1**, D) the signal for C12 of labeled **1** obtained from DMAPP and (*E*)-(4-^{13}C,4-^{2}H)IPP (blue H = ^{2}H, black dot = ^{13}C). The singlet for C12 indicates that the blue H (12-*pro-S* in GGPP) shifts away from C12 and the slight upfield shift is in agreement with deuterium located at a neighboring carbon (C11). E) The signal for the deuterated C12 of labeled **1** obtained from DMAPP and (*Z*)-(4-^{13}C,4-^{2}H)IPP (red H = ^{2}H, black dot = ^{13}C). The coupling constant of the triplet and the slight upfield chemical shift value for C12 indicate a direct ^{2}H-^{13}C bond. Thus, the red H (12-*pro-R* in GGPP) stays bound to C12.

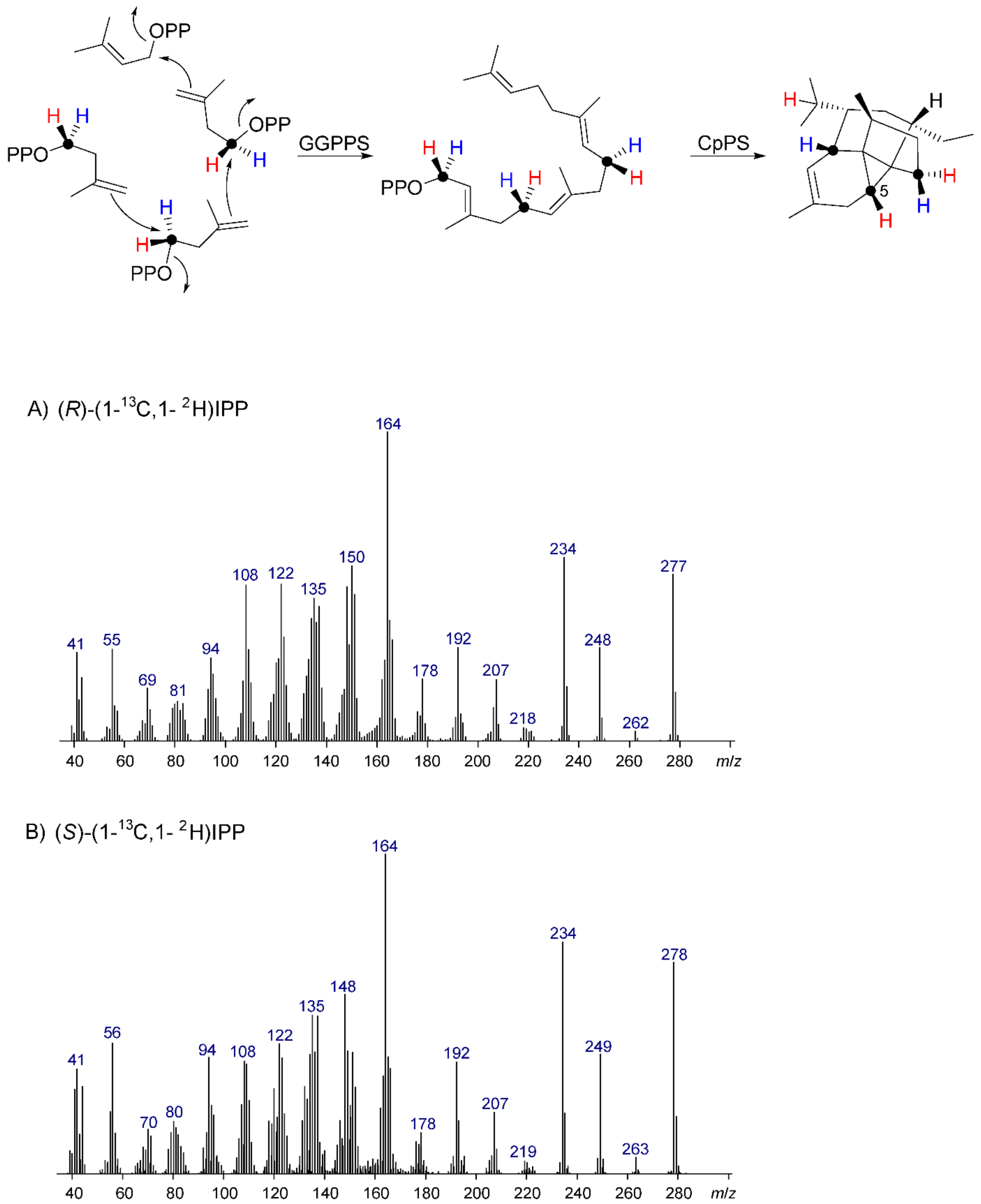

Figure S35. The deprotonation from **J** to **1**. A) Mass spectrum of labeled **1** obtained from DMAPP and (*R*)-(1-^{13}C,1-^{2}H)IPP (blue H = ^{2}H), B) mass spectrum of labeled **1** obtained from DMAPP and (*S*)-(1-^{13}C,1-^{2}H)IPP (red H = ^{2}H). The molecular ions at *m*/*z* = 277 in A) and at *m*/*z* = 278 in B) indicate loss of deuterium from (*R*)-(1-^{13}C,1-^{2}H)IPP and retainment from (*S*)-(1-^{13}C,1-^{2}H)IPP. Thus, the 5-*pro-R* hydrogen of GGPP is lost and the 5-*pro-S* hydrogen is retained in the deprotonation from **J** to **1**.

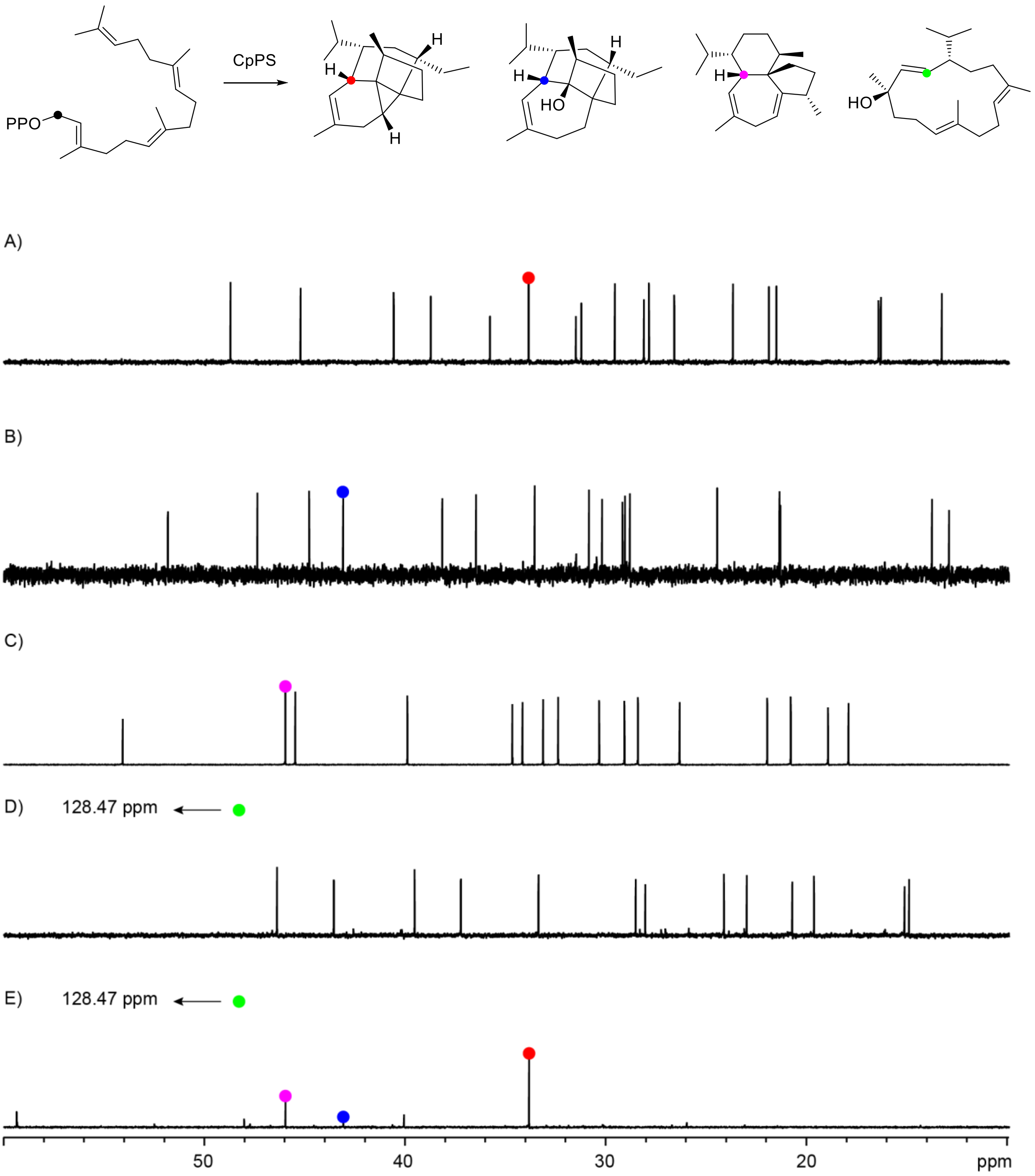

Figure S36. Enzymatic conversion of (1-^{13}C)GGPP with CpPS. ^{13}C-NMR spectra of A) unlabelled **1**, B) unlabelled **2**, C) unlabelled **3**, D) unlabelled **4** and E) the mixture of enzyme products obtained from (1-^{13}C)GGPP with CpPS. Coloured dots correlate the carbons of compounds **1** – **4** to the peaks observed in the ^{13}C-NMR spectra. In experiment E) an additional signal at 128.47 ppm for C1 of compound **4** is observed (not shown).

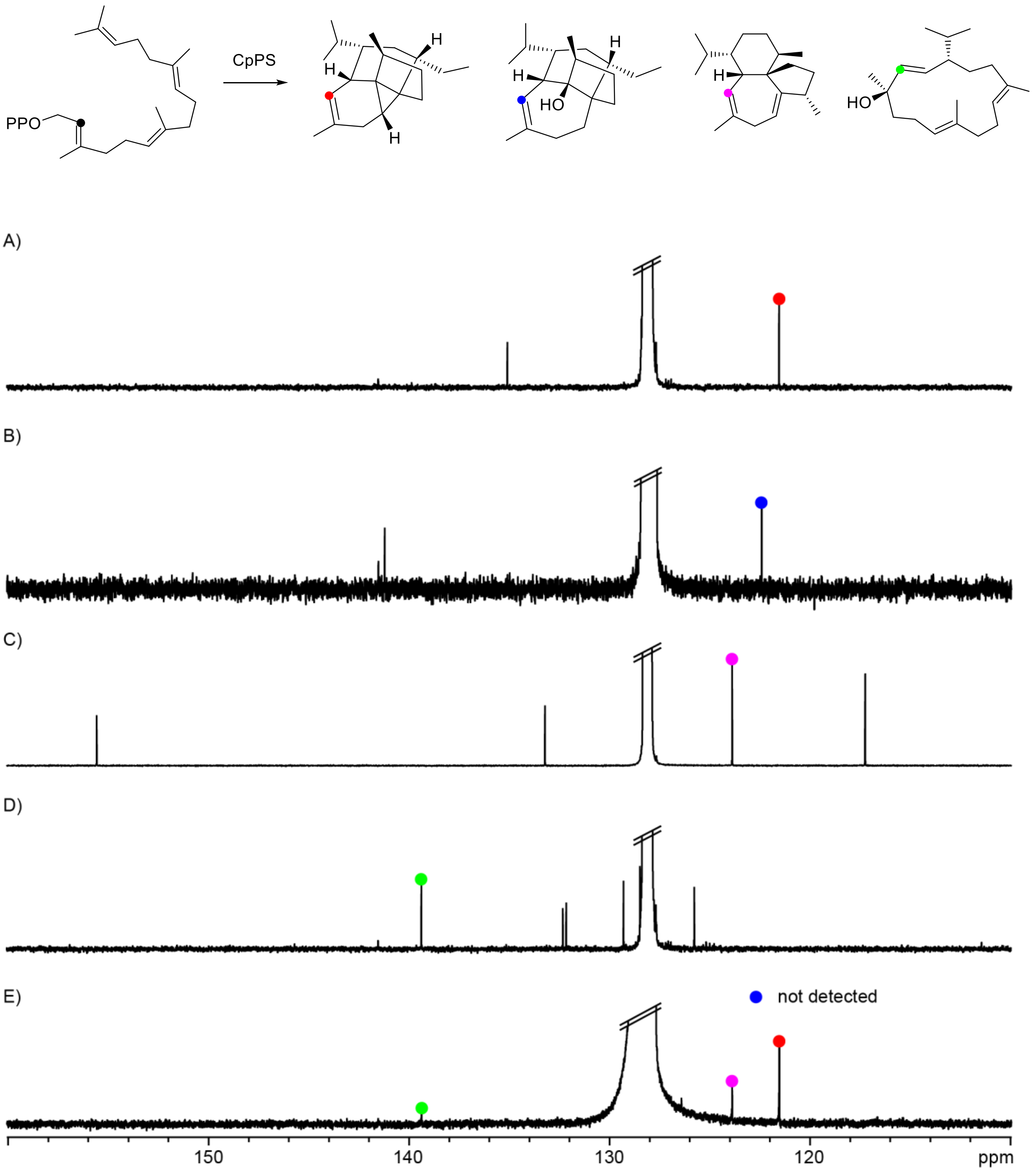

Figure S37. Enzymatic conversion of (2-^{13}C)GGPP with CpPS. ^{13}C-NMR spectra of A) unlabelled **1**, B) unlabelled **2**, C) unlabelled **3**, D) unlabelled **4** and E) the mixture of enzyme products obtained from (2-^{13}C)GGPP with CpPS. Coloured dots correlate the carbons of compounds **1** – **4** to the peaks observed in the ^{13}C-NMR spectra.

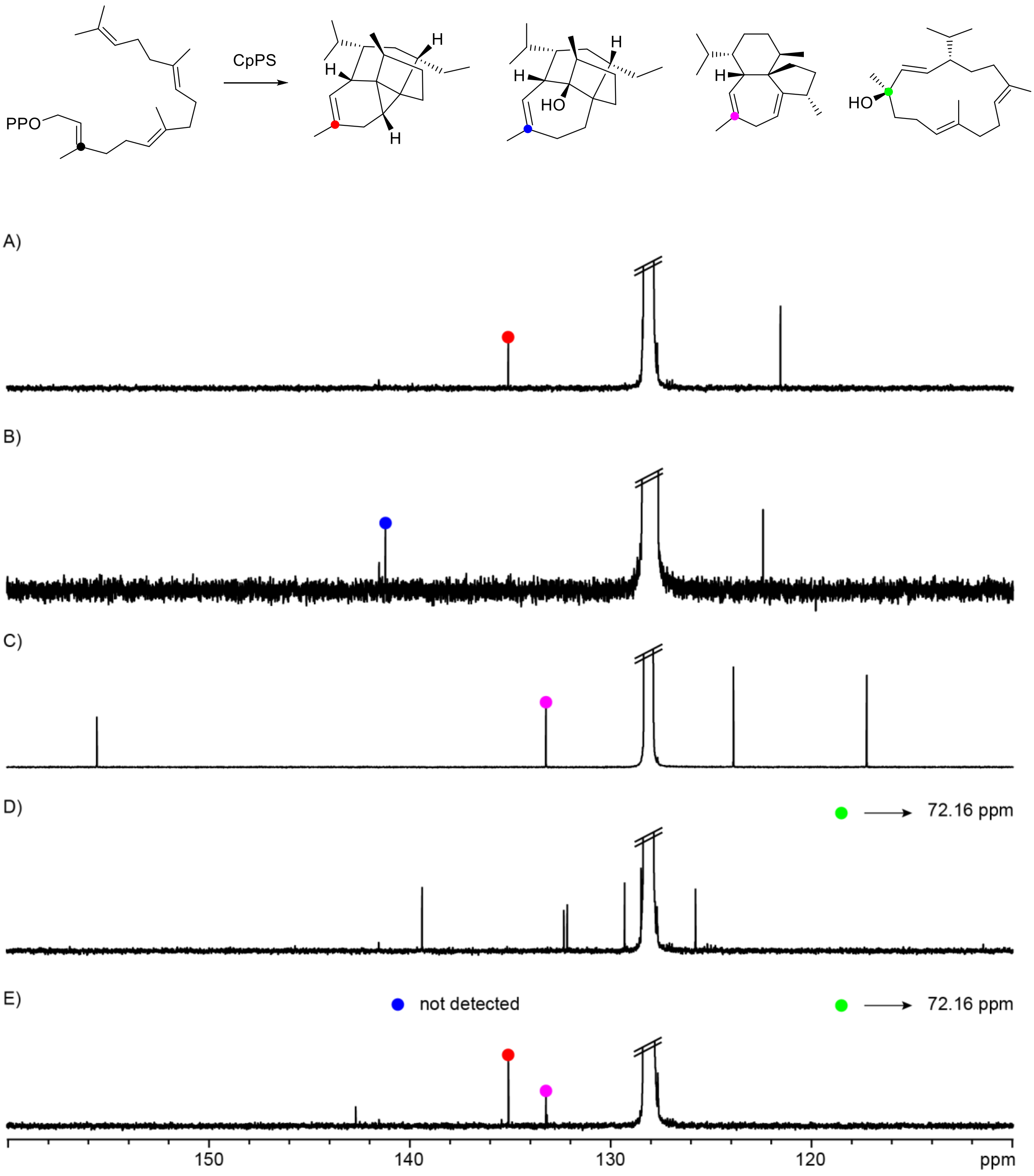

Figure S38. Enzymatic conversion of (3-^{13}C)GGPP with CpPS. ^{13}C-NMR spectra of A) unlabelled **1**, B) unlabelled **2**, C) unlabelled **3**, D) unlabelled **4** and E) the mixture of enzyme products obtained from (3-^{13}C)GGPP with CpPS. Coloured dots correlate the carbons of compounds **1** – **4** to the peaks observed in the ^{13}C-NMR spectra. In experiment E) an additional signal at 72.16 ppm for C3 of compound **4** is observed (not shown).

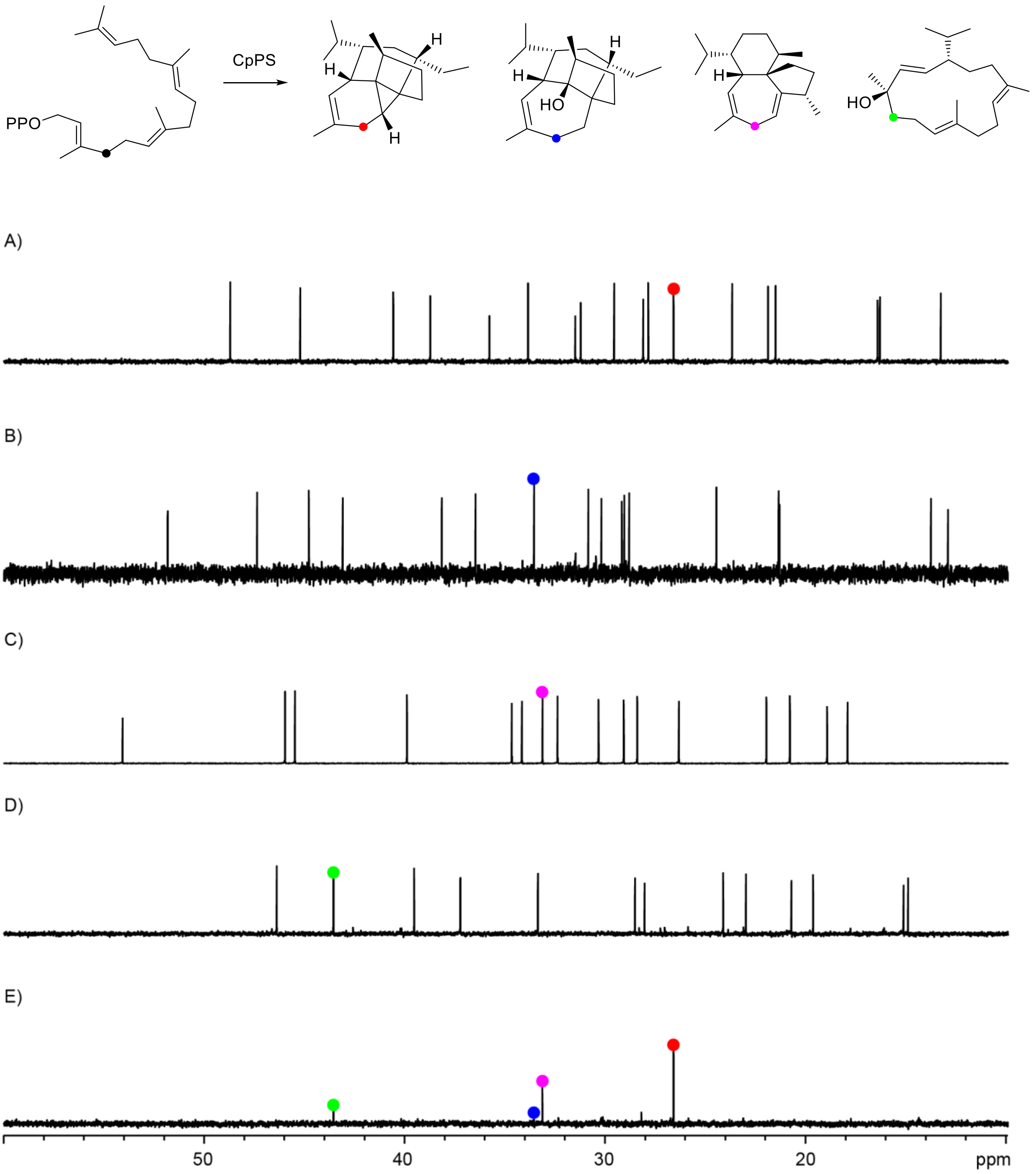

Figure S39. Enzymatic conversion of (4-^{13}C)GGPP with CpPS. ^{13}C-NMR spectra of A) unlabelled **1**, B) unlabelled **2**, C) unlabelled **3**, D) unlabelled **4** and E) the mixture of enzyme products obtained from (4-^{13}C)GGPP with CpPS. Coloured dots correlate the carbons of compounds **1** – **4** to the peaks observed in the ^{13}C-NMR spectra.

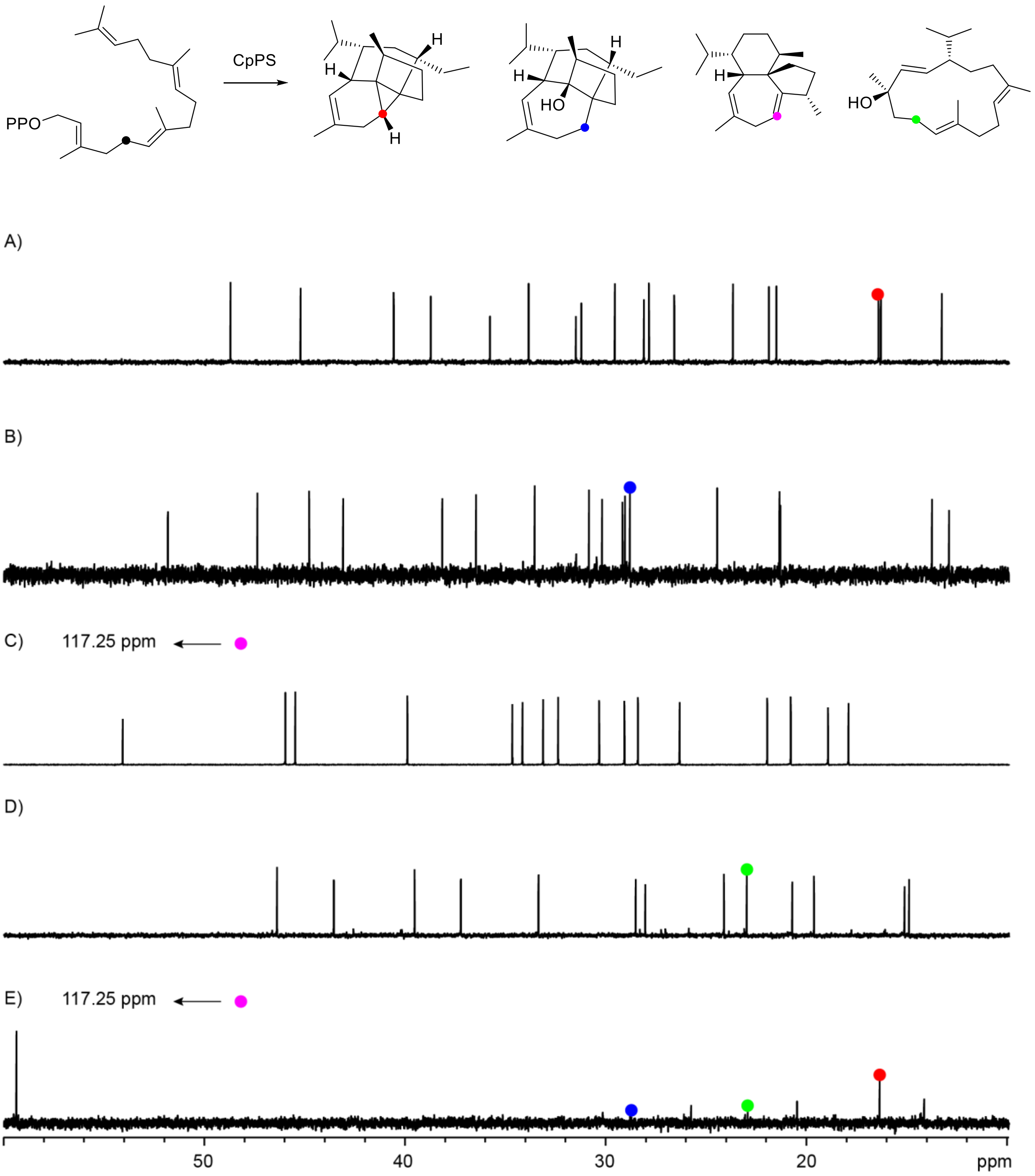

Figure S40. Enzymatic conversion of (5-^{13}C)GGPP with CpPS. ^{13}C-NMR spectra of A) unlabelled **1**, B) unlabelled **2**, C) unlabelled **3**, D) unlabelled **4** and E) the mixture of enzyme products obtained from (5-^{13}C)GGPP with CpPS. Coloured dots correlate the carbons of compounds **1** – **4** to the peaks observed in the ^{13}C-NMR spectra. In experiment E) an additional signal at 117.25 ppm for C5 of compound **3** is observed (not shown).

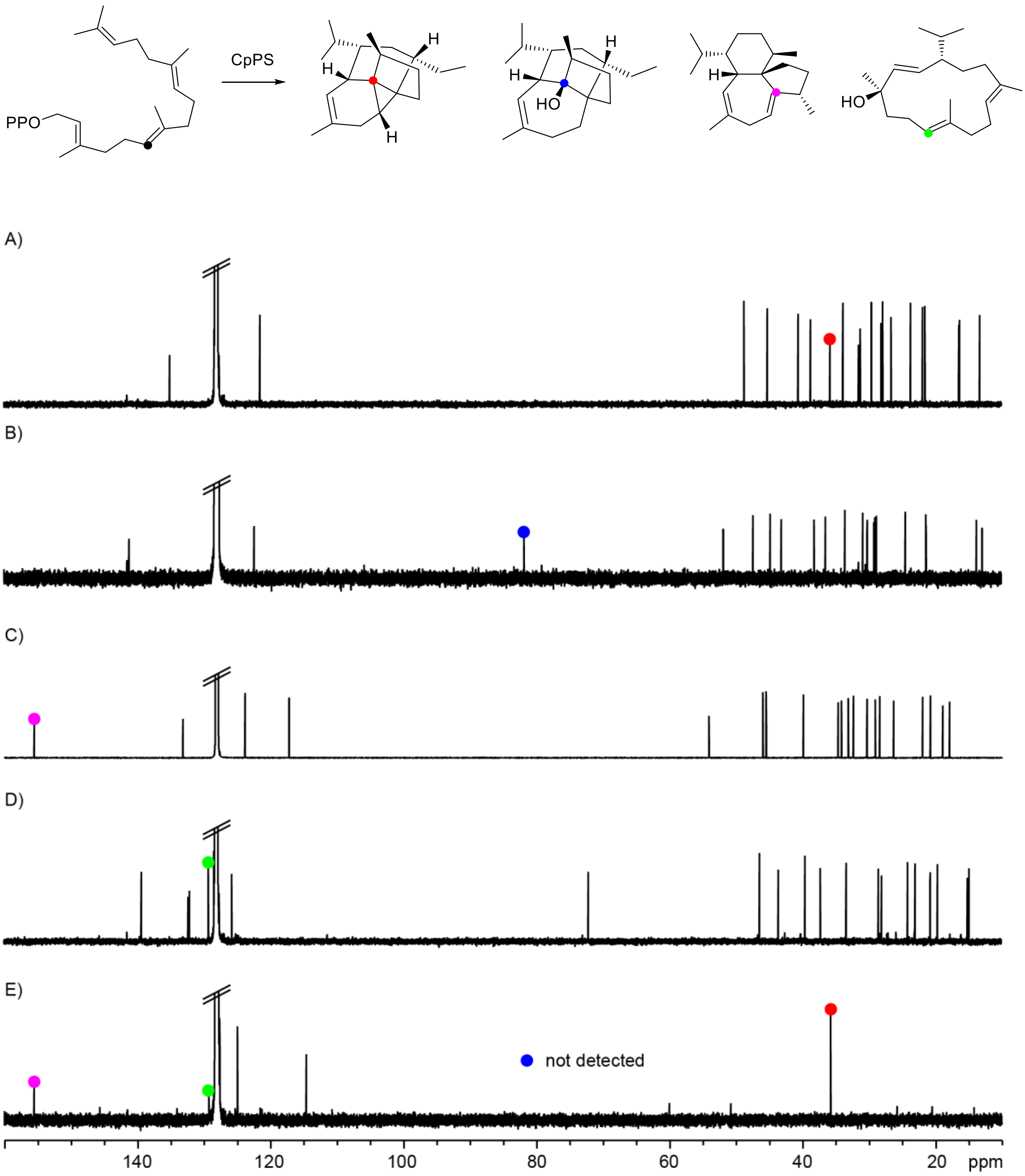

Figure S41. Enzymatic conversion of (6-^{13}C)GGPP with CpPS. ^{13}C-NMR spectra of A) unlabelled **1**, B) unlabelled **2**, C) unlabelled **3**, D) unlabelled **4** and E) the mixture of enzyme products obtained from (6-^{13}C)GGPP with CpPS. Coloured dots correlate the carbons of compounds **1** – **4** to the peaks observed in the ^{13}C-NMR spectra.

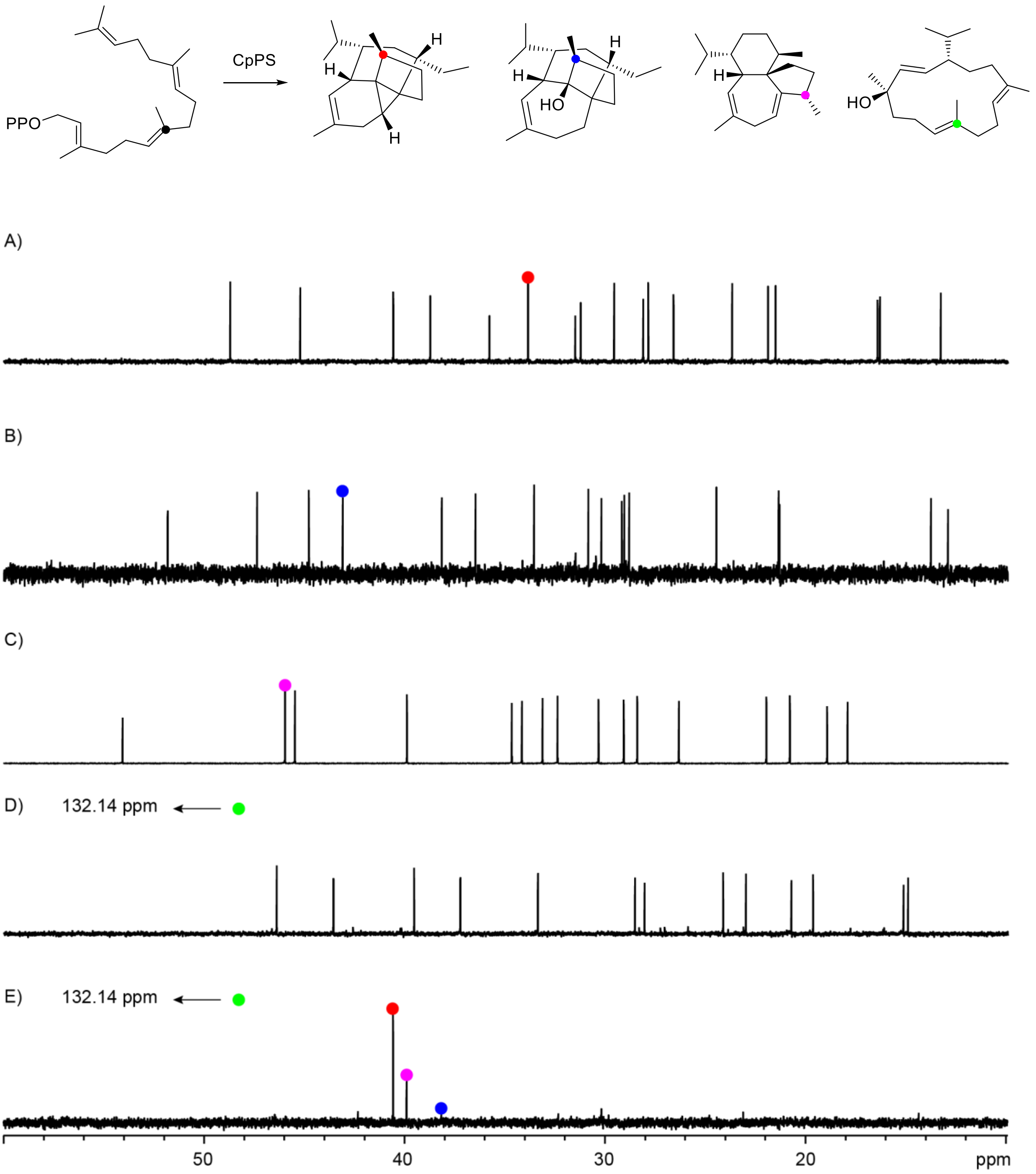

Figure S42. Enzymatic conversion of (7-^{13}C)GGPP with CpPS. ^{13}C-NMR spectra of A) unlabelled **1**, B) unlabelled **2**, C) unlabelled **3**, D) unlabelled **4** and E) the mixture of enzyme products obtained from (7-^{13}C)GGPP with CpPS. Coloured dots correlate the carbons of compounds **1** – **4** to the peaks observed in the ^{13}C-NMR spectra. In experiment E) an additional signal at 132.14 ppm for C7 of compound **4** is observed (not shown).

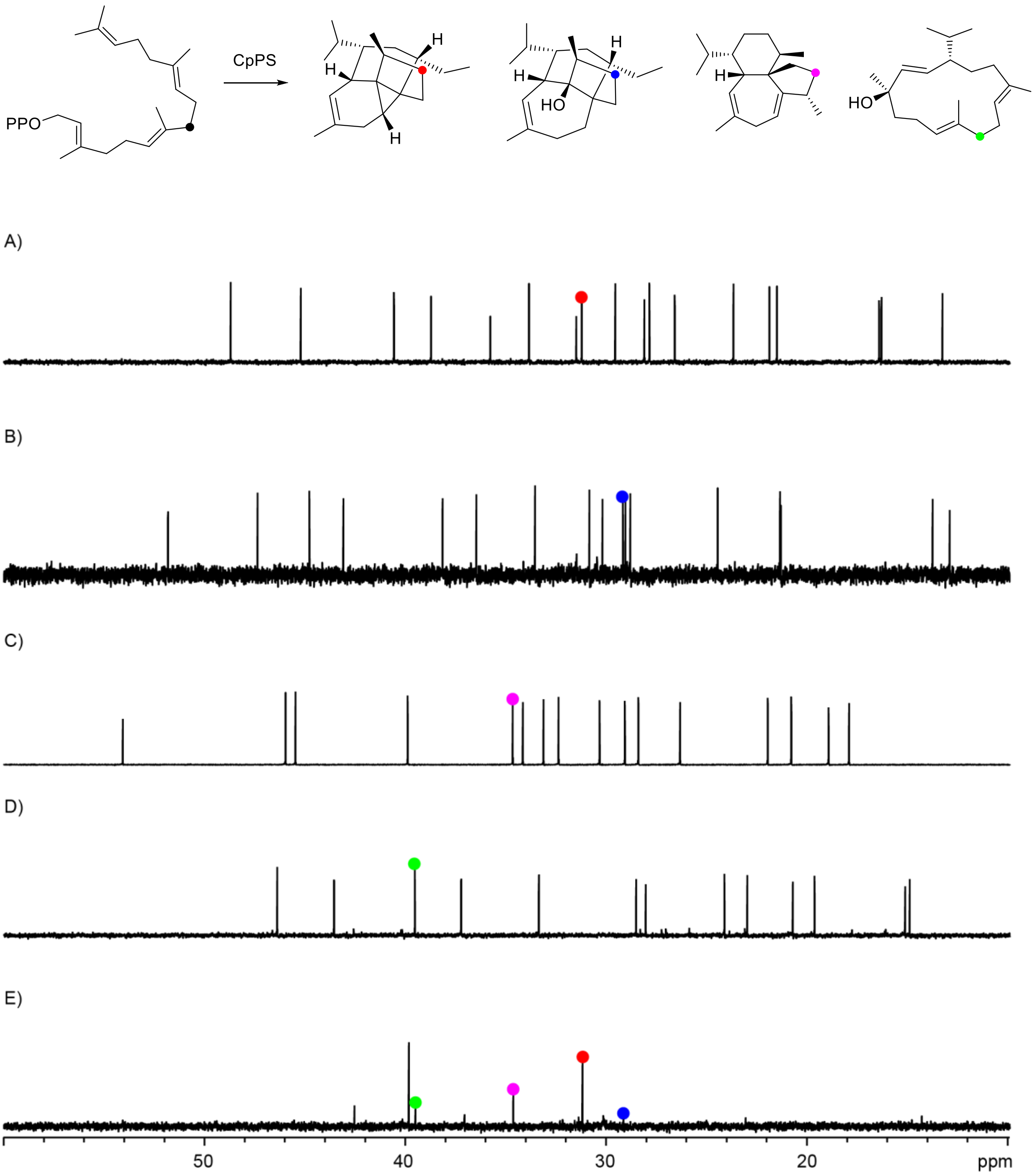

Figure S43. Enzymatic conversion of (8-^{13}C)GGPP with CpPS. ^{13}C-NMR spectra of A) unlabelled **1**, B) unlabelled **2**, C) unlabelled **3**, D) unlabelled **4** and E) the mixture of enzyme products obtained from (8-^{13}C)GGPP with CpPS. Coloured dots correlate the carbons of compounds **1** – **4** to the peaks observed in the ^{13}C-NMR spectra.

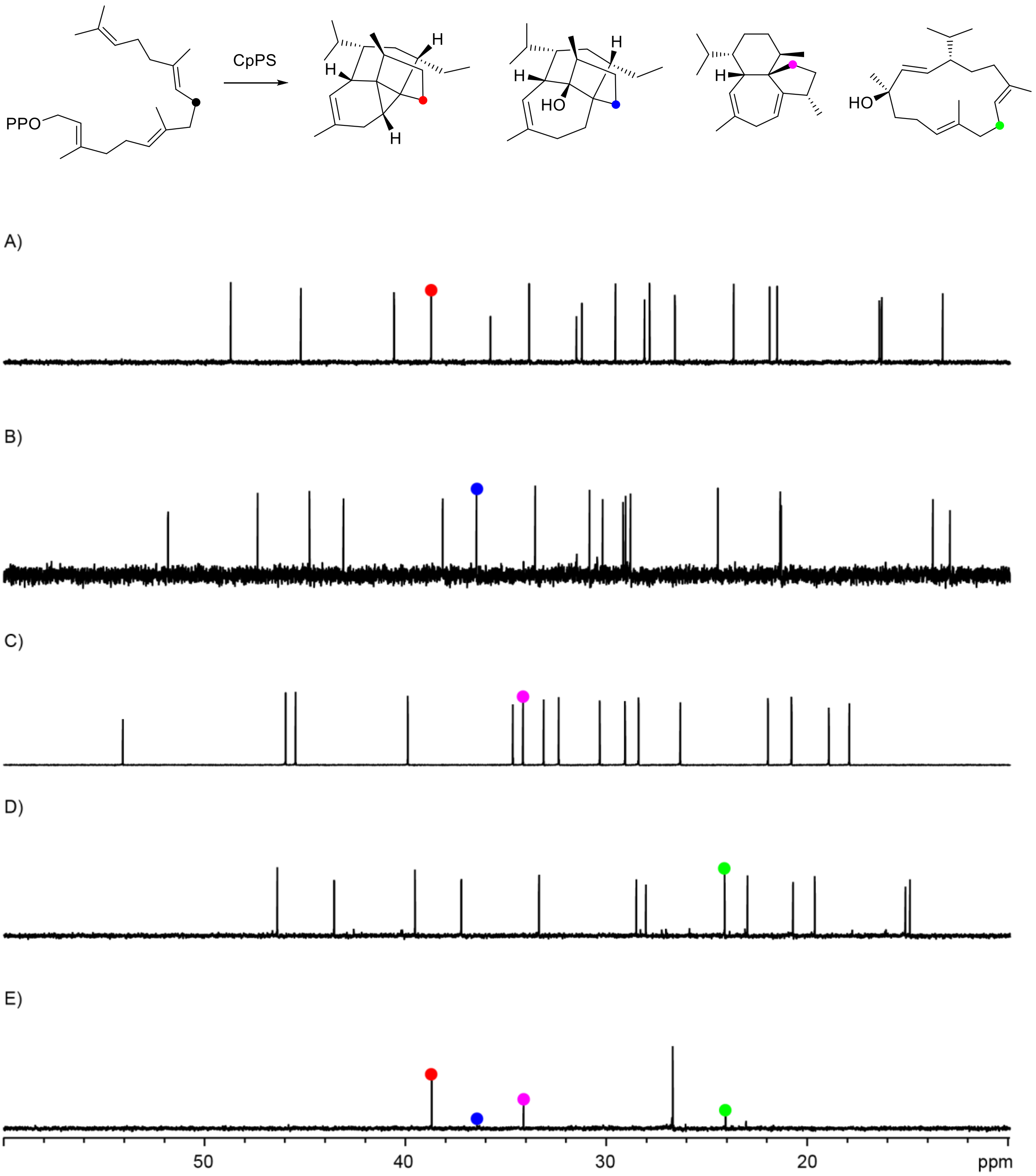

Figure S44. Enzymatic conversion of (9-^{13}C)GGPP with CpPS. ^{13}C-NMR spectra of A) unlabelled **1**, B) unlabelled **2**, C) unlabelled **3**, D) unlabelled **4** and E) the mixture of enzyme products obtained from (9-^{13}C)GGPP with CpPS. Coloured dots correlate the carbons of compounds **1** – **4** to the peaks observed in the ^{13}C-NMR spectra.

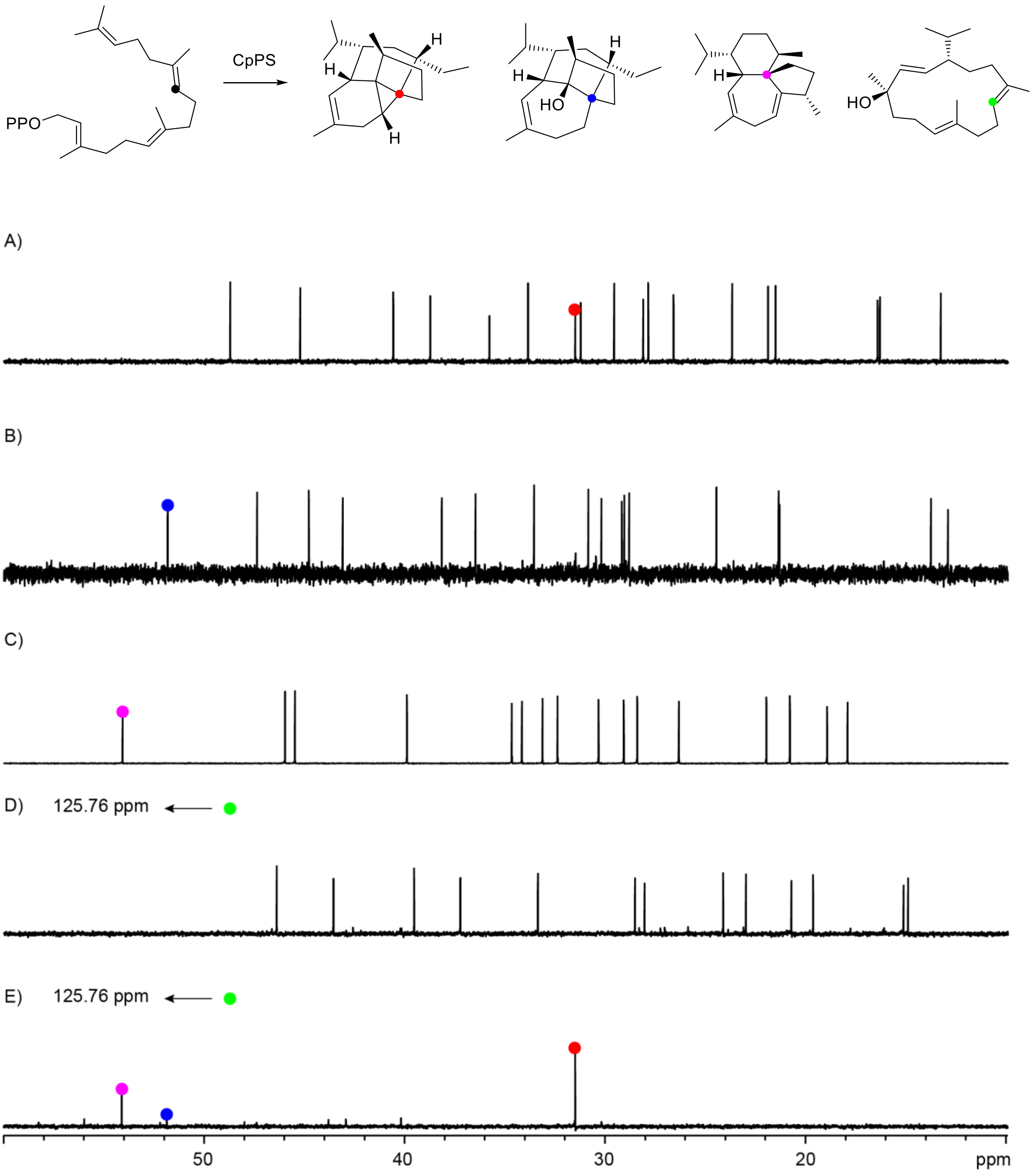

Figure S45. Enzymatic conversion of (10-^{13}C)GGPP with CpPS. ^{13}C-NMR spectra of A) unlabelled **1**, B) unlabelled **2**, C) unlabelled **3**, D) unlabelled **4** and E) the mixture of enzyme products obtained from (10-^{13}C)GGPP with CpPS. Coloured dots correlate the carbons of compounds **1** – **4** to the peaks observed in the ^{13}C-NMR spectra. In experiment E) an additional signal at 125.76 ppm for C10 of compound **4** is observed (not shown).

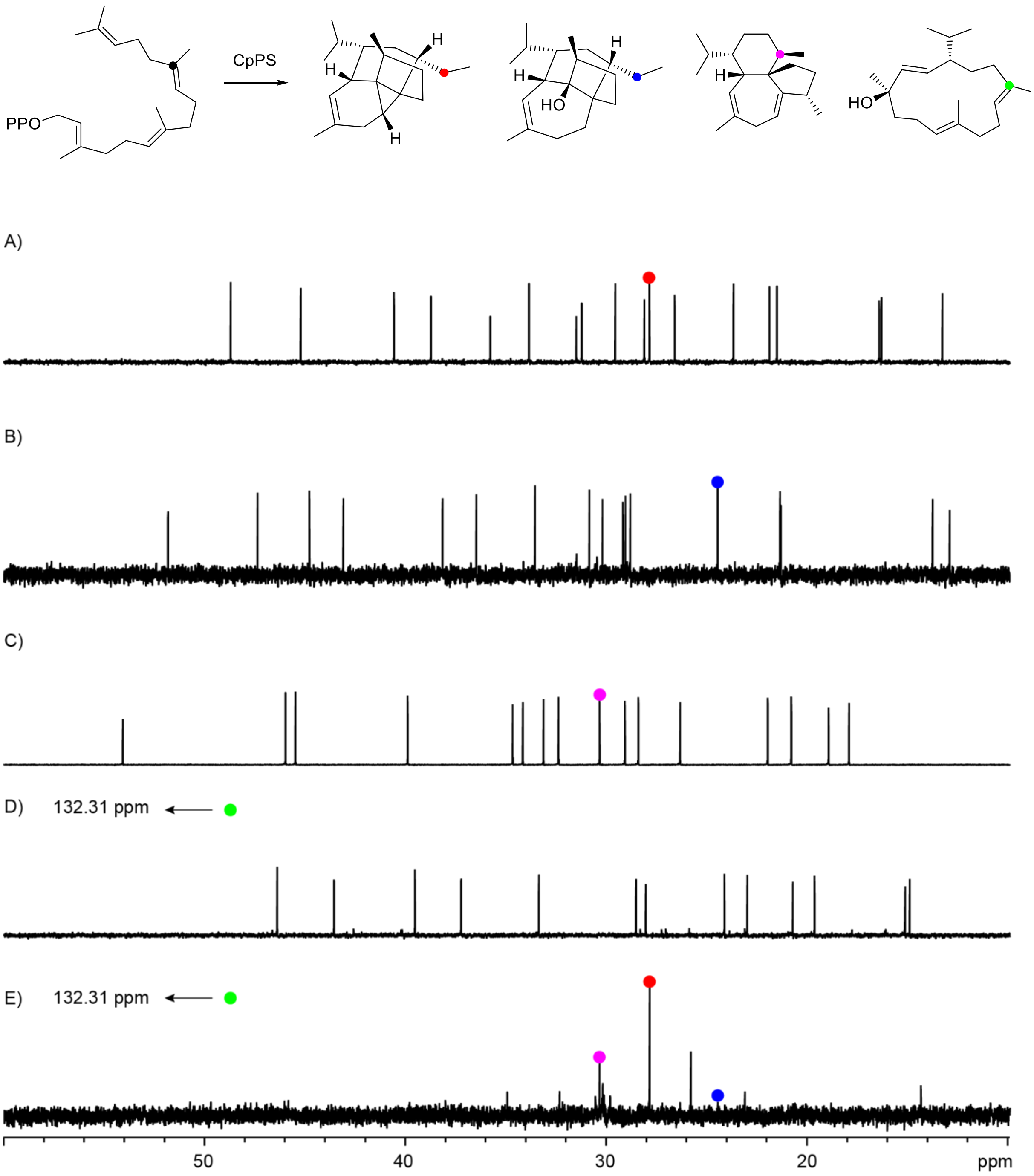

Figure S46. Enzymatic conversion of (11-^{13}C)GGPP with CpPS. ^{13}C-NMR spectra of A) unlabelled **1**, B) unlabelled **2**, C) unlabelled **3**, D) unlabelled **4** and E) the mixture of enzyme products obtained from (11-^{13}C)GGPP with CpPS. Coloured dots correlate the carbons of compounds **1** – **4** to the peaks observed in the ^{13}C-NMR spectra. In experiment E) an additional signal at 128.47 ppm for C1 of compound **4** is observed (not shown). In experiment E) an additional signal at 132.31 ppm for C11 of compound **4** is observed (not shown).

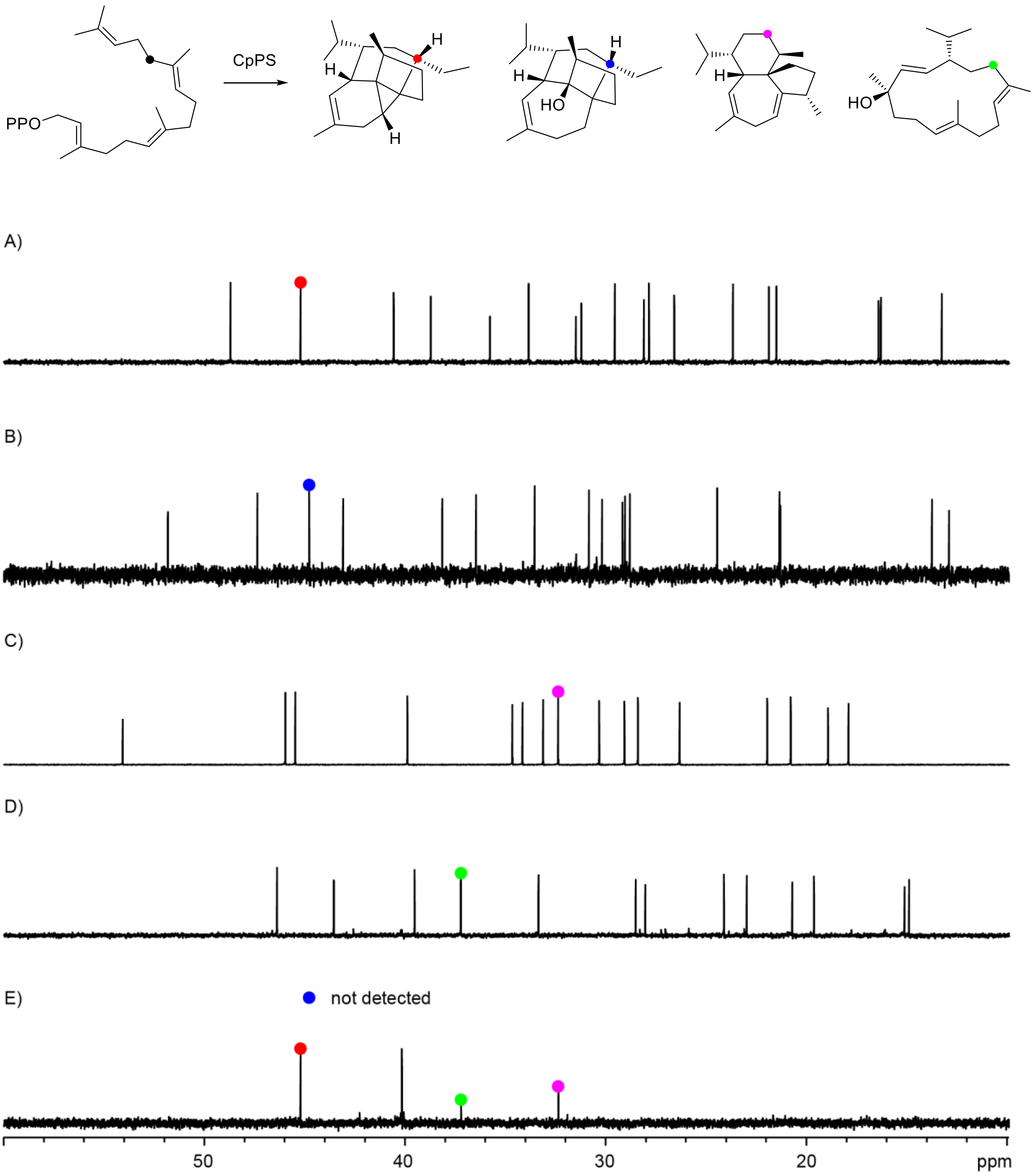

Figure S47. Enzymatic conversion of (12-^{13}C)GGPP with CpPS. ^{13}C-NMR spectra of A) unlabelled **1**, B) unlabelled **2**, C) unlabelled **3**, D) unlabelled **4** and E) the mixture of enzyme products obtained from (12-^{13}C)GGPP with CpPS. Coloured dots correlate the carbons of compounds **1** – **4** to the peaks observed in the ^{13}C-NMR spectra.

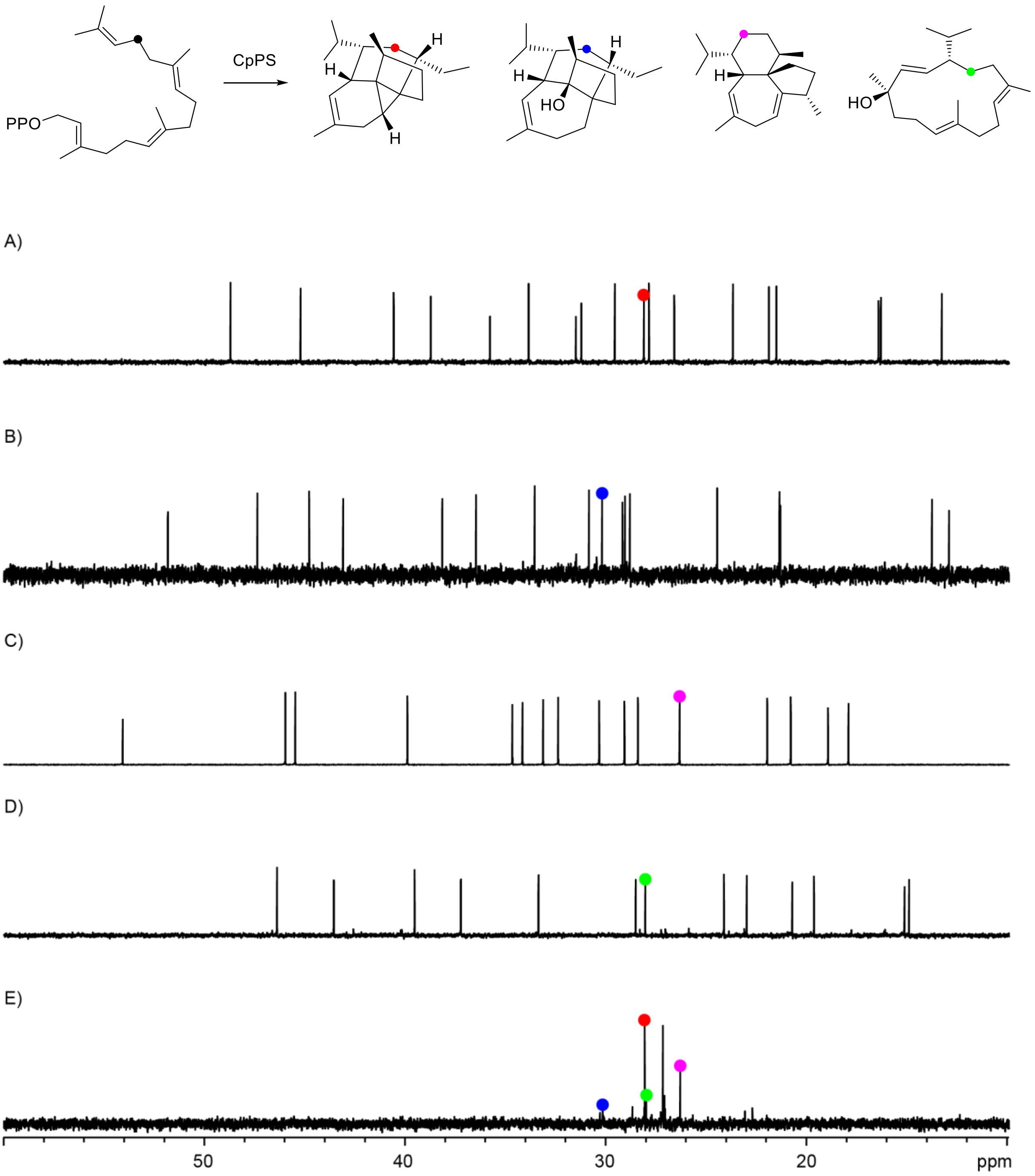

Figure S48. Enzymatic conversion of (13-^{13}C)GGPP with CpPS. ^{13}C-NMR spectra of A) unlabelled **1**, B) unlabelled **2**, C) unlabelled **3**, D) unlabelled **4** and E) the mixture of enzyme products obtained from (13-^{13}C)GGPP with CpPS. Coloured dots correlate the carbons of compounds **1** – **4** to the peaks observed in the ^{13}C-NMR spectra.

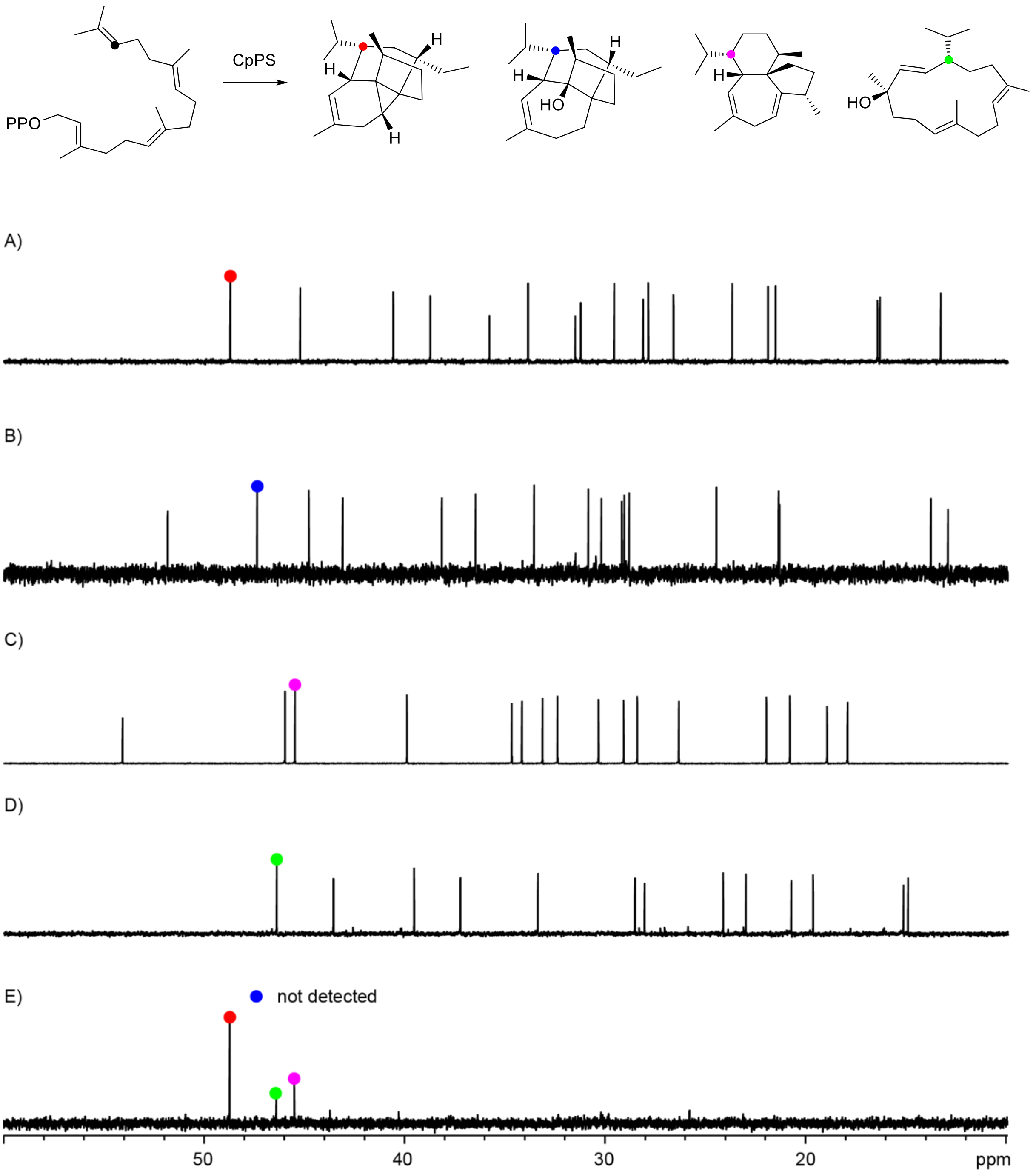

Figure S49. Enzymatic conversion of (14-^{13}C)GGPP with CpPS. ^{13}C-NMR spectra of A) unlabelled **1**, B) unlabelled **2**, C) unlabelled **3**, D) unlabelled **4** and E) the mixture of enzyme products obtained from (14-^{13}C)GGPP with CpPS. Coloured dots correlate the carbons of compounds **1** – **4** to the peaks observed in the ^{13}C-NMR spectra.

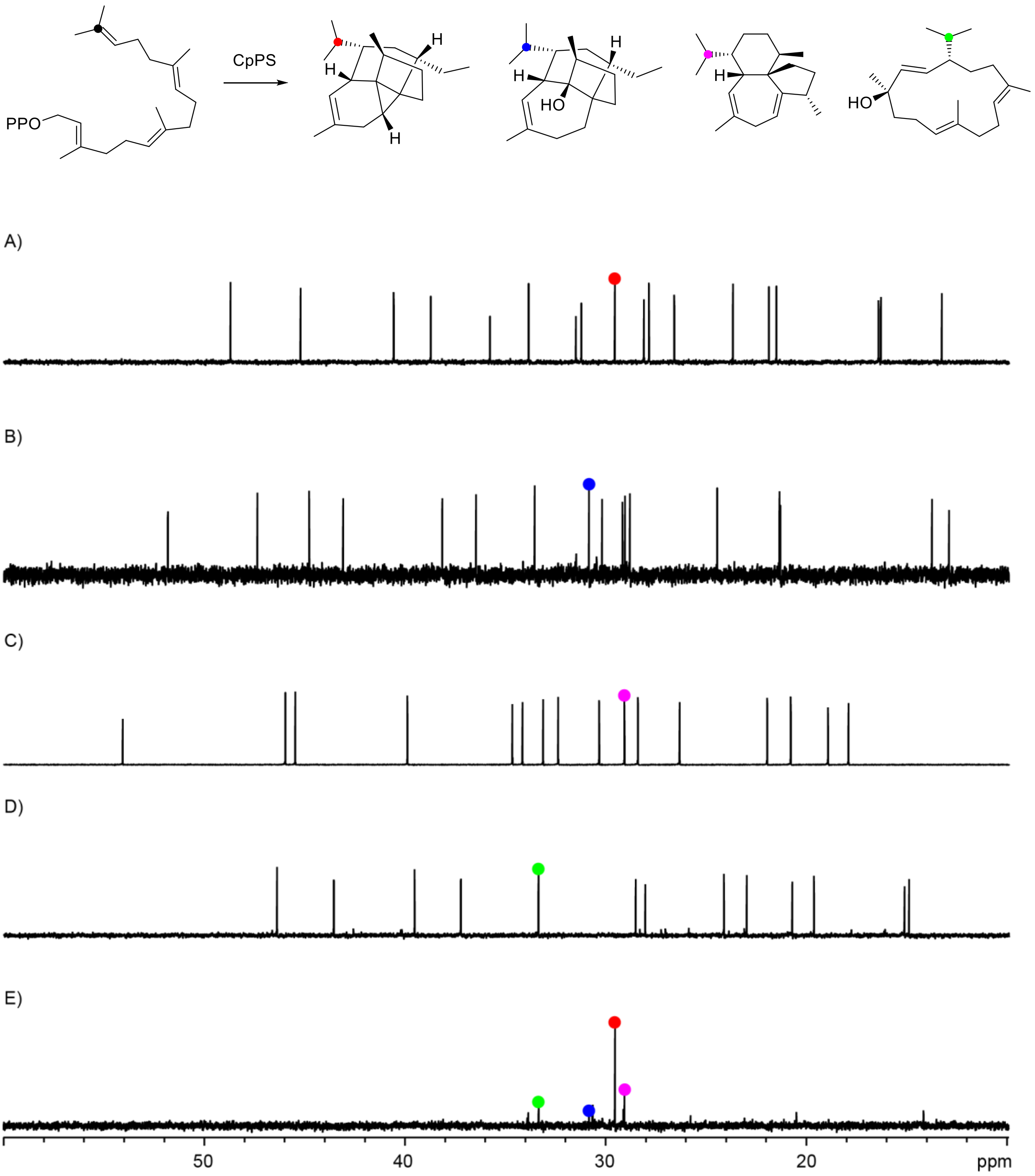

Figure S50. Enzymatic conversion of (15-^{13}C)GGPP with CpPS. ^{13}C-NMR spectra of A) unlabelled **1**, B) unlabelled **2**, C) unlabelled **3**, D) unlabelled **4** and E) the mixture of enzyme products obtained from (15-^{13}C)GGPP with CpPS. Coloured dots correlate the carbons of compounds **1** – **4** to the peaks observed in the ^{13}C-NMR spectra.

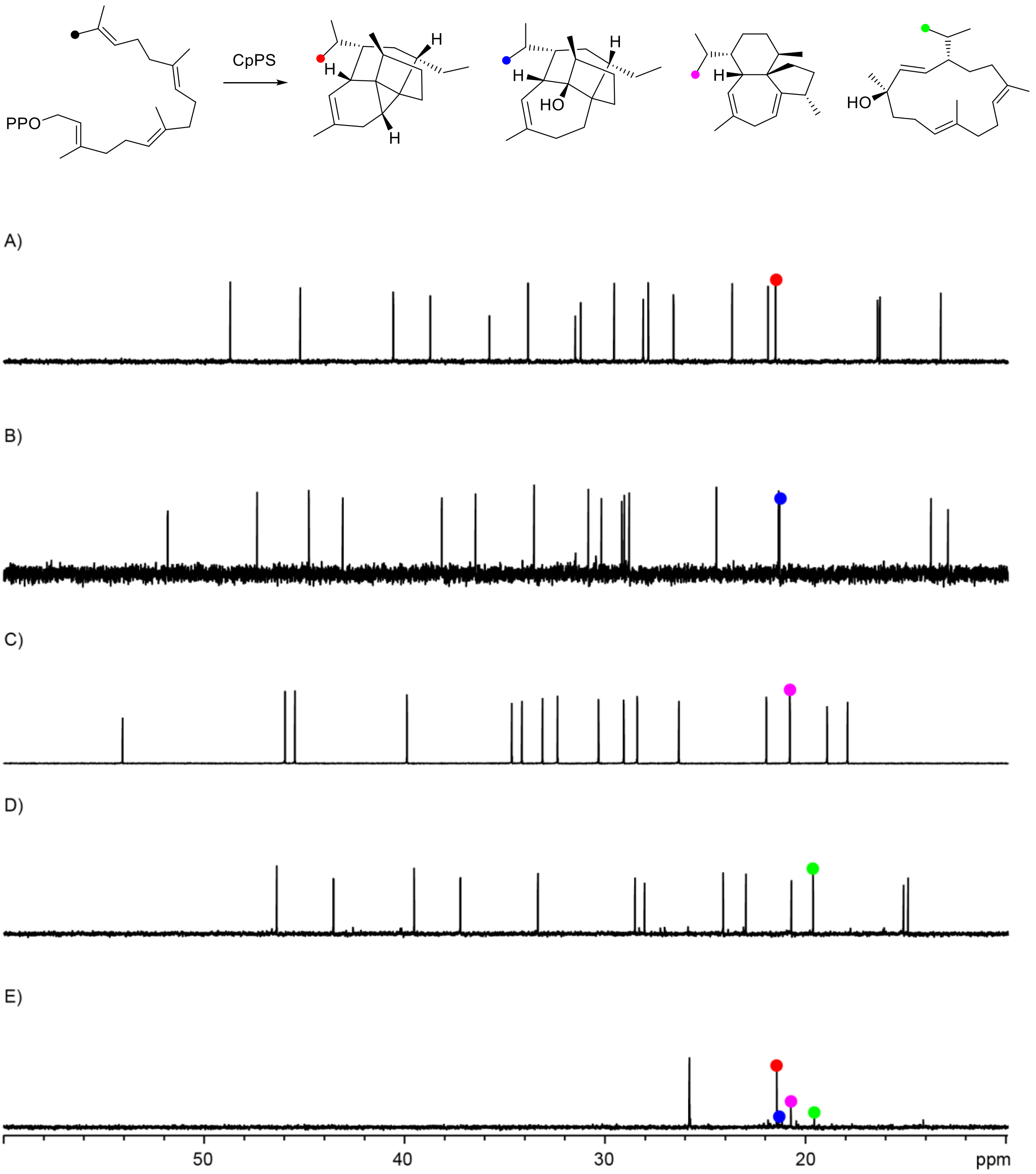

Figure S51. Enzymatic conversion of (16-^{13}C)GGPP with CpPS. ^{13}C-NMR spectra of A) unlabelled **1**, B) unlabelled **2**, C) unlabelled **3**, D) unlabelled **4** and E) the mixture of enzyme products obtained from (16-^{13}C)GGPP with CpPS. Coloured dots correlate the carbons of compounds **1** – **4** to the peaks observed in the ^{13}C-NMR spectra.

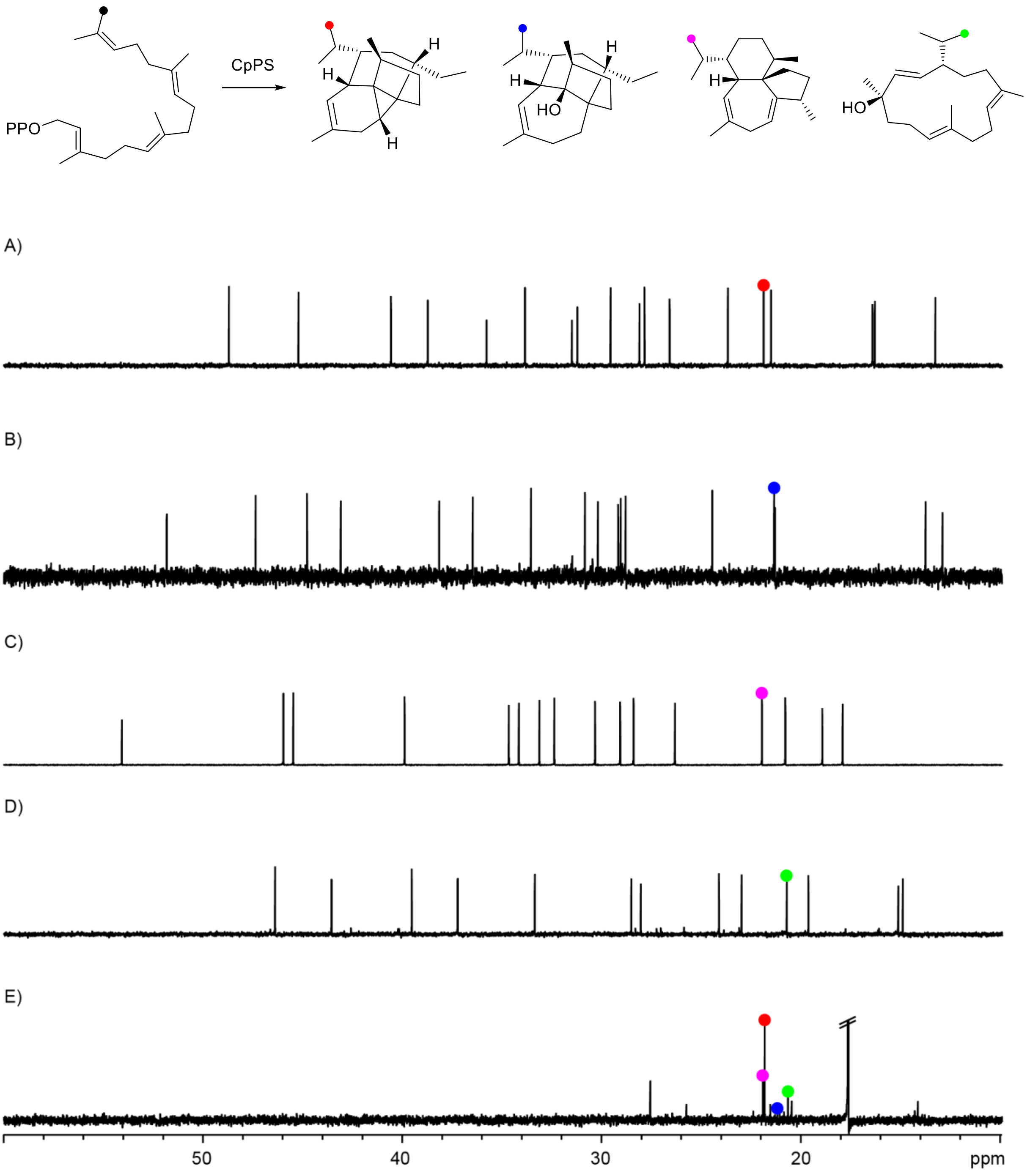

Figure S52. Enzymatic conversion of (17-^{13}C)GGPP with CpPS. ^{13}C-NMR spectra of A) unlabelled **1**, B) unlabelled **2**, C) unlabelled **3**, D) unlabelled **4** and E) the mixture of enzyme products obtained from (17-^{13}C)GGPP with CpPS. Coloured dots correlate the carbons of compounds **1** – **4** to the peaks observed in the ^{13}C-NMR spectra.

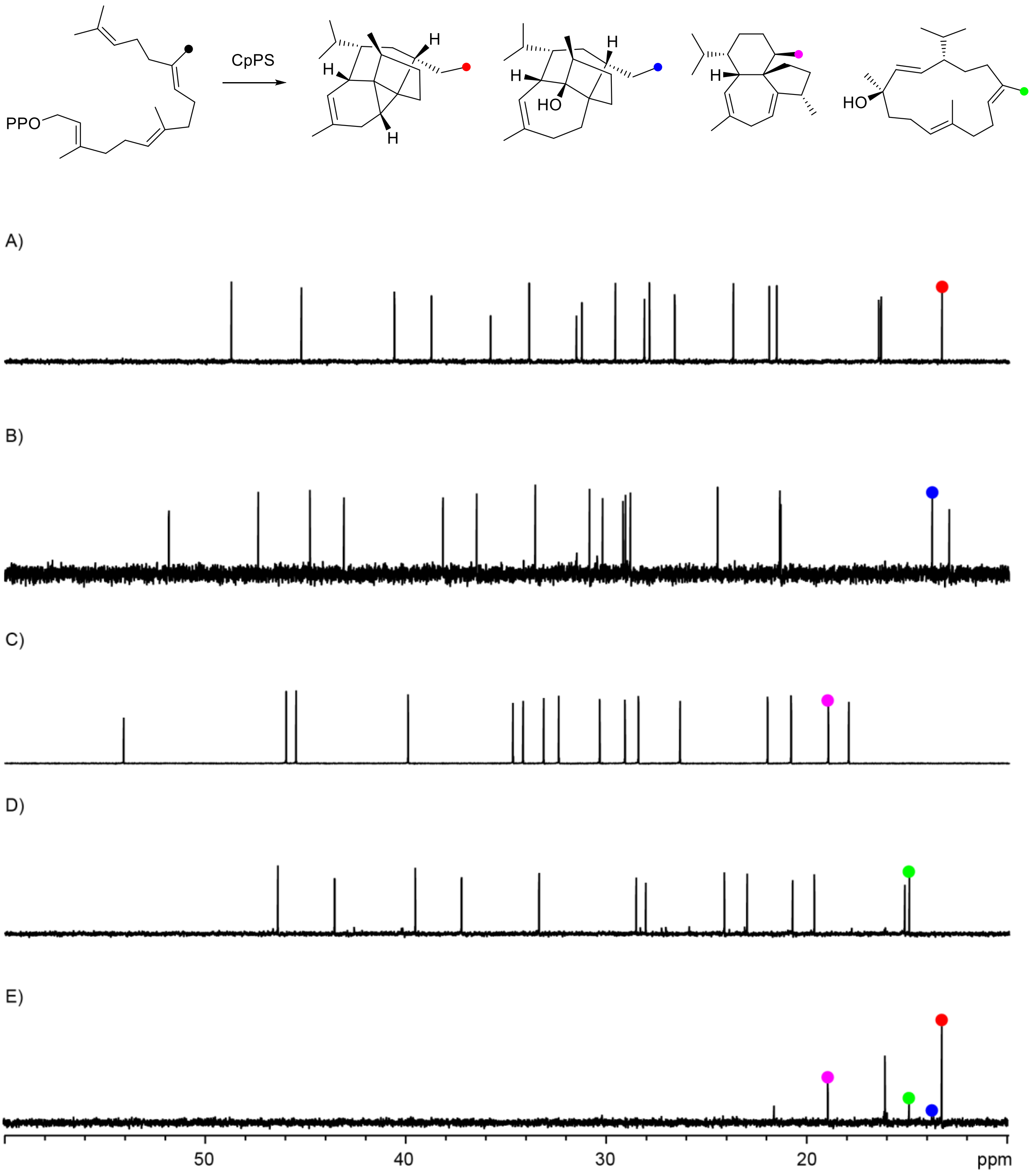

Figure S53. Enzymatic conversion of (18-^{13}C)GGPP with CpPS. ^{13}C-NMR spectra of A) unlabelled **1**, B) unlabelled **2**, C) unlabelled **3**, D) unlabelled **4** and E) the mixture of enzyme products obtained from (18-^{13}C)GGPP with CpPS. Coloured dots correlate the carbons of compounds **1** – **4** to the peaks observed in the ^{13}C-NMR spectra.

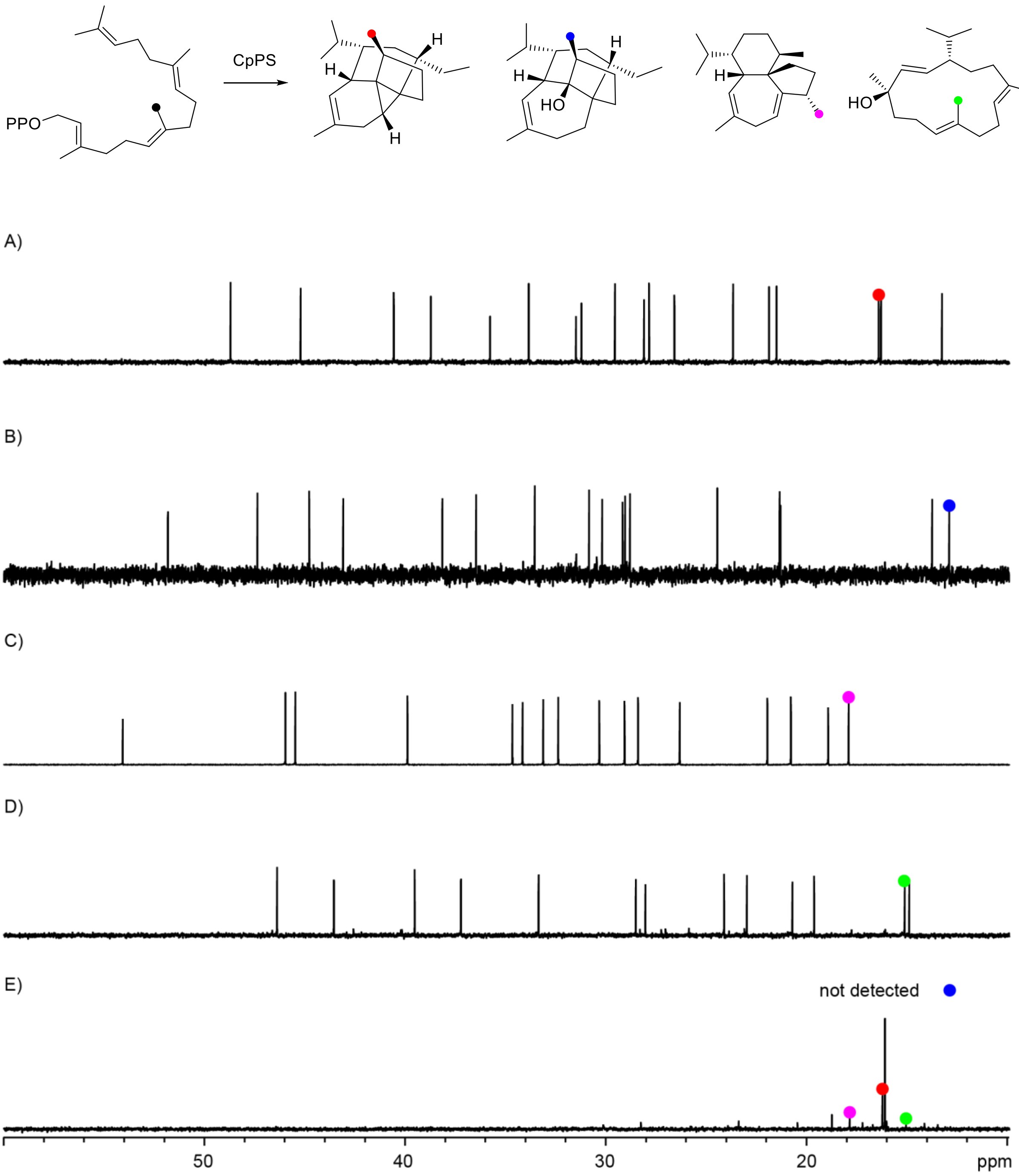

Figure S54. Enzymatic conversion of (19-^{13}C)GGPP with CpPS. ^{13}C-NMR spectra of A) unlabelled **1**, B) unlabelled **2**, C) unlabelled **3**, D) unlabelled **4** and E) the mixture of enzyme products obtained from (19-^{13}C)GGPP with CpPS. Coloured dots correlate the carbons of compounds **1** – **4** to the peaks observed in the ^{13}C-NMR spectra.

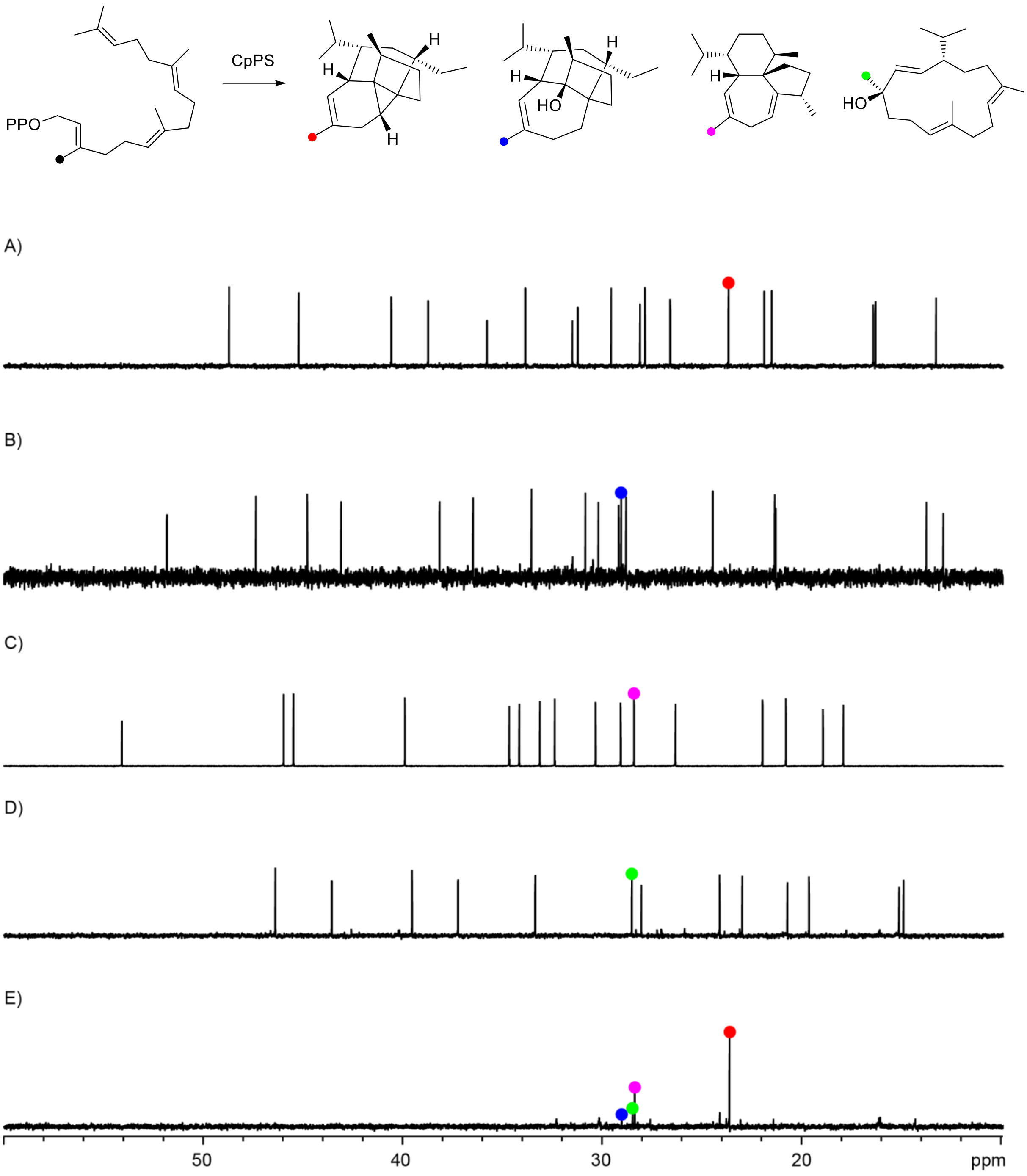

Figure S55. Enzymatic conversion of (20-^{13}C)GGPP with CpPS. ^{13}C-NMR spectra of A) unlabelled **1**, B) unlabelled **2**, C) unlabelled **3**, D) unlabelled **4** and E) the mixture of enzyme products obtained from (20-^{13}C)GGPP with CpPS. Coloured dots correlate the carbons of compounds **1** – **4** to the peaks observed in the ^{13}C-NMR spectra.

```
Consensus                                          ----------------------MXMPQDPDFXLPFPSXXHPXLAXARXXXAXAWVRXXGLV   39
Selina-4(15),7(11)-diene (S. pristinaespiralis)    ---------------------MEPELTVPPLFSPIRQAIHPKHADIDVQTAAWAETFRI-   39
Polytrichastrene A (C. polytrichastri)             -----------MKTSISNEEFYAGLQQLPKPKYPFPDSMHPDFQQQREEYYDWIDREYIF   50
Spiroviolene (S. violens)                          --------------------MTVNEIDLPPIFCPLESARHPRAHLVDERAREWIRTSPMC   41
a-Amorphene (S. viridochromogenes)                 --MSTTHEEIALAGPDGIPAVDLRDLIDAQLYMPFPFERNPHASEAAAGVDHWLSTWGLT   59
Germacrene A (M. marina)                           ---------------------MRDFALSALHEPPFPSRCHPAVDLAAVQSDAWVRAFGLA   40
Caryolan-1-ol (S. griseus subsp. griseus)          ---------------------MSQITLPAFHMPFQSAGCHPGLAETREAAWEWAAAEGLD   40
epi-Cubenol (S. griseus subsp. griseus)            -----------------------------------------MAHTRGHLDSWTRRTGLV   19
7-epi-a-Eudesmol (S. viridochromogenes)            ------------------------MPQDVRFDLPFETPVSKHLESARARHLRWVWEMRLV   37
Avermitilol (S. avermitilis)                       ------------------------MPQDIDFGLPAPAGISPGLEATRRHNLGWVRRLGLV   37
Pentalenene (S. exfoliatus)                        ------------------------MPQDVDFHIPLPGRQSPDHARAEAEQLAWPRSLGLI   37

Consensus                                          XSXEAREXXAXXDXADLAARXYPHAXX-AXLDLLADWIAWLFLFDDQFDD-GPLGRRPXR   96
Selina-4(15),7(11)-diene (S. pristinaespiralis)    GSEELRGKLVTQDIGTFSARILPEGRE-EVVSLLADFILWLFGVDDGHCEEGELGHRPGD   97
Polytrichastrene A (C. polytrichastri)             HSKEAREKHKKHNLTDIASRGCPSLKTFAELRPLASYAANGAMMDDYWDTCTHSE-----  104
Spiroviolene (S. violens)                          TTDEERTWVAASCSTDFFARFAPDAATDDRLLWTSLWVYWGFAFDDHRCDNGPFSNRPAA  100
a-Amorphene (S. viridochromogenes)                 DDPAVAAMISCTRPAELAAFNGPDMDS-GLLQIAANQIAYQFVFDDRAEDIGRHSPGR--  115
Germacrene A (M. marina)                           GDQAGHRRLAGARAAELAARACPEASP-SGLRLLTDLINWLFVVDDACDDDGLGAAPTL-   97
Caryolan-1-ol (S. griseus subsp. griseus)          LSVPARRKMIRTRPELWISLIFPQATQ-AHLDLFCQWLFWAFLVDDEFDDGPAGRDPLM-   97
epi-Cubenol (S. griseus subsp. griseus)            HRESARNRFEQADFGAFVGMVYPTADE-EHLDLVADWFVWLFLVDDQLDDGHLGRSPDR-   76
7-epi-a-Eudesmol (S. viridochromogenes)            HSREGFEEYRSWDLPQAAARTYPHASA-DDMVVLMNWFSLAFLFDDQFDASRPDRADR--   93
Avermitilol (S. avermitilis)                       GDGPSLAWYTSWDMPRLAACGFPHARG-AALDLCADAMAFFFVFDDQFDGPLGRDPAR--   93
Pentalenene (S. exfoliatus)                        RSDAAAERHLRGGYADLASRFYPHATG-ADLDLGVDLMSWFFLFDDLFDGPRGENPED--   93

Consensus                                          LAXXLXRLXAVLDGP-G-P----XXXAXPXAAALADLWRRTXXGMTPAWVARFAXXLREY  150
Selina-4(15),7(11)-diene (S. pristinaespiralis)    LAGLLHRLIRVAQNP-EAP----MMQDDPLAAGLRDLRMRVDRFGTAGQTARWVDALREY  152
Polytrichastrene A (C. polytrichastri)             MMEISKRIMLLLTGE-DTN---EPTDNGIFHQFWVLRQDCLTCEMPERLYKKFIKSLNEV  160
Spiroviolene (S. violens)                          FSALAGRVQRALEAP-SAR-----DESDGFIPALQEIAAQFRSFGTPLQVRRFAAAHRAW  154
a-Amorphene (S. viridochromogenes)                 LLPMLSESVAILRDG-QPP-------TTPLGAALADLHRQVQERCTPAQAARWAWNSREY  167
Germacrene A (M. marina)                           LAPTLAGLLAVLDRH-GDPEVPVPPDGGPVAVALHDLIRRARDRNRPAHLLRLVSQLREY  156
Caryolan-1-ol (S. griseus subsp. griseus)          CERAIARLVDVFDGA-A-P-------NGPMERALAGLRDRTCRGRSPQWNRQFRRDTAAW  148
epi-Cubenol (S. griseus subsp. griseus)            VRDVVDRMRAVVDGS-A-PEVLPDEDAPAAVTALVDLWKRTMPEAAPHWRTRFAWHLVTY  134
7-epi-a-Eudesmol (S. viridochromogenes)            IAEVARELIVTPLRPAGTP----PRVACPITLAWTEVWKHLSHGMSLTWQSRFAASWGRF  149
Avermitilol (S. avermitilis)                       AARVCRRLTGIVHGA-G-P----GPGADACSAAFADVWARSTDGAHPGWVARTAHEWEYY  147
Pentalenene (S. exfoliatus)                        TKQLTDQVAAALDGP-L-P----DT-APPIAHGFADIWRRTCEGMTPAWCARSARHWRNY  146
```

```
Consensus                                       L-XXYXWEAVNRAXGTVP--XLAEYLAMRRHTXGVQPFLDLXERAAGI-EXPAXXXXHPA  206
Selina-4(15),7(11)-diene (S. pristinaespiralis) F-FSVVWEAAHRRAGTVP--DLNDYTLMRLYDGATSVVLPMLEMGHGY-ELQPYERDRTA  208
Polytrichastrene A (C. polytrichastri)          L-IGYADERVYYRTNTIP--PLAVYLLIREATSGAQPFCKYVAMQKEYRQLPDDVLEHSH  217
Spiroviolene (S. violens)                       L-SGVTWQIGNAAAGRMP--GLDEYVAMRLLSAGGEPPFAMLELATGL-EVPAQDLERPA  210
a-Amorphene (S. viridochromogenes)              V-HGLLYEAVAQAHPAPV--ESGLCRSIRSLIAGVEPFYPLCEAAQRC-ELAPEELHHPA  223
Germacrene A (M. marina)                        L-LALLWEAANRERRRVP--GVAEYVQMRRHTGGVRPSFTVTDLARPS-APRAEQRVASA  212
Caryolan-1-ol (S. griseus subsp. griseus)       L-WTYYAEAVERAAGQVP--SRAEFAKHRRDSVAMQPFLCLHEITAGI-DLPDSARSLPA  204
epi-Cubenol (S. griseus subsp. griseus)         LTTATTWEAGNRAEDVVP--SEETYIAKRRHTGAIHVCMDLIEIVAGI-DAPESVHNDPR  191
7-epi-a-Eudesmol (S. viridochromogenes)         L-EAHCEEVDLAARGLEGTLGLVEFTEFRRRTVGIHHSIDAGERSRGF-EVPAQAMAHPV  207
Avermitilol (S. avermitilis)                    F-AAQAHEAINRLRGTPG--DMESYLQVRRGIAGTDLPLSLGERAAGI-TVPAAAFHSPQ  203
Pentalenene (S. exfoliatus)                     F-DGYVDEAESRFWNAPCD-SAAQYLAMRRHTIGVQPTVDLAERAGRF-EVPHRVFDSAV  203

Consensus                                       MRALRELAADVIGWCNDIFSLEKEX-RRGXMHNLVXVLRRERGXSLQEAVDAAARXXXRR  265
Selina-4(15),7(11)-diene (S. pristinaespiralis) VRAVAEMASFIITWDNDIFSYHKERRGSGYYLNALRVLEQERGLTPAQALDAAISQRDRV  268
Polytrichastrene A (C. polytrichastri)          IQRLHTLCAMMIGIHNDIISLPKELHREGDTMNLVKVLQQEHKTSINEAYMMALELHDNY  277
Spiroviolene (S. violens)                       VRALTEMAIMVAALDNDRHSLRKELARGQTDQNVYSVLMQETGLPLQEAVAAATRLRDRV  270
a-Amorphene (S. viridochromogenes)              MRRLSRLSADAAVWIPDLFSAVKEQ-RAGGMINLALAYRRTHRCSLPAAVTLAVRHINST  282
Germacrene A (M. marina)                        LTVLDALATDLVCWCNDLFSYGKERGTVPEAHNLITTIAGETGQDESAALHAAANRFNKA  272
Caryolan-1-ol (S. griseus subsp. griseus)       YIALRNAVTDHSGLCNDICSFEKEA-ALGYEHNAVRLIQRDRGSTLQEAVDEAGIQLARI  263
epi-Cubenol (S. griseus subsp. griseus)         FITALEAACNHVCWANDVYSFEKEQ-VLGEIHNLVHLVRHHRGLGEQQALDHVAERLAME  250
7-epi-a-Eudesmol (S. viridochromogenes)         MERMRDLAADTIGFMNDIHSFEREK-RRGDGHNLIAVLRRERGCSWQEATDEAYRMTIAR  266
Avermitilol (S. avermitilis)                    LRIMREAAIDVTLMCNDVYSLEKEE-ARGDMDNLVLVIEHARRCTRDEAVTAARGEVARR  262
Pentalenene (S. exfoliatus)                     MSAMLQIAVDVNLLLNDIASLEKEE-ARGEQNNMVMILRREHGWSKSRSVSHMQNEVRAR  262

Consensus                                       LERFLXLEEEL-PELXPEL---GXX-----------LDGXRGWIRGNYDWXR-XXXRYXA  309
Selina-4(15),7(11)-diene (S. pristinaespiralis) MCLFTTVSEQLAEQGSPQLR--QY------------LHSLRCFIRGAQDWGI-SSVRYTT  313
Polytrichastrene A (C. polytrichastri)          LKEFLLLQENL-PSFD------KWQNMVYDY-----VQDLGIMVAGVYAWHTNDTTRYVN  325
Spiroviolene (S. violens)                       LLRFMAVHDRVRPGAGLELS--TY------------LQGLRYGIRGNAEWGL-RVPRYLS  315
a-Amorphene (S. viridochromogenes)              IREFEDLYGEVRPELSPSGI--GY------------VEGMAGWIRGCYFWSR-TVPRYAD  327
Germacrene A (M. marina)                        LATYAERDAAL-SAVADE----GMRAF---------LDTRRDWIRATYDWSR-AAGRYA-  317
Caryolan-1-ol (S. griseus subsp. griseus)       AERVQRAERELIEEIEAA----GIDGPTRTALERC-VRDYRGLVRGDFDYHA-RAERYTR  317
epi-Cubenol (S. griseus subsp. griseus)         TERFLTAEDEL-LELYPELS--GMLVPY--------LDGMRSWMRGNLDWSR-QTPRYNP  298
7-epi-a-Eudesmol (S. viridochromogenes)         LDEYLELQERV-PQMCDEL---RLDEAQRDGVRLG-VEAIQHWINGNYEWAL-TSGRYAA  320
Avermitilol (S. avermitilis)                    VIRFEQLAREV-PALCAQL---GLSAVERAHVDTY-LGVMEAWMSGYHAWQT-QTRRYTG  316
Pentalenene (S. exfoliatus)                     LEQYLLLESCL-PKVGEIY---QLDTAEREALERYRTDAVRTVIRGSYDWHR-SSGRYDA  317
```

Consensus	ADXVAAXX--------------XRGEXDXLXXVAWWWDXAXXXXRSVRRQVPAQRSA	352
Selina-4(15),7(11)-diene (S. pristinaespiralis)	PDDPANMPSVFTDVPTD-----DSTEPLDIPAVSWWWDLLAEDARSVRRQVPAQRSA	365
Polytrichastrene A (C. polytrichastri)	GGYVDAEFI-------------TQGE----------------------------------	338
Spiroviolene (S. violens)	LGRVPDPMDEAPLEWAESPADDDRSAPRGLPTVAWWWDDALLGV-------------	359
a-Amorphene (S. viridochromogenes)	TLTAPAGL---	335
Germacrene A (M. marina)	--	316
Caryolan-1-ol (S. griseus subsp. griseus)	PDLVELD---------------ERDSLSRHFAA-----------------------	335
epi-Cubenol (S. griseus subsp. griseus)	ADVGQYEEPEEYLEETVLGVPPARSETAAPAPCGAEAPRAR----------------	339
7-epi-a-Eudesmol (S. viridochromogenes)	AKEGAVATAELA----------GRGSVDDLLTV-----------------------	343
Avermitilol (S. avermitilis)	APHVLPST--------------GPGYFDEVLPT-----------------------	335
Pentalenene (S. exfoliatus)	EFALAAG---------------AQGYLEELGSSAH----------------------	337

Figure S56. Amino acid sequence alignment of SdS with CpPS and several other characterised sesqui- and diterpene synthases. The highly conserved proline, aspartate-rich motif (DDXXD), the pyrophosphate sensor, the NSE triad, the Trp and the RY pair are marked in yellow. Active site residues (summarised in Table S2 for all characterised type I enzymes and in Table S7 for CpPS) are marked in green. They are located two and six positions upstream of the highly conserved Pro (itself 21 positions upstream of the Asp-rich motif), three, four, seven and eleven positions upstream of the Asp-rich motif, four, five and six positions downstream of the pyrophosphate sensor, three and four positions upstream of the NSE triad, and seven positions upstream of the conserved Trp, itself located six or seven positions upstream of the RY pair. Active site residues of CpPS mutated in this study are highlighted in bold.

Table S7. Sum of calculated van der Waals volumes (Å^3) of active site residues in wildtype polytrichastrene synthase (CpPS) and its enzyme variants and their activity with GGPP.

**Enzyme**	**Active site residues, CpPS position**													**ΣV$_{vdW}$ (Å^3)**	**activity[a]**
	**66**	**70**	**83**	**87**	**90**	**91**	**186**	**190**	**191**	**192**	**229**	**230**	**308**		
CpPS (wildtype)	I	G	L	A	G	A	R	S	G	A	I	G	M	550	100±22%
CpPS (I66F)	F	G	L	A	G	A	R	S	G	A	I	G	M	570	195±43%
CpPS (A87F)	I	G	L	F	G	A	R	S	G	A	I	G	M	622	–
CpPS (G90F)	I	G	L	A	F	A	R	S	G	A	I	G	M	640	23±2%
CpPS (A91F)	I	G	L	A	G	F	R	S	G	A	I	G	M	622	–
CpPS (M308W)	I	G	L	A	G	A	R	S	G	A	I	G	W	589	–
CpPS (A87T)	I	G	L	T	G	A	R	S	G	A	I	G	M	576	117±6%
CpPS (A192V)	I	G	L	A	G	A	R	S	G	V	I	G	M	584	176±8%
CpPS (A87T, A192V)	I	G	L	T	G	A	R	S	G	V	I	G	M	610	108±13%

[a] Mean ± standard deviation from triplicates. Relative amounts for the production of individual compounds are given in Table S9.

Site-directed mutagenesis

The single and double mutations were performed by the overlap extension PCR (OE-PCR) method.[82] The expression plasmid that contained the wildtype gene[1] was used as a template, and the Q5® High-Fidelity DNA polymerase was purchased from NEB (Ipswich, Massachusetts, USA). The primers used for PCR were designed to carry suitable mutations to obtain the desired amino acid exchanges (Table S8) and were used to amplify the CpPS gene in two or three overlapping fragments. These first-round PCRs were performed using the following temperature program: 1) 98 °C for 30 s; 2) 98 °C for 10 s, 64 °C for 30 s, 72 °C for 40 s; repeated 32 times; 3) 72 °C for 2 min. In the second-round PCR the two or three fragments were mixed. Amplification was achieved through the following process. Step 1: 1) 98 °C for 30 s; 2) 98 °C for 10 s, 60 °C for 30 s, 72 °C for 45 s; repeated 5 times; 3) 72 °C for 2 min. Step 2: addition of primers 23I4 and 23I5 for amplification of the whole *CpPS* gene carrying additional homology arms for cloning into the expression vector pYE-Express by homologous recombination. The PCR was then continued: 1) 98 °C for 30 s; 2) 98 °C for 10 s, 64 °C for 30 s, 72 °C for 45 s; repeated 32 times; 3) 72 °C for 2 min. The mutation gene was then analyzed by gel electrophoresis and purified by the Wizard® SV Gel and PCR Clean-Up System (Promega, Madison, Wisconsin, USA).

The mutated genes were incorporated into the pET28 based expression vector pYE-Express by homologous recombination in yeast using the standard PEG/LiOAc/salmon sperm protocol.[83,84] *S. cerevisiae* cultures containing the plasmids were grown on SM-URA plates for 3 days. The colonies were collected to isolate the plasmid mixture by using the Zymoprep™ Yeast Plasmid Miniprep II kit (Zymo Research, Irvine, CA, USA). The isolated plasmids were introduced into *E. coli* BL21(DE3) electrocompetent cells by electroporation. Cells were plated on LB medium and grown over night. Single colonies were picked and used to inoculate 3 mL LB cultures (kanamycin). After incubation at 37 °C for 12 h, the plasmids were extracted by the PureYield™ Plasmid Miniprep System (Promega) and checked by sequencing. The transformants containing the correct mutations were used for protein expression.

Table S8. Primers used for site-directed mutagenesis of CpPS.

Enzyme variant	Primer	Nucleotide sequence (5‘ → 3‘)[a]
CpPS wildtype	23I4	GGCAGCCATATGGCTAGCATGACTGGTGGAATGAAAACATCAATTTCAAACGAAGAATTCTATG
	23I5	TCTCAGTGGTGGTGGTGGTGGTGCTCGAGTCTATTCTCCCTGAGTGATAAATTCAGC
A87T		
fragment 1	23I2	ATGAAAACATCAATTTCAAACGAAGAATTCTATG
	38D8	CATCCATCATAGCTCCATTAGCGGTGTAACTGGCCAAAGGGC
fragment 2	38D7	GCCCTTTGGCCAGTTACACCGCTAATGGAGCTATGATGGATG
	23I3	CTATTCTCCCTGAGTGATAAATTCAGC
A192V		
fragment 1	23I2	ATGAAAACATCAATTTCAAACGAAGAATTCTATG
	38E1	TGCGACATATTTACAGAATGGTTGCACCCCACTTGTAGCCTCAC
fragment 2	38D9	GTGAGGCTACAAGTGGGGTGCAACCATTCTGTAAATATGTCGCA
	23I3	CTATTCTCCCTGAGTGATAAATTCAGC

[a] Homology arms for gene cloning are shown in red. Triplet codon exchanges introduced for mutations are underlined.

Table S8 (continued). Primers used for site-directed mutagenesis of CpPS.

**Mutation**	**Primer**	**Nucleotide sequence (5‘ → 3‘)[a]**
A87T, A192V		
fragment 1	23I2	ATGAAAACATCAATTTCAAACGAAGAATTCTATG
	38D8	CATCCATCATAGCTCCATTAGCGGTGTAACTGGCCAAAGGGC
fragment 2	38D7	GCCCTTTGGCCAGTTACACCGCTAATGGAGCTATGATGGATG
	38E1	TGCGACATATTTACAGAATGGTTGCACCCCACTTGTAGCCTCAC
fragment 3	38D9	GTGAGGCTACAAGTGGGGTGCAACCATTCTGTAAATATGTCGCA
	23I3	CTATTCTCCCTGAGTGATAAATTCAGC
I66F		
fragment 1	23I2	ATGAAAACATCAATTTCAAACGAAGAATTCTATG
	37A5	GCAACCTCGGGATGCAAAATCAGTCAAGTTATGTTTTTTGTGC
fragment 2	37A4	GCACAAAAAACATAACTTGACTGATTTTGCATCCCGAGGTTGC
	23I3	CTATTCTCCCTGAGTGATAAATTCAGC
A87F		
fragment 1	23I2	ATGAAAACATCAATTTCAAACGAAGAATTCTATG
	37A7	CATCCATCATAGCTCCATTAGCAAAGTAACTGGCCAAAGGGC
fragment 2	37A6	GCCCTTTGGCCAGTTACTTTGCTAATGGAGCTATGATGGATG
	23I3	CTATTCTCCCTGAGTGATAAATTCAGC
G90F		
fragment 1	23I2	ATGAAAACATCAATTTCAAACGAAGAATTCTATG
	37A9	GTATCCCAATAATCATCCATCATAGCAAAATTAGCTGCGTAACTGGCC
fragment 2	37A8	GGCCAGTTACGCAGCTAATTTTGCTATGATGGATGATTATTGGGATAC
	23I3	CTATTCTCCCTGAGTGATAAATTCAGC
A91F		
fragment 1	23I2	ATGAAAACATCAATTTCAAACGAAGAATTCTATG
	38B2	CAAGTATCCCAATAATCATCCATCATAAATCCATTAGCTGCGTAACTGG
fragment 2	38B1	CCAGTTACGCAGCTAATGGATTTATGATGGATGATTATTGGGATACTTG
	23I3	CTATTCTCCCTGAGTGATAAATTCAGC
M308W		
fragment 1	23I2	ATGAAAACATCAATTTCAAACGAAGAATTCTATG
	38B4	GCATAAACGCCGGCTACCCAAAATGCCAAGATCCTGTACATAATC
fragment 2	38B3	GATTATGTACAGGATCTTGGCATTTGGGTAGCCGGCGTTTATGC
	23I3	CTATTCTCCCTGAGTGATAAATTCAGC

[a] Homology arms for gene cloning are shown in red. Triplet codon exchanges introduced for mutations are underlined.

Expression and purification of enzyme variants

The transformants harboring the mutated gene were inoculated in LB medium (3 mL, kanamycin). The cultures were incubated at 37 °C overnight to form the precultures, 0.4 mL of the precultures were then used to inoculate the expression culture (LB medium, 400 mL). The cultures were incubated at 37 °C until an OD_{600} = 0.4 – 0.6 was reached, and then cooled to 18 °C. IPTG (400 µL, 400 mM in H_2O) was added to induce the protein expression, followed by incubation with shaking at 18 °C for 16 h.

The *E. coli* cells were harvested by centrifugation (3500 rpm, 30 min). The cells were suspended in binding buffer (8 mL; 50 mM Tris, 300 mM NaCl, 20 mM imidazole, 2 mM β-mercaptoethanol, 1 mM $MgCl_2$, pH = 7.6) and then lyzed by ultra-sonification (50% power, on ice, 6 x 45 s). The lysates were then centrifuged to remove the debris, and supernatant was filtered through a syringe filter (0.45 µm) and loaded onto a Ni^{2+}-NTA affinity chromatography column (Protino™ Ni-NTA, MachereyNagel, Düren, Germany). The column was then washed with binding buffer (1 mL), washing buffer (2 x 1 mL; 50 mM Tris, 300 mM NaCl, 60 mM imidazole, 2 mM β-mercaptoethanol, 1 mM $MgCl_2$, pH = 7.6), and the protein was eluted by using elution buffer (1 mL; 50 mM Tris, 300 mM NaCl, 300 mM imidazole, 5% glycerol, pH = 7.6).

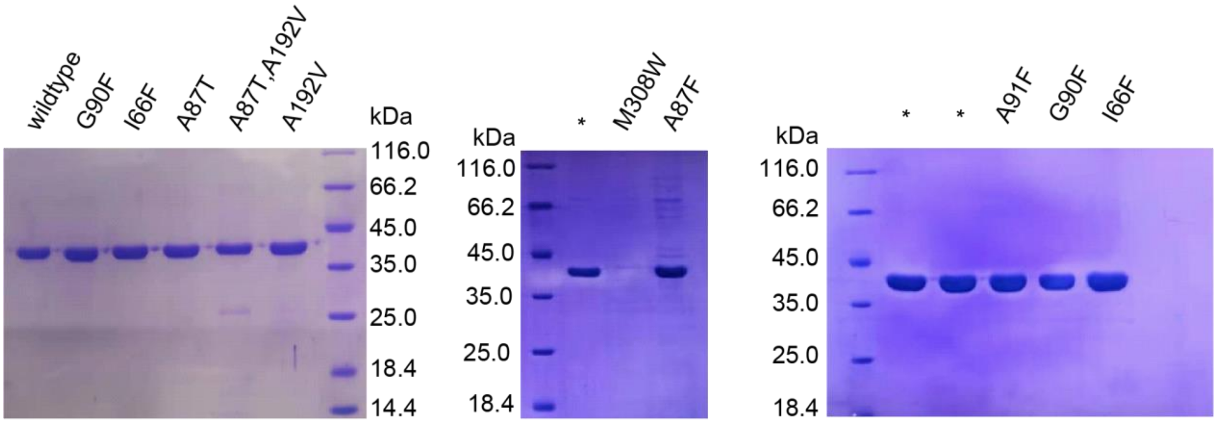

Figure S57. SDS-PAGE analysis of the purified CpPS variants. Asterisks indicate additional CpPS variants not reported here.

Activity of enzyme variants

The concentration of the purified proteins was determined by the Bradford method,[85] and then adjusted to 0.8 mg mL^{-1} for all enzyme variants for activity testings. The protein preparation (0.1 mL), Tris buffer (0.3 mL; 50 mM Tris, 1 mM $MgCl_2$, pH 7.6), incubation buffer (0.5 mL; 50 mM Tris, 10 mM $MgCl_2$, 20% glycerol, pH = 7.6) and GGPP (0.1 mL; 1 mg mL^{-1} in 25 mM aqueous NH_4HCO_3) were mixed and incubated at 28 °C overnight. The reaction was extracted with hexane (0.2 mL; containing 0.08 mg L^{-1} tetradecane as internal standard). The layers were separated by centrifugation (16000 rpm, 5 min). The organic layer was pipetted off and analyzed by GC/MS. Triplicates were performed for every enzyme variant.

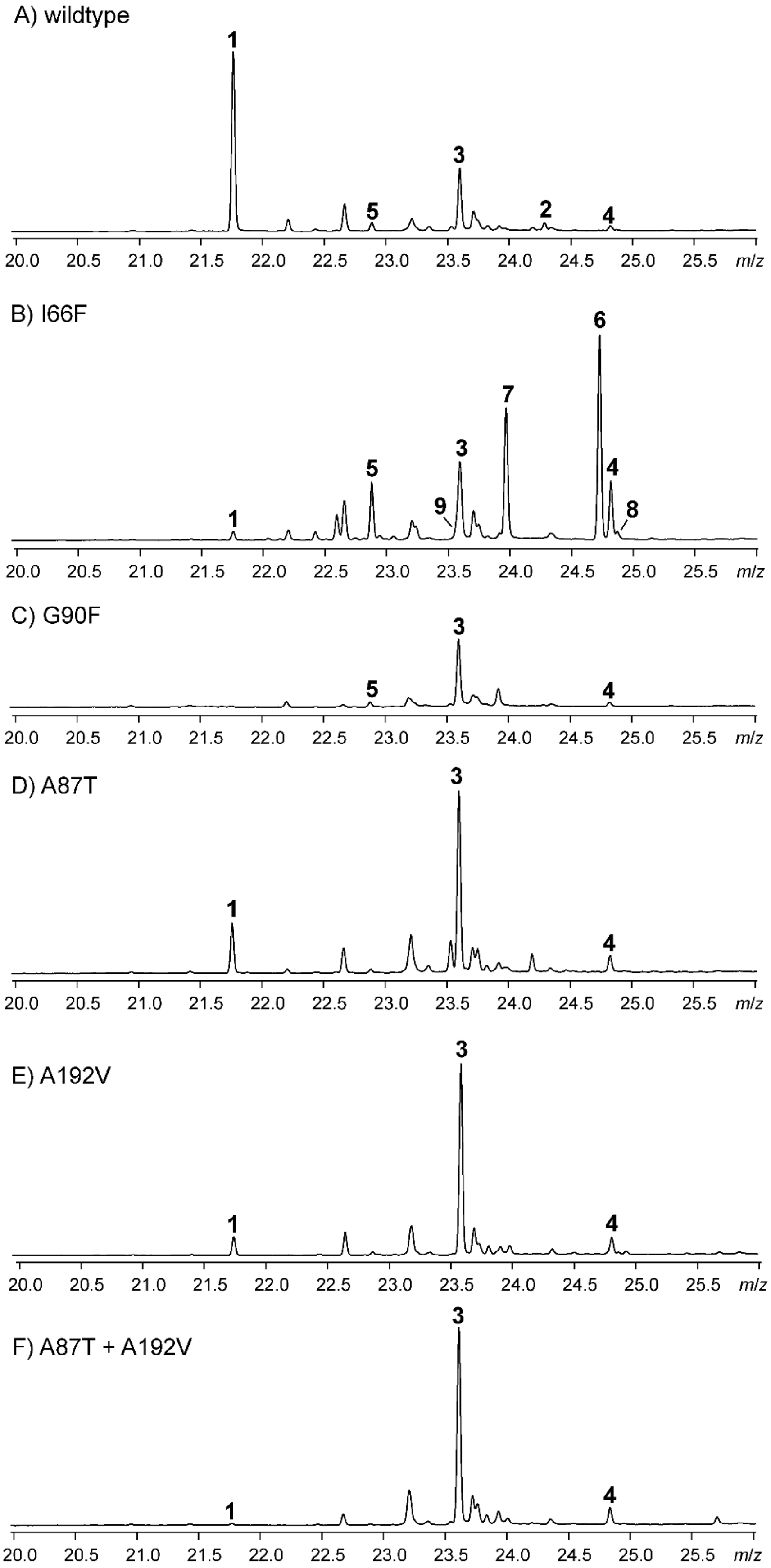

Figure S58. Total ion chromatograms of extracts from incubations of GGPP with A) wildtype CpPS, and the CpPS enzyme variants B) I66F, C) G90F, D) A87T, E) A192V, and F) A97T,A192V.

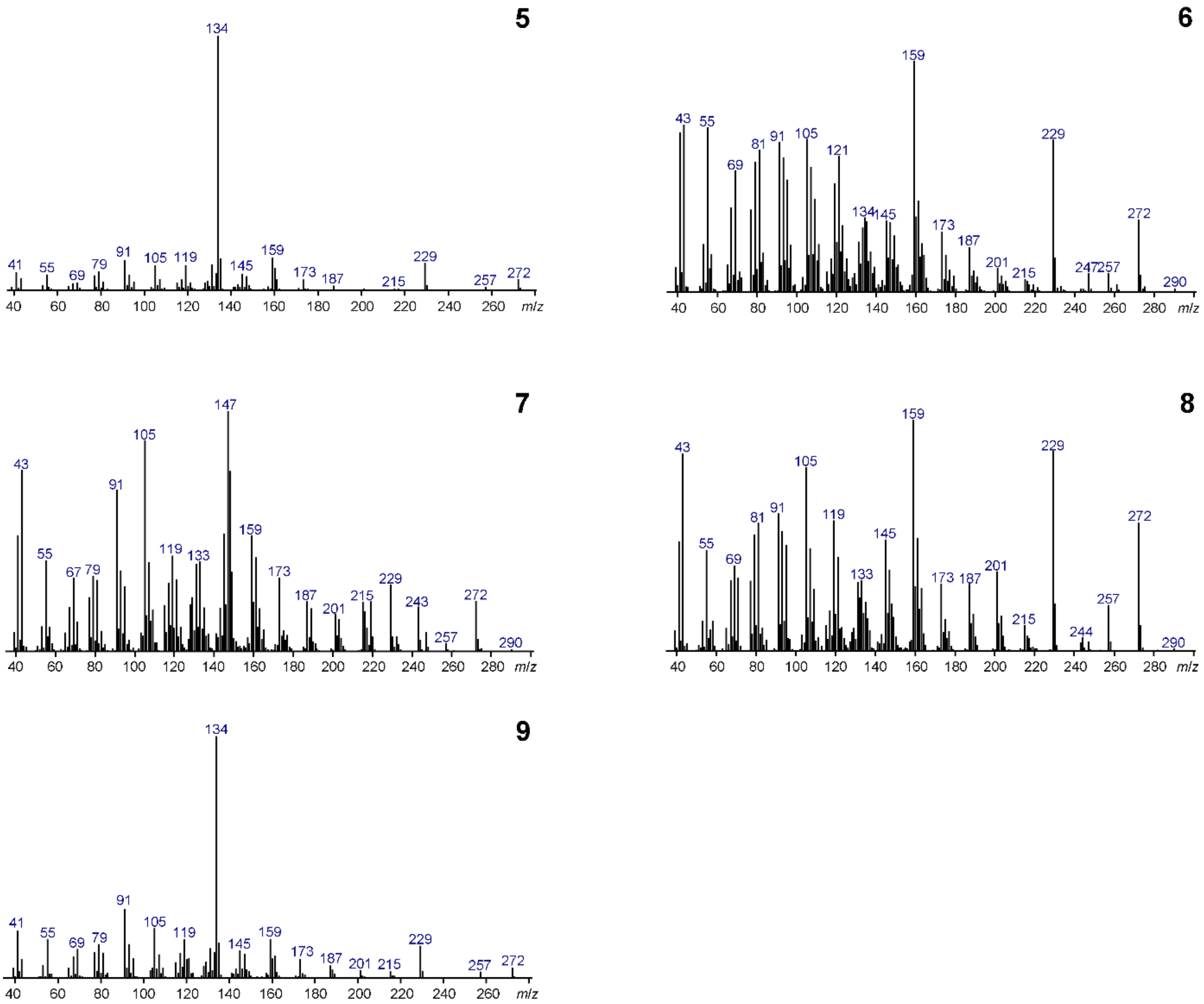

Figure S59. EI mass spectra of diterpenes obtained from CpPS enzyme variants. Mass spectrum of A) wanju-2,6-diene (**5**), B) wanju-2-en-6α-ol (**6**), C) polytrichastrol B (**7**), bonn-2-en-11α-ol (**8**), polytrichastrene B (**9**).

Table S9. Production of compounds **1** – **8** by wildtype CpPS and its enzyme variants, and relative enzyme activities.

**Enzyme variant**	**1**[a]	**2**	**3**	**4**	**5**	**6**	**7**	**8**	**activity**[b]
CpPS (wildtype)	100±19%	4.3±1.6%	37±7%	2.1±0.4%	4.8±0.8%	–	–	–	100±22%
CpPS (A87T)	25±2%	–	96±9%	10±0.3%	1.9±0.3%	–	–	–	117±6%
CpPS (A192V)	17±1%	–	184±8%	20±1%	3.2±0.4%	–	–	–	176±8%
CpPS (A87T, A192V)	–	–	114±10%	12±2%	–	–	–	–	108±13%
CpPS (I66F)	4.6±1.1%	–	56±13%[c]	31±9%	29±8%	113±24%	76±16%	4.8±0.9%	195±43%
CpPS (G90F)	–	–	24±2%	1.5±0.3%	1.7±0.3%	–	–	–	23±2%
CpPS (A87F)	–	–	–	–	–	–	–	–	–
CpPS (A91F)	–	–	–	–	–	–	–	–	–
CpPS (M308W)	–	–	–	–	–	–	–	–	–

[a] Production of compounds **1** – **9** by peak integration of total ion chromatograms from triplicates. Production of **1** by wildtype CpPS is set to 100%. [b] Enzyme activities were calculated from total production of all compounds by peak integrations from triplicates. Activity of wildtype CpPS is set to 100%. [c] Sum of peak areas for **3** and co-eluting **9**.

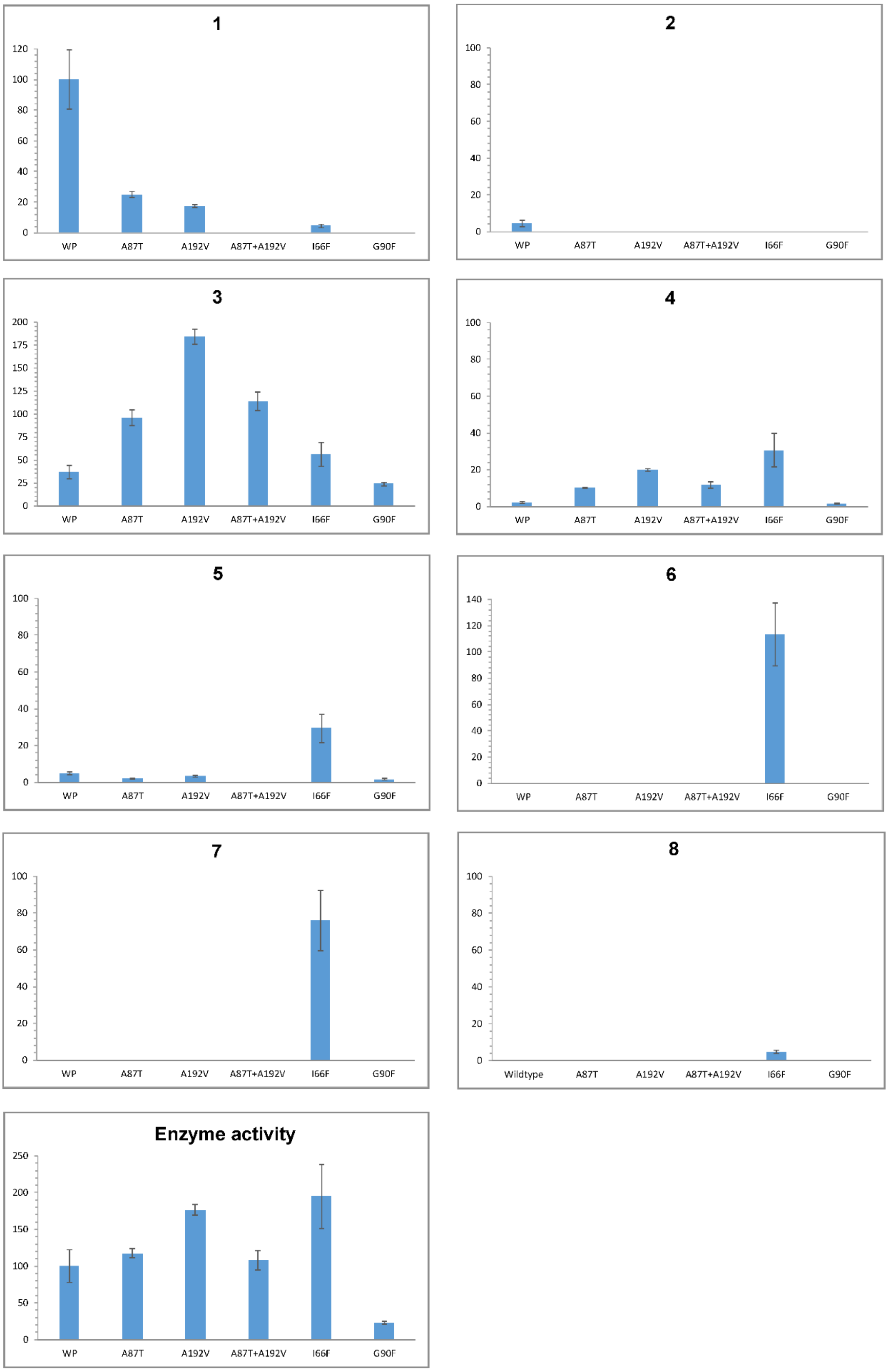

Figure S60. Production of compounds **1** – **9** by peak integration of total ion chromatograms from triplicates. Production of **1** by wildtype CpPS is set to 100%. Compound **9** was only produced by CpPS(I66F) and co-eluted with compound **3**. Enzyme activities were calculated from total production of all compounds by peak integrations from triplicates. Activity of wildtype CpPS is set to 100%.

Enzyme incubation of GGPP with CpPS (I66F) and compound isolation

The enzyme variant CpPS (I66F) was expressed and purified following the same procedure as described for wildtype CpPS.[66] GGPP trisammonium salt (50 mg, 0.13 mmol) was dissolved in NH_4HCO_3 solution (50 mL; 25 mM in H_2O), followed by addtion of Tris buffer (150 mL; 50 mm Tris, 1 mM $MgCl_2$, pH = 7.6) and incubation buffer (250 mL; 50 mM Tris, 5 mM $MgCl_2$, 5% glycerol, pH = 7.6). Then purified protein fraction (50 mL, ca. 5 mg/mL), obtained from 4 L of *E. coli* expression culture, was added. The reaction mixture was incubated at 28 °C overnight and extracted with hexane (2 x 300 mL). This enzymatic reaction was performed four times (total amount of converted GGPP trisammonium salt: 200 mg, 398.8 μmol), and all extracts were combined and purified via silica gel chromatograpy. The first fraction was obtained by elution with pentane to afford impure compounds **5** and **9**, which were further purified via $AgNO_3$ coated preparetive TLC (hexane) to afford compound **5** (1.4 mg, 5.1 μmol, 1.3%) and compound **9** (0.5 mg, 1.8 μmol, 0.5%). The next fractions were obtained through elution with hexane/Et_2O (5/1) to afford pure compounds **6** (6.2 mg, 21.3 μmol, 5.3%), **7** (3.8 mg, 13.1 μmol, 3.3%) and **8** (0.3 mg, 1.0 μmol, 0.3%).

Wanju-2,6-diene (5). Yield: 1.4 mg (5.1 μmol, 1.3%), from 200 mg (398.8 μmol) GGPP trisammonium salt. TLC (hexane): R_f = 0.83. GC (HP5-MS): *I* = 1895. IR (diamond ATR): ṽ = 2956 (m), 2924 (s), 2868 (m), 2853 (m), 2278 (w), 1710 (w), 1665 (w), 1566 (w), 1454 (m), 1444 (m), 1381 (w), 1373 (w), 1345 (w), 1261 (w), 1227 (w), 1181 (w), 1091 (w), 1019 (w), 946 (w), 838 (m), 816 (m), 802 (w), 745 (w), 643 (w), 626 (w), 587 (w), 543 (m), 466 (w), 438 (w), 417 (w) cm^{-1}. HR-MS (APCI): calc. for $[C_{20}H_{32}]^+$ m/z = 272.2499; found: m/z = 272.2500. Optical rotary power: $[\alpha]_D^{25}$ = +16.4 (*c* 0.14, CH_2Cl_2).

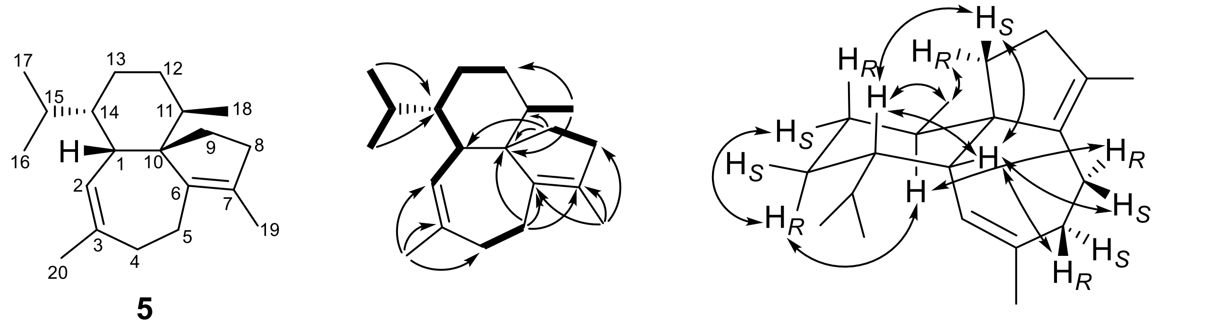

5

Figure S61. Structure elucidation of **5**. Bold: $^1H,^1H$-COSY, single headed arrows: key HMBC, and double headed arrows: NOESY correlations. Carbon numbering follows GGPP numbering to indicate the origin of each carbon. Diastereotopic hydrogens are labelled H_R (*pro-R*) and H_S (*pro-S*).

Table S10. NMR data of wanju-2,6-diene (**5**) in C_6D_6 recorded at 298 K.

C[a]	type	^{1}H[b]	^{13}C[b]
1	CH	2.51 (br s)	47.77
2	CH	5.37 (m)	125.23
3	C_q	–	140.10
4	CH_2	2.08 (m, H_R)	33.36
		1.88 (dddd, J = 13.8, 6.2, 1.4, 1.4, H_S)	
5	CH_2	2.44 (ddd, J = 13.5, 6.2, 1.6, H_S)	24.27
		1.67 (m, H_R)	
6	C_q	–	142.50
7	C_q	–	130.58
8	CH_2	2.13 (m, 2H)	36.68
9	CH_2	2.02 (m, H_R)	30.68
		1.47 (ddd, J = 13.3, 10.1, 7.9, H_S)	
10	C_q	–	58.24
11	CH	1.91 (m)	31.94
12	CH_2	1.55 (m, H_S)	31.96
		1.26 (m, H_R)	
13	CH_2	1.69 (m, H_S)	25.73
		1.33 (m, H_R)	
14	CH	1.26 (m)	44.36
15	CH	1.51 (m)	29.68
16	CH_3	0.88 (d, J = 6.6)	21.03
17	CH_3	0.93 (d, J = 6.6)	21.65
18	CH_3	0.68 (d, J = 6.8)	16.71
19	CH_3	1.62 (m)	14.05
20	CH_3	1.81 (m)	27.22

[a] Carbon numbering as shown in main text. [b] Chemical shifts δ in ppm, multiplicity: s = singlet, d = doublet, m = multiplet, br = broad, coupling constants J are given in Hertz.

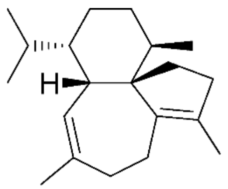

5

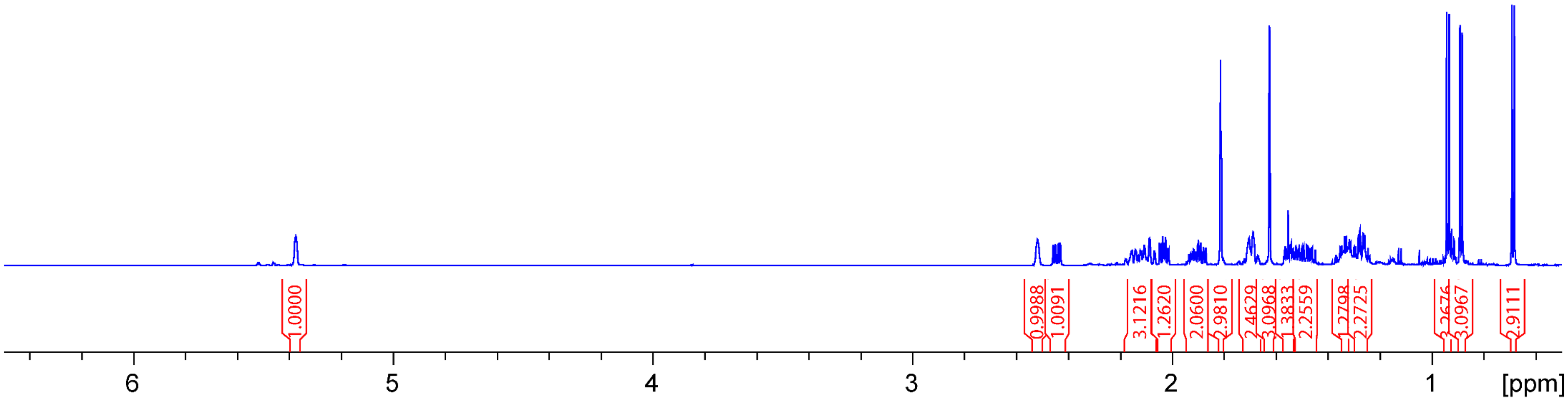

Figure S62. ^{1}H-NMR spectrum of **5** (700 MHz, C_6D_6).

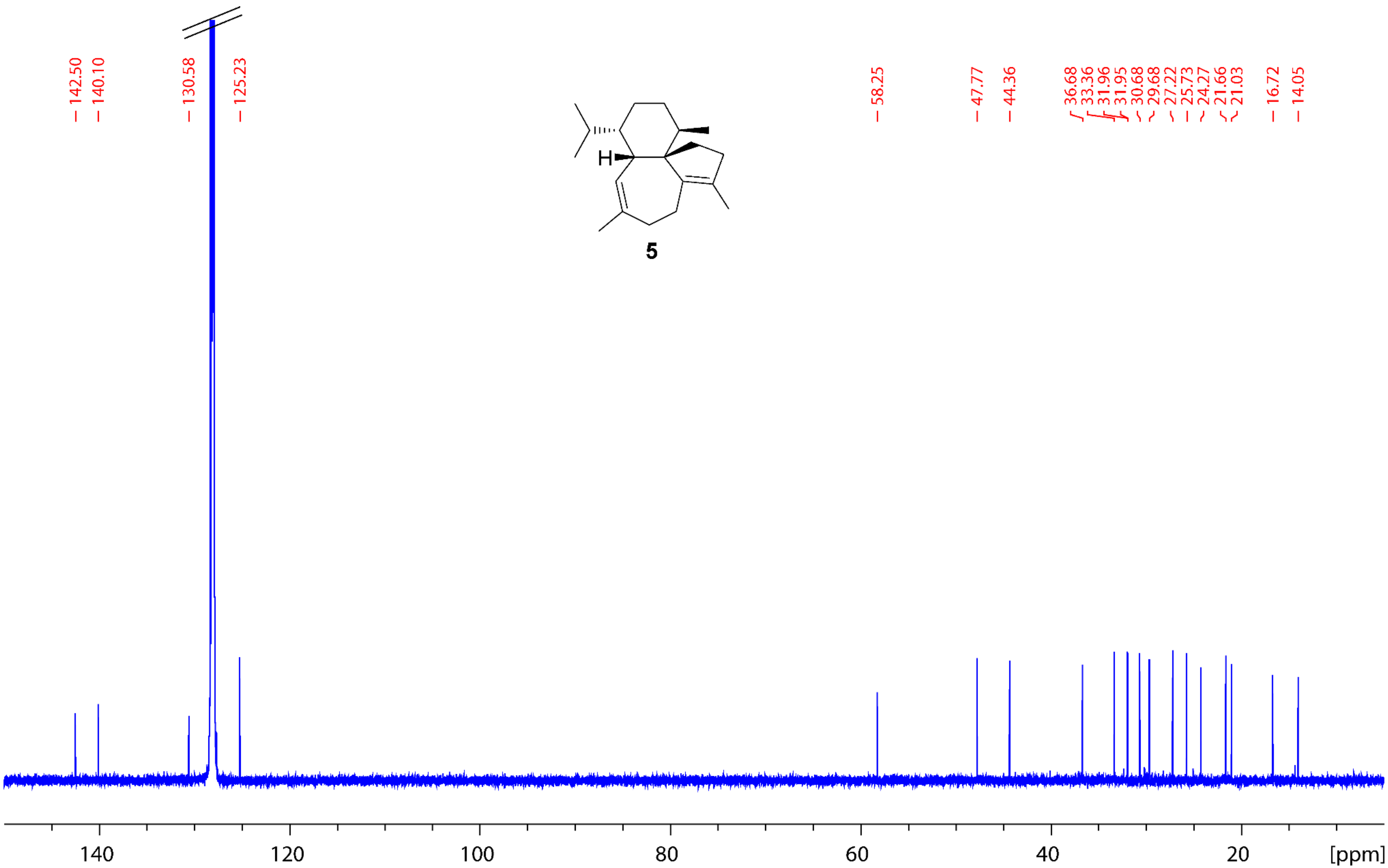

Figure S63. ^{13}C-NMR spectrum of **5** (176 MHz, C_6D_6).

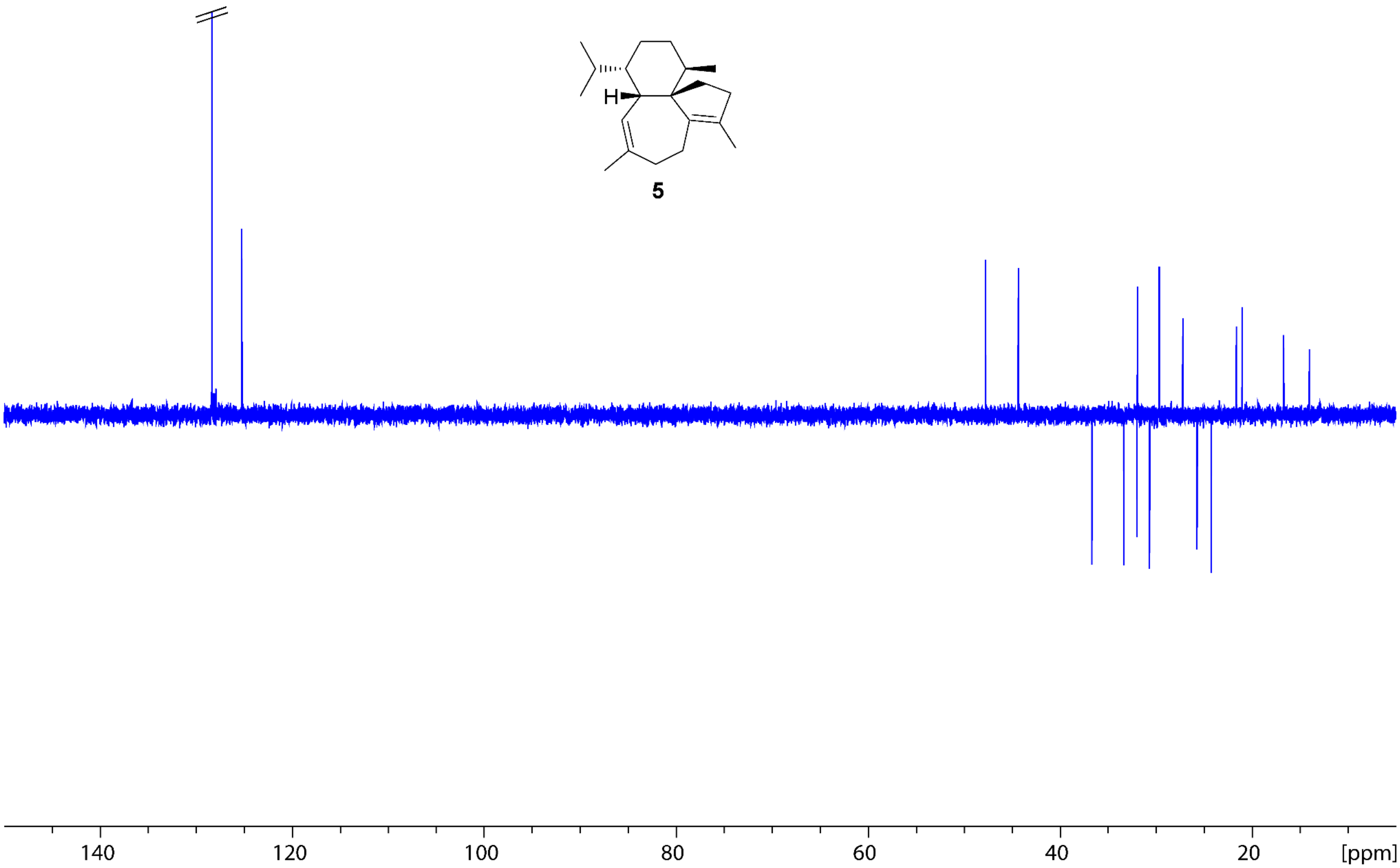

Figure S64. ^{13}C-DEPT spectrum of **5** (176 MHz, C_6D_6).

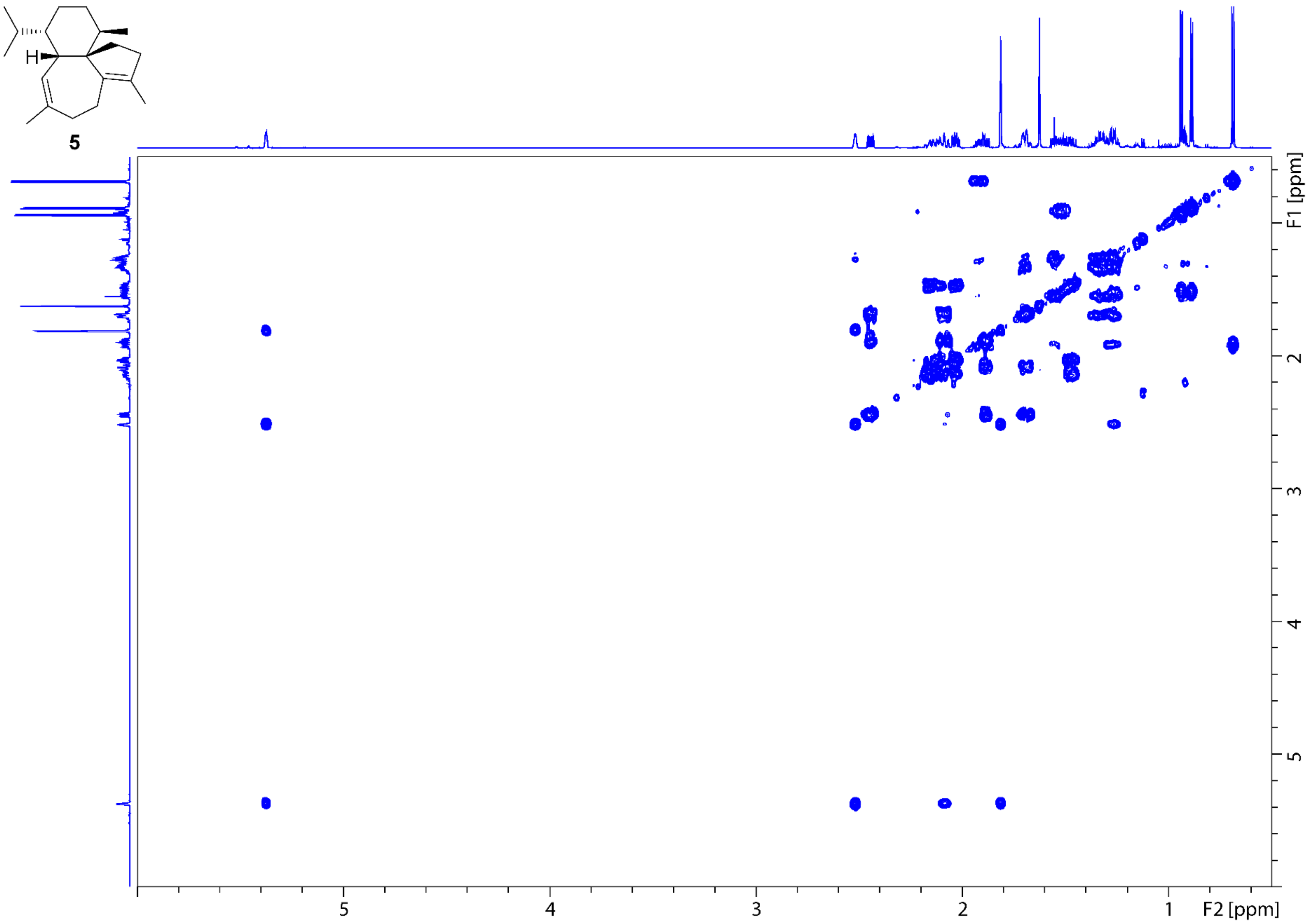

Figure S65. ^{1}H-^{1}H-COSY spectrum of **5** (700 MHz, C_6D_6).

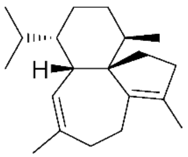

5

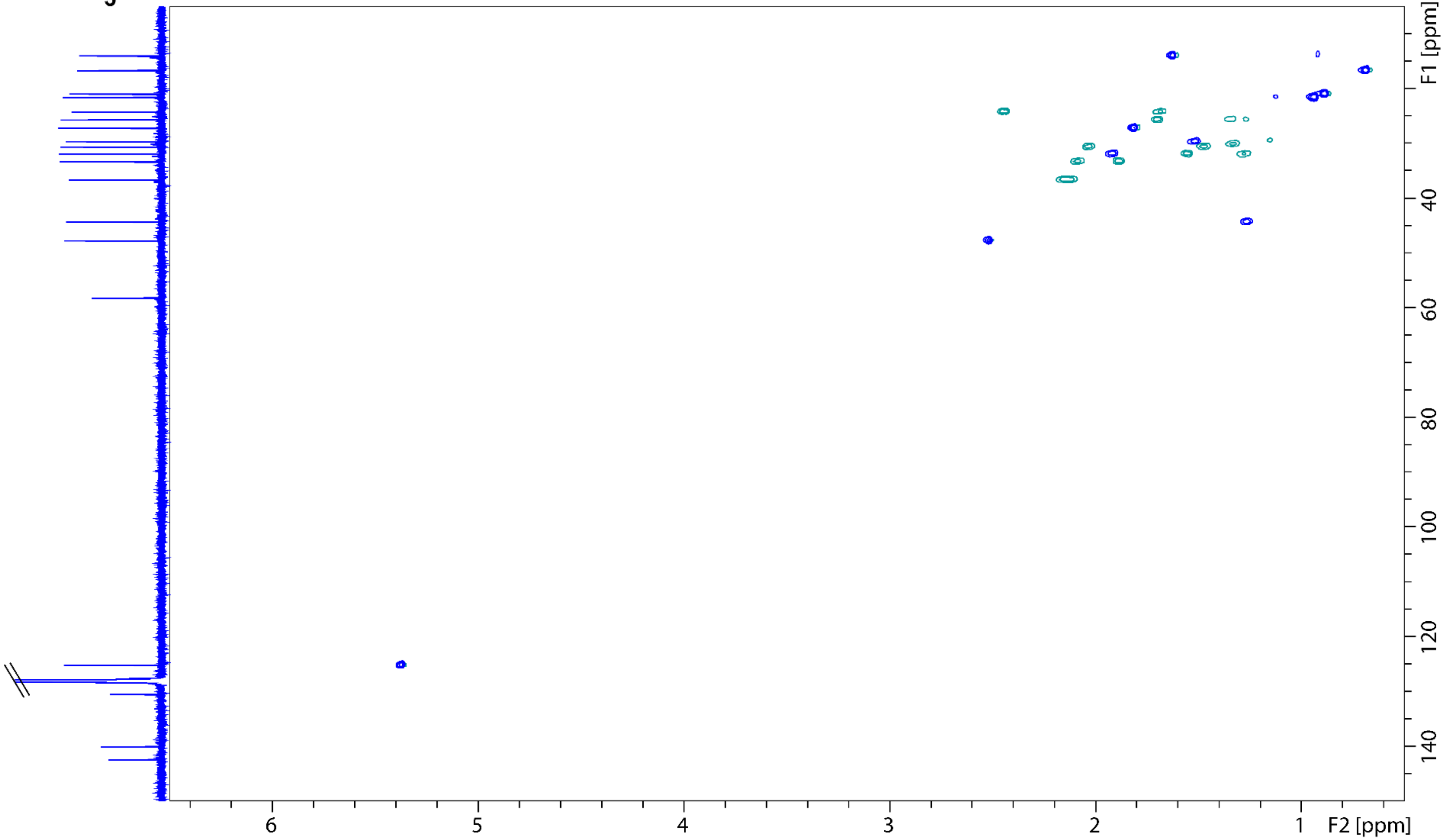

Figure S66. HSQC spectrum of **5** (C_6D_6).

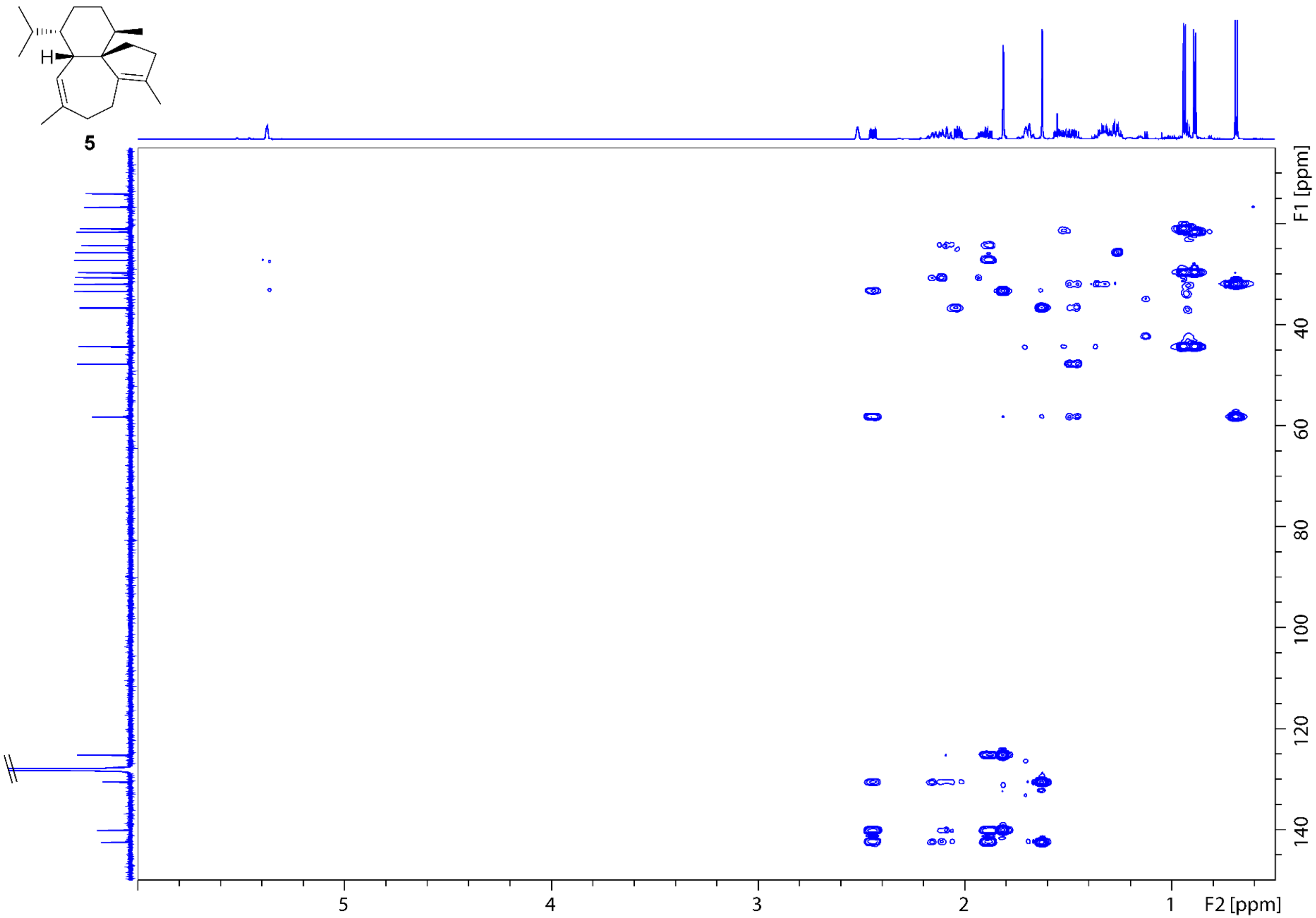

Figure S67. HMBC spectrum of **5** (C_6D_6).

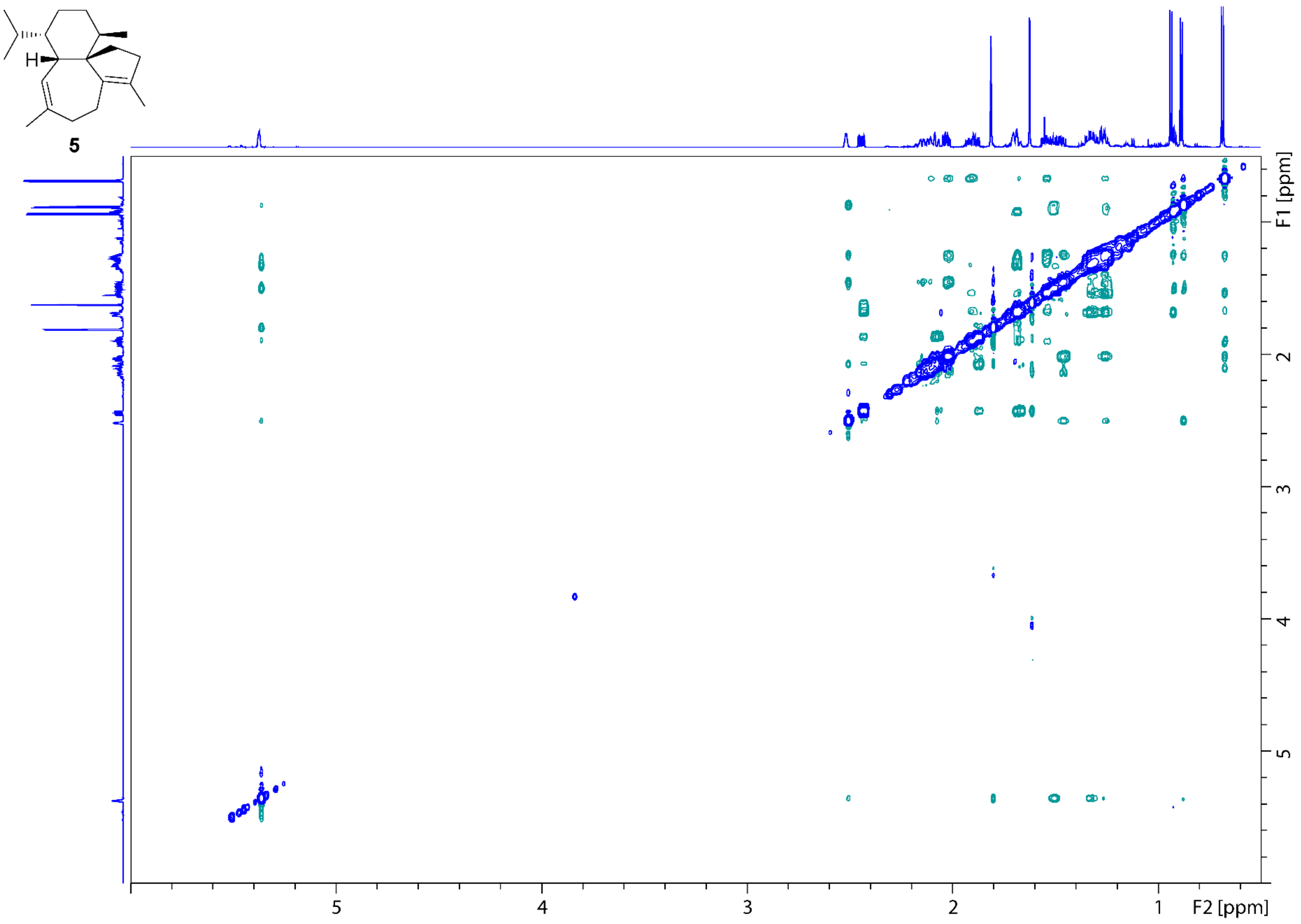

Figure S68. NOESY spectrum of **5** (C_6D_6).

Wanju-2-en-6α-ol (6). Yield: 6.2 mg (21.3 μmol, 5.3%), from 200 mg (398.8 μmol) GGPP trisammonium salt. TLC (hexane/Et_2O = 5/1): R_f = 0.67. GC (HP5-MS): I = 2083. IR (diamond ATR): $\tilde{\nu}$ = 3594 (w), 2953 (s), 2925 (s), 2871 (s), 1671 (w), 1469 (m), 1454 (m), 1375 (w), 1365 (w), 1280 (w), 1218 (w), 1170 (w), 1079 (w), 1049 (w), 1030 (w), 1011 (w), 954 (w), 886 (w), 839 (w), 829 (w), 544 (w), 435 (w) cm^{-1}. HR-MS (Q-TOF, 70 eV): calc. for $[C_{20}H_{34}O + H]^+$ m/z = 291.2682; found: m/z = 291.2678. Optical rotary power: $[\alpha]_D^{25}$ = –3.1 (c 0.62, CH_2Cl_2).

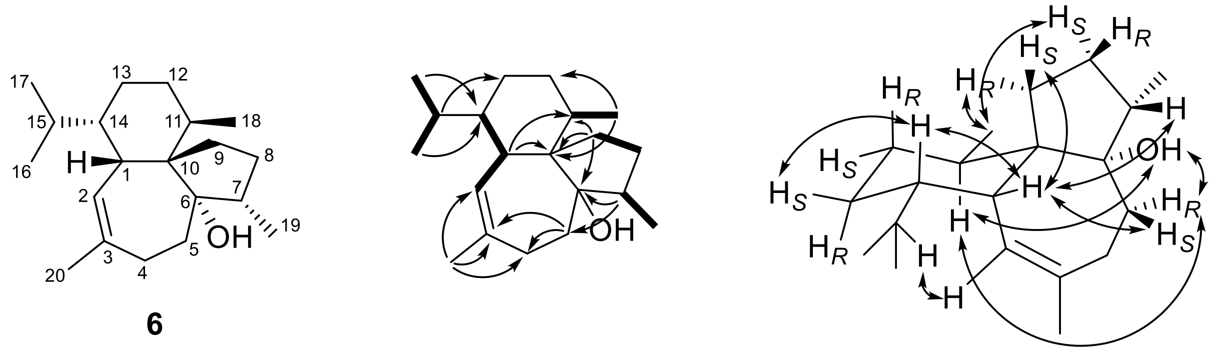

Figure S69. Structure elucidation of **6**. Bold: ^{1}H,^{1}H-COSY, single headed arrows: key HMBC, and double headed arrows: NOESY correlations. Carbon numbering follows GGPP numbering to indicate the origin of each carbon. Diastereotopic hydrogens are labelled H_R (*pro-R*) and H_S (*pro-S*).

Table S11. NMR data of wanju-2-en-6α-ol (**6**) in C_6D_6 recorded at 298 K.

C[a]	type	^{1}H[b]	^{13}C[b]
1	CH	2.74 (br s)	43.20
2	CH	5.19 (br d, *J* = 4.9)	124.75
3	C_q	–	135.14
4	CH_2	2.05 (m, 2H)	30.30
5	CH_2	2.07 (m, H_S)	37.20
		1.48 (m, H_R)	
6	C_q	–	85.08
7	CH	1.72 (m)	46.89
8	CH_2	1.57 (m, H_R)	32.44
		1.34 (dddd, *J* = 12.0, 12.0, 12.0, 6.9, H_S)	
9	CH_2	1.98 (ddd, *J* = 13.2, 7.1, 1.7, H_R)	32.59
		1.01 (m, H_S)	
10	C_q	–	57.42
11	CH	1.83 (m)	31.40
12	CH_2	1.52 (m, H_S)	34.07
		1.25 (m, H_R)	
13	CH_2	1.57 (m, H_S)	24.78
		1.25 (m, H_R)	
14	CH	1.04 (m)	44.11
15	CH	1.41 (m)	29.78
16	CH_3	0.89 (d, *J* = 6.6)	21.08
17	CH_3	0.88 (d, *J* = 6.6)	21.49
18	CH_3	1.15 (d, *J* = 6.7)	22.34
19	CH_3	1.04 (d, *J* = 7.0)	13.56
20	CH_3	1.60 (br s)	25.92
	OH	1.09 (br s)	

[a] Carbon numbering as shown in main text. [b] Chemical shifts δ in ppm, multiplicity: s = singlet, d = doublet, m = multiplet, br = broad, coupling constants *J* are given in Hertz.

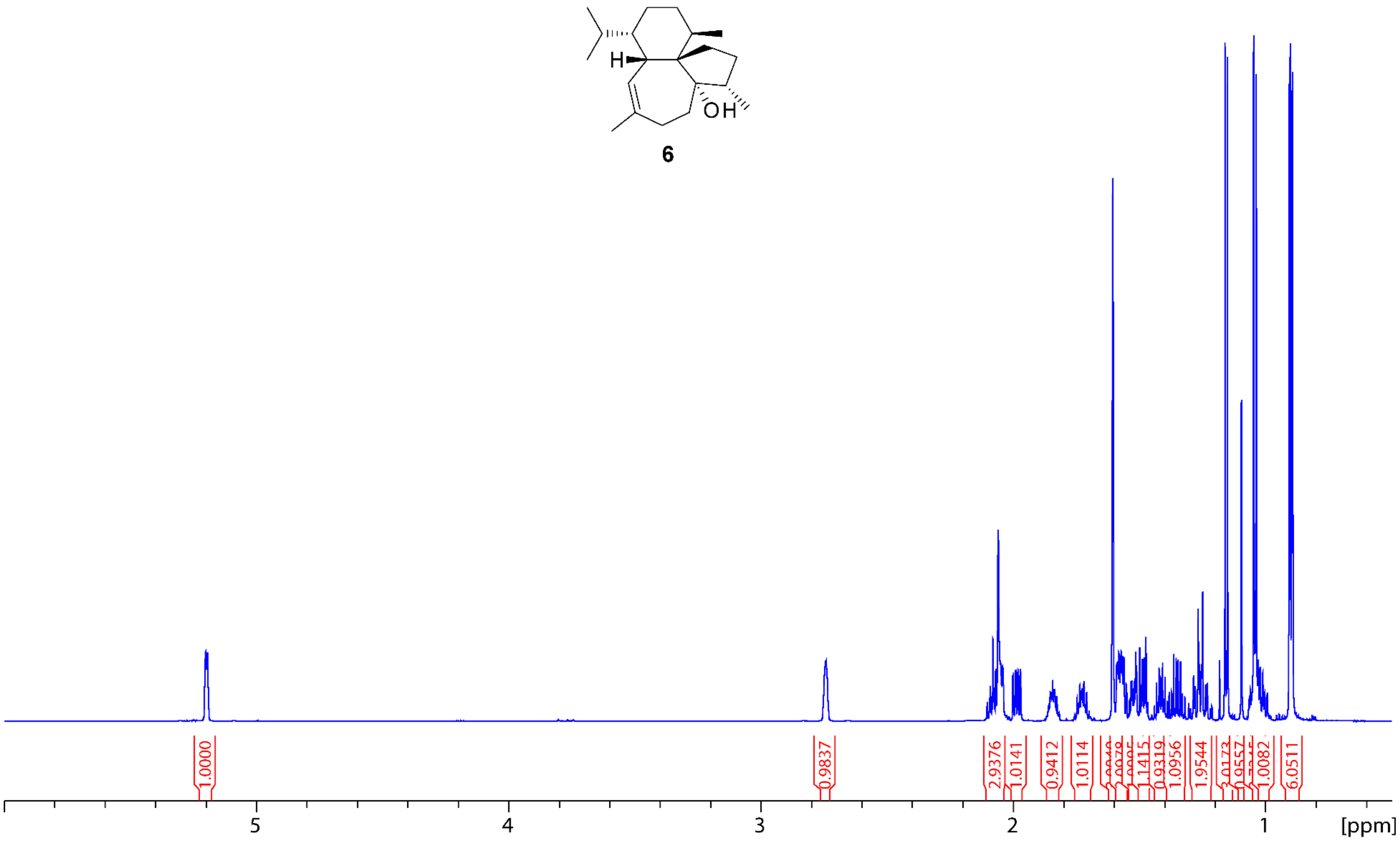

Figure S70. ^{1}H-NMR spectrum of **6** (700 MHz, C_6D_6).

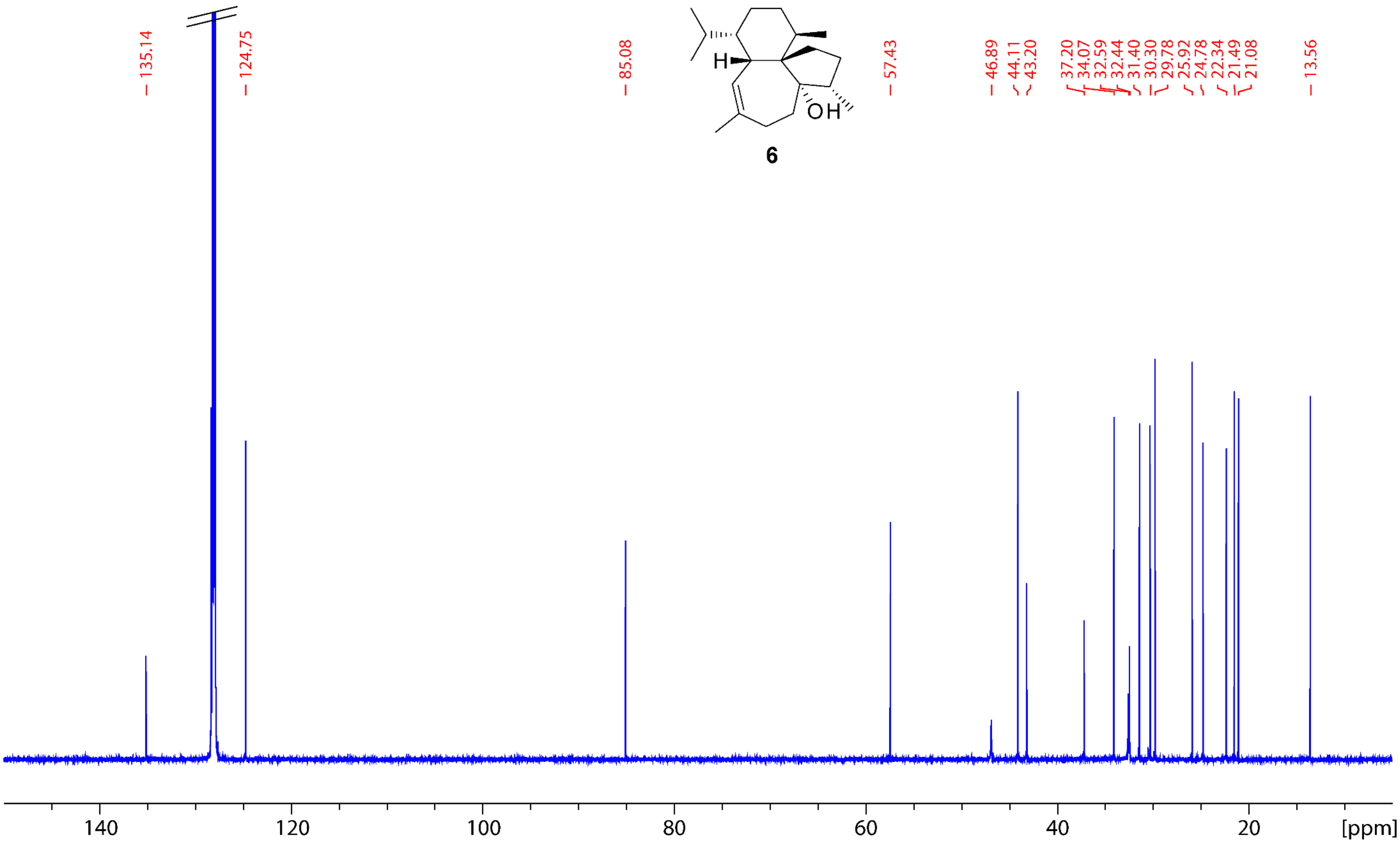

Figure S71. ^{13}C-NMR spectrum of **6** (176 MHz, C_6D_6).

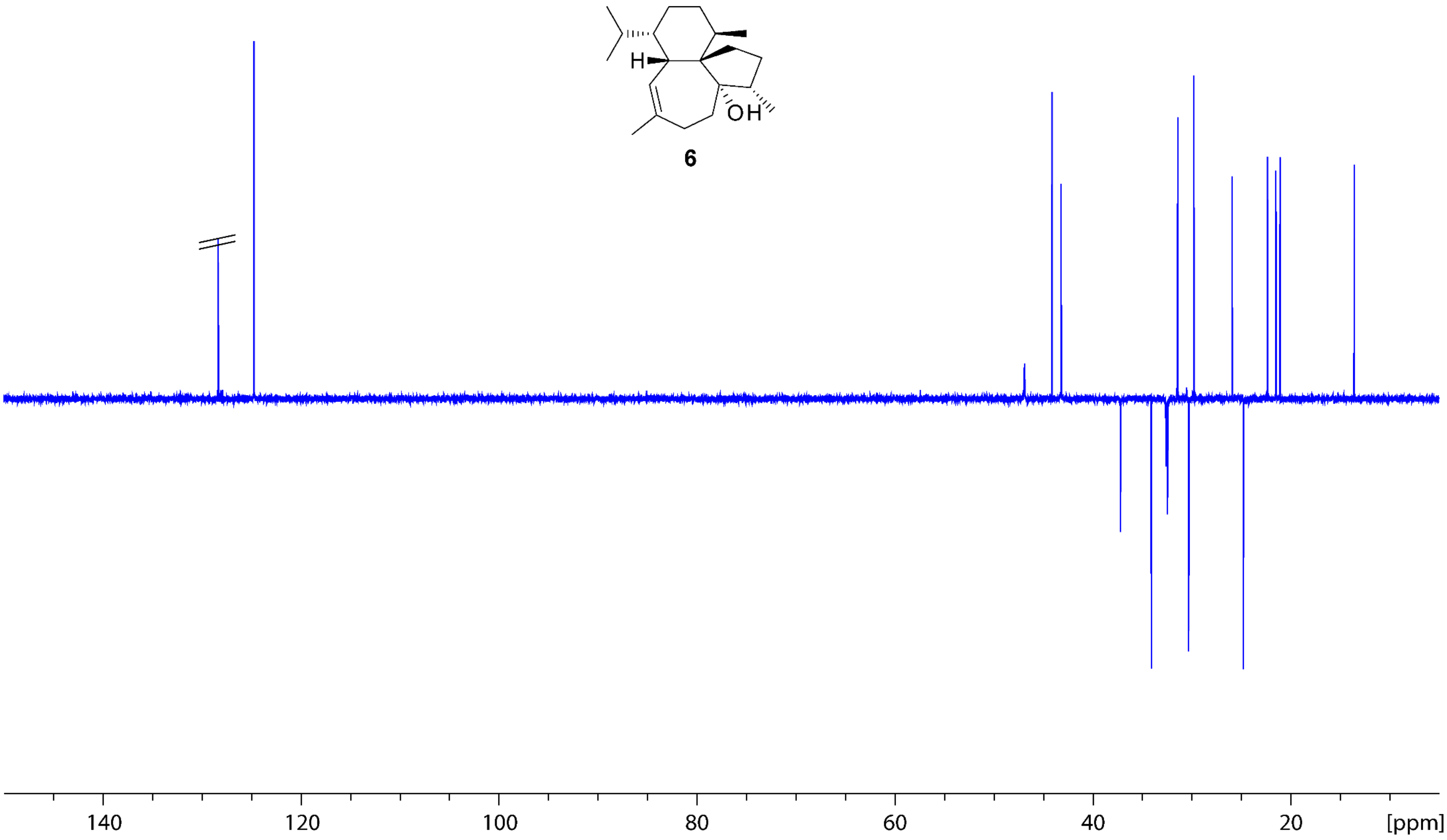

Figure S72. ^{13}C-DEPT spectrum of **6** (176 MHz, C_6D_6).

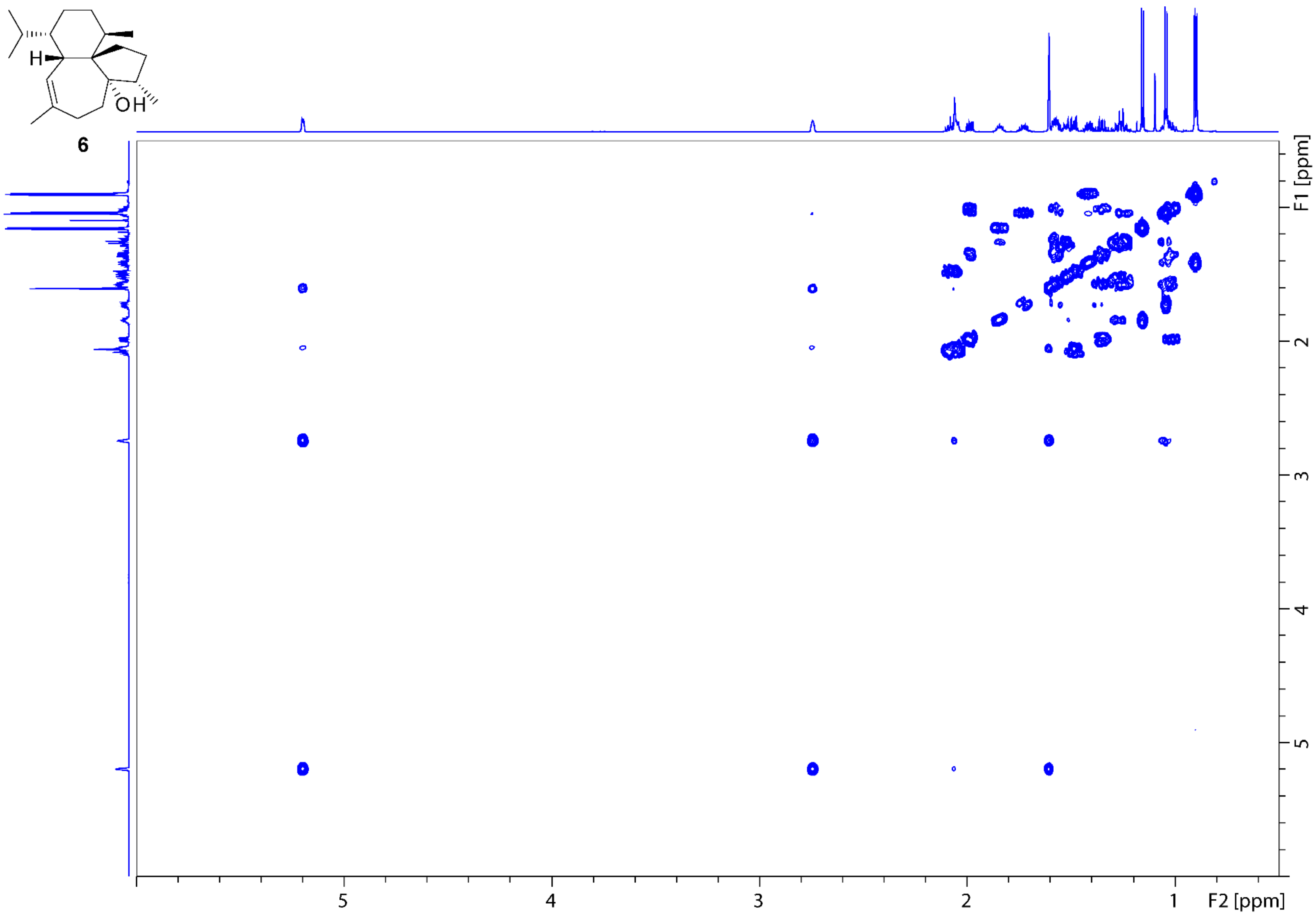

Figure S73. ^{1}H-^{1}H-COSY spectrum of **6** (700 MHz, C_6D_6).

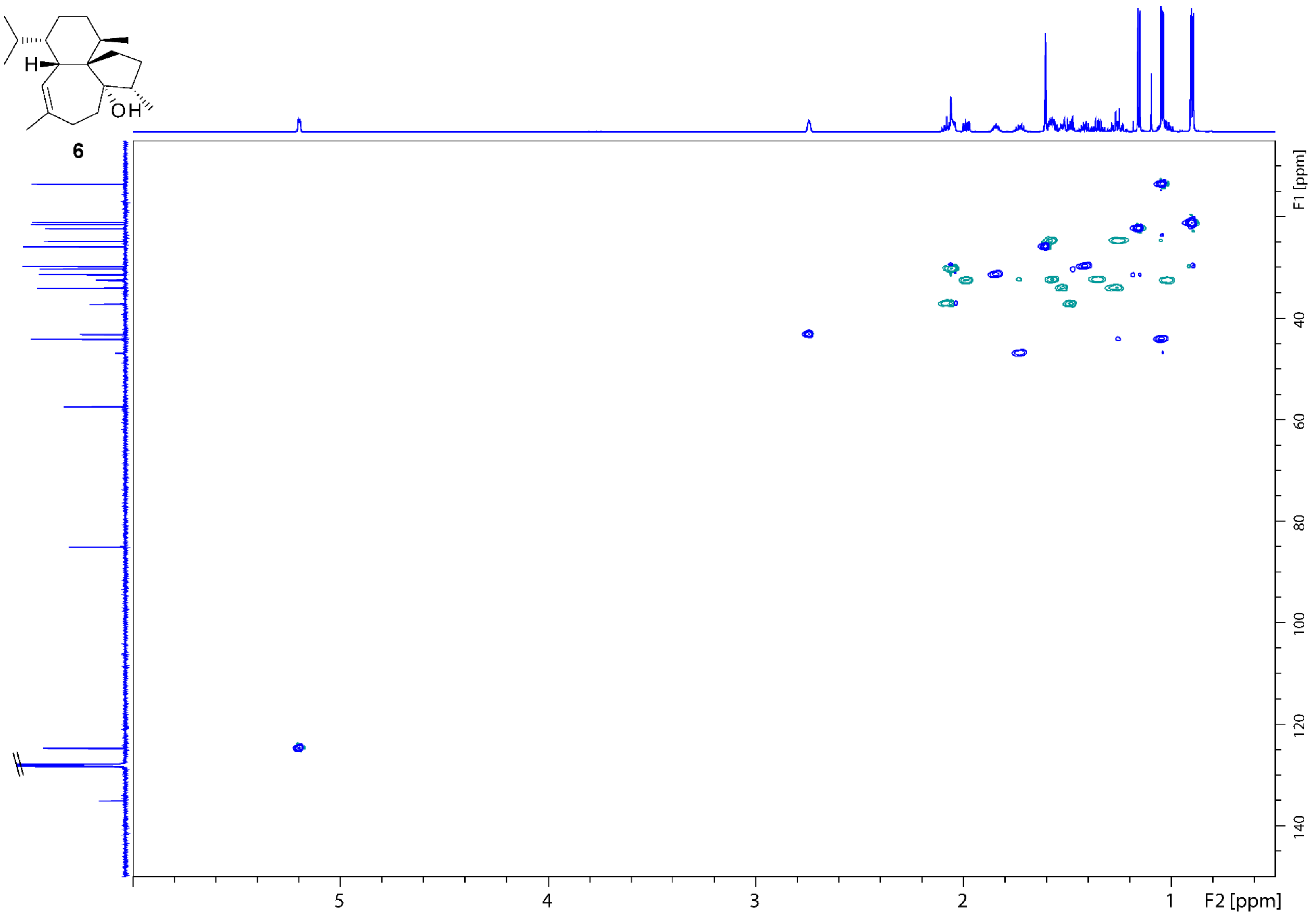

Figure S74. HSQC spectrum of **6** (C_6D_6).

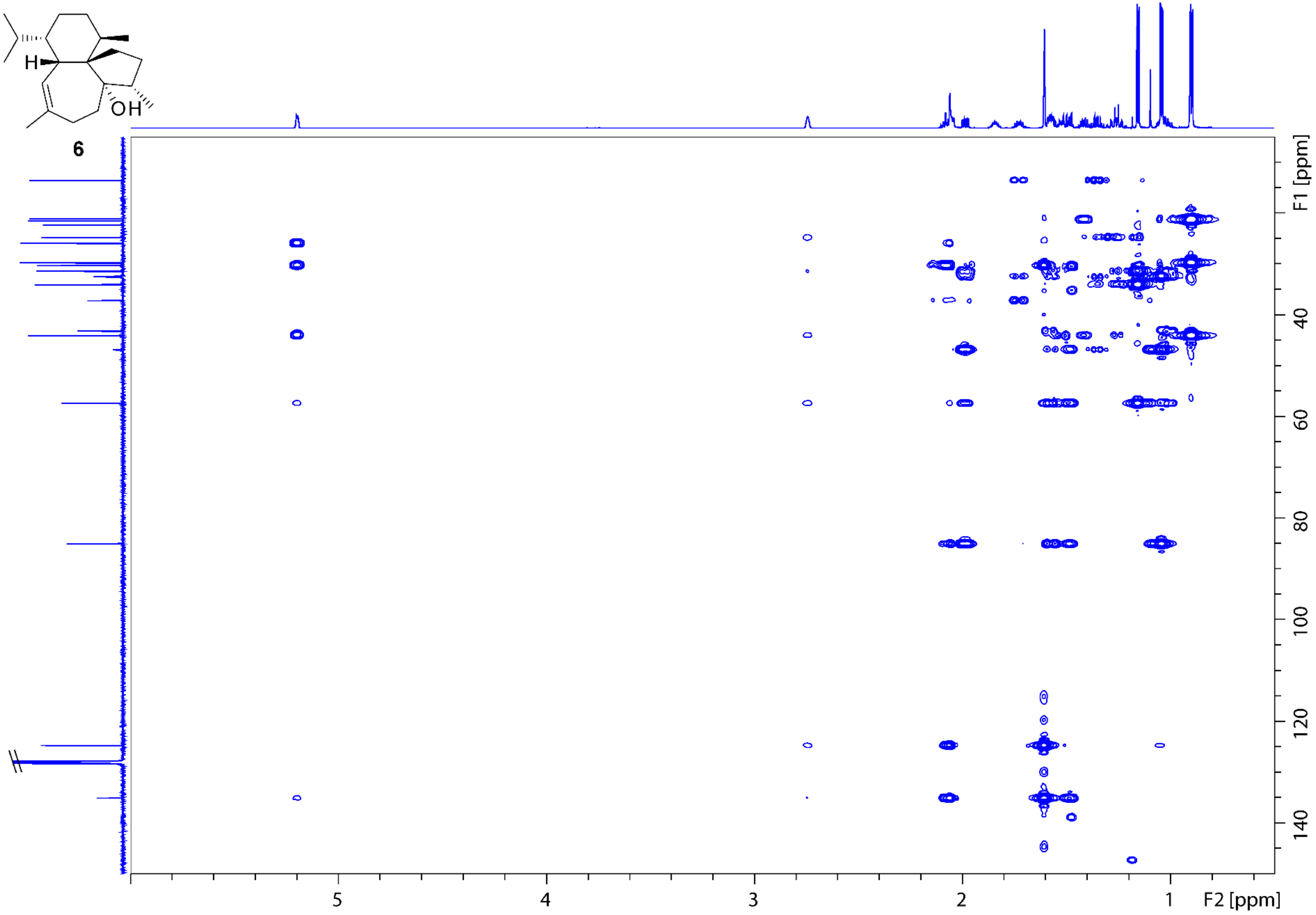

Figure S75. HMBC spectrum of **6** (C_6D_6).

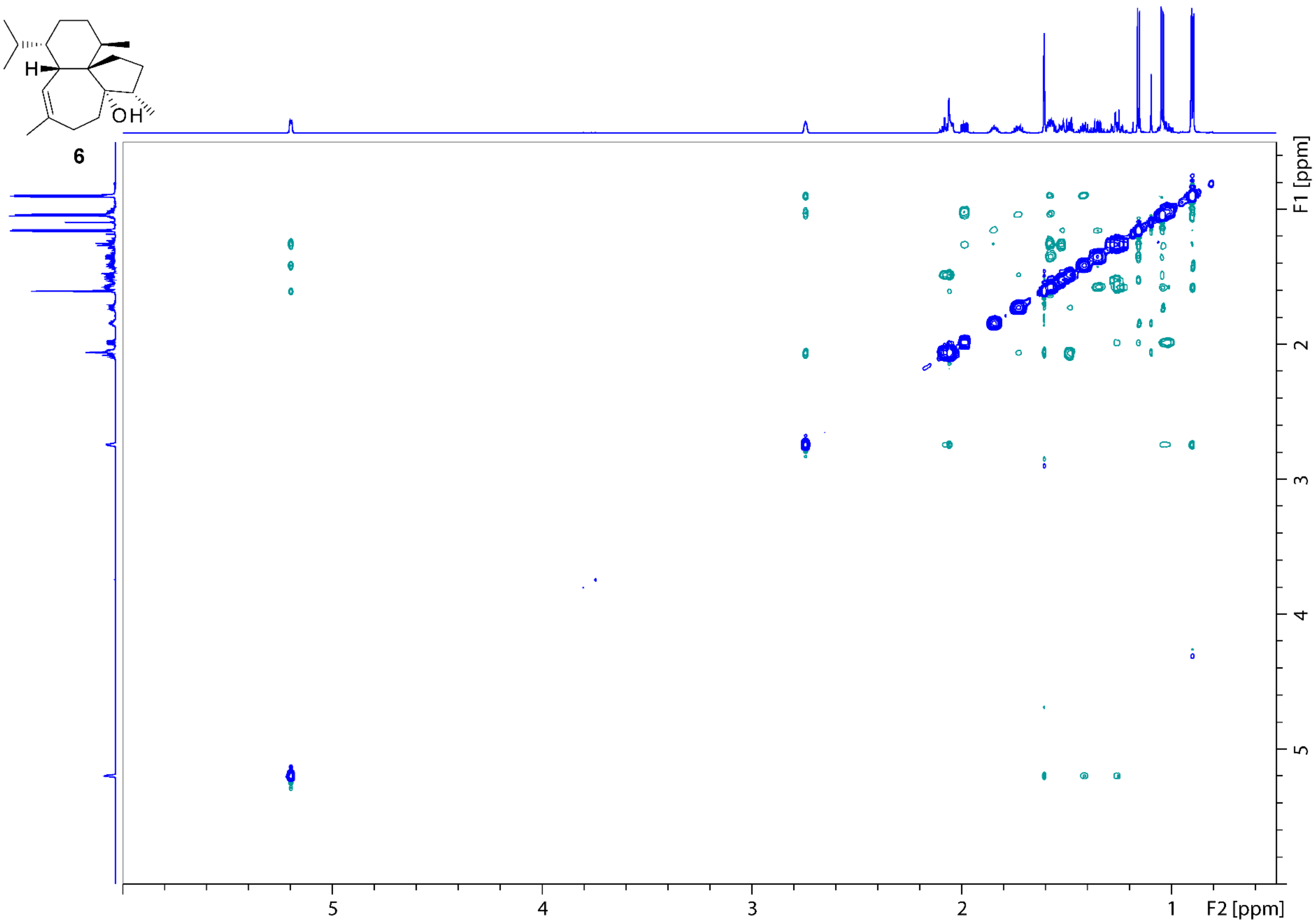

Figure S76. NOESY spectrum of **6** (C_6D_6).

Polytrichastrol B (7). Yield: 3.8 mg (13.1 μmol, 3.3%), from 200 mg (398.8 μmol) GGPP trisammonium salt. TLC (hexane/Et_2O = 5/1): R_f = 0.33. GC (HP5-MS): *I* = 2004. IR (diamond ATR): ṽ = 3608 (w), 3457 (w), 2953 (s), 2933 (s), 2864 (s), 1676 (w), 1457 (m), 1375 (m), 1345 (m), 1297 (w), 1262 (w), 1181 (w), 1169 (w), 1127 (m), 1114 (m), 1041 (w), 1015 (w), 983 (w), 939 (w), 926 (w), 907 (w), 880 (w), 846 (w), 816 (w), 745 (w), 638 (w), 543 (m), 467 (w) cm^{-1}. HR-MS (Q-TOF, 70 eV): calc. for $[C_{20}H_{34}O - HO]^+$ *m*/*z* = 273.2577; found: *m*/*z* = 273.2571. Optical rotary power: $[\alpha]_D^{20}$ = +76.1 (*c* 0.38, CH_2Cl_2).

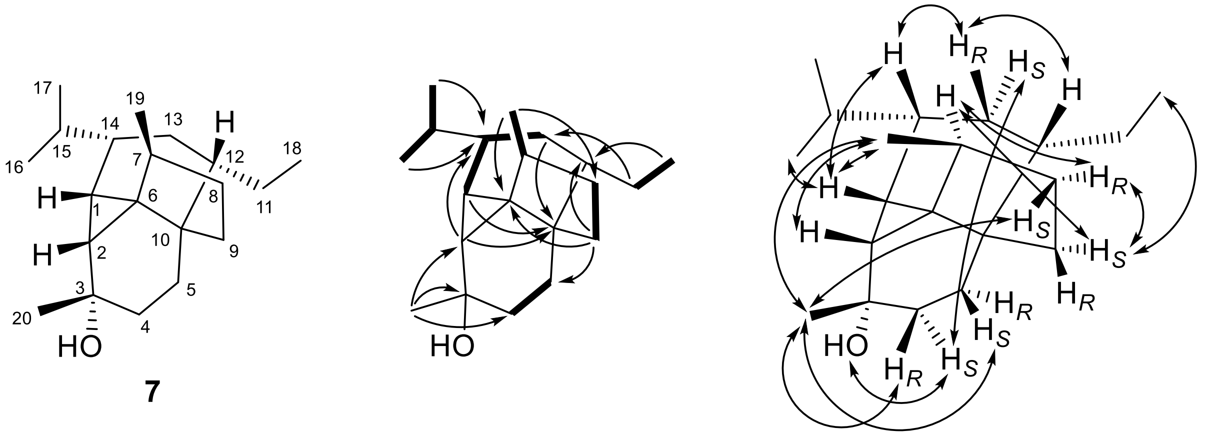

Figure S77. Structure elucidation of **7**. Bold: $^1H,^1H$-COSY, single headed arrows: key HMBC, and double headed arrows: NOESY correlations. Carbon numbering follows GGPP numbering to indicate the origin of each carbon. Diastereotopic hydrogens are labelled H_R (*pro*-*R*) and H_S (*pro*-*S*).

Table S12. NMR data of polytrichastrol B (**7**) in C_6D_6 recorded at 298 K.

C[a]	type	^{1}H[b]	^{13}C[b]
1	CH	0.76 (dd, *J* = 9.7, 6.8)	23.36
2	CH	0.78 (br d, *J* = 9.7)	29.56
3	C_q	–	69.30
4	CH_2	1.61 (m, H_S)	36.24
		1.33 (m, H_R)	
5	CH_2	1.63 (m, H_R)	31.46
		0.91 (m, H_S)	
6	C_q	–	36.31
7	CH	1.81 (m)	40.22
8	CH_2	1.69 (m, H_R)	34.03
		1.12 (dddd, *J* = 11.7, 11.7, 11.7, 6.2, H_S)	
9	CH_2	1.51 (ddd, *J* = 12.3, 6.3, 1.7, H_R)	42.86
		1.37 (ddd, *J* = 12.1, 12.1, 6.1, H_S)	
10	C_q	–	40.24
11	CH_2	1.48 (m)	24.13
		1.02 (m)	
12	CH	0.93 (m)	49.57
13	CH_2	1.83 (m, H_R)	30.98
		0.89 (m, H_S)	
14	CH	1.47 (m)	39.96
15	CH	1.69 (m)	34.93
16	CH_3	0.96 (d, *J* = 6.5)	21.77
17	CH_3	1.15 (d, *J* = 6.6)	21.83
18	CH_3	0.93 (t, *J* = 7.4)	13.63
19	CH_3	0.62 (d, *J* = 6.7)	15.58
20	CH_3	1.28 (s)	32.35
	OH	1.07 (br s)	–

[a] Carbon numbering as shown in main text. [b] Chemical shifts δ in ppm, multiplicity: s = singlet, d = doublet, t = triplet, m = multiplet, br = broad, coupling constants *J* are given in Hertz.

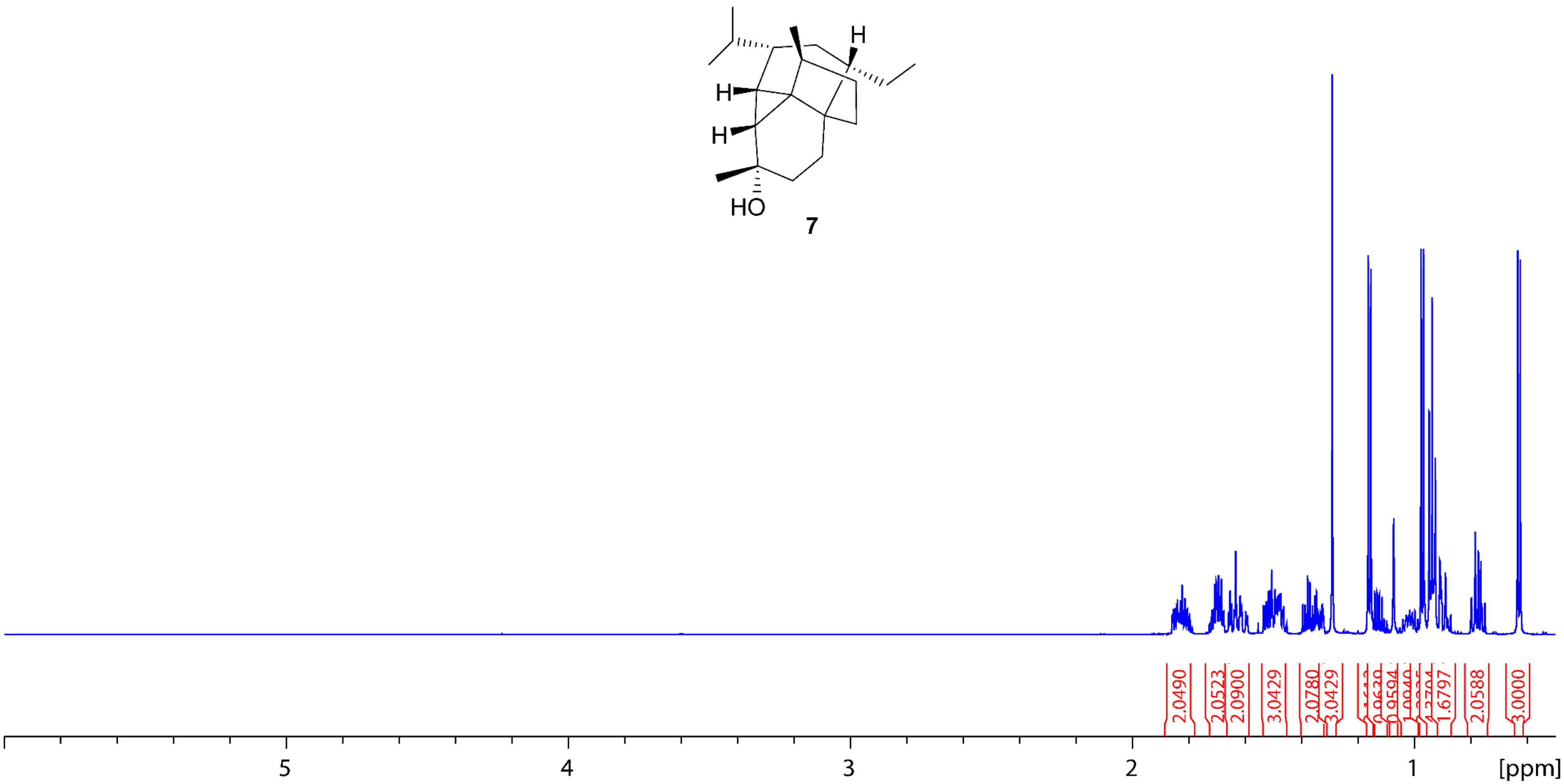

Figure S78. ^{1}H-NMR spectrum of **7** (700 MHz, C_6D_6).

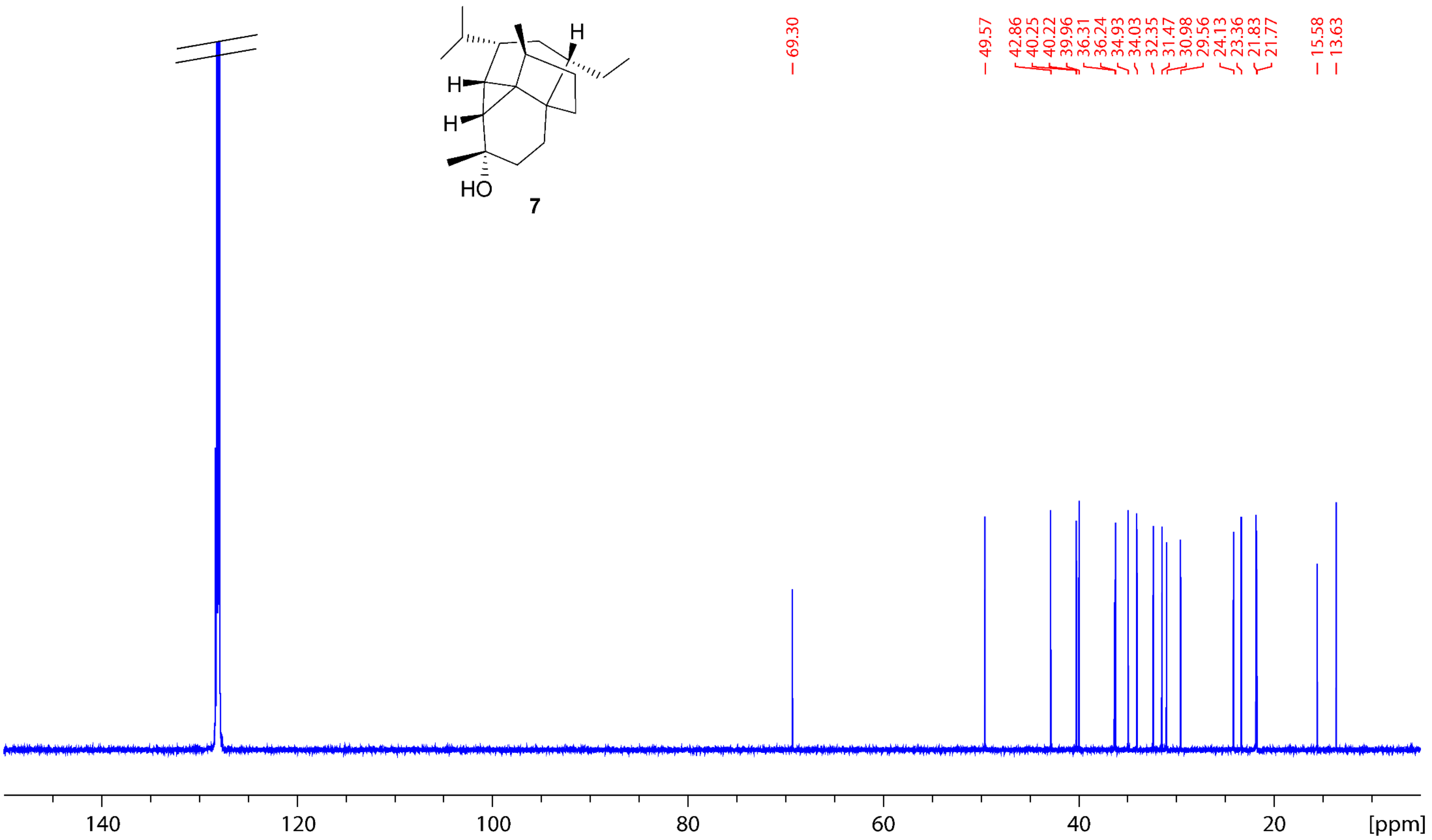

Figure S79. ^{13}C-NMR spectrum of **7** (176 MHz, C_6D_6).

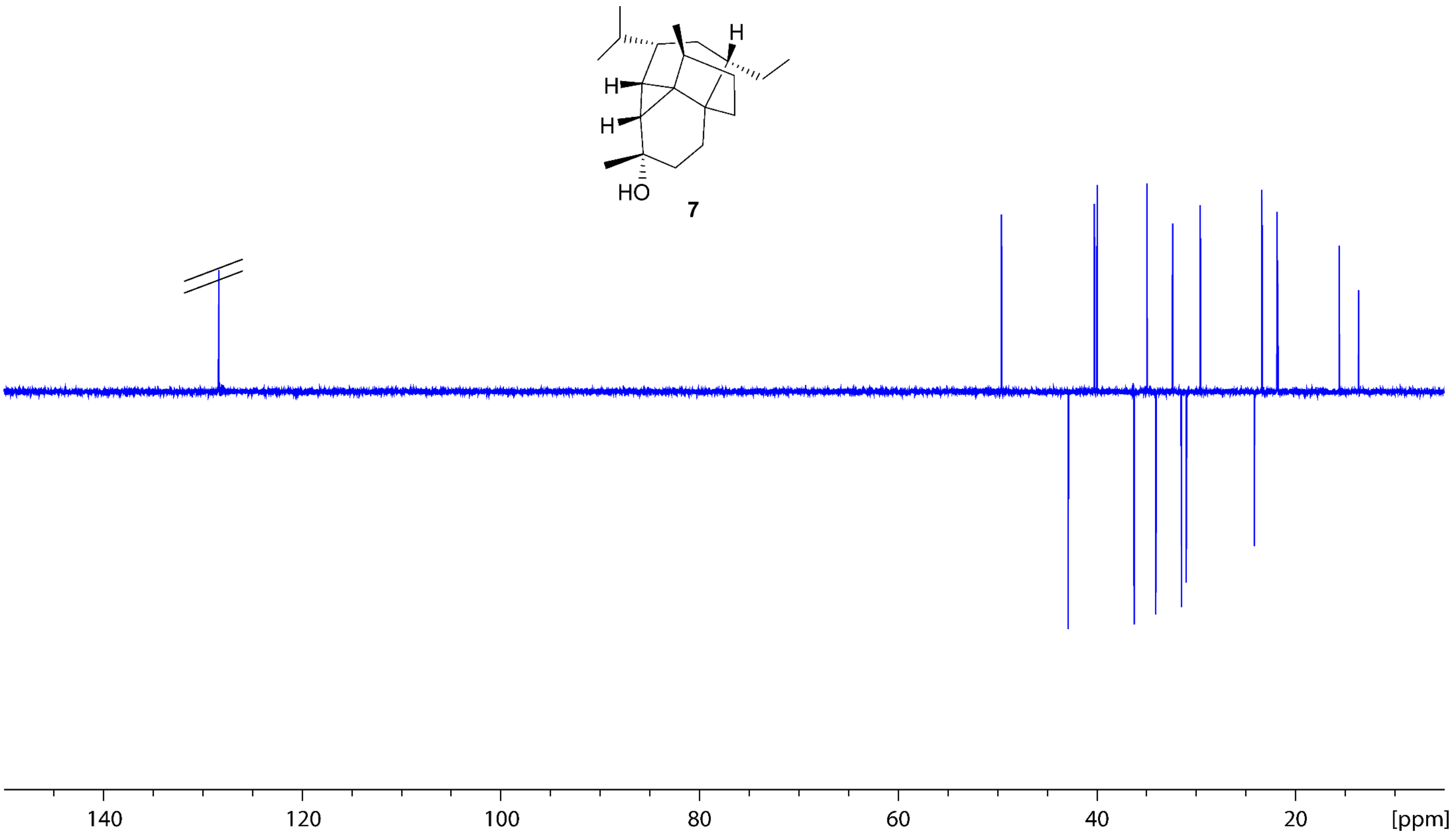

Figure S80. ^{13}C-DEPT spectrum of **7** (176 MHz, C_6D_6).

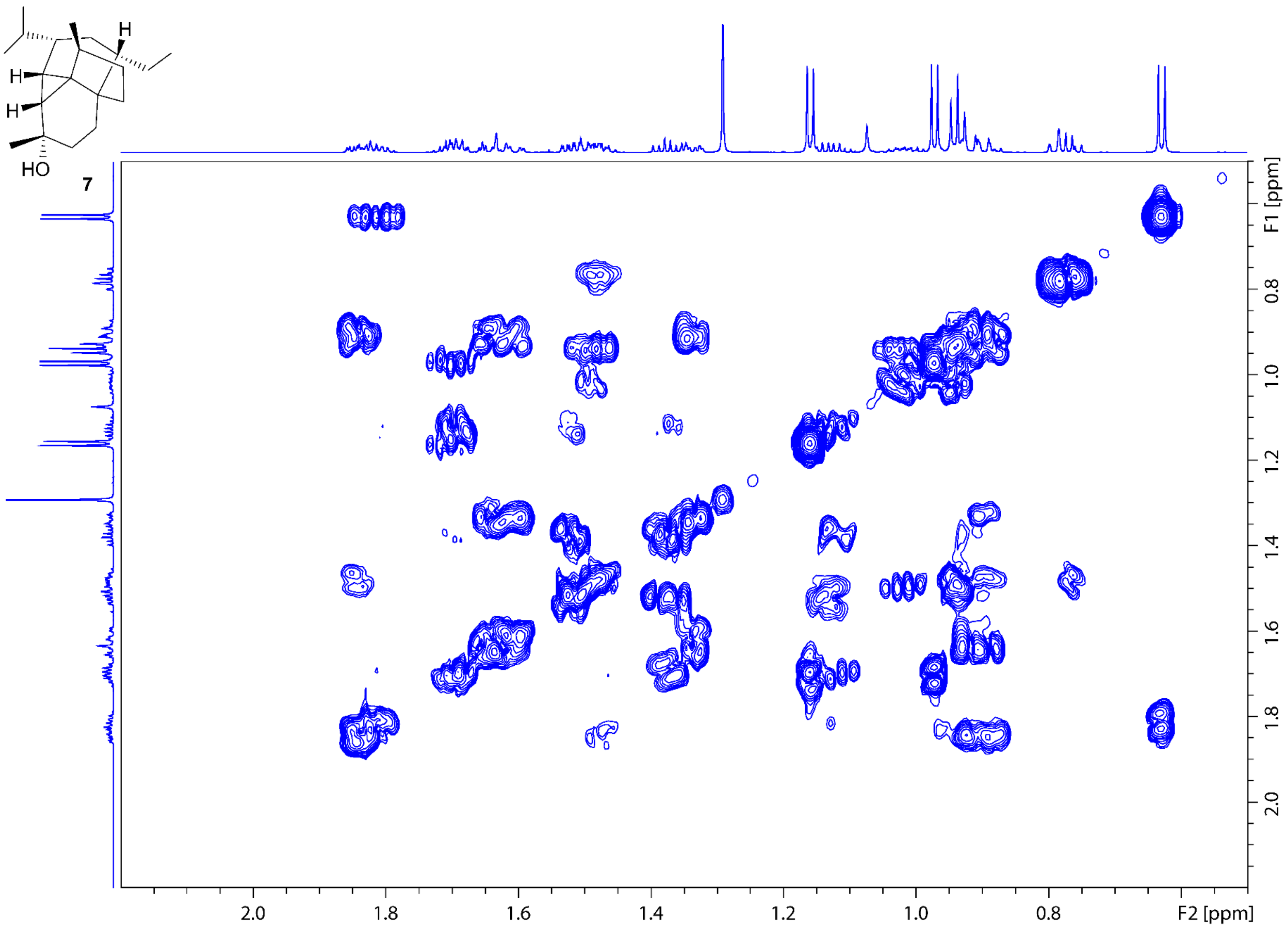

Figure S81. ^{1}H-^{1}H-COSY spectrum of **7** (700 MHz, C_6D_6).

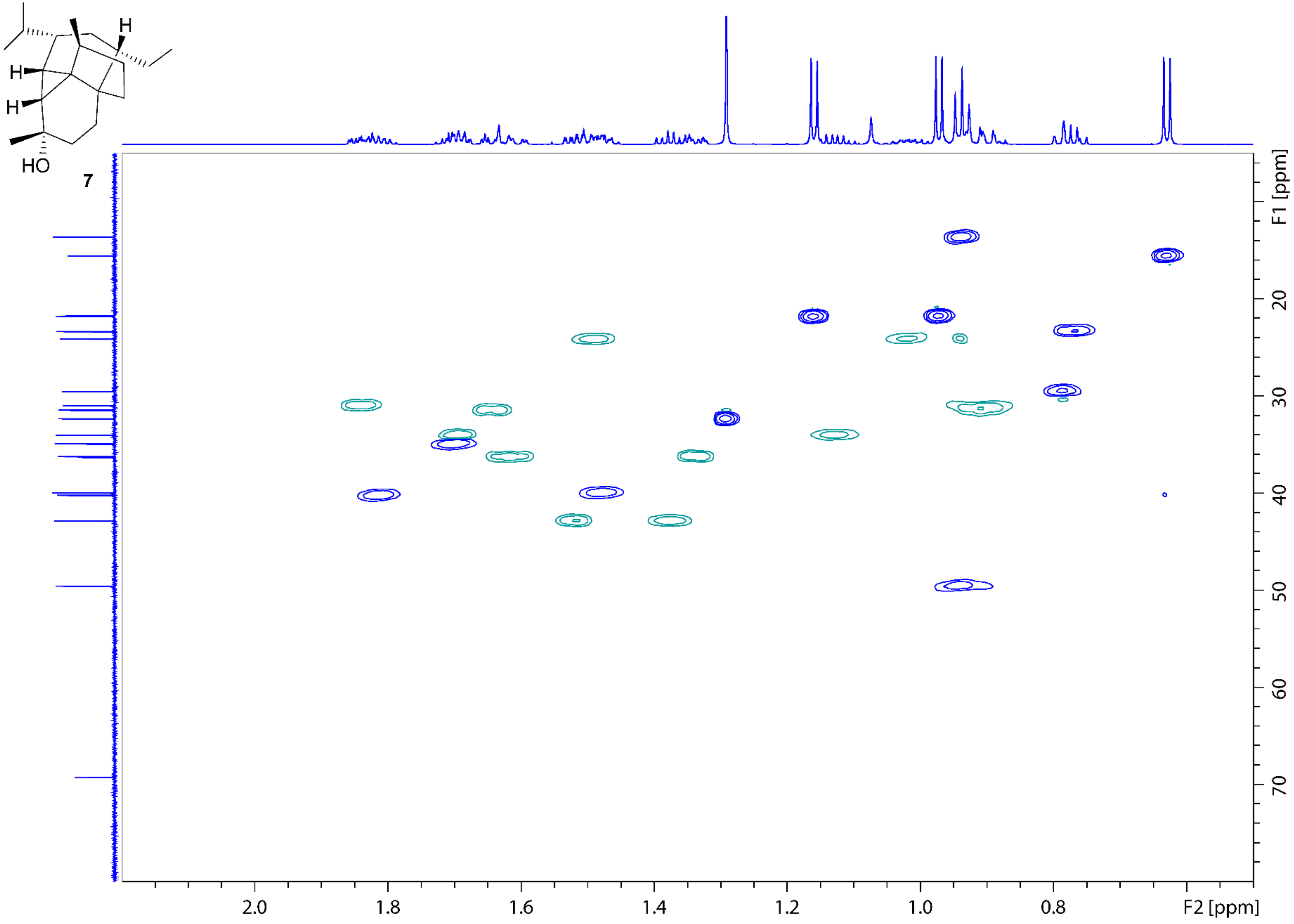

Figure S82. HSQC spectrum of **7** (C_6D_6).

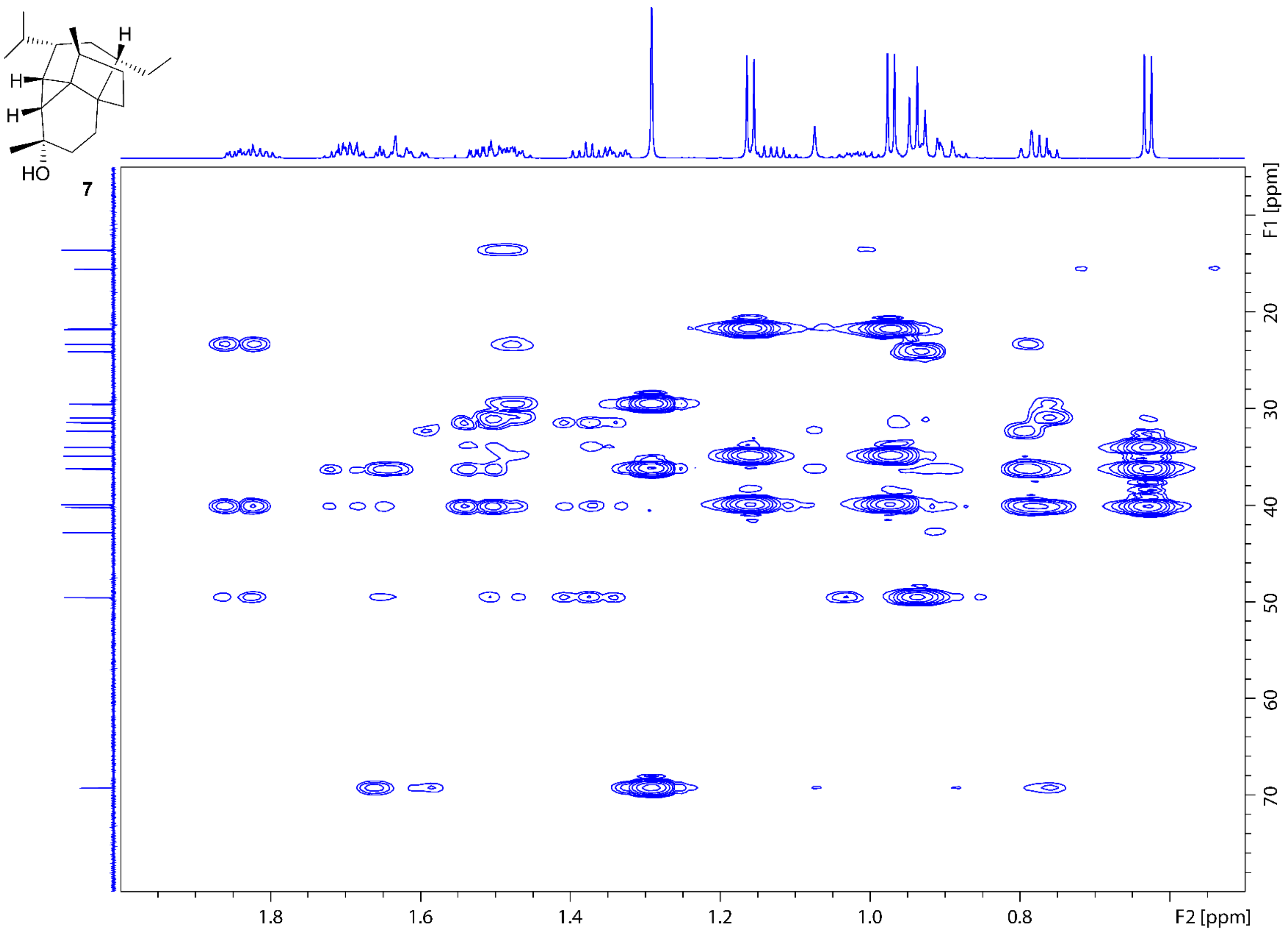

Figure S83. HMBC spectrum of **7** (C_6D_6).

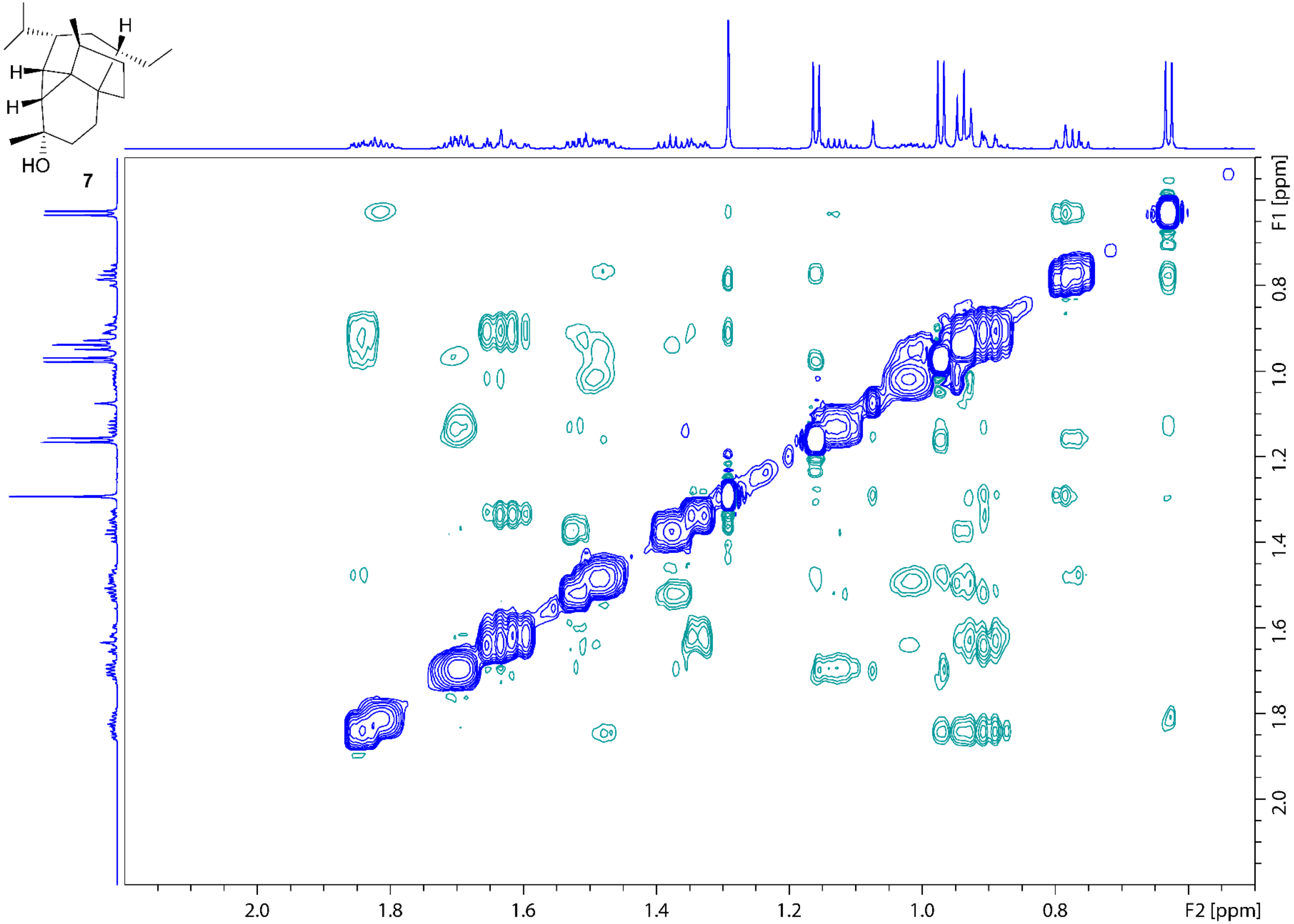

Figure S84. NOESY spectrum of **7** (C_6D_6).

Bonn-2-en-11α-ol (8). Yield: 0.3 mg (1.0 μmol, 0.3 %), from 200 mg (398.8 μmol) GGPP trisammonium salt. TLC (hexane/Et_2O = 5/1): R_f = 0.46. GC (HP5-MS): *I* = 2098. IR (diamond ATR): ṽ = 3506 (w), 2954 (m), 2925 (s), 2870 (m), 2855 (m), 2278 (w), 1729 (w), 1676 (w), 1607 (w), 1566 (w), 1510 (w), 1450 (m), 1379 (w), 1344 (w), 1315 (w), 1260 (m), 1197 (w), 1182 (w), 1086 (w), 1017 (m), 982 (w), 910 (w), 844 (w), 799 (m), 745 (w), 701 (w), 626 (w), 587 (w), 543 (s), 467 (w), 437 (w) cm^{-1}. HR-MS (Q-TOF, 70 eV): calc. for $[C_{20}H_{34}O - HO]^+$ *m*/*z* = 273.2577; found: *m*/*z* = 273.2578. Optical rotary power: $[\alpha]_D^{25}$ = +7.14 (*c* 0.03, CH_2Cl_2).

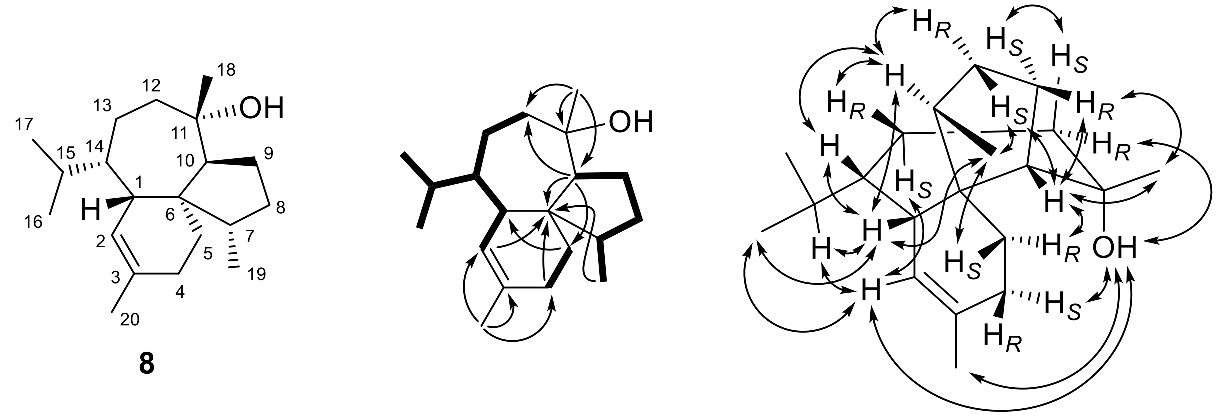

Figure S85. Structure elucidation of **8**. Bold: $^1H,^1H$-COSY, single headed arrows: key HMBC, and double headed arrows: NOESY correlations. Carbon numbering follows GGPP numbering to indicate the origin of each carbon. Diastereotopic hydrogens are labelled H_R (*pro*-*R*) and H_S (*pro*-*S*).

Table S13. NMR data of bonn-2-en-11α-ol (**8**) in C_6D_6 recorded at 298 K.

C[a]	type	^{1}H[b]	^{13}C[b]
1	CH	2.39 (br s)	41.72
2	CH	5.50 (br s)	125.86
3	C_q	–	138.25
4	CH_2	2.45 (m, H_R)	27.16
		1.69 (m, H_S)	
5	CH_2	1.27 (m, H_S)	32.71
		1.07 (ddd, J = 13.0, 5.0, 2.0, H_R)	
6	C_q	–	45.87
7	CH	1.94 (m)	41.59
8	CH_2	1.53 (m, H_R)	31.53
		1.12 (m, H_S)	
9	CH_2	1.65 (m, H_R)	31.42
		1.12 (m, H_S)	
10	C_q	1.88 (m)	60.58
11	CH_2	–	74.00
12	CH_2	1.55 (m, 2H)	38.07
13	CH_2	1.53 (m, H_S)	24.48
		1.44 (m, H_R)	
14	CH	1.27 (m)	44.65
15	CH	1.44 (m)	31.85
16	CH_3	0.86 (d, J = 6.7)	21.79
17	CH_3	0.91 (d, J = 6.7)	22.20
18	CH_3	1.23 (d, J = 1.3)	32.23
19	CH_3	0.77 (d, J = 6.9)	14.01
20	CH_3	1.66 (br)	23.74
	OH	3.41 (br s)	–

[a] Carbon numbering as shown in main text. [b] Chemical shifts δ in ppm, multiplicity: s = singlet, d = doublet, m = multiplet, br = broad, coupling constants *J* are given in Hertz.

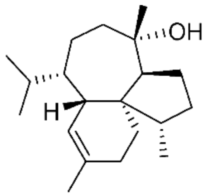

8

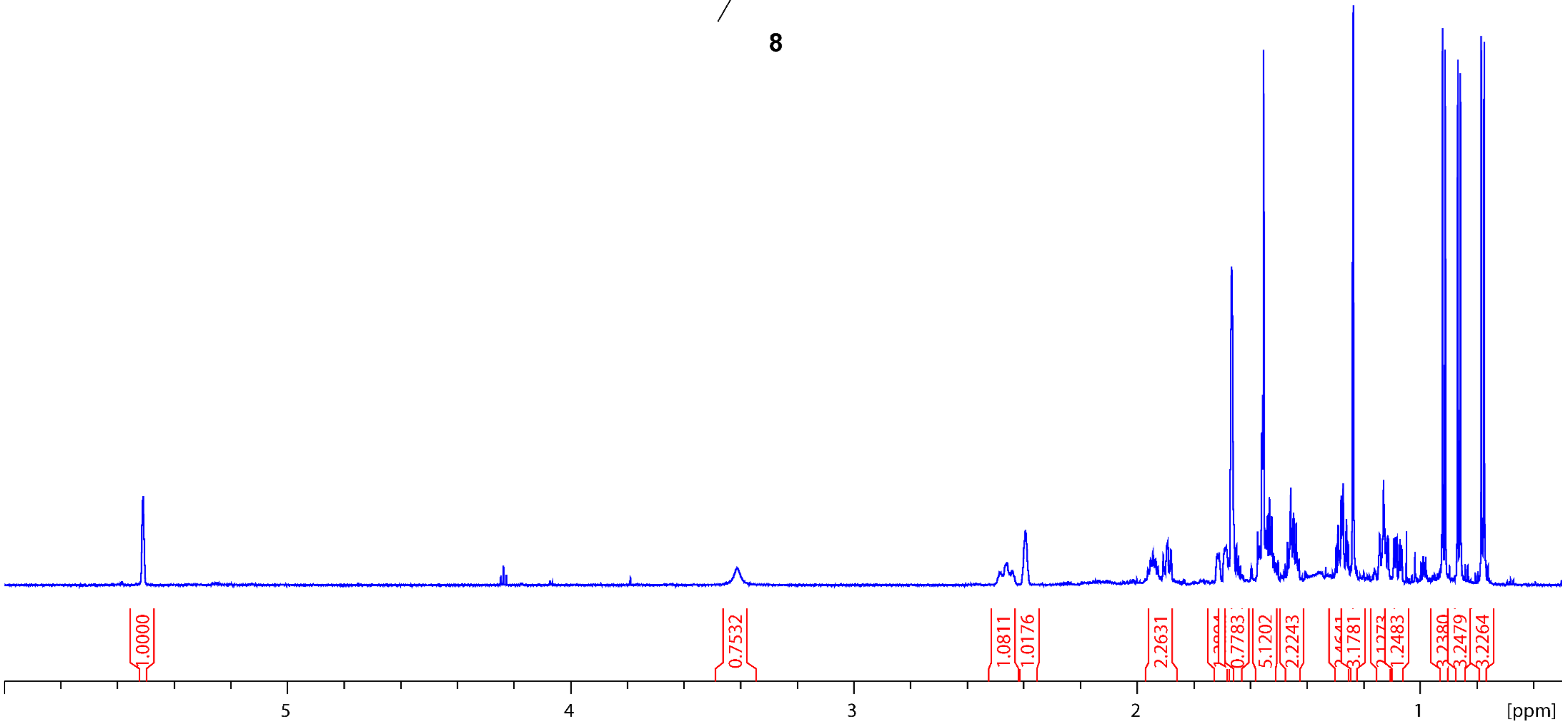

Figure S86. ^{1}H-NMR spectrum of **8** (700 MHz, C_6D_6).

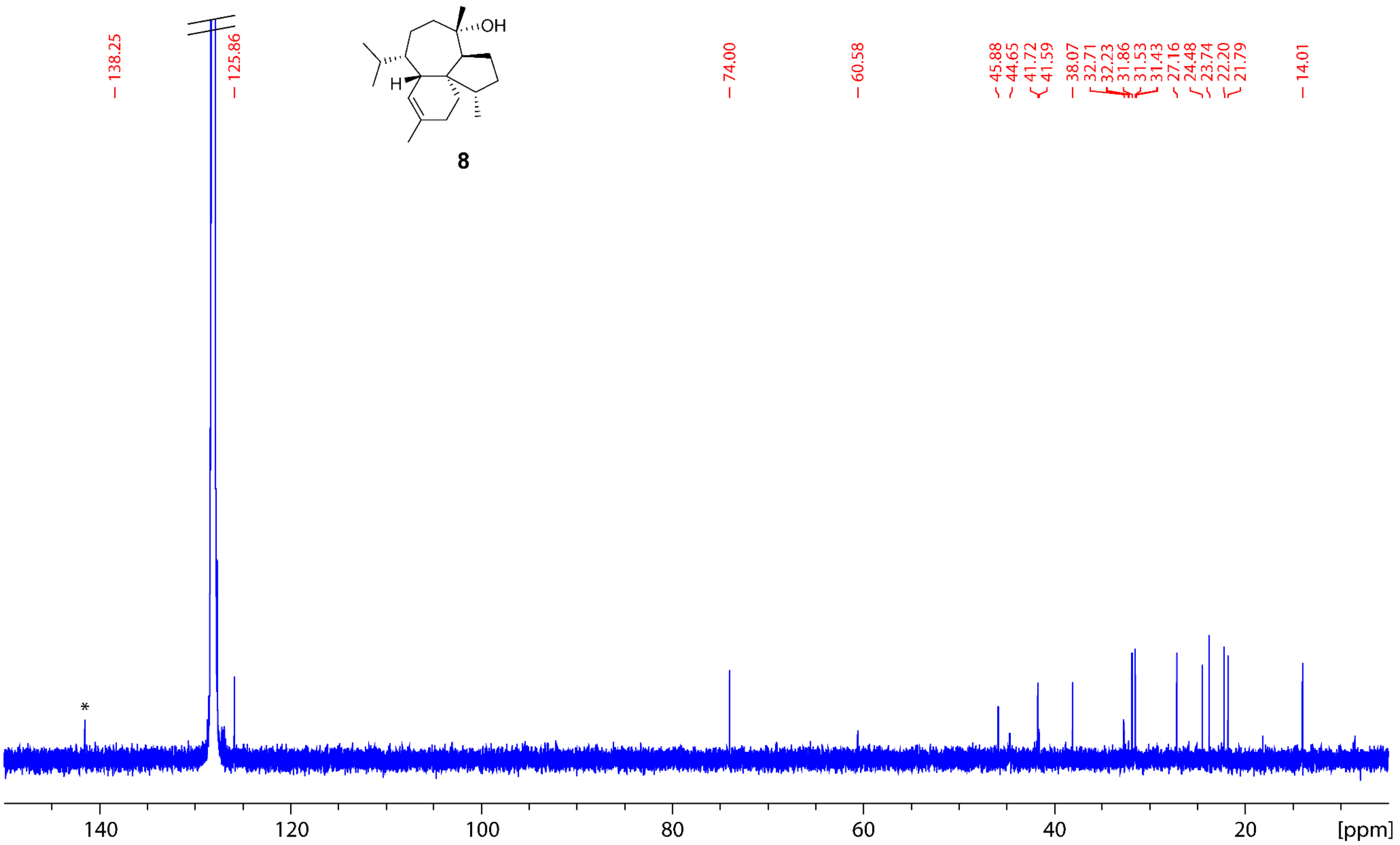

Figure S87. ^{13}C-NMR spectrum of **8** (176 MHz, C_6D_6). The asterisk indicates a peak arising from a contamination in commercial C_6D_6. The singal at 138.25 ppm is missing, likely because of line broadening, but could be inferred from the HMBC spectrum (Figure S91).

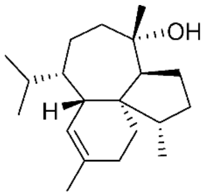

8

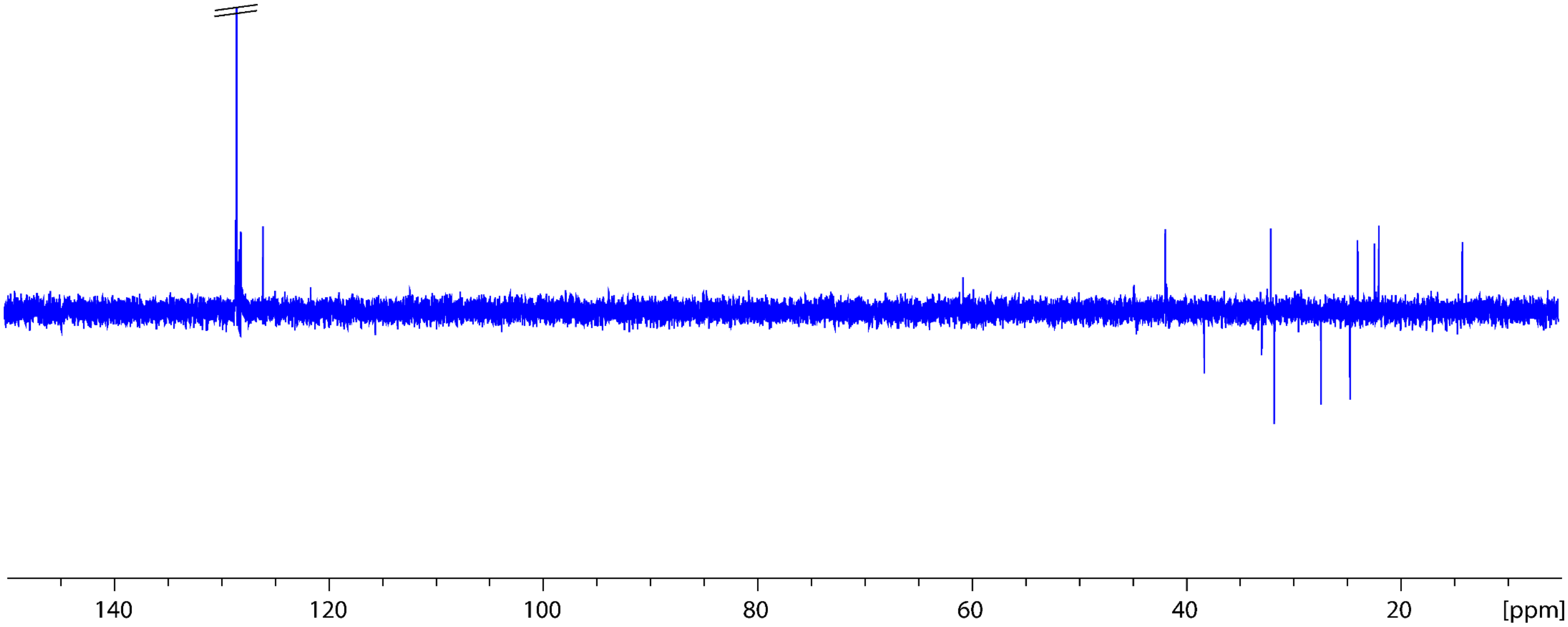

Figure S88. ^{13}C-DEPT spectrum of **8** (176 MHz, C_6D_6).

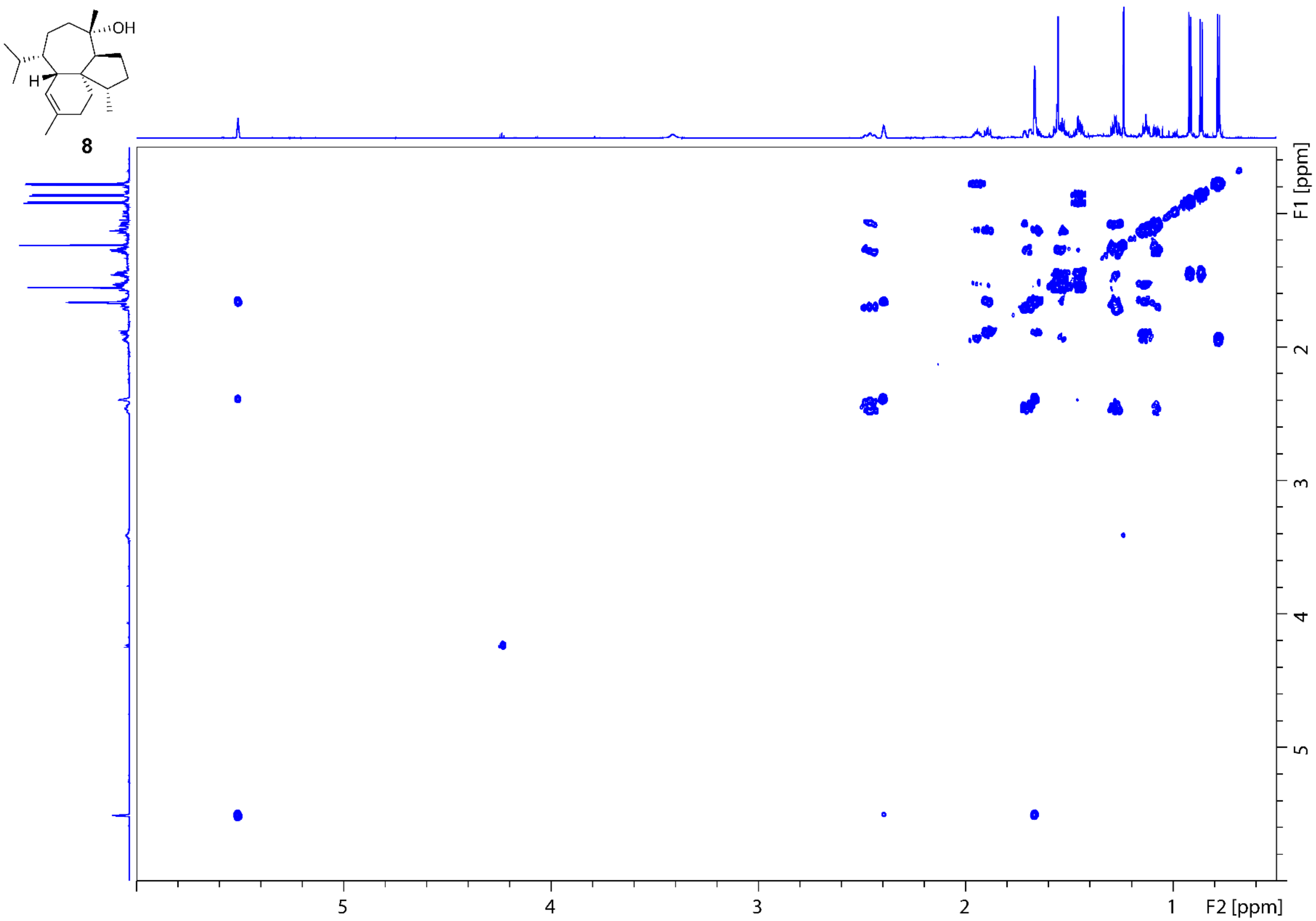

Figure S89. ^{1}H-^{1}H-COSY spectrum of **8** (700 MHz, C_6D_6).

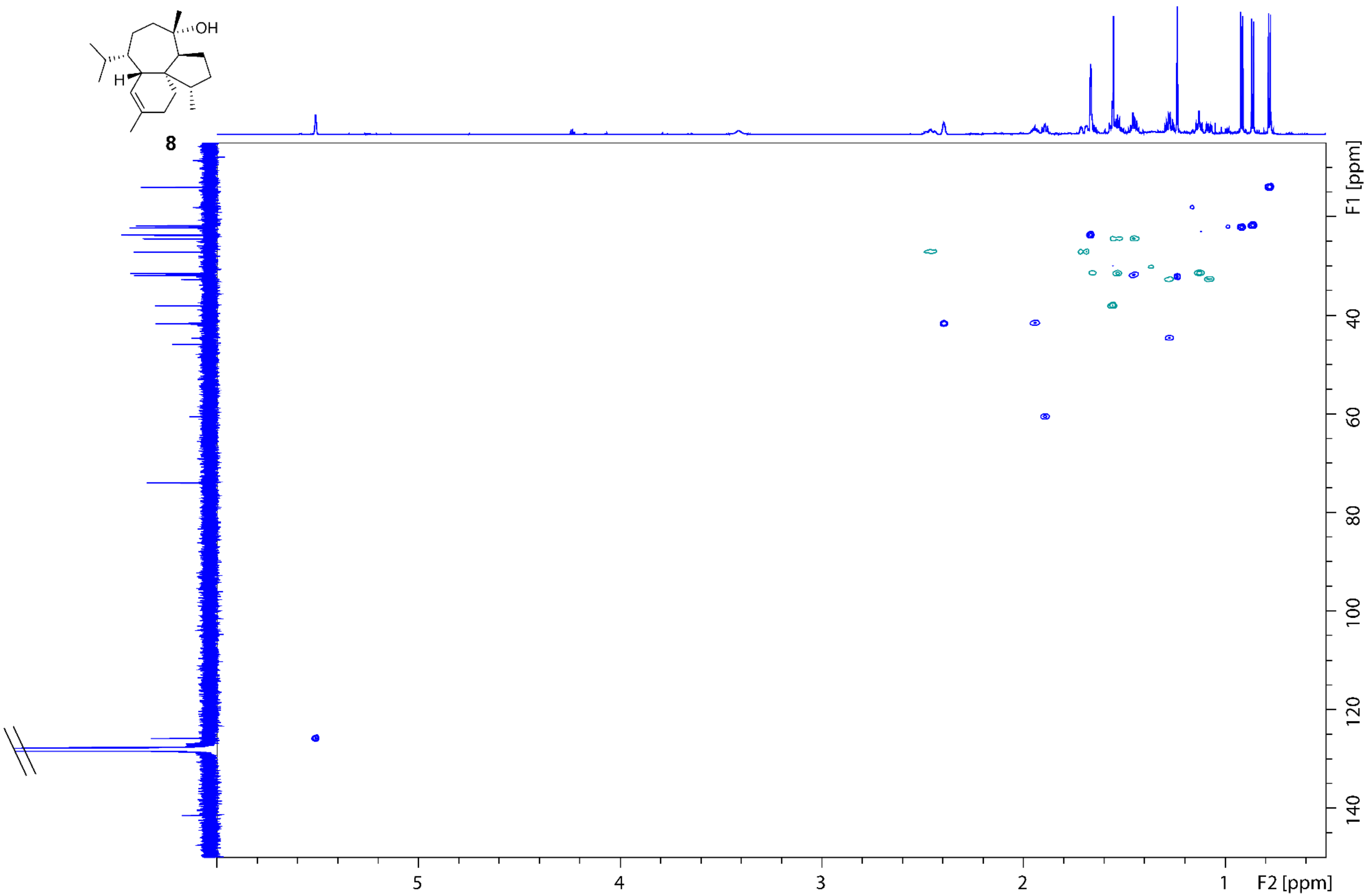

Figure S90. HSQC spectrum of **8** (C_6D_6).

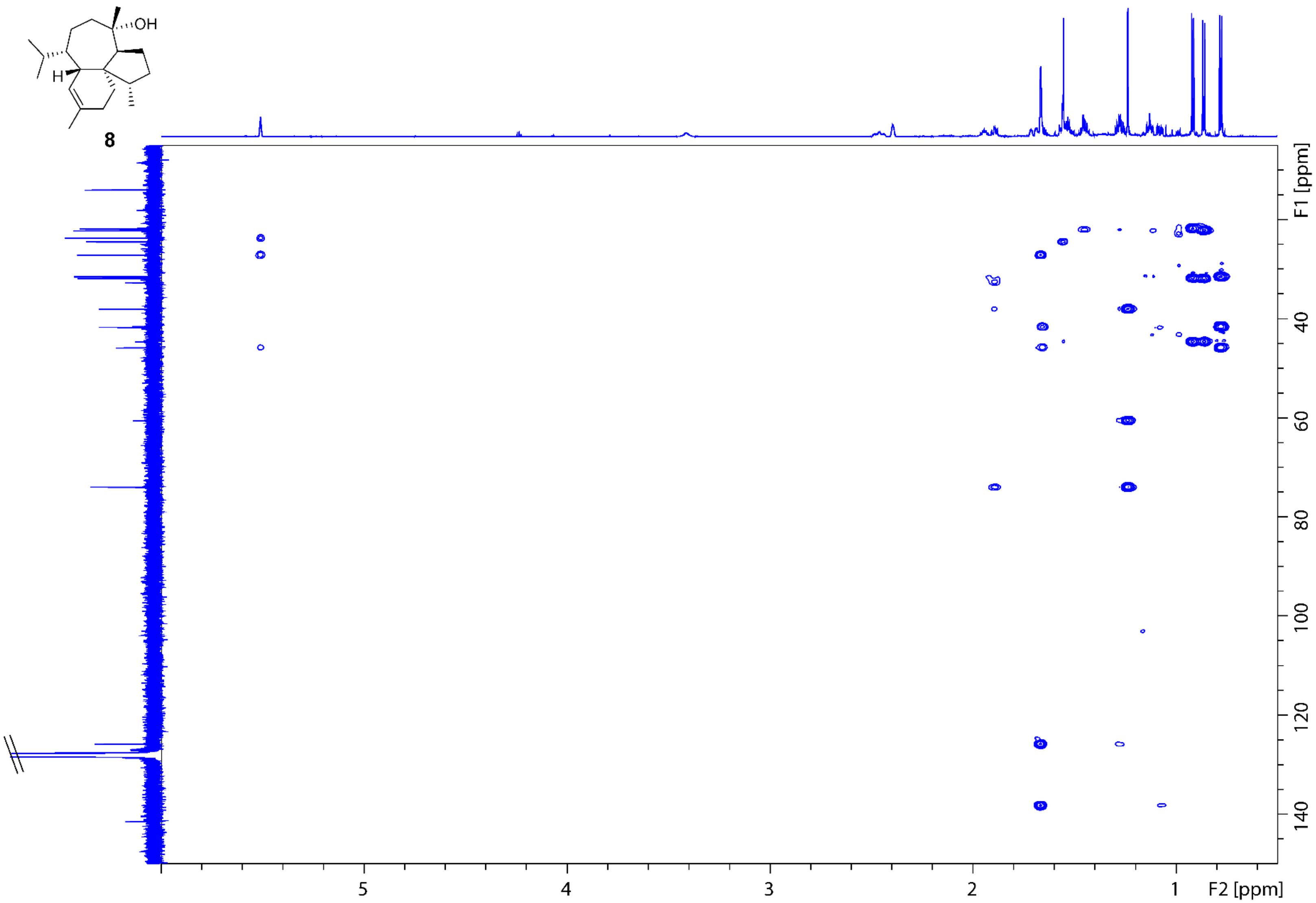

Figure S91. HMBC spectrum of **8** (C_6D_6).

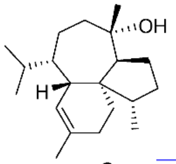

8

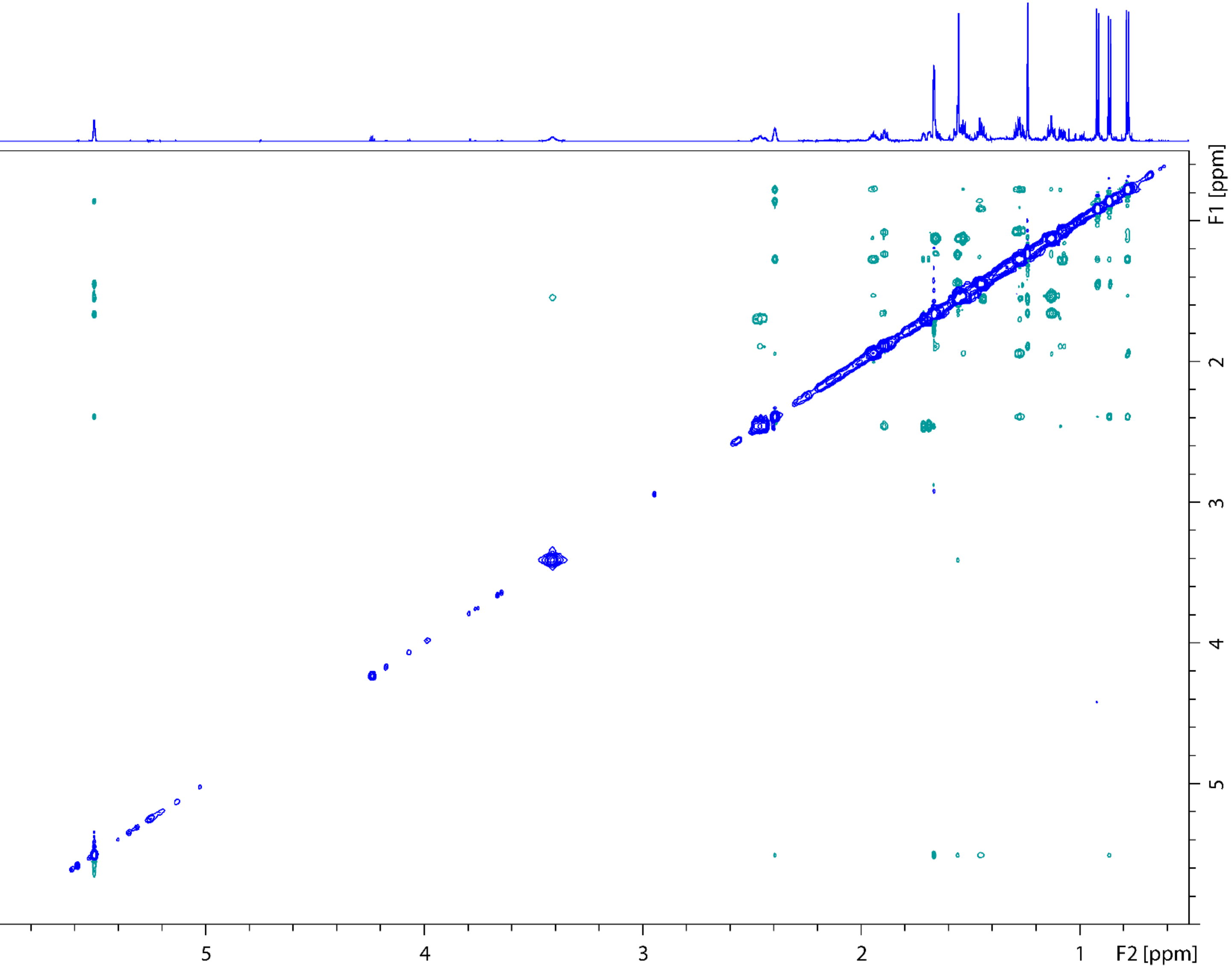

Figure S92. NOESY spectrum of **8** (C_6D_6).

Polytrichastrene B (9). Yield: 0.5 mg (1.8 μmol, 0.5 %), from 200 mg (398.8 μmol) GGPP trisammonium salt. TLC (hexane): R_f = 0.83. GC (HP5-MS): *I* = 1962. IR (diamond ATR): ṽ = 2954 (s), 2925 (s), 2869 (m), 2855 (m), 2278 (w), 1738 (w), 1674 (w), 1639 (w), 1567 (w), 1466 (w), 1374 (w), 1345 (w), 1261 (w), 1230 (w), 1094 (w), 1020 (w), 882 (w), 816 (w), 800 (w), 625 (w), 587 (w), 543 (s), 466 (w), 438 (w) cm^{-1}. HR-MS (Q-TOF, 70 eV): calc. for $[C_{20}H_{32}]^+$ *m*/*z* = 272.2499; found: *m*/*z* = 272.2499. Optical rotary power: $[\alpha]_D^{25}$ = –30.0 (*c* 0.05, CH_2Cl_2).

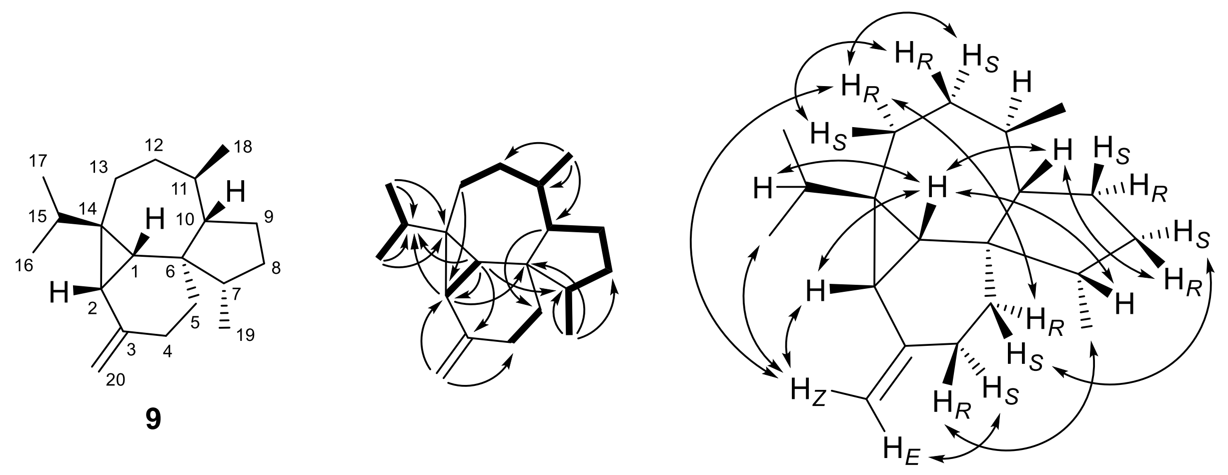

Figure S93. Structure elucidation of **9**. Bold: $^1H,^1H$-COSY, single headed arrows: key HMBC, and double headed arrows: NOESY correlations. Carbon numbering follows GGPP numbering to indicate the origin of each carbon. Diastereotopic hydrogens are labelled H_R (*pro*-*R*) and H_S (*pro*-*S*).

Table S14. NMR data of polytrichastrene B (**9**) in C_6D_6 recorded at 298 K.

C[a]	type	^{1}H[b]	^{13}C[b]
1	CH	0.56 (d, *J* = 8.8)	31.11
2	CH	1.44 (m)	27.43
3	C_q	–	144.56
4	CH_2	2.17 (m, H_R)	32.38
		2.07 (ddd, *J* = 13.8, 4.6, 3.6, H_S)	
5	CH_2	1.13 (m, 2H)	21.93
6	C_q	–	42.06
7	CH	1.73 (m)	51.19
8	CH_2	1.65 (m, H_R)	29.28
		1.20 (m, H_S)	
9	CH_2	1.70 (m, H_S)	28.31
		1.04 (m, H_R)	
10	CH	1.36 (m)	58.94
11	CH	1.30 (m)	36.53
12	CH_2	1.45 (m, H_S)	36.59
		1.36 (m, H_R)	
13	CH_2	1.73 (m, H_S)	25.88
		1.18 (m, H_R)	
14	C_q	–	31.35
15	CH	1.07 (m)	37.48
16	CH_3	0.83 (d, *J* = 6.9)	19.19
17	CH_3	1.01 (d, *J* = 6.9)	21.38
18	CH_3	0.81 (d, *J* = 6.0)	22.64
19	CH_3	1.00 (d, *J* = 7.0)	16.33
20	CH_2	5.14 (q, *J* = 2.3, H_E)	111.28
		4.94 (q, *J* = 2.2, H_E)	

[a] Carbon numbering as shown in main text. [b] Chemical shifts δ in ppm, multiplicity: s = singlet, d = doublet, q = quartet, m = multiplet, coupling constants *J* are given in Hertz.

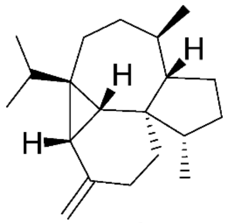

9

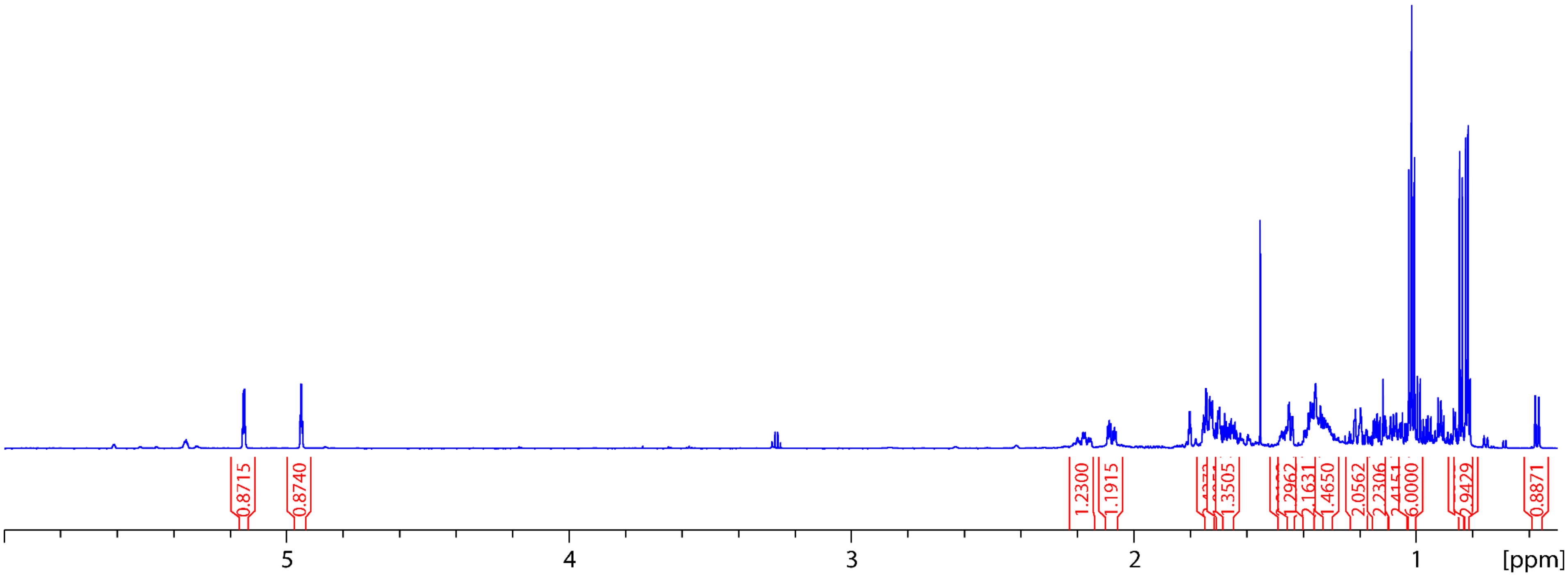

Figure S94. ^{1}H-NMR spectrum of **9** (700 MHz, C_6D_6).

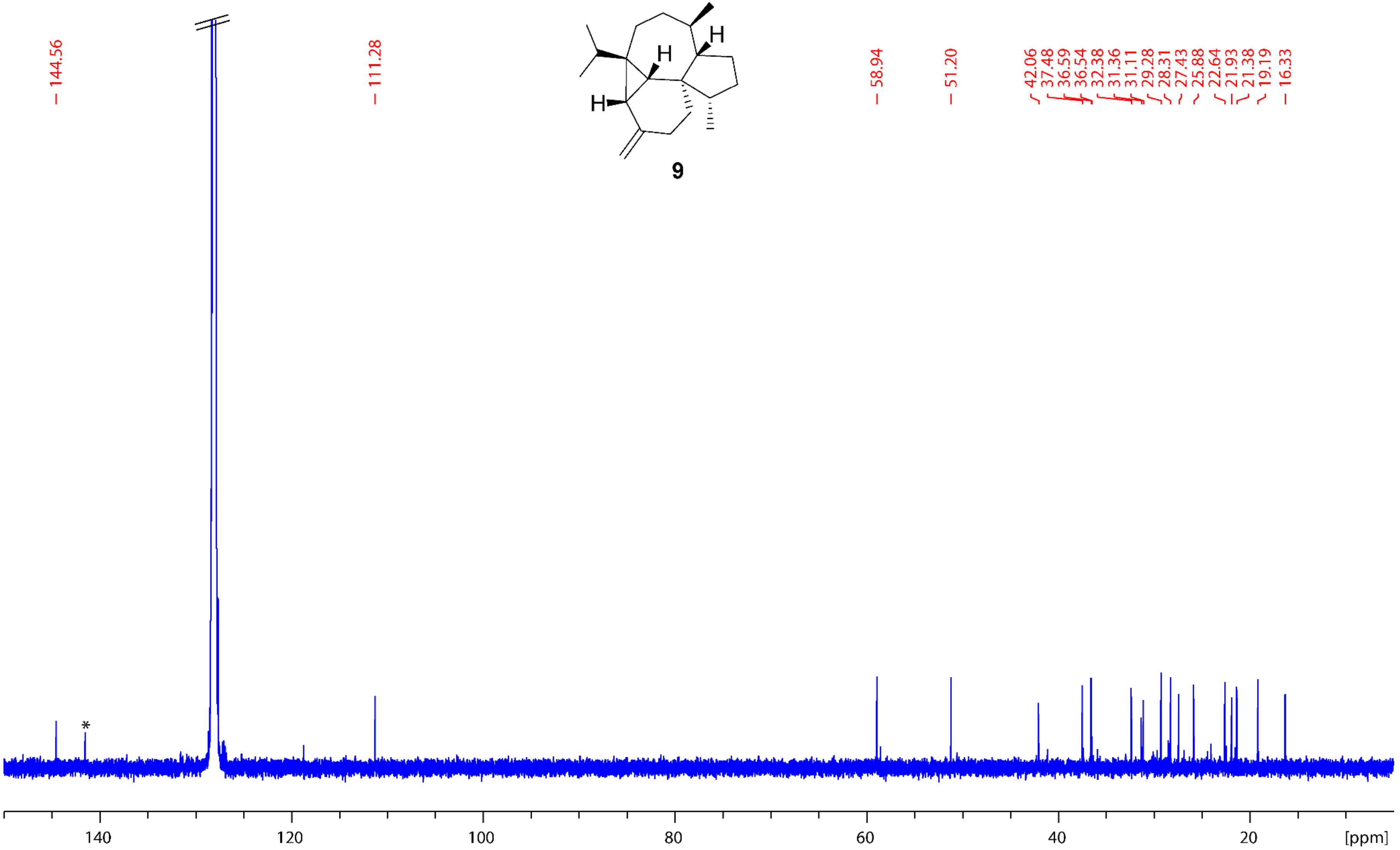

Figure S95. ^{13}C-NMR spectrum of **9** (176 MHz, C_6D_6). The asterisk indicates a peak arising from a contamination in commercial C_6D_6.

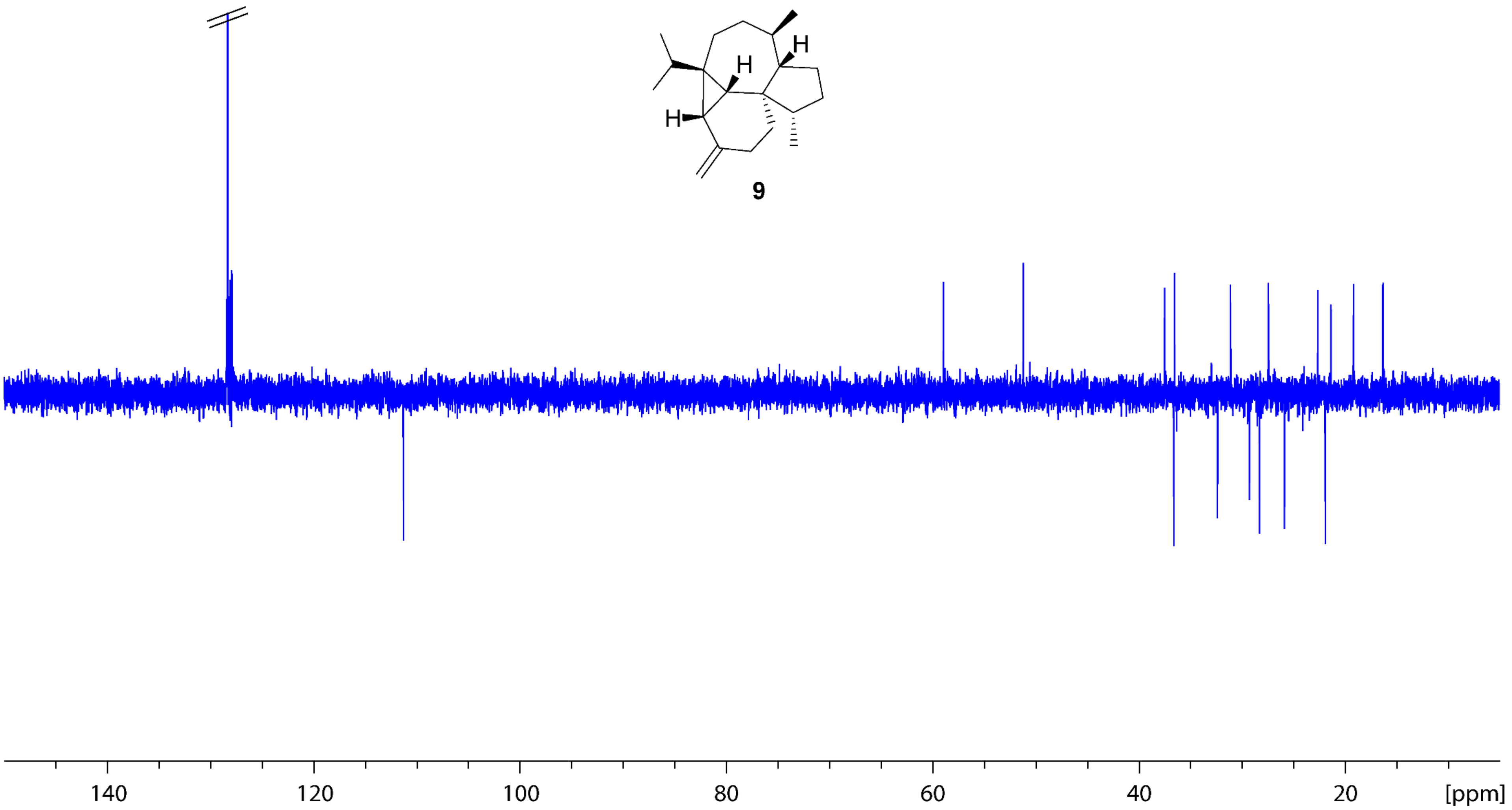

Figure S96. ^{13}C-DEPT spectrum of **9** (176 MHz, C_6D_6).

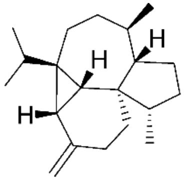

9

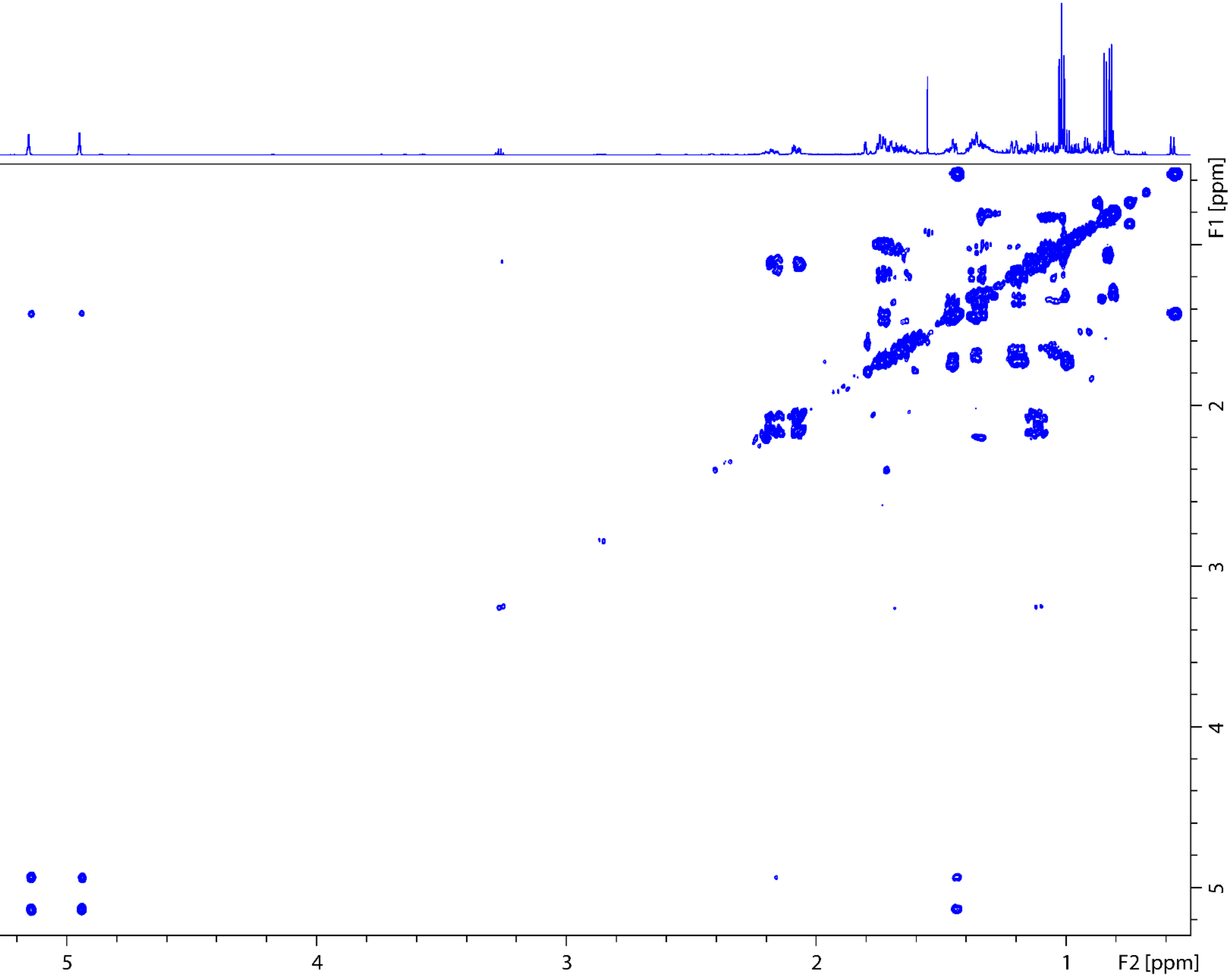

Figure S97. ^{1}H-^{1}H-COSY spectrum of **9** (700 MHz, C_6D_6).

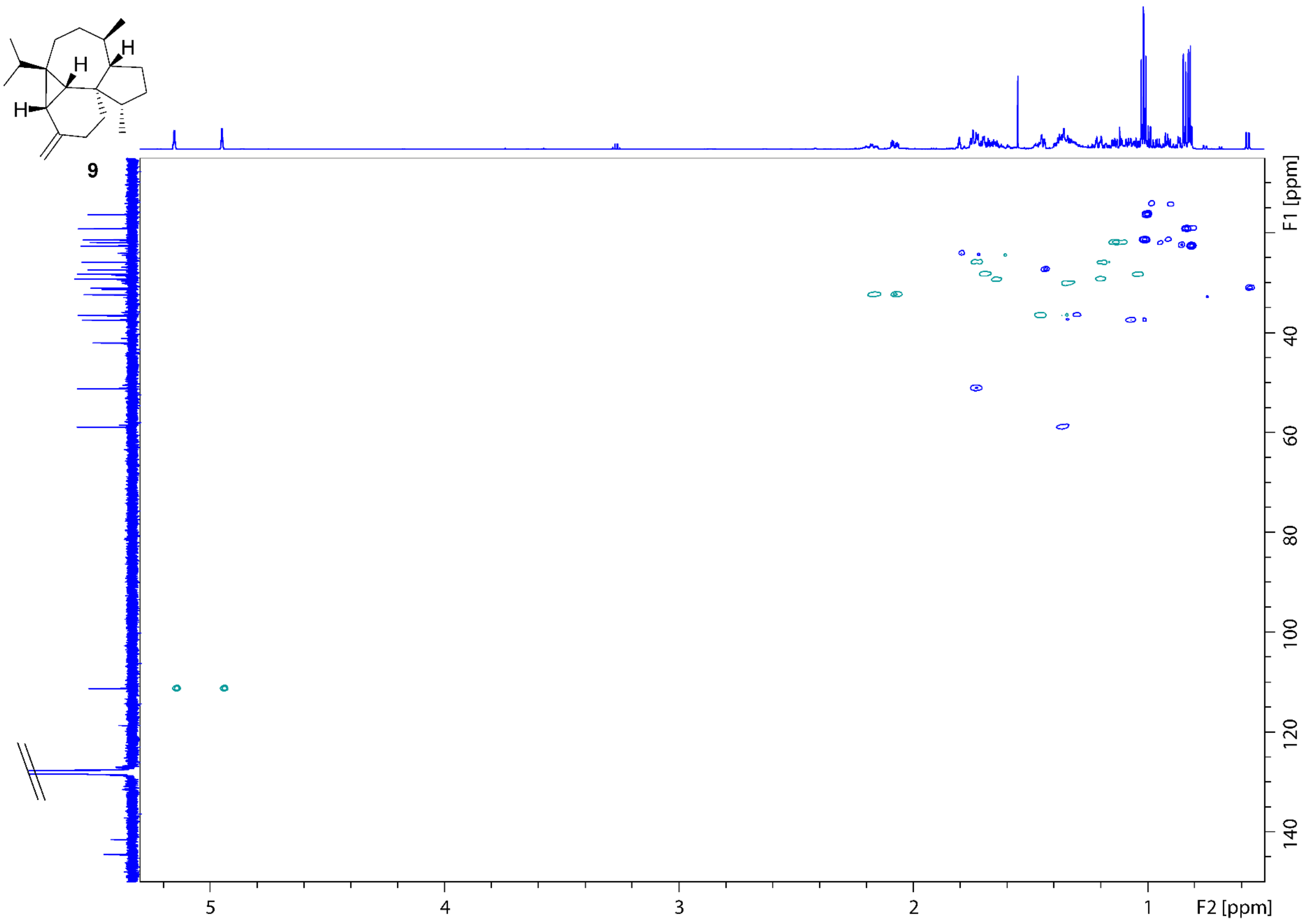

Figure S98. HSQC spectrum of **9** (C_6D_6).

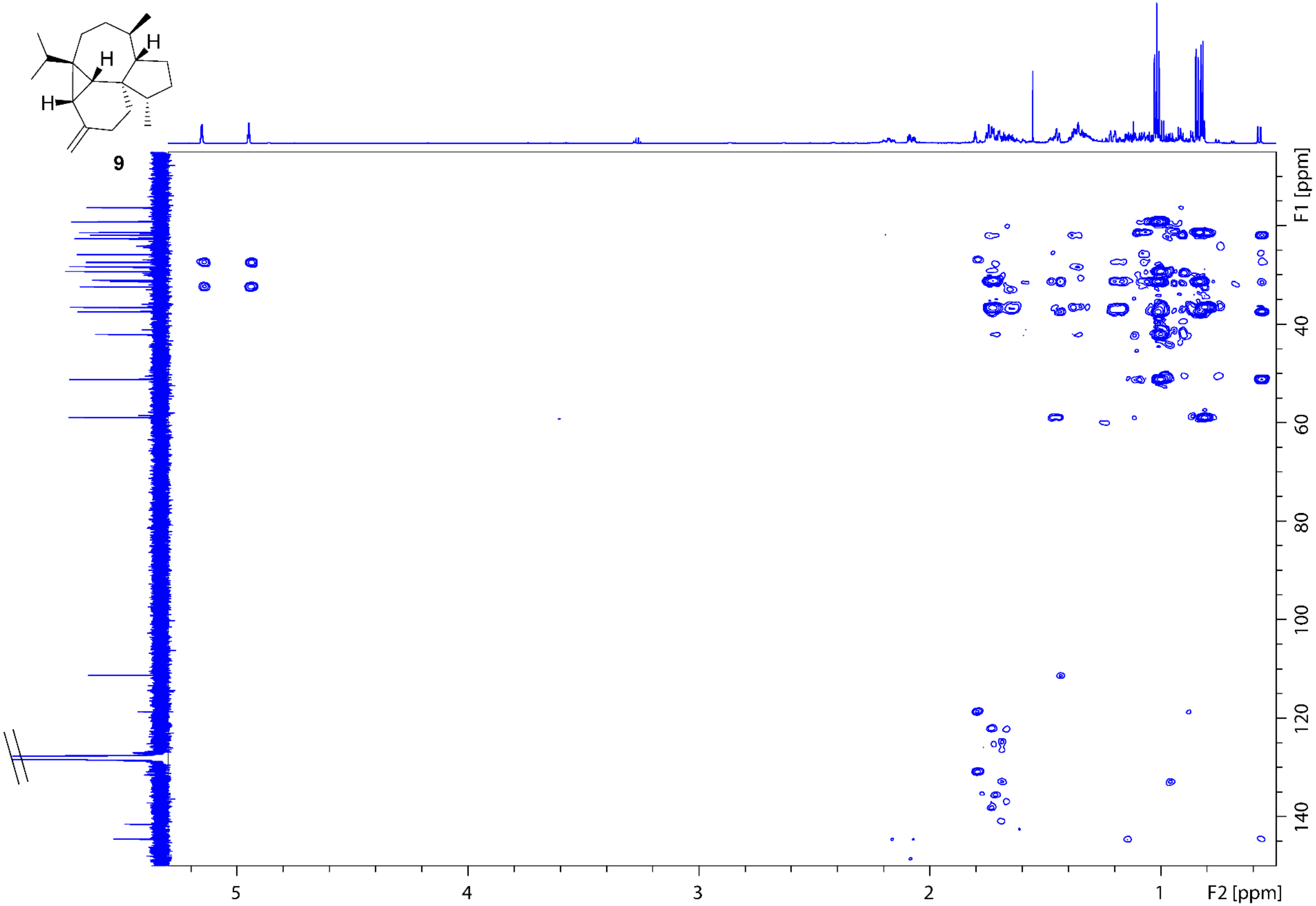

Figure S99. HMBC spectrum of **9** (C_6D_6).

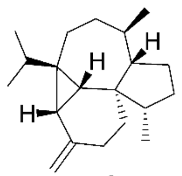

9

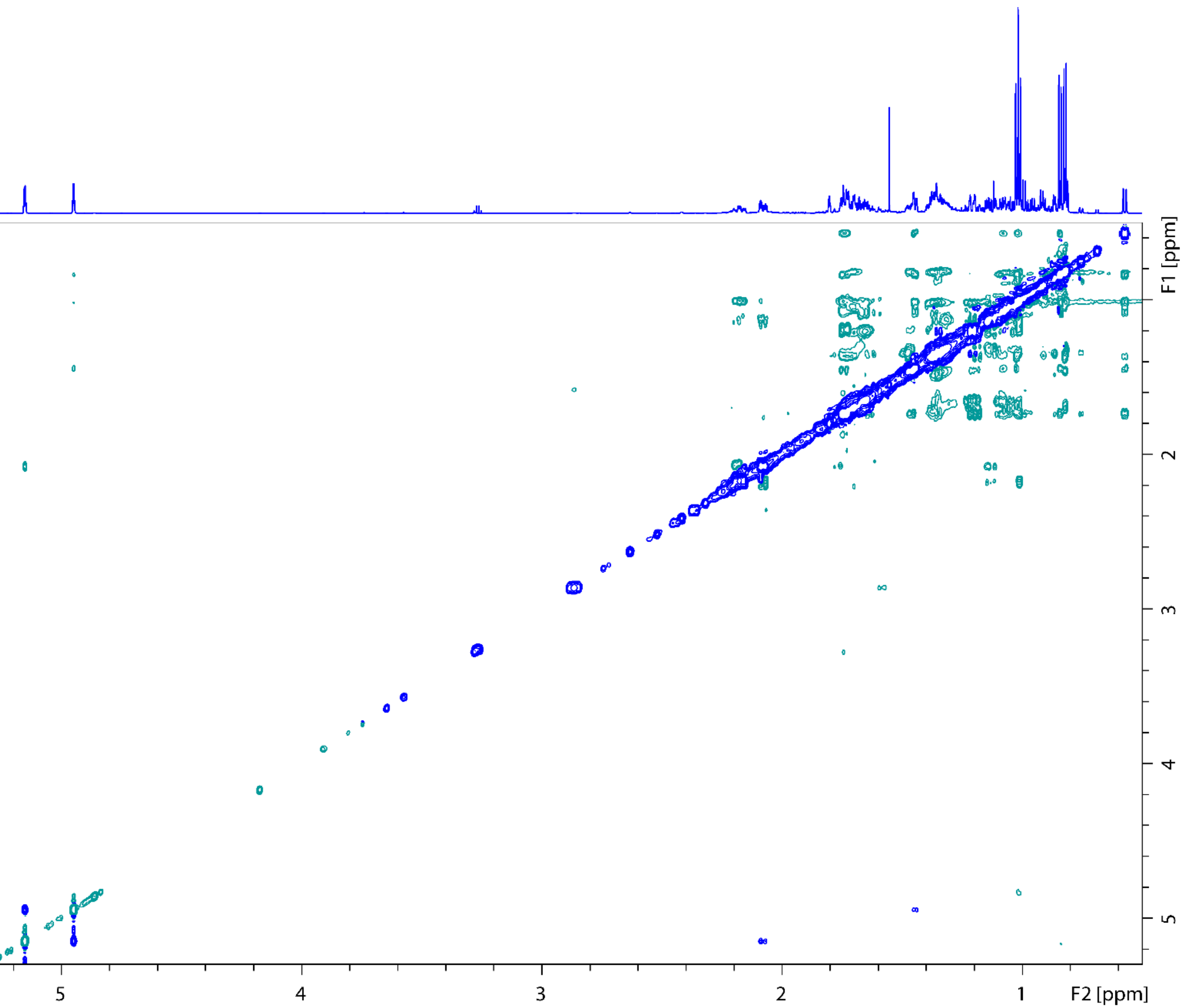

Figure S100. NOESY spectrum of **9** (C_6D_6).

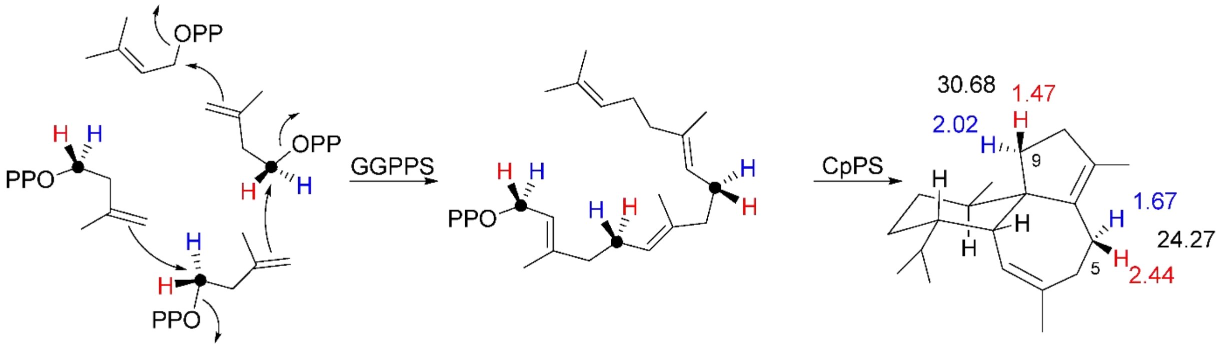

A) unlabeled **5**

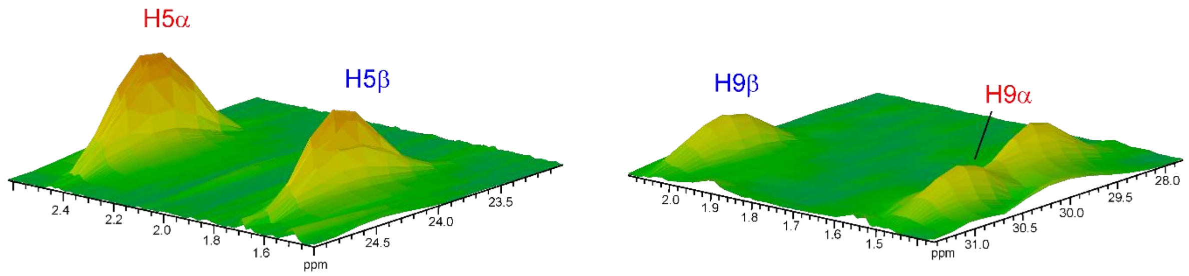

B) (*R*)-(1-^{13}C,1-^{2}H)IPP

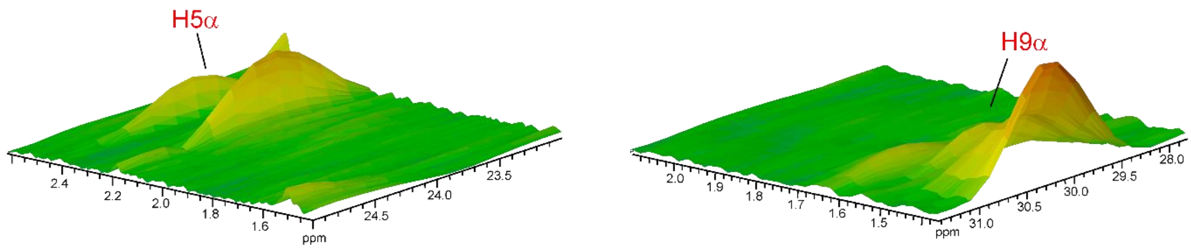

C) (*S*)-(1-^{13}C,1-^{2}H)IPP

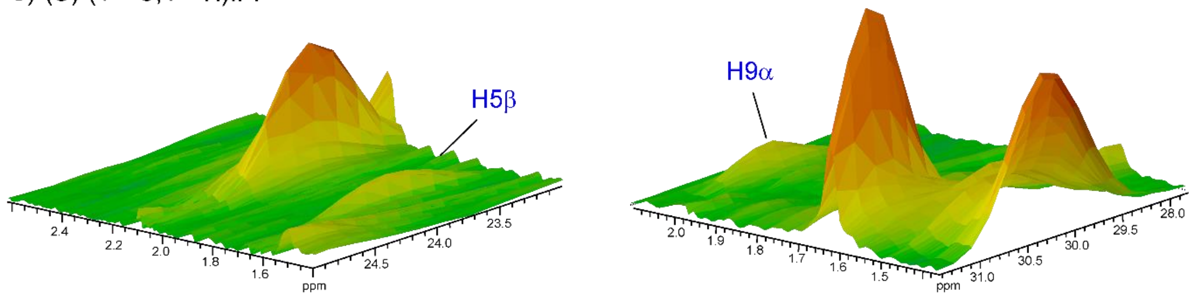

Figure S101. The absolute configuration of **5**. Partial HSQC spectra of A) unlabeled **5**, B) labeled **5** obtained from DMAPP and (*R*)-(1-^{13}C,1-^{2}H)IPP (blue H = ^{2}H), and C) labeled **1** from DMAPP and (*S*)-(1-^{13}C,1-^{2}H)IPP (red H = ^{2}H). The specific incorporation at C5 and C9 with known configuration at this carbon in experiments B) and C) together with the NOESY based assignments of relative orientations of H5α, H5β, H9α and H9β (Figure S61) with respect to the naturally present stereogenic centers in **5** allows to assign the shown absolute configuration for **5**.

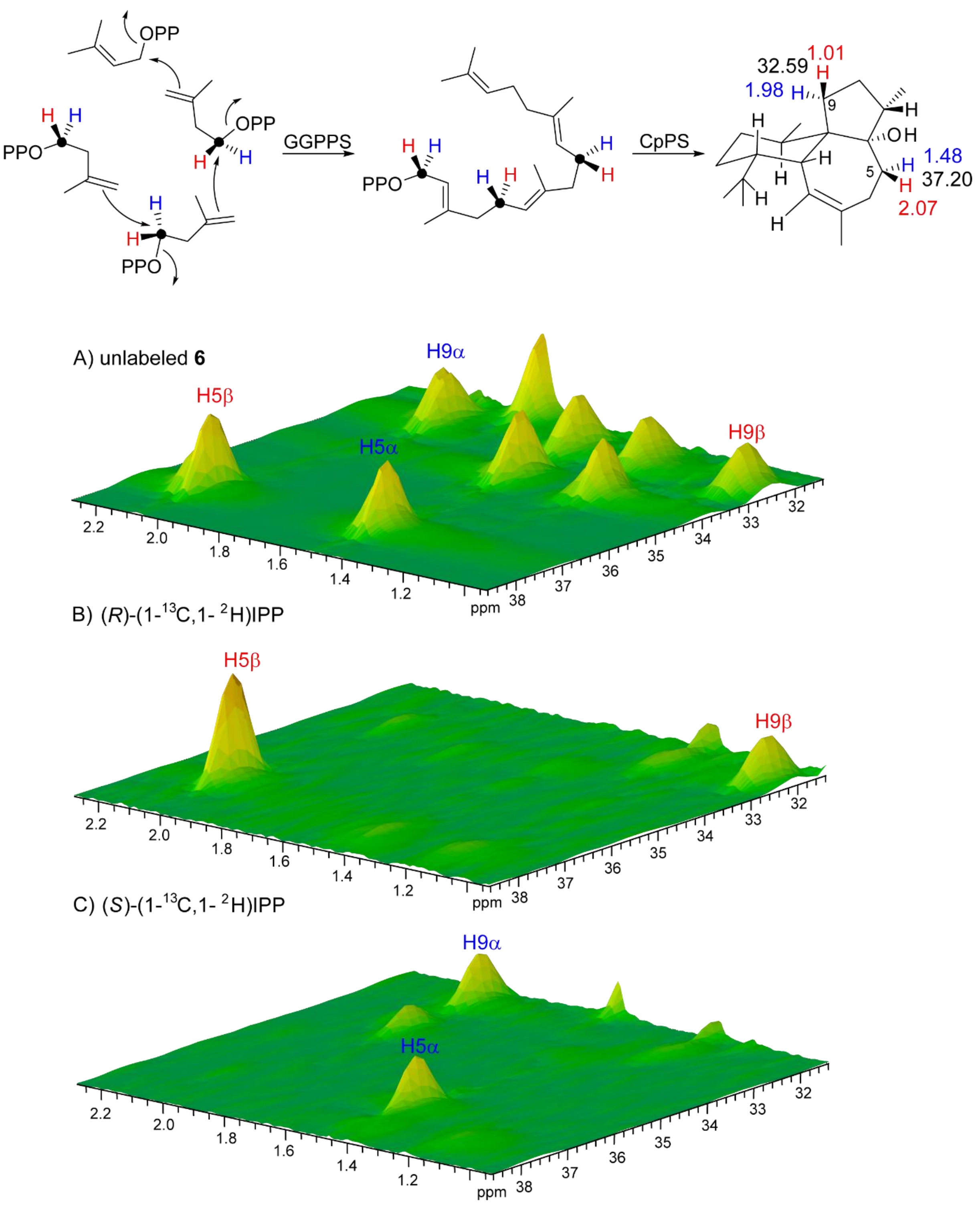

Figure S102. The absolute configuration of **6**. Partial HSQC spectra of A) unlabeled **6**, B) labeled **6** obtained from DMAPP and (*R*)-(1-^{13}C,1-^{2}H)IPP (blue H = ^{2}H), and C) labeled **6** from DMAPP and (*S*)-(1-^{13}C,1-^{2}H)IPP (red H = ^{2}H). The specific incorporation at C5 and C9 with known configuration at this carbon in experiments B) and C) together with the NOESY based assignments of relative orientations of H5α, H5β, H9α and H9β (Figure S69) with respect to the naturally present stereogenic centers in **6** allows to assign the shown absolute configuration for **6**.

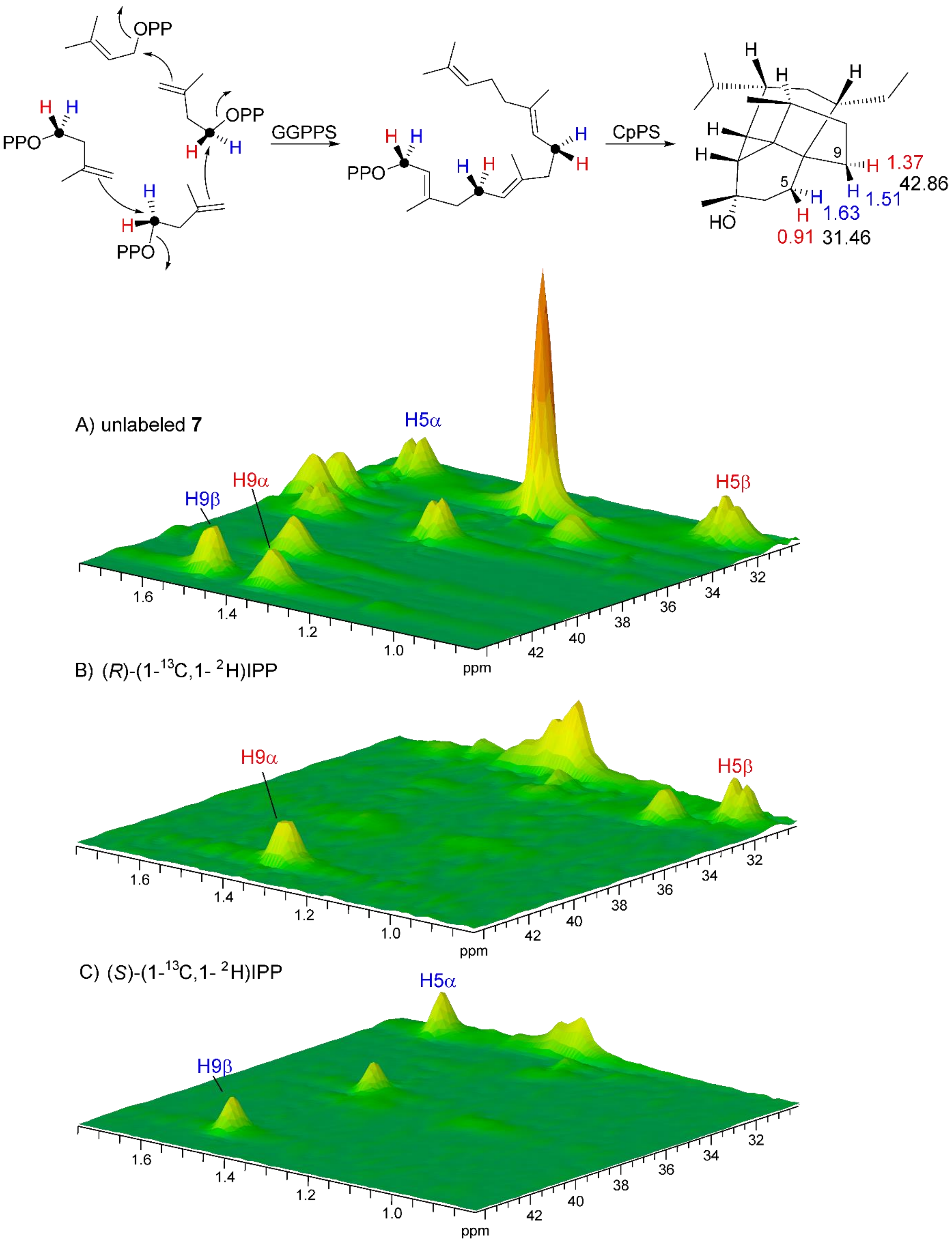

Figure S103. The absolute configuration of **7**. Partial HSQC spectra of A) unlabeled **7**, B) labeled **7** obtained from DMAPP and (*R*)-(1-^{13}C,1-^{2}H)IPP (blue H = ^{2}H), and C) labeled **7** from DMAPP and (*S*)-(1-^{13}C,1-^{2}H)IPP (red H = ^{2}H). The specific incorporation at C5 and C9 with known configuration at this carbon in experiments B) and C) together with the NOESY based assignments of relative orientations of H5α, H5β, H9α and H9β (Figure S77) with respect to the naturally present stereogenic centers in **7** allows to assign the shown absolute configuration for **7**.

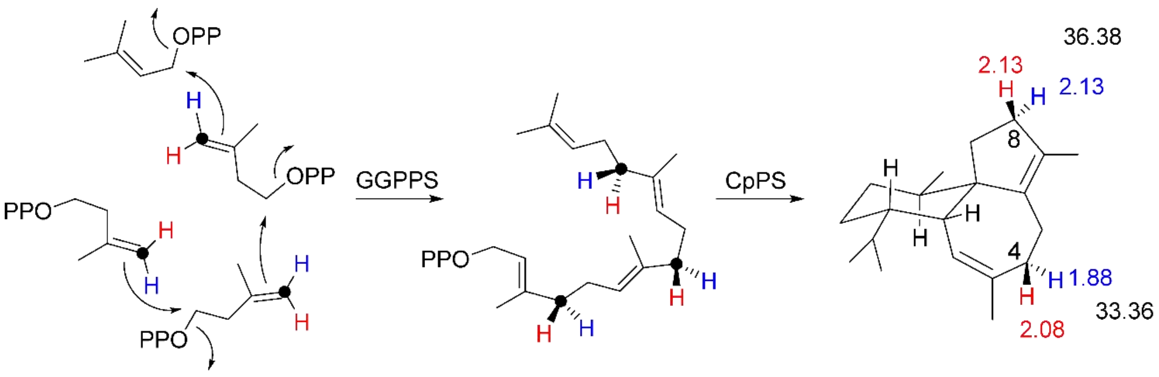

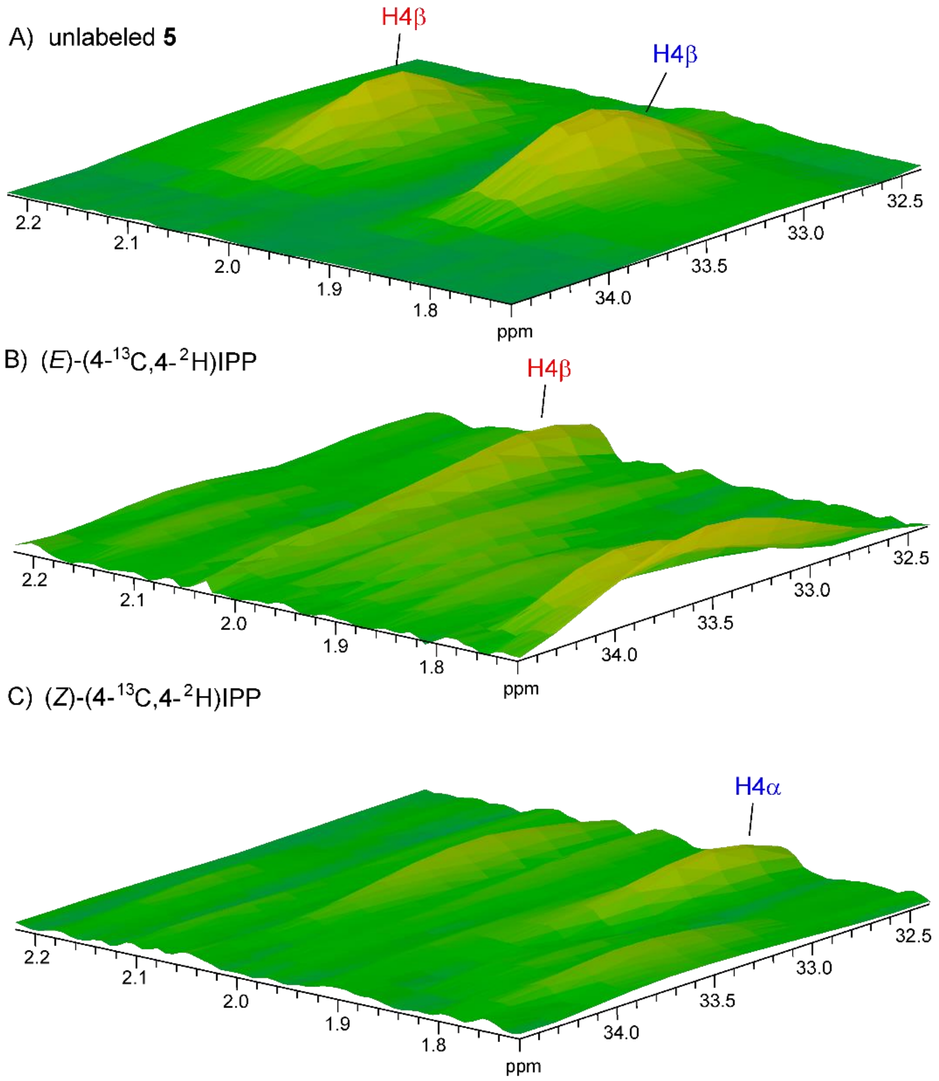

Figure S104. The absolute configuration of **5**. Partial HSQC spectra of A) unlabeled **5**, B) labeled **5** obtained from DMAPP and (*E*)-(4-^{13}C,4-^{2}H)IPP (blue H = ^{2}H), and C) labeled **5** from DMAPP and (*Z*)-(4-^{13}C,4-^{2}H)IPP (red H = ^{2}H). The specific incorporation at C4 with known configuration at this carbons in experiments B) and C) together with the NOESY based assignments of relative orientations of H4α and H4β (Figure S61) with respect to the naturally present stereogenic centers in **5** allows to assign the shown absolute configuration for **5**. Signals for H8α and H8β cannot be used for this assignment, because they show the same chemical shift.

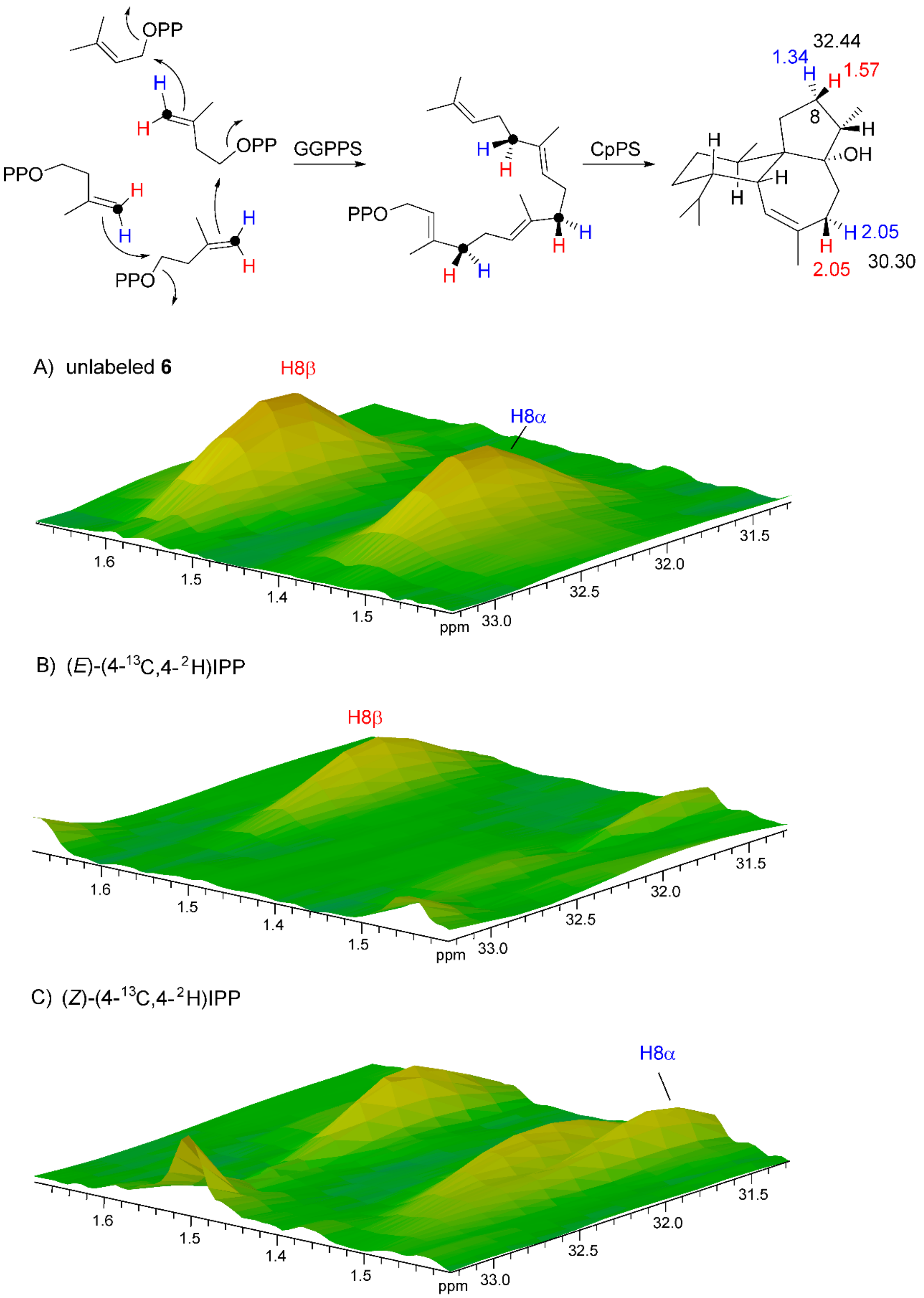

Figure S105. The absolute configuration of **6**. Partial HSQC spectra of A) unlabeled **6**, B) labeled **6** obtained from DMAPP and (*E*)-(4-^{13}C,4-^{2}H)IPP (blue H = ^{2}H), and C) labeled **6** from DMAPP and (*Z*)-(4-^{13}C,4-^{2}H)IPP (red H = ^{2}H). The specific incorporation at C8 with known configuration at this carbons in experiments B) and C) together with the NOESY based assignments of relative orientations of H8α and H8β (Figure S69) with respect to the naturally present stereogenic centers in **6** allows to assign the shown absolute configuration for **6**. Signals for H4α and H4β cannot be used for this assignment, because they show the same chemical shift.

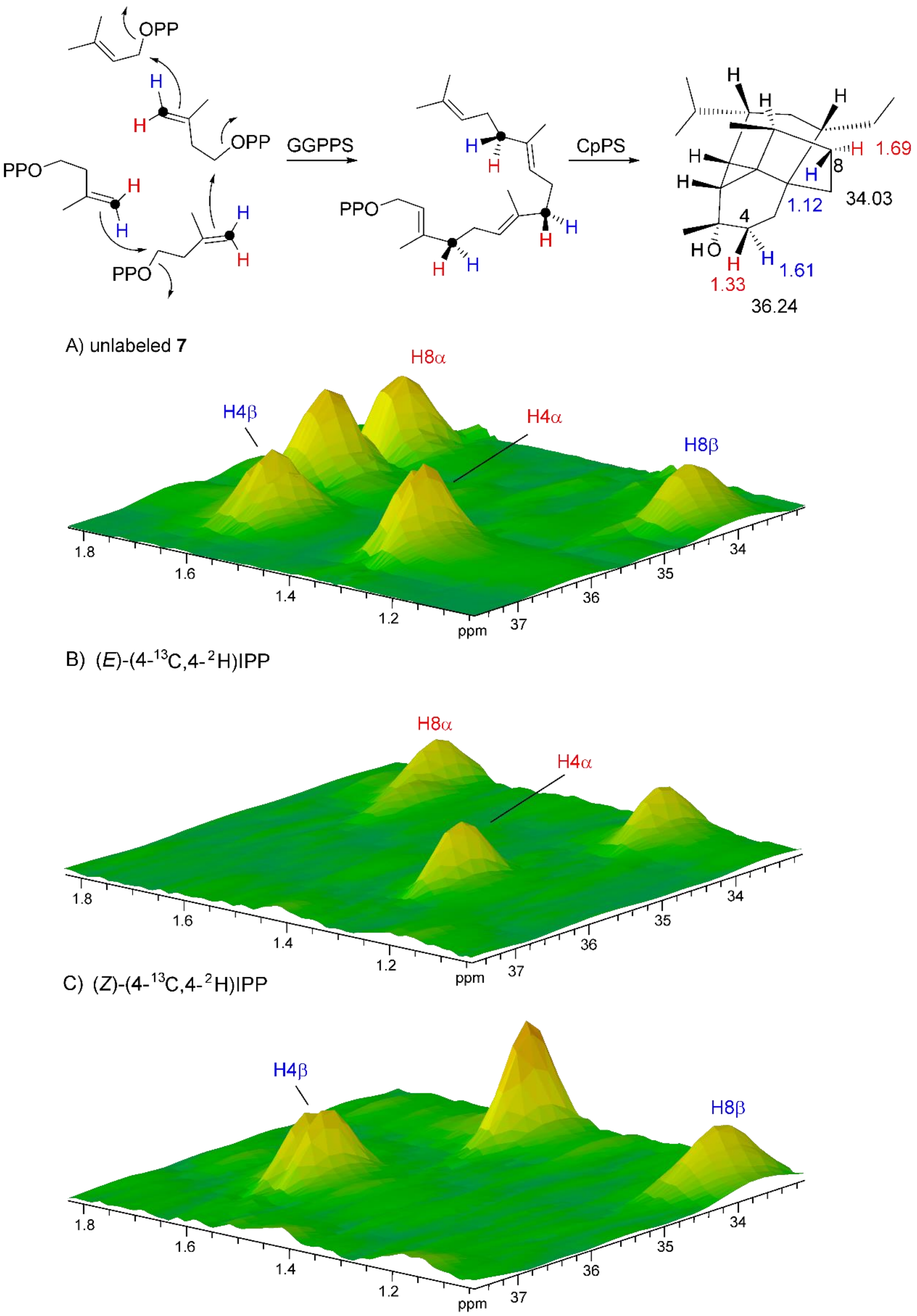

Figure S106. The absolute configuration of **7**. Partial HSQC spectra of A) unlabeled **7**, B) labeled **7** obtained from DMAPP and (*E*)-(4-^{13}C,4-^{2}H)IPP (blue H = ^{2}H), and C) labeled **7** from DMAPP and (*Z*)-(4-^{13}C,4-^{2}H)IPP (red H = ^{2}H). The specific incorporation at C4 and C8 with known configuration at this carbons in experiments B) and C) together with the NOESY based assignments of relative orientations of H4α, H4β, H8α and H8β (Figure S77) with respect to the naturally present stereogenic centers in **7** allows to assign the shown absolute configuration for **7**.

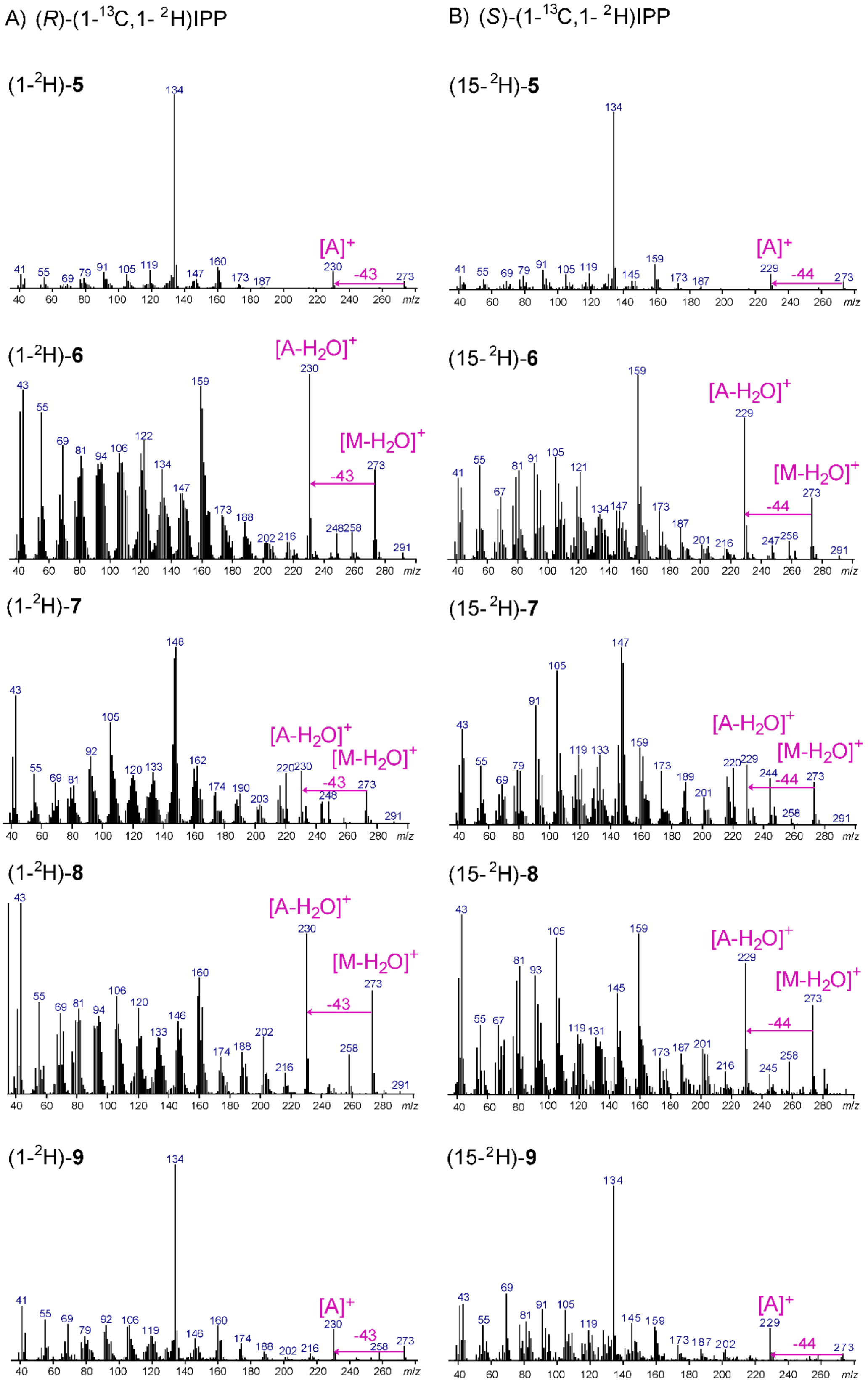

Figure S107. The absolute configurations of **5** – **9**. A) Mass spectra of labeled **5** – **9** obtained from (*R*)-(1-^{2}H)GGPP. B) Mass spectra of labeled **5** – **9** obtained from (*S*)-(1-^{2}H)GGPP. The fragment ion [A]$^{+}$ arises by cleavage of an *i*Pr group, showing retainment of deuterium from (*R*)-(1-^{2}H)GGPP and loss of deuterium from (*S*)-(1-^{2}H)GGPP. These findings are explainable through the stereochemical course for the steps from GGPP to **B** and thus point to the shown absolute configurations of **5** – **9** as in Scheme 1 of main text.

Computational methods

All computed structures were geometry optimized without restrictions and were characterized as minima or as transition state structures by frequency analyses, also providing Gibbs corrections, using the B97D3/6-31g(d,p) method with the density fitting approximation for s- and p-functions, including Grimme's empirical D3-dispersion correction[86] in Gaussian16.[87] For improved single point energies, the mPW1PW91 functional was applied with the 6-311+G(d,p) basis set without density fitting and the ultra-fine integration grid, as this method was shown to be very reliable for examining carbocation cyclization and rearrangement reactions.[1,88-91] The Gibbs corrections include an entropic quasi-harmonic treatment with a frequency cut-off value of 100.0 wavenumbers, according to Grimme, using a mixture of RRHO and free-rotor vibrational entropies.[92,93] Computational data are summarized in Table S15 and visualized in Figure S108.

Table S15. Results of DFT calculations.

Structure	Single Point Energy Hartree	Gibbs Correction Hartree	plus free $MeNH_2$ Hartree[a]	relative to A kcal/mol
free $MeNH_2$	–95,8638271	0,000002	–95,8638251	
A	–781,6974804	0,003006	–877,5582995	0,00
A-TS	–781,6911868	0,002695	–877,5523169	+3,75
B	–781,7229856	0,003447	–877,5833637	–15,73
B1-TS	–781,7070867	0,002330	–877,5685818	–6,45
C1	–781,7107792	0,002191	–877,5724133	–8,86
C1-TS	–781,7044879	0,002012	–877,5663010	–5,02
D1	–781,7227487	0,002173	–877,5844008	–16,38
D1-TS	–781,7261909	0,001855	–877,5881610	–18,74
E1	–781,7452862	0,001803	–877,6073083	–30,75
E1-TS	–781,7436479	0,001958	–877,6055150	–29,63
F1	–781,7558499	0,001852	–877,6178230	–37,35
B2-TS	–781,7054750	0,001845	–877,5674551	–5,75
C2	–781,7121614	0,001929	–877,5740575	–9,89
C2-TS	–781,6832687	0,002390	–877,5447038	+8,53
D2	–781,7269630	0,001727	–877,5890611	–19,30
D2-TS	–781,7307180	0,001838	–877,5927051	–21,59
E2	–781,7363630	0,002099	–877,5980891	–24,97
E2-TS	–781,7174990	0,001960	–877,5793641	–13,22
F2	–781,7542540	0,001615	–877,6164641	–36,50
F2-TS	–781,7360482	0,001588	–877,5982853	–25,09
M	–781,7621223	0,001936	–877,6240114	–41,23
M + $MeNH_2$	–877,6398158	0,004491	–877,6353248	–48,33
N-TS	–877,6323156	0,003613	–877,6287026	–44,18
9 + $MeNH_2$	–877,6423468	0,003507	–877,6388398	–50,54
FF-TS	–781,7417822	0,001932	–877,6036753	–28,47
F1-TS (dyR)	–781,6965754	0,002771	–877,5576295	+0,42
F1 + $MeNH_2$	–877,6269998	0,004222	–877,6227778	–40,46
F1-TS	–877,5981357	0,004357	–877,5937787	–22,26
G + $MeNH_2$	–877,6247650	0,005537	–877,6192280	–38,23
G-TS	–877,6060845	0,004844	–877,6012405	–26,95
H + $MeNH_2$	–877,6202126	0,004918	–877,6152946	–35,76
H	–781,7525766	0,002056	–877,6143457	–35,17
H-TS	–781,7496997	0,001859	–877,6116658	–33,49
I	–781,7498291	0,001972	–877,6116822	–33,50
I-TS	–781,7471167	0,001831	–877,6091108	–31,88
J	–781,7623885	0,002225	–877,6239886	–41,22
J-TS	–781,7529481	0,001750	–877,6150232	–35,59
K	–781,7601793	0,002251	–877,6217534	–39,82
K-TS	–781,7619986	0,002161	–877,6236627	–41,02
L	–781,7626213	0,002318	–877,6241284	–41,31

[a] The structures in green were generated with $MeNH_2$ added as a catalytic base. To make the computational data comparable, the single point energy of free $MeNH_2$ with Gibbs correction was added to the energies of all other structures.

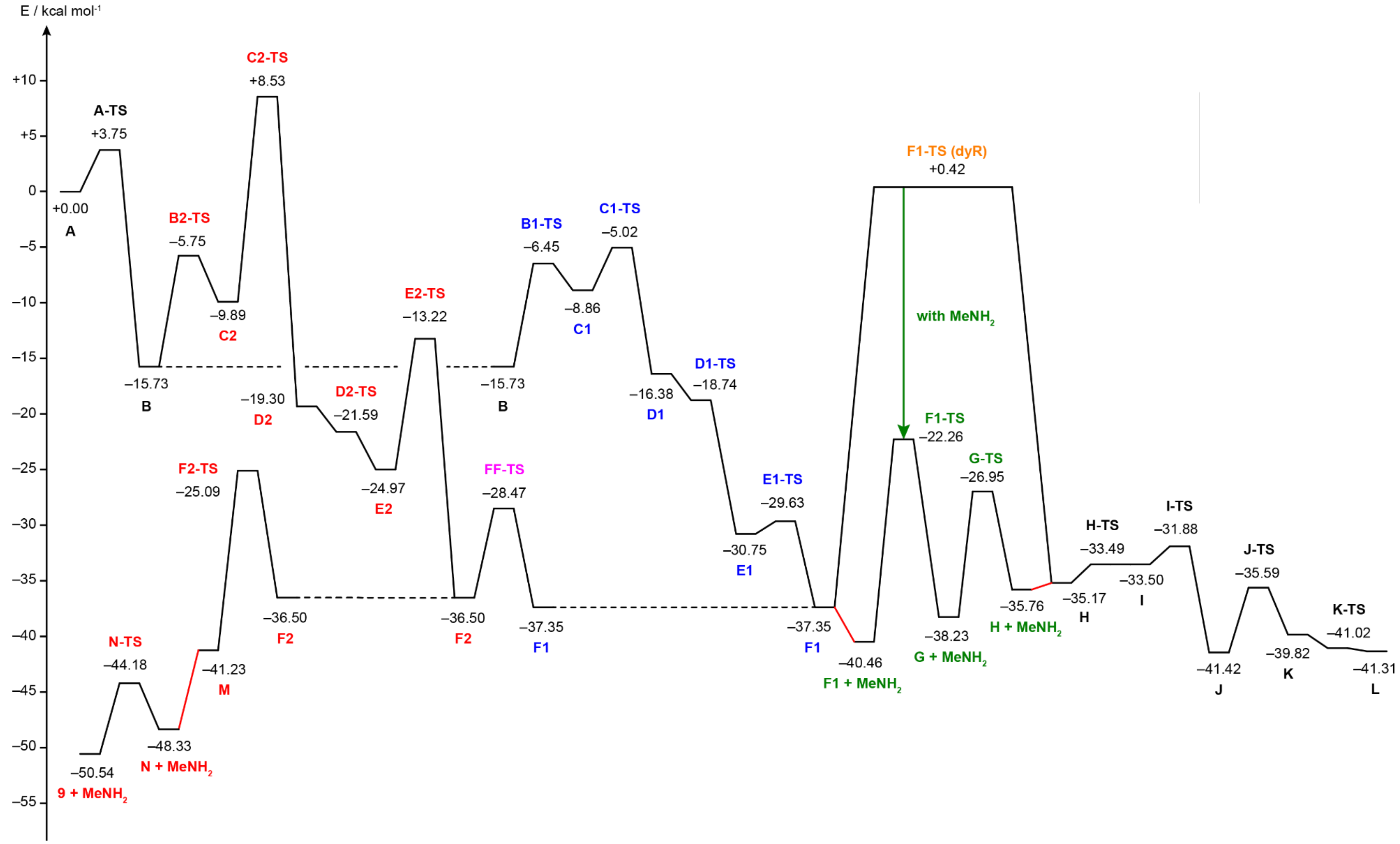

Figure S108. Energy profile for the cyclization mechanism by CpPS as shown in Scheme 1 of main text based on data from DFT calculations (Table S15). The dashed lines connect identical structures. The red lines connect structures with $MeNH_2$ added as an interacting catalytic base and structures without $MeNH_2$, but instead with added single point energy of free $MeNH_2$.

References

[1] L. Lauterbach, B. Goldfuss, J. S. Dickschat, *Angew. Chem. Int. Ed.* **2020**, *59*, 11943.

[2] Y. Yamada, T. Kuzuyama, M. Komatsu, K. Shin-ya, S. Omura, D. E. Cane, H. Ikeda, *Proc. Natl. Acad. Sci. USA* **2015**, *112*, 857.

[3] C. Nakano, S. Horinouchi, Y. Ohnishi, *J. Biol. Chem.* **2011**, *286*, 27980.

[4] L. Lauterbach, J. S. Dickschat, *Org. Biomol. Chem.* **2020**, *18*, 4547.

[5] J. Rinkel, L. Lauterbach, P. Rabe, J. S. Dickschat, *Angew. Chem. Int. Ed.* **2018**, *57*, 3238.

[6] P. Rabe, J. Rinkel, T. A. Klapschinski, L. Barra, J. S. Dickschat, *Org. Biomol. Chem.* **2016**, *14*, 158.

[7] S.-Y. Kim, P. Zhao, M. Igarashi, R. Sawa, T. Tomita, M. Nishiyama, T. Kuzuyama, *Chem. Biol.* **2009**, *16*, 736.

[8] J. Rinkel, P. Rabe, P. Garbeva, J. S. Dickschat, *Angew. Chem. Int. Ed.* **2016**, *55*, 13593.

[9] G. Li, Y.-W. Guo, J. S. Dickschat, *Angew. Chem. Int. Ed.* **2021**, *60*, 1488.

[10] P. Rabe, J. S. Dickschat, *Angew. Chem. Int. Ed.* **2013**, *52*, 1810.

[11] J. Rinkel, S. T. Steiner, J. S. Dickschat, *Angew. Chem. Int. Ed.* **2019**, *58*, 9230.

[12] L. Lauterbach, J. Rinkel, J. S. Dickschat, *Angew. Chem. Int. Ed.* **2018**, *57*, 8280.

[13] P. Rabe, J. Rinkel, E. Dolja, T. Schmitz, B. Nubbemeyer, T. H. Luu, J. S. Dickschat, *Angew. Chem. Int. Ed.* **2017**, *56*, 2776.

[14] P. Baer, P. Rabe, K. Fischer, C. A. Citron, T. A. Klapschinski, M. Groll, J. S. Dickschat, *Angew. Chem. Int. Ed.* **2014**, *53*, 7652.

[15] Y. Hu, W. K. W. Chou, R. Hopson, D. E. Cane, *Chem. Biol.* **2011**, *18*, 32.

[16] P. Rabe, L. Barra, J. Rinkel, R. Riclea, C. A. Citron, T. A. Klapschinski, A. Janusko, J. S. Dickschat, *Angew. Chem. Int. Ed.* **2015**, *54*, 13448.

[17] A. Hou, J. S. Dickschat, *Angew. Chem. Int. Ed.* **2020**, *59*, 19961.

[18] Z. Li, Y. Jiang, X. Zhang, Y. Chang, S. Li, X. Zhang, S. Zheng, C. Geng, P. Men, L. Ma, Y. Yang, Z. Gao, Y.-J. Tang, S. Li, *ACS Catal.* **2020**, *10*, 5846.

[19] C. Nakano, F. Kudo, T. Eguchi, Y. Ohnishi, *ChemBioChem* **2011**, *12*, 2271.

[20] W. K. W. Chou, I. Fanizza, T. Uchiyama, M. Komatsu, H. Ikeda, D. E. Cane, *J. Am. Chem. Soc.* **2010**, *132*, 8850.

[21] D. E. Cane, J. K. Sohng, C. R. Lamberson, S. M. Rudnicki, Z. Wu, M. D. Lloyd, J. S. Oliver, B. R. Hubbard, *Biochemistry* **1994**, *33*, 5846.

[22] P. Rabe, M. Samborskyy, P. F. Leadlay, J. S. Dickschat, *Org. Biomol. Chem.* **2017**, *15*, 2353.

[23] T. A. Klapschinski, P. Rabe, J. S. Dickschat, *Angew. Chem. Int. Ed.* **2016**, *55*, 10141.

[24] J. Rinkel, J. S. Dickschat, *ChemBioChem* **2020**, *21*, 807.

[25] J. Rinkel, J. S. Dickschat, *Org. Lett.* **2019**, *21*, 9442.

[26] X. Lin, R. Hopson, D. E. Cane, *J. Am. Chem. Soc*. **2006**, *128*, 6022.

[27] S. A. Agger, F. Lopez-Gallego, T. R. Hoye, C. Schmidt-Dannert, *J. Bacteriol.* **2008**, *190*, 6084.

[28] J. S. Dickschat, J. Rinkel, P. Rabe, A. Beyraghdar Kashkooli, H. J. Bouwmeester, *Beilstein J. Org. Chem.* **2017**, *13*, 1770.

[29] J. Rinkel, J. S. Dickschat, *Org. Lett.* **2019**, *21*, 2426.

[30] J. S. Dickschat, K. A. K. Pahirulzaman, P. Rabe, T. A. Klapschinski, *ChemBioChem* **2014**, *15*, 810.

[31] A. Schifrin, T. T. B. Ly, N. Günnewich, J. Zapp, V. Thiel, S. Schulz, F. Hannemann, Y. Khatri, R. Bernhardt, *ChemBioChem* **2015**, *16*, 337.

[32] C. Nakano, T. Tezuka, S. Horinouchi, Y. Ohnishi, *J. Antibiot.* **2012**, *65*, 551.

[33] J. Rinkel, L. Lauterbach, J. S. Dickschat, *Angew. Chem.* **2017**, *129*, 16603.

[34] H. Xu, J. Rinkel, J. S. Dickschat, *Org. Chem. Front.* **2021**, *8*, 1177.

[35] P. Rabe, K. A. K. Pahirulzaman, J. S. Dickschat, *Angew. Chem. Int. Ed.* **2015**, *54*, 6041.

[36] Y. H. Zhao, M. H. Abraham, A. M. Zissimos, *J. Org. Chem.* **2003**, *68*, 7368.

[37] C. Nakano, H.-K. Kim, Y. Ohnishi, *ChemBioChem* **2011**, *12*, 1988.
[38] J. J. Shaw, T. Berbasova, T. Sasaki, K. Jefferson-George, D. J. Spakowicz, B. F. Dunican, C. E. Portero, A. Narvaez-Trujillo, S. A. Strobel, *J. Biol. Chem.* **2015**, *290*, 8511.
[39] J. Rinkel, J. S. Dickschat, *Beilstein J. Org. Chem.* **2019**, *15*, 789.
[40] J. Rinkel, J. S. Dickschat, *Beilstein J. Org. Chem.* **2019**, *15*, 1008.
[41] K. Murai, L. Lauterbach, K. Teramoto, Z. Quan, L. Barra, T. Yamamoto, K. Nonaka, K. Shiomi, M. Nishiyama, T. Kuzuyama, J. S. Dickschat, *Angew. Chem. Int. Ed.* **2019**, *58*, 15046.
[42] A. Schifrin, Y. Khatri, P. Kirsch, V. Thiel, S. Schulz, R. Bernhardt, *Org. Biomol. Chem.* **2016**, *14*, 3385.
[43] C. Pinedo, C.-M. Wang, J.-M. Pradier, B. Dalmais, M. Choquer, P. Le Pecheur, G. Morgan, I. G. Collado, D. E. Cane, M. Viaud, *ACS Chem. Biol.* **2008**, *3*, 791.
[44] N. L. Brock, K. Huss, B. Tudzynski, J. S. Dickschat, *ChemBioChem* **2013**, *14*, 311.
[45] D. E. Cane, I. Kang, *Arch. Biochem. Biophys.* **2000**, *376*, 354.
[46] I. Burkhardt, T. Siemon, M. Henrot, L. Studt, S. Rösler, B. Tudzynski, M. Christmann, J. S. Dickschat, *Angew. Chem. Int. Ed.* **2016**, *55*, 8748.
[47] C. Nakano, H.-K. Kim, Y. Ohnishi, *ChemBioChem* **2011**, *12*, 2403.
[48] D. E. Cane, S. Swanson, P. P. N. Murthy, *J. Am. Chem. Soc.* **1981**, *103*, 2136.
[49] P. Rabe, J. Rinkel, B. Nubbemeyer, T. G. Köllner, F. Chen, J. S. Dickschat, *Angew. Chem. Int. Ed.* **2016**, *55*, 15420.
[50] H. Xu, J. Rinkel, X. Chen, T. G. Köllner, F. Chen, J. S. Dickschat, *Org. Biolmol. Chem.* **2021**, *19*, 370.
[51] I. Burkhardt, N. Kreuzenbeck, C. Beemelmanns, J. S. Dickschat, *Org. Biomol. Chem.* **2019**, *17*, 3348.
[52] G. Bian, A. Hou, Y. Yuan, B. Hu, S. Cheng, Z. Ye, Y. Di, Z. Deng, T. Liu, *Org. Lett.* **2018**, *20*, 1626.
[53] J. Rinkel, T. G. Köllner, F. Chen, J. S. Dickschat, *ChemComm* **2019**, *55*, 13255.
[54] E.-M. Niehaus, J. Schumacher, I. Burkhardt, P. Rabe, M. Münsterkötter, U. Güldener, J. S. Dickschat, B. Tudzynski, *Front. Microbiol.* **2017**, *8*, 1175.
[55] P. Baer, P. Rabe, C. A. Citron, C. C. de Oliveira Mann, N. Kaufmann, M. Groll, J. S. Dickschat, *ChemBioChem* **2014**, *15*, 213.
[56] J. Rinkel, L. Lauterbach, J. S. Dickschat, *Angew. Chem. Int. Ed.* **2019**, *58*, 452.
[57] G. Bian, J. Rinkel, Z. Wang, L. Lauterbach, A. Hou, Y. Yuan, Z. Deng, T. Liu, J. S. Dickschat, *Angew. Chem. Int. Ed.* **2018**, *57*, 15887.
[58] A. Meguro, T. Tomita, M. Nishiyama, T. Kuzuyama, *ChemBioChem* **2013**, *14*, 316.
[59] J. Rinkel, P. Rabe, X. Chen, T. G. Köllner, F. Chen, J. S. Dickschat, *Chem. Eur. J.* **2017**, *23*, 10501.
[60] J. Rinkel, S. T. Steiner, G. Bian, R. Chen, T. Liu, J. S. Dickschat, *ChemBioChem* **2020**, *21*, 486.
[61] T. Toyomasu, A. Kaneko, T. Tokiwano, Y. Kanno, Y. Kanno, R. Niida, S. Miura, T. Nishioka, C. Ikeda, W. Mitsuhashi, T. Dairi, T. Kawano, H. Oikawa, N. Kato, T. Sassa, *J. Org. Chem*. **2009**, *74*, 1541.
[62] F. L. Lin, L. Lauterbach, J. Zhou, Y. H. Wang, J. M. Lv, G. D. Chen, D. Hu, H. Gao, X. S. Yao, J. S. Dickschat, *ACS Catal.* **2020**, *10*, 4306.
[63] T. Toyomasu, M. Tsukahara, A. Kaneko, R. Niida, W. Mitsuhashi, T. Dairi, N. Kato, T. Sassa, *Proc. Natl. Acad. Sci. USA* **2007**, *104*, 3084.
[64] F. Kudo, Y. Matsuura, T. Hayashi, M. Fukushima, T. Eguchi, *J. Antibiot.* **2016**, *69*, 541.
[65] T. Mitsuhashi, T. Kikuchi, S. Hoshino, M. Ozeki, T. Awakawa, S. Shi, M. Fujita, I. Abe, *Org. Lett*. **2018**, *20*, 5606.
[66] A. Hou, L. Lauterbach, J. S. Dickschat, *Chem. Eur. J.* **2020**, *26*, 2178.
[67] Y. Matsuda, T. Mitsuhashi, Z. Quan, I. Abe, *Org. Lett*. **2015**, *17*, 4644.

[68] Z. Quan, J. S. Dickschat, *Org. Lett.* **2020**, *22*, 7552.
[69] K. Narita, H. Sato, A. Minami, K. Kudo, L. Gao, C. Liu, T. Ozaki, M. Kodama, X. Lei, T. Taniguchi, K. Monde, M. Yamazaki, M. Uchiyama, H. Oikawa, *Org. Lett*. **2017**, *19*, 6696.
[70] Y. Ye, A. Minami, A. Mandi, C. Liu, T. Taniguchi, T. Kuzuyama, K. Monde, K. Gomi, H. Oikawa, *J. Am. Chem. Soc.* **2015**, *137*, 11846.
[71] G. Bian, Y. Han, A. Hou, Y. Yuan, X. Liu, Z. Deng, T. Liu, *Metab. Eng.* **2017**, *42*, 1.
[72] M. Okada, Y. Matsuda, T. Mitsuhashi, S. Hoshino, T. Mori, K. Nakagawa, Z. Quan, B. Qin, H. Zhang, F. Hayashi, H. Kawaide, I. Abe, *J. Am. Chem. Soc.* **2016**, *138*, 10011.
[73] R. Chiba, A. Minami, K. Gomi, H. Oikawa, *Org. Lett*. **2013**, *15*, 594.
[74] T. Mitsuhashi, J. Rinkel, M. Okada, I. Abe, J. S. Dickschat, *Chem. Eur. J.* **2017**, *23*, 10053.
[75] J. Guo, Y.-S. Cai, F. Cheng, C. Yang, W. Zhang, W. Yu, J. Yan, Z. Deng, K. Hong, *Org. Lett.* **2021**, *23*, 1525.
[76] Y. Matsuda, T. Mitsuhashi, S. Lee, M. Hoshino, T. Mori, M. Okada, H. Zhang, F. Hayashi, M. Fujita, I. Abe, *Angew. Chem. Int. Ed.* **2016**, *55*, 5785.
[77] I. J. O. Jondiko, G. Pattenden, *Phytochemistry* **1989**, *28*, 3159.
[78] C. Nakano, T. Okamura, T. Sato, T. Dairi, T. Hoshino, *Chem. Commun.* **2005**, 1016.
[79] G. R. Fulmer, A. J. M. Miller, N. H. Sherden, H. E. Gottlieb, A. Nudelman, B. M. Stoltz, J. E. Bercaw, K. I. Goldberg, *Organometallics* **2010**, *29*, 2176.
[80] S. Carmely, A. Groweiss, Y. Kashman, *J. Org. Chem.* **1981**, *46*, 4279.
[81] Z. Quan, J. S. Dickschat, *Org. Biomol. Chem.* **2020**, *18*, 6072.
[82] R. Higuchi, B. Krummel, R. Saiki, *Nucleic Acids Res.* **1988**, *16*, 7351.
[83] R. D. Giets, R. H. Schiestl, *Nat. Protoc.* **2007**, *2*, 31.
[84] J. S. Dickschat, K. A. K. Pahirulzaman, P. Rabe, T. A. Klapschinski, *ChemBioChem* **2014**, *15*, 810.
[85] M. M. Bradford, *Anal. Biochem.* **1976**, *72*, 248.
[86] S. Grimme, S. Ehrlich, L. Goerigk, *J. Comp. Chem.* **2011**, *32*, 1456.
[87] Gaussian 16, Revision B.01, M. J. Frisch, G. W. Trucks, H. B. Schlegel, G. E. Scuseria, M. A. Robb, J. R. Cheeseman, G. Scalmani, V. Barone, G. A. Petersson, H. Nakatsuji, X. Li, M. Caricato, A. V. Marenich, J. Bloino, B. G. Janesko, R. Gomperts, B. Mennucci, H. P. Hratchian, J. V. Ortiz, A. F. Izmaylov, J. L. Sonnenberg, D. Williams-Young, F. Ding, F. Lipparini, F. Egidi, J. Goings, B. Peng, A. Petrone, T. Henderson, D. Ranasinghe, V. G. Zakrzewski, J. Gao, N. Rega, G. Zheng, W. Liang, M. Hada, M. Ehara, K. Toyota, R. Fukuda, J. Hasegawa, M. Ishida, T. Nakajima, Y. Honda, O. Kitao, H. Nakai, T. Vreven, K. Throssell, J. A. Montgomery, Jr., J. E. Peralta, F. Ogliaro, M. J. Bearpark, J. J. Heyd, E. N. Brothers, K. N. Kudin, V. N. Staroverov, T. A. Keith, R. Kobayashi, J. Normand, K. Raghavachari, A. P. Rendell, J. C. Burant, S. S. Iyengar, J. Tomasi, M. Cossi, J. M. Millam, M. Klene, C. Adamo, R. Cammi, J. W. Ochterski, R. L. Martin, K. Morokuma, O. Farkas, J. B. Foresman, D. J. Fox, Gaussian, Inc., Wallingford CT, **2016**.
[88] Y. J. Hong, D. J. Tantillo, *J. Org. Chem.* **2018**, *83*, 3780.
[89] C. Adamo, V. Barone, *J. Chem. Phys.* **1998**, *108*, 664.
[90] S. P. T. Matsuda, W. K. Wilson, Q. Xiong, *Org. Biomol. Chem.* **2006**, *4*, 530.
[91] H. Xu, B. Goldfuss, J. S. Dickschat, *Chem. Eur. J.* **2021**, *27*, 9758.
[92] S. Grimme, *Chem. Eur. J.* **2012**, *18*, 9955.
[93] GoodVibes v3.0.1, G. Luchini, J. V. Alegre-Requena, Y. Guan, I. Funes-Ardoiz, R. S. Paton, **2019**.
